# Enantiopure β-isocyano-boronic esters: synthesis and exploitation in isocyanide-based multicomponent reactions

**DOI:** 10.1007/s11030-022-10549-8

**Published:** 2022-10-19

**Authors:** Marco Manenti, Simone Gusmini, Leonardo Lo Presti, Giorgio Molteni, Alessandra Silvani

**Affiliations:** Dipartimento di Chimica, Università degli Studi di Milano, Via Golgi 19, 20133 Milan, Italy

**Keywords:** Isocyanides, Boronic acids, Multicomponent reactions, Peptidomimetics, Heterocycles

## Abstract

Various boron-containing isocyanides have been efficiently synthesized from the corresponding enantiopure β-substituted β-amino boronic acid pinacol esters, without need for protecting group interconversion, through a two-step, purification-free procedure. They were employed in a variety of isocyanide-based multicomponent reactions, proving to be reliable components for all of them and allowing the efficient synthesis of unprecedented, boron-containing peptidomimetics and heteroatom-rich small molecules, including biologically relevant cyclic boronates. Jointing together the β-amido boronic acid moiety, deriving from the isocyanide component, with prominent pharmacophoric rings emerging from the multicomponent process, a successful application of the molecular hybridization concept could be realized.

**Graphical abstract:**

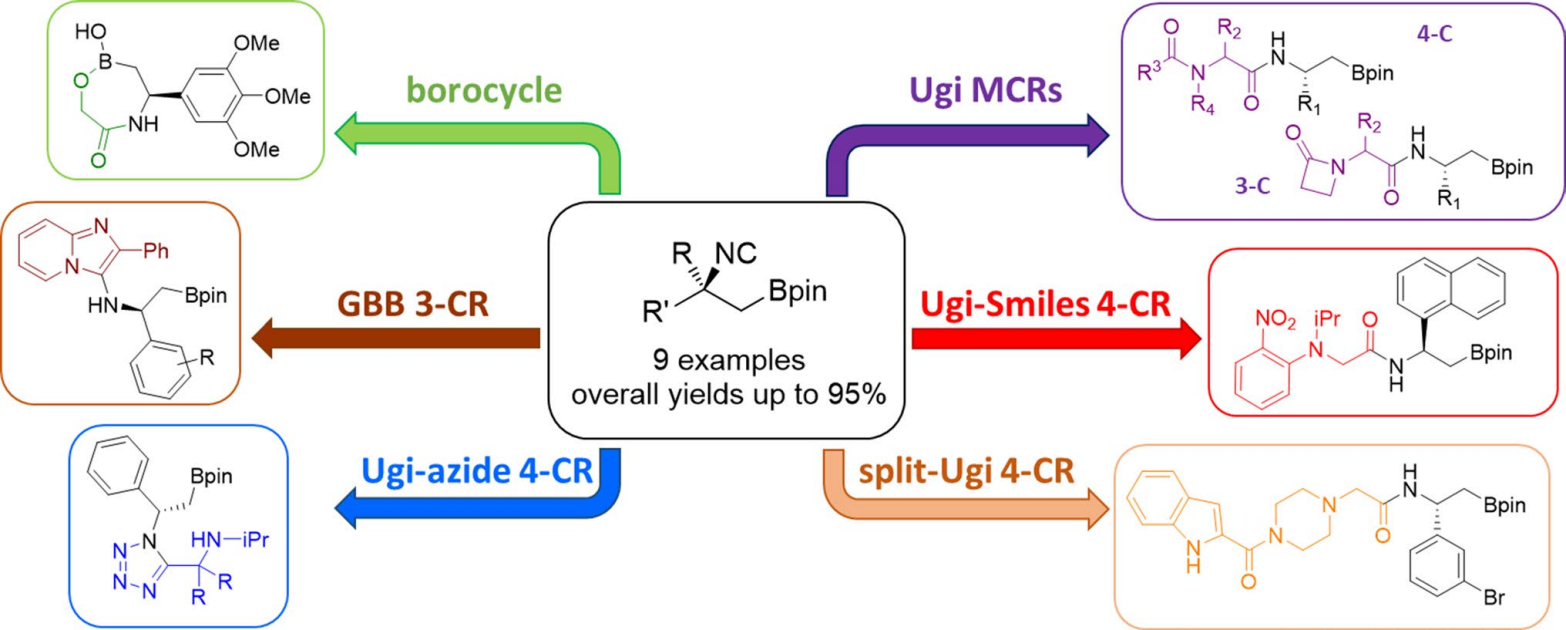

**Supplementary Information:**

The online version contains supplementary material available at 10.1007/s11030-022-10549-8.

## Introduction

The rather unusual electronic structure of isocyanides has attracted considerable attention in organic chemistry, even because many isocyanides are thermally, hydrolytically, and aerobically stable, thus they can be easily isolated and employed in chemical reactions [1]. The outstanding reactivity of the isocyanide functional group includes the ability to react with both electrophiles and nucleophiles simultaneously, and places isocyanide-containing compounds as privileged building blocks for synthetic, medicinal, and material chemistry applications. In particular, their compatibility with domino and cascade processes has paved the way for developing various isocyanide-based multicomponent strategies, which benefit from high degree of atom and bond economy and require relatively mild reaction conditions and simplicity of work up procedures [2–5].

Nowadays, isocyanide-based multicomponent reactions (IMCRs) play a prominent role in drug discovery due to their robustness and scope, allowing the synthesis of complex biologically relevant compounds in a one-pot domino process. [6, 7]. Among IMCRs, long standing Ugi four-component reaction (U-4CR) leads to diversely substituted peptidomimetic backbones starting from abundantly available building blocks and, in combination with subsequent cyclization reactions, has become a valuable tool in the design and synthesis of cyclic constrained peptidomimetics and heterocyclic compounds [8, 9], also contributing in recent years to the emergence of many important drugs for the treatment of several diseases [10]. By tactical application of multi-functionalized building blocks and combination with other prominent synthetic strategies, such as ring closing metathesis [11] or Pd-catalyzed reactions [12], high molecular diversity can be rapidly achieved by IMCRs [13], establishing their relevance also in the total synthesis of complex natural products [14]. Finally, IMCRs offer multiple possibilities for polymer chemistry, as demonstrated with the synthesis of monomers and grafting-onto reactions, as well as direct polymerizations [15].

However, despite the successful application in the preparation of large arrays of functional compounds, some limitations of the IMCRs are not still overcame, most of them related to the isocyanide component. Besides the unpleasant odor, the relatively high price and the sensitivity in acidic medium, the main drawback of isocyanide-containing compounds is their difficult functionalization, which also limits the functional groups variability in peptidomimetic, heterocyclic and natural products targets. Starting from this consideration and going on with our diversity-oriented synthetic chemistry programs, focused on both IMCRs [16–24] and boron-containing compounds [25, 26], we considered aliphatic isocyano boronic acids as attractive components, able to greatly increase IMCRs products scope, as better well-known boryl aldehydes [27, 28] and amino boronic acids [29] already do. Boron-containing peptidomimetics are currently highly relevant in drug discovery, mainly due to the ability of amino boronic acids to act as amino acid bioisosteres and reversible covalent inhibitors [30]. Their demonstrated mechanisms of interaction with nucleophilic groups in biomolecules underlie the use of such compounds in medicinal chemistry, resulting in many boron-containing drugs approved by FDA in the last few years [31].

After the preliminary work of van Leusen in 1995 [32], stable α-boryl isocyanides, derived from the corresponding α-boryl isocyanates, were introduced for the first time by Professor Yudin et al. as racemic compounds [33, 34]. Their potential was fully demonstrated in U-4CRs, allowing the identification of new bioactive inhibitors of human caseinolytic protease P (hClpP) [35]. Quite recently, similar α-boryl isocyanides are also reported in enantiopure form [36]. In general, stability of such compounds is likely guaranteed through tetracoordination at the boron atom, by means of the MIDA (N-methyliminodiacetyl) protecting group.

**Fig. 1:**
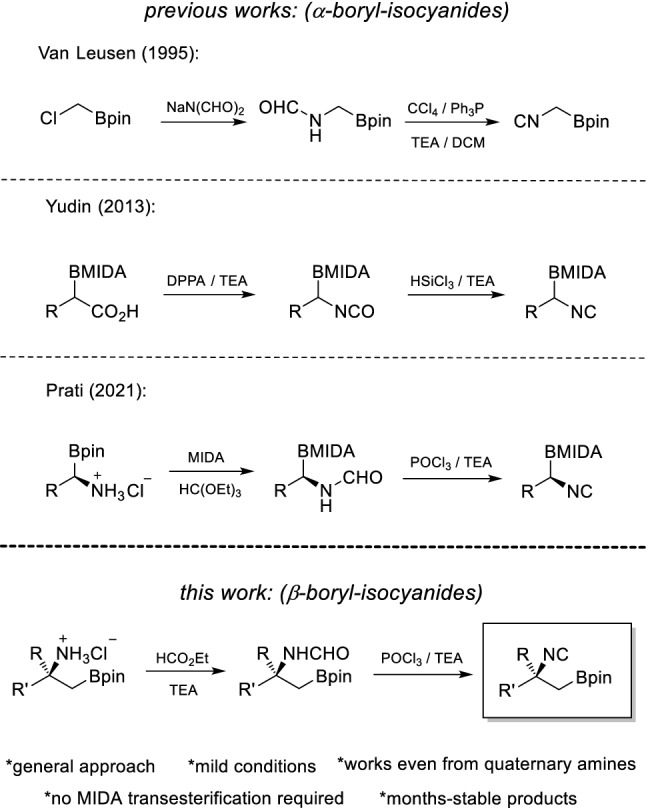
Synthesis of isocyano boronic acid derivatives

However, the interconversion of pinacol ester with the MIDA protecting group requires harsh conditions, such as high temperature generally under acidic conditions, so the functional groups tolerance of this reaction is quite low. Being well known that β-amino boronic acids are far more stable than α-ones, we reasoned that the same principle should work for α- and β-boryl isocyanides. Aiming to a general procedure that doesn’t require the MIDA protection of the boron atom and relying on our experience in the field of β-amino boronic acids derivatives [26], we envisioned β-boryl isocyanides as promising target compounds, potentially more stable if compared to α-ones and, as such, suitable to be involved in various, multicomponent isocyanide-based processes (Fig. 1). To the best of our knowledge, such boron-containing isocyanides are unprecedented. We report herein their efficient two-step preparation starting from enantiopure β-substituted β-amino boronates [37], as well as their application to the one-pot, multicomponent synthesis of boron-containing peptidomimetic and heterocyclic small molecules.

## Results and discussion

Because of the wide applicability of the protocol, the dehydration of formamides is recognized as the most general method for the preparation of isocyanides. Starting from this consideration, we began our investigation using the known (*S*)-β-phenyl β-amino boronate hydrochloride **1a** and considering its conversion to the corresponding formamide **2a** (Table 1).

**Table 1:** Reaction conditions for the formylation of **1a** to give intermediate **2a** and for the subsequent dehydration to **3a**
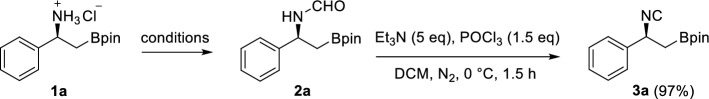

Entry	Reagent	Solvent (0.2 M)	Base	Temp. (°C)	Time (h)	Yield %^a^ (**2a**)
1	–	HCO_2_Me or HC(OEt)_3_	–	Reflux	24	n.d
2	HCO_2_Et (1 eq)	DMF	–	90	6	35
3	HCO_2_Et (1 eq)	DMF	–	150	6	33
4	HCO_2_Et (1 eq)	DMF	Et_3_N	90	6	62
5	Formic acid, HBTU	DCM	Et_3_N	r.t	12	42
6	–	HCO_2_Et/DMF 3:2	Et_3_N	90	3	97

^a^Isolated yields

Performing the reaction in methyl formate or, alternatively, in triethoxy methane, used as reagent and solvent, no conversion was observed, likely due to the poor solubility of **1a**, even at high temperature (entry 1). Switching to DMF as solvent and ethyl formate as formylating agent, the desired product **2a** can be isolated in a 35% yield (entry 2). Increasing temperature from 90 to 150 °C had no significant influence on the reaction (entry 3), while the addition of a stochiometric amount of triethylamine proved to be beneficial for the reaction (entry 4). The use of formic acid with a condensing agent (HBTU) in dichloromethane afforded the product in lower yield (entry 5). Finally, performing the reaction in a 3:2 mixture of ethyl formate and DMF, an almost quantitative formylation could be achieved, with product **2a** obtained in 97% isolated yield, after 3 h (entry 6). Subsequent dehydration to give the target (*S*)-β-phenyl-β-isocyano boronic ester **3a** was straightforward. Using phosphoryl oxychloride as dehydrating agent, in dichloromethane at 0 °C, the desired **3a** was achieved as a pure compound, in almost quantitative yield.

**Scheme 1:**
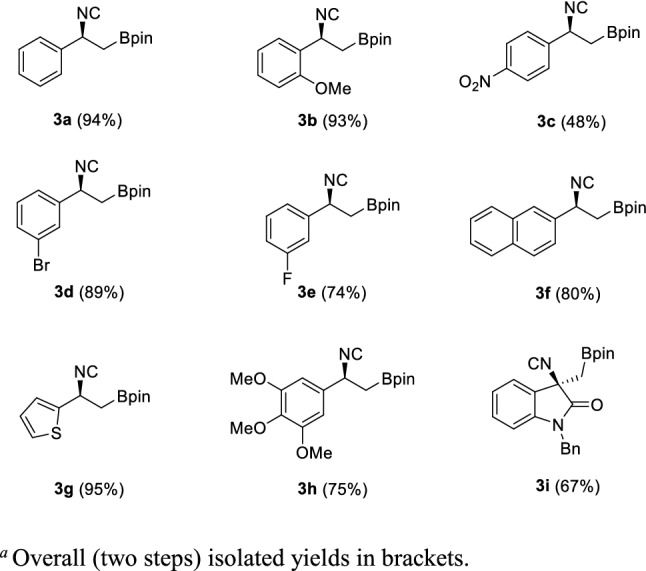
Substrate scope of β-substituted β-isocyano boronic esters **3**

Through this procedure, nine enantiopure β-isocyano boronic esters **3** were readily prepared, starting from the corresponding amine hydrochlorides **1** (Scheme 1).

The formylation-dehydration protocol tolerates well the presence of electron donating (**3b**), electron withdrawing (**3c**) and halogen (**3d**, **3e**) substituents on the phenyl ring. Also, β-amino boronates substituted with the naphtyl, the thienyl and the electron-rich and naturally occurring 3,4,5-trimethoxyphenyl ring react smoothly, affording the corresponding isocyanides **3f**, **3 g** and **3 h** in good yields. Finally, isocyanide **3i**, deriving from a quaternary oxindole-based amine, could also be achieved in good yield. All the obtained compounds proved to be stable under nitrogen at 4 °C, without any traces of degradation observed after 4 months.

**Scheme 2:**
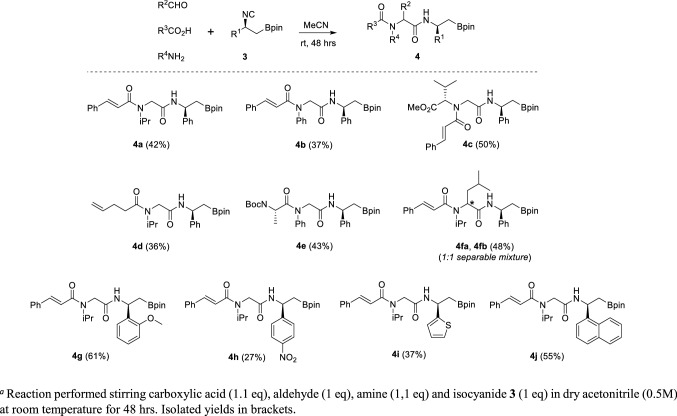
Substrate scope of (*S*)-β-substituted β-amido boronates **4**, obtained by Ugi 4-CR

Aiming to establish the synthetic versatility of obtained derivatives, we set up a series of different IMCRs, starting our investigation using compound **3a** in a classical Ugi 4-CR. After a brief solvent optimisation screening, performed on a model reaction involving cinnamic acid, paraformaldehyde, isopropyl amine, besides isocyanide **3a**, the substrate scope was fully demonstrated, as reported in Scheme 2.

Together with β-isocyano boronate **3a** and paraformaldehyde, various amine and carboxylic acid components were successfully evaluated, either aliphatic or aromatic or derived from properly protected amino acids (**4a-e**). Switching from paraformaldehyde to isovaleraldehyde as the carbonyl component led to a 1:1 mixture of diastereoisomers **4fa** and **4fb**, which could be partially separated by careful flash chromatography. The scope of isocyanides **3** was also evaluated. β-2-methoxyphenyl-β-isocyano boronic ester **3b** reacts with cinnamic acid, paraformaldehyde and isopropyl amine to give the expected Ugi product **4g** with a satisfying 61% yield, while using β-4-nitrophenyl-β-isocyano boronic ester **3c** the corresponding derivative **4h** was achieved in only 27% yield. These experimental outputs agree with the known reaction’s mechanism, according to which more electron-rich isocyanides are expected to perform as better nucleophiles. Finally, both naphtyl and thienyl derivatives **3g** and **3h** react smoothly, giving the corresponding β-amido boronates **4i** and **4j** in acceptable yields.

**Scheme 3:**
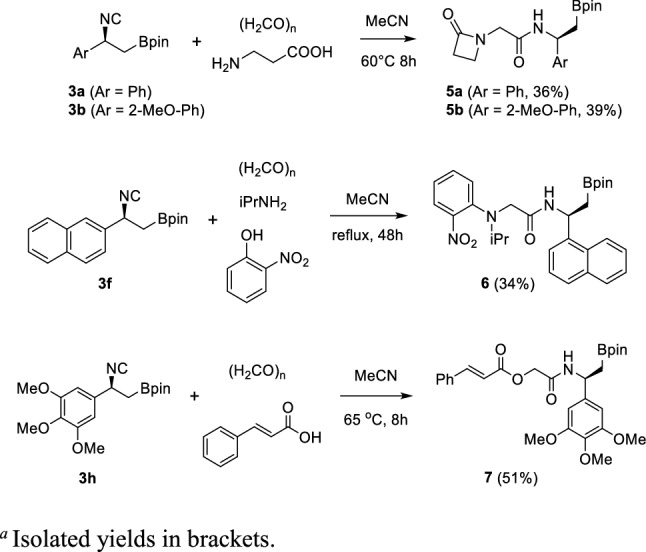
Synthesis of AA-Ugi 3-CR β-lactam derivatives **5**, Ugi-Smiles 3-CR derivative **6** and Passerini 3-CR derivative **7**

Aiming to combine the β-amido boronic acid moiety with the biologically relevant β-lactam ring, a bifunctional amino acid component, namely β-alanine, was employed in a three-component process (AA-Ugi 3-CR). Starting from isocyanides **3a** and **3b**, the desired products **5a** and **5b** were obtained in acceptable yields, conducting the reaction in acetonitrile at 60 °C. Further evaluation of the isocyanide scope was pursued by reacting the β-isocyano boronic esters **3f** and **3h** in a Ugi-Smiles 3-CR and in a Passerini 3-CR, respectively. Corresponding products **6** and **7** were obtained in good yield (Scheme 3).

**Scheme 4:**
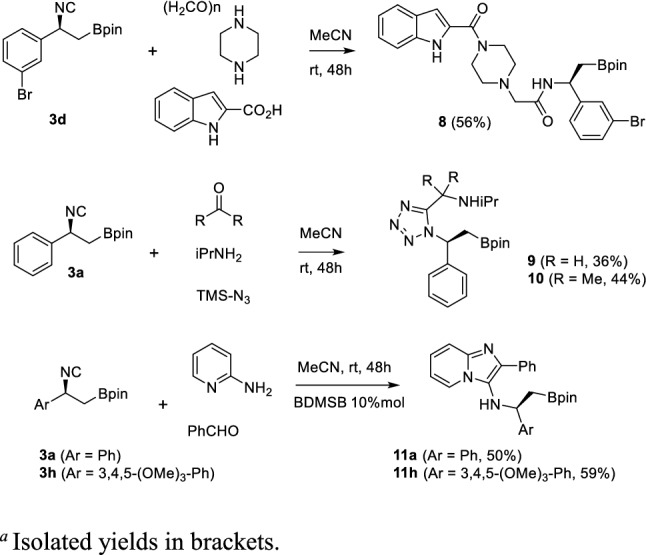
Synthesis of split-Ugi 4-CR derivative **8**, Ugi-azide 4-CR derivatives **9** and **10** and GBB 3-CR derivatives **11a** and **11 h**

The exploitation of further IMCRs was then continued within a more general molecular hybridization strategy [38, 39], by which the β-amino boronic acid moiety could be combined with other prominent pharmacophoric moieties, to produce novel hybrid compounds. Therefore, isocyanide **3d** was reacted with piperazine as a model secondary amine in a split-Ugi 4-CR, affording in one-pot the peptidomimetic **8** in good yield, avoiding the use of any protecting group. Boron-containing tetrazole derivatives **9** and **10** were achieved by the Ugi-azide 4-CR from compound **3a**, using both paraformaldehyde and acetone as carbonyl components, while imidazopyridines **11a** and **11h** arose from isocyanides **3a** and **3h,** respectively, via a Groebcke-Blackburn-Bienaymè reaction (GBB 3-CR) (Scheme 4). In this last reaction, best conversions were achieved adding 10 mol% of bromodimethylsulfonium bromide (BDMS) as catalyst, at room temperature [40].

**Scheme 5:**
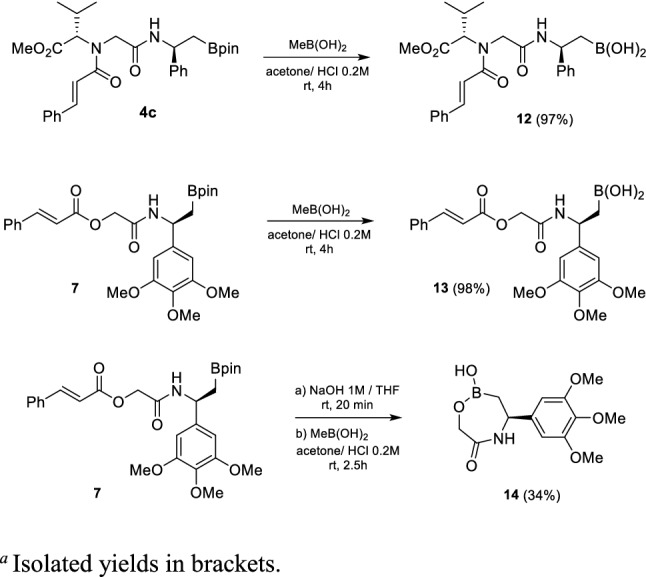
Pinacol removal from boronic esters **6** and **7** and synthesis of cyclic boronate **14**

Aware of the need for free boronic acids or, even better, cyclic boronates for biological activity [41–43], the mild pinacol ester removal was demonstrated starting from compounds **6** and **7**, to afford the corresponding β-amido boronic acids **12** and **13** quantitatively. Furthermore, amido ester **7** was also hydrolysed at the cinnamate ester and then sequentially deprotected from pinacol, allowing a ring closure with the formation of the unprecedented 1,4,5-oxazaborepan-2-one ring. Compound **14** was achieved in moderate yield, due to the concurring deboronation side-reaction during the hydrolysis of the cinnamate ester (Scheme 5).

**Fig. 2:**
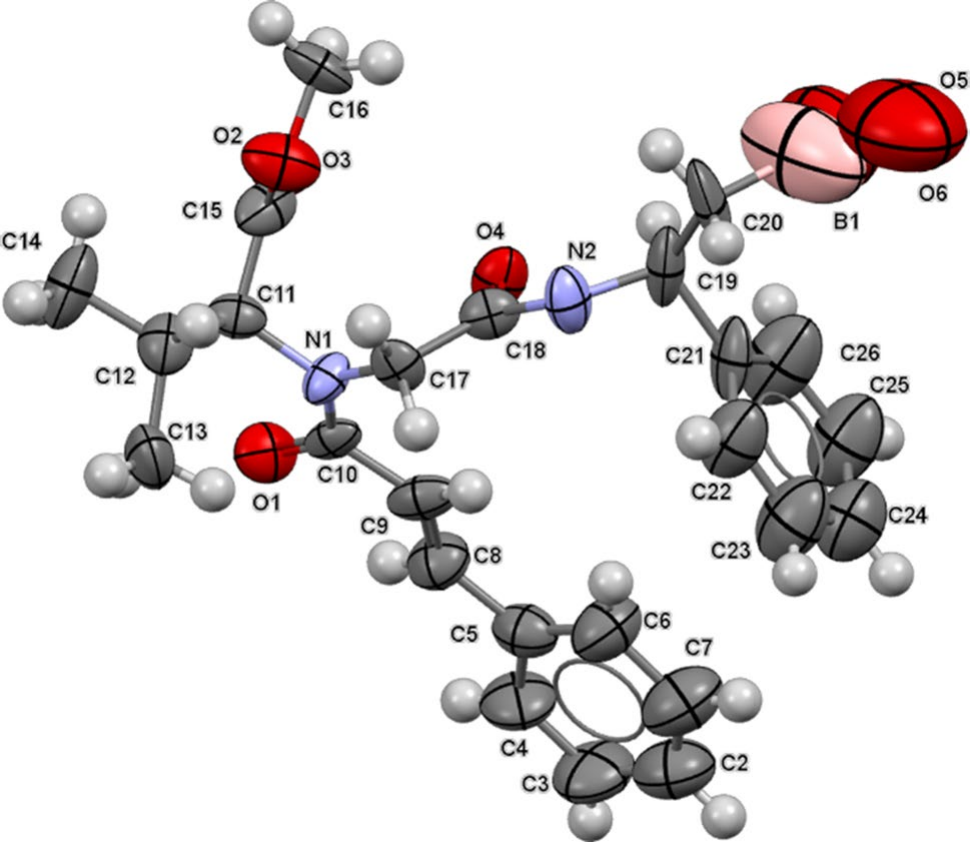
Asymmetric unit of compound **12**, with the atom-numbering scheme. Thermal ellipsoids at RT were drawn at the 25% probability level. Atoms are represented with the usual color code (C: grey; N: blue; O: red; H: white; B: pink)

All boron-containing described compounds are fully characterized by ^1^H, ^13^C, and ^11^B NMR. In presence of amide bonds, *trans–cis* rotamery affects ^1^H and ^13^C spectra, but could be confidently quantified by integrating the ^1^H NMR peaks assigned to each rotamer [44] (see Experimental Section). By way of example, the molecular structure of **12** was further confirmed in the solid state, by means of single crystal X-ray diffraction [45] (Fig. 2, full details are deposited in the Supporting Information).

## Conclusion

The straightforward preparation of chiral, non-racemic aliphatic isocyano boronic esters is disclosed here, together with the exhaustive exploitation of such components in a variety of IMCRs. Boron-containing isocyanides were obtained from the corresponding β-amino boronic acid pinacol esters, without need for protecting group interconversion, with overall yield up to 95%, over two purification-free steps. They fully proved to be reliable building blocks for any type of IMCRs, ranging from classical Ugi and Passerini reactions to their many variants, opening the way to new boron-containing peptidomimetics and heteroatom-rich small molecules, including cyclic boronates. We believe that the described work can be useful to support the design and synthesis of boron-containing covalent inhibitor libraries. At the same time, it can inspire new synthetic pathways that exploit the carbon-boron bond to increase structural complexity, for instance through Pd-catalyzed cross coupling reactions.

## Experimental section

### General information

All reactions were carried out under nitrogen atmosphere. All hydrochlorides **1** were prepared according to the methods reported in the literature [25, 37]. All employed reagents, including aldehydes, carboxylic acids and amines, are commercially available. Solvents were purchased as “anhydrous” and used without further purification. ^1^H NMR, ^13^C NMR and ^11^B NMR spectra were recorded using a Bruker AV 400 Ultrashield spectrometer. ^1^H NMR and ^13^C NMR chemical shifts were reported in parts per million (ppm) downfield from tetramethylsilane,^11^B NMR chemical shifts were determined relative to BF_3_·Et_2_O and spectra were recorded using quartz NMR tubes. Coupling constants (*J*) were reported in Hertz (Hz). The residual solvent peaks were used as internal references: ^1^H NMR (CDCl_3_ 7.26 ppm, DMSO d-6 2.51 ppm), ^13^C NMR (CDCl_3_ 77.0 ppm, DMSO d-6 40.0 ppm). The following abbreviations were used to explain the multiplicities: s = singlet, d = doublet, t = triplet, m = multiplet, br = broad, app = apparent. Reactions involving boron-containing compounds were followed by TLC using a curcumin solution, which was prepared as reported in the literature [46]. Chromatographic purifications were performed by Flash Chromatography (FC), using Merck Silica gel 60.

### General procedure A for the synthesis of formylated compounds 2

To a suspension of β-amino boronic hydrochloride (0.956 mmol, 1 eq) in ethyl formate: dimethylformamide 3:2 (9.5 mL, 0.1 M), freshly distilled triethylamine (0.956 mmol, 1 eq) was added dropwise. The reaction was stirred at room temperature for 10 min, then heated at 90 °C for 3 h. The reaction was cooled to room temperature and solvents were removed under reduced pressure. The crude product was dissolved in diethyl ether and washed with an equal amount of aqueous NH_4_Cl sat. (1x), water (1x) and brine (1x). The organic phase was dried over anhydrous sodium sulphate and the solvent removed under reduced pressure to afford compound **2**.

### General procedure B for the synthesis of β-substituted β-isocyano boronic esters 3

Synthesized following a reported literature procedure for dehydration of formylated compounds [36]: Under nitrogen, to a 0 °C solution of **2** (0.325 mmol, 1 eq) in dichloromethane (3.5 mL, 0.1 M), freshly distilled triethylamine (1.625 mmol, 5 eq) and phosphorus oxychloride (0.488 mmol, 1.5 eq) were added dropwise and the reaction stirred for 1.5 h at 0 °C. Aqueous NaHCO_3_ sat. (20 mL) was added and the reaction stirred at room temperature for 10 min. The product was extracted with dichloromethane (3 × 40 mL), then freshly distilled triethylamine (1 mL) was added to the combined organic phases which were washed again with aqueous NaHCO_3_ sat. (20 mL) and water (20 mL). The mixture was dried over sodium sulphate and the solvent removed under reduced pressure to afford compound **3**.

### General procedure C for the synthesis of β-amido boronates 4

In a round-bottom flask, under nitrogen, the desired aldehyde (1 eq), amine (1.1 eq), carboxylic acid (1.1 eq) and **(3)** (1 eq) were dissolved in dry acetonitrile (0.5 M) and stirred at room temperature for 48 h. The solvent was removed under reduced pressure and the crude product purified by FC to afford compound **4**.

### General procedure D for the synthesis of free boronic acids

Synthesized following a modified version of the previously reported literature [47]: In a round-bottom flask, the desired β-amido boronic ester (0.20 mmol, 1 eq) and methylboronic acid (120 mg, 2.00 mmol, 10 eq) were dissolved in an acetone/0.2 N HCl_aq_ (1:1 v/v) solution (5 mL, 0.04 M) and stirred at room temperature for 4 h (the reaction changes from yellow to pale light-yellow). The solvent was evaporated under reduced pressure using a 50 °C hot bath, then the crude was dissolved in MeCN/H_2_O 1:1 (4 mL) and freeze-dried to afford the desired β-amido boronic acids.

### Supplementary Information

Below is the link to the electronic supplementary material.

Supplementary file1 (PDF 4457 kb)
