# Supplementary material for: Enantiopure β-isocyano-boronic esters: synthesis and exploitation in isocyanide-based multicomponent reactions
Source: Mol Divers. 2022 Oct 19;27(5):2161–8. doi: 10.1007/s11030-022-10549-8 (PMC10520151; doi:10.1007/s11030-022-10549-8)
Supplement: Supplementary file 1 — Supplementary file1 (PDF 4457 kb) [file 11030_2022_10549_MOESM1_ESM.pdf]

Electronic Supporting Information

for

Enantiopure β-isocyano-boronic esters: synthesis and applications in isocyanide-based multicomponent reactions

Marco Manenti, Simone Gusmini, Leonardo Lo Presti, Giorgio Molteni and Alessandra Silvani*

Dipartimento di Chimica, Università degli Studi di Milano, Via Golgi 19, Milano, 20133, Italy
E-mail: alessandra.silvani@unimi.it.

Table of contents

Characterization data for all new compounds ………………………………………………...S2-S13

Copies of ^{1}H, ^{13}C and ^{11}B NMR spectra for all new compounds….…………………………S14-S95

Crystallographic data for compound **12**………..……………………………………………S96-S97

***(S)-N-(1-phenyl-2-(4,4,5,5-tetramethyl-1,3,2-dioxaborolan-2-yl)ethyl)formamide* (2a)**

Synthetized following the general procedure A starting from *(S)-1-phenyl-2-(4,4,5,5-tetramethyl-1,3,2-dioxaborolan-2-yl)ethan-1-aminium chloride*; yield: 97%; $[\alpha]^{20}_{D}$= – 55.4 (c 1.0, $CHCl_3$); ^1H NMR (400 MHz, $CDCl_3$, rotamers mixture 75:25) δ 8.16 (s, 1H), 7.31-7.10 (m, 5H), 6.77 (d, J = 8.4 Hz, 0.75H), 6.62-6.57 (m, 0.25H), 5.37(dd, J_2 = 14.3 Hz, J_3 = 7.4 Hz, 0.75H), 4.81 (dd, J_2 = 14.3 Hz, J_3 = 7.4 Hz, 0.25H), 1.47 (dd, J_2 = 15.6 Hz, J_3 = 7.6 Hz, 1H), 1.38 (dd, J_2 = 15.6 Hz, J_3 = 7.6 Hz, 1H), 1.12 (m, 12H); ^{13}C NMR (101 MHz, $CDCl_3$, rotamers mixture 75:25) δ 164.1 and 160.2 (1C), 143.1 and 143.0 (1C), 128.6 and 128.4 (2C), 127.5 and 127.1 (1C), 126.2 and 125.9 (2C), 83.8 and 83.6 (2C), 52.6 and 48.1 (1C), 24.8 (2C), 24.6 (2C), (1C missing due to boron-quadrupole-induced relaxation); ^{11}B NMR (128 MHz, $CDCl_3$) δ 32.8; HRMS (ESI) calcd for $C_{15}H_{22}BNNaO_3^+$ $[M+Na]^+$ 298.1585, found 298.1580

***(S)-N-(1-(2-methoxyphenyl)-2-(4,4,5,5-tetramethyl-1,3,2-dioxaborolan-2-yl)ethyl)formamide* (2b)**

Synthetized following the general procedure A starting from *(S)-1-(2-methoxyphenyl)-2-(4,4,5,5-tetramethyl-1,3,2-dioxaborolan-2-yl)ethan-1-aminium chloride*; yield: 96%; $[\alpha]^{20}_{D}$= – 44.0 (c 1.0, $CHCl_3$); ^1H NMR (400 MHz, $CDCl_3$, rotamers mixture 60:40) δ 8.17 (br. s, 0.4H), 8.11 (s, 0.6H), 7.24-7.15 (m, 2H), 6.89-6.54 (m, 2.6H), 6.57 (app. t, 0.4H), 5.52-5.46 (m, 0.6H), 4.96-4.90 (m, 0.4H), 3.82 (s, 3H), 1.58-1.38 (m, 2H), 1.15 (s, 6H), 1.11 (s, 6H); ^{13}C NMR (101 MHz, $CDCl_3$, rotamers mixture 60:40) δ 164.1 and 159.9 (1C), 156.8 and 156.4 (1C), 130.9 and 130.7 (1C), 128.7 and 128.3 (1C), 128.0 and 127.0 (1C), 120.5 and 120.3 (1C), 110.8 (1C), 83.6 and 83.3 (2C), 55.2 (1C), 50.2 and 46.2 (1C), 24.9 and 24.8 (2C), 24.7 and 24.6 (2C), (1C missing due to boron-quadrupole-induced relaxation); ^{11}B NMR (128 MHz, $CDCl_3$) δ 33.3; HRMS (ESI) calcd for $C_{16}H_{24}BNNaO_4^+$ $[M+Na]^+$ 328.1702, found 328.1713

***(S)-N-(1-(4-nitrophenyl)-2-(4,4,5,5-tetramethyl-1,3,2-dioxaborolan-2-yl)ethyl)formamide* (2c)**

Synthetized following the general procedure A starting from *(S)-1-(4-nitrophenyl)-2-(4,4,5,5-tetramethyl-1,3,2-dioxaborolan-2-yl)ethan-1-aminium chloride*; yield: 51%; $[\alpha]^{20}_{D}$= – 60.6 (c 1.0, $CHCl_3$); ^1H NMR (400 MHz, $CDCl_3$) δ 8.29 (s, 1H), 8.23-8.17 (m, 2H), 7.51 (d, J = 8.6 Hz, 2H), 6.78 (br s, 1H), 5.45 (dd, J_2 = 13.1 Hz, J_3 = 6.0 Hz, 1H), 1.49-1.48 (m, 1H), 1.39-1.35 (m, 1H), 1.18-1.17 (m, 12H); ^{13}C NMR (101 MHz, $CDCl_3$) δ 160.8 (1C), 150.9 (1C), 137.6 (1C), 133.0 (1C), 131.6 (1C), 128.8 (1C), 127.2 (1C), 84.0 (2C), 48.2 (1C), 25.0 (2C), 24.8 (2C), (1C missing due to boron-quadrupole-induced relaxation); ^{11}B NMR (128 MHz, $CDCl_3$) δ 33.2; HRMS (ESI) calcd for $C_{15}H_{21}BN_2NaO_5^+$ $[M+Na]^+$ 343.1436, found 343.1431

***(S)-N-(1-(3-bromophenyl)-2-(4,4,5,5-tetramethyl-1,3,2-dioxaborolan-2-yl)ethyl)formamide* (2d)**

Synthetized following the general procedure A starting from *(S)-1-(3-bromophenyl)-2-(4,4,5,5-tetramethyl-1,3,2-dioxaborolan-2-yl)ethan-1-aminium chloride*; yield: 95%; $[\alpha]^{20}_{D}$= – 56.1 (c 0.3, $CHCl_3$); ^1H NMR (400 MHz, $CDCl_3$, rotamers mixture 80:20) δ 8.22 (s, 1H), 7.46 (s, 1H), 7.39-7.33 (m, 1H), 7.25-7.14 (m, 2H), 6.81 (br. s, 0.8H), 6.65 (br. s, 0.2H), 5.35-5.29 (dd, J_2 = 13.1 Hz, J_3 = 6.7 Hz, 0.8H), 4.82-4.77 (dd, J_2 = 13.1 Hz, J_3 = 6.7 Hz, 0.2H), 1.47-1.37 (m, 2H), 1.16 (s, 12H); ^{13}C NMR (101 MHz, $CDCl_3$, rotamers mixture 80:20) δ 164.8 and 161.0 (1C), 146.2 (1C), 131.2 and 130.8 (1C), 130.9 and 130.7 (1C), 130.0 and 129.5 (1C), 125.7 and 125.3 (1C), 123.4 and 123.1 (1C), 84.6 and 84.4 (2C), 52.9 and 48.5 (1C), 25.5 (2C), 25.3 (2C), (1C missing due to boron-

quadrupole-induced relaxation); ^{11}B NMR (128 MHz, $CDCl_3$) δ 33.1; HRMS (ESI) calcd for $C_{15}H_{21}BBrNNaO_3^+$ $[M+Na]^+$ 376.0690, found 376.0699

***(S)-N-(1-(3-fluorophenyl)-2-(4,4,5,5-tetramethyl-1,3,2-dioxaborolan-2-yl)ethyl)formamide* (2e)**

Synthetized following the general procedure A starting from *(S)-1-(3-fluorophenyl)-2-(4,4,5,5-tetramethyl-1,3,2-dioxaborolan-2-yl)ethan-1-aminium chloride*; yield: 87%; $[\alpha]^{20}_D$= – 51.7 (c 1.0, $CHCl_3$); ^{1}H NMR (400 MHz, $CDCl_3$, rotamers mixture 80:20) δ 8.20 (s, 0.80H), 8.15 (d, *J* = 12.3 Hz, 0.20H), 7.32-7.23 (m, 1H), 7.09 (d, *J* = 8.0 Hz, 1H), 7.06-7.00 (m, 1H), 6.97-6.88 (m, 1H), 6.72 (d, *J* = 7.8 Hz, 0.80H), 6.56 (m, 0.20H), 5.39-5.34 (m, 0.80H), 4.85-4.81 (m, 0.20H), 1.45 (dd, J_2 = 15.3 Hz, J_3 = 7.0 Hz, 1H), 1.38 (dd, J_2 = 15.3 Hz, J_3 = 7.0 Hz, 1H), 1.16-1.14 (m, 12H); ^{13}C NMR (101 MHz, $CDCl_3$, rotamers mixture 80:20) δ 164.8-164.6 (1C), 162.2 (1C), 146.8 and 146.7 (1C), 137.3 and 136.6 (1C), 122.6 and 122.2 (1C), 115.0-114.3 (1C), 114.0-113.6 (1C), 84.5 and 84.2 (2C), 52.9 and 48.6 (1C), 25.4 (2C), 25.2 (2C), (1C missing due to boron-quadrupole-induced relaxation); ^{11}B NMR (128 MHz, $CDCl_3$) δ 33.0; HRMS (ESI) calcd for $C_{15}H_{21}BFNNaO_3^+$ $[M+Na]^+$ 316.1491, found 316.1499

***(S)-N-(1-(naphthalen-2-yl)-2-(4,4,5,5-tetramethyl-1,3,2-dioxaborolan-2-yl)ethyl)formamide* (2f)**

Synthetized following the general procedure A starting from *(S)-N-(1-(naphthalen-2-yl)-2-(4,4,5,5-tetramethyl-1,3,2-dioxaborolan-2-yl)ethyl)formamide*; yield: 82%; $[\alpha]^{20}_D$= – 97.2 (c 1.0, $CHCl_3$); ^{1}H NMR (400 MHz, $CDCl_3$, rotamers mixture 75:25) δ 8.30 (s, 0.75H), 8.25 (d, *J* = 13.7 Hz, 0.25H), 7.85-7.76 (m, 4H), 7.52-7.41 (m, 3H), 6.68 (d, *J* = 8.2 Hz, 0.75H), 6.55 (app. t, 0.25H), 5.61 (app. q, 0.75H), 5.05-5.00 (m, 0.25H), 1.62 (dd, J_2 = 15.7 Hz, J_3 = 7.0 Hz, 1H), 1.52 (dd, J_2 = 15.7 Hz, J_3 = 7.0 Hz, 1H), 1.16-1.15 (m, 12H); ^{13}C NMR (101 MHz, $CDCl_3$, rotamers mixture 75:25) δ 164.8 and 160.9 (1C), 141.2 (1C), 133.9 (1C), 133.3 (1C), 129.3 and 129.0 (1C), 128.5 (1C), 128.3 (1C), 127.1 and 126.8 (1C), 126.4 (1C), 125.5 (1C), 125.1 and 124.9 (1C), 84.5 and 84.3 (2C), 53.4 and 48.8 (1C), 25.5 (2C), 25.3 (2C), (1C missing due to boron-quadrupole-induced relaxation); ^{11}B NMR (128 MHz, $CDCl_3$) δ 32.2; HRMS (ESI) calcd for $C_{19}H_{24}BNNaO_3^+$ $[M+Na]^+$ 348.1741, found 348.1749

***(S)-N-(2-(4,4,5,5-tetramethyl-1,3,2-dioxaborolan-2-yl)-1-(thiophen-2-yl)ethyl)formamide* (2g)**

Synthetized following the general procedure A starting from *(S)-2-(4,4,5,5-tetramethyl-1,3,2-dioxaborolan-2-yl)-1-(thiophen-2-yl)ethan-1-aminium chloride*; yield: 97%; $[\alpha]^{20}_D$= – 56.1 (c 0.3, $CHCl_3$); ^{1}H NMR (400 MHz, $CDCl_3$, rotamers mixture 75:25) δ 8.21 (s, 1H), 7.22 (dd, J_3 = 5.1 Hz, J_4 = 1.2 Hz, 0.25H), 7.17 (dd, J_3 = 5.1 Hz, J_4 = 1.2 Hz, 0.75H), 6.96-6.90 (m, 2H), 6.70-6.69 (m, 0.75H), 6.48 (s, 0.25H), 5.70-5.65 (m, 0.75H), 5.08-5.02 (m, 0.25H), 1.59 (dd, J_2 = 15.6 Hz, J_3 = 6.6 Hz, 1H), 1.47 (dd, J_2 = 15.6 Hz, J_3 = 6.6 Hz, 1H), 1.18 (s, 6H), 1.16 (s, 6H); ^{13}C NMR (101 MHz, $CDCl_3$, rotamers mixture 75:25) δ 164.7 and 160.1 (1C), 147.3 (1C), 127.0 and 126.7 (1C), 124.8 and 124.1 (1C), 124.0 and 123.9 (1C), 83.9 and 83.7 (2C), 49.0 and 44.0 (1C), 24.8 (2C), 24.5 (2C), (1C missing due to boron-quadrupole-induced relaxation); ^{11}B NMR (128 MHz, $CDCl_3$) δ 33.3; HRMS (ESI) calcd for $C_{13}H_{20}BN_2NaO_3S^+$ $[M+Na]^+$ 304.1149, found 304.1137

***(S)-N-(2-(4,4,5,5-tetramethyl-1,3,2-dioxaborolan-2-yl)-1-(3,4,5-trimethoxyphenyl)ethyl)formamide* (2h)**

Synthetized following the general procedure A starting from *(S)-2-(4,4,5,5-tetramethyl-1,3,2-dioxaborolan-2-yl)-1-(3,4,5-trimethoxyphenyl)ethan-1-aminium chloride*; yield: 85%; $[\alpha]^{20}_{D}$= - 51.4 (c 1.0, $CHCl_3$); ^{1}H NMR (400 MHz, $CDCl_3$, rotamers mixture 70:30) δ 8.18 (s, 0.7H), 8.14 (d, *J* = 11.9 Hz, 0.30H), 6.61 (d, *J* = 8.4 Hz, 0.70H), 6.54 (s, 2H), 6.49 (s, 0.30H), 5.31 (app. t, 0.70H), 4.77-4.72 (m, 0.30H), 3.82 (s, 6H), 3.77 (s, 3H), 1.46 (dd, J_2 = 15.7 Hz, J_3 = 7.3 Hz, 1H), 1.37 (dd, J_2 = 15.7 Hz, J_3 = 7.3 Hz, 1H), 1.15-1.13 (m, 12H); ^{13}C NMR (101 MHz, $CDCl_3$, rotamers mixture 70:30) δ 164.8 and 160.9 (1C), 153.9 and 153.7 (2C), 139.7 and 139.6 (1C), 137.8 and 137.7 (1C), 104.0 and 103.7 (2C), 84.4 and 84.2 (2C), 61.4 (1C), 56.7 (2C), 49.0 (1C), 25.4 (2C), 25.3 (2C), (1C missing due to boron-quadrupole-induced relaxation); ^{11}B NMR (128 MHz, $CDCl_3$) δ 33.3; HRMS (ESI) calcd for $C_{18}H_{28}BNNaO_6^+$ $[M+Na]^+$ 388.1902, found 388.1893

***(R)-N-(1-benzyl-2-oxo-3-((4,4,5,5-tetramethyl-1,3,2-dioxaborolan-2-yl)methyl)indolin-3-yl)formamide* (2i)**

Synthetized following the general procedure A starting from *(R)-1-benzyl-2-oxo-3-((4,4,5,5-tetramethyl-1,3,2-dioxaborolan-2-yl)methyl)indolin-3-aminium chloride*; yield: 72%; $[\alpha]^{20}_{D}$= + 6.5 (c 1.0, $CHCl_3$); ^{1}H NMR (400 MHz, $CDCl_3$) δ 8.11 (s, 1H), 7.44 (d, *J*= 7.5 Hz, 2H), 7.33 (t, *J*= 7.5 Hz, 2H), 7.28-7.25 (m, 2H), 7.14 (app t, J_2= 7.9 Hz, J_3= 1.3 Hz, 1H), 7.07 (br s, 1H), 6.99 (app t, J_2= 7.9 Hz, J_3= 1.3 Hz, 1H), 6.65 (d, *J*= 7.9 Hz, 1H), 5.08 (d, *J*= 16.0 Hz, 1H), 4.90 (d, *J*= 16.0 Hz, 1H), 1.63 (d, *J*= 15.8 Hz, 1H), 1.37 (d, *J*= 15.8 Hz, 1H), 1.30 (s, 6H), 1.26 (s, 6H); ^{13}C NMR (101 MHz, $CDCl_3$) δ 176.6 (1C), 159.7 (1C), 142.3 (1C), 135.8 (1C), 131.2 (1C), 128.7 (3C), 127.4 (1C), 127.2 (2C), 122.6 (1C), 122.0 (1C), 109.5 (1C), 84.3 (2C), 58.7 (1C), 44.2 (1C), 24.9 (2C), 24.8 (2C), (1C missing due to boron-quadrupole-induced relaxation); ^{11}B NMR (128 MHz, $CDCl_3$) δ 32.4; HRMS (ESI) calcd for $C_{23}H_{27}BN_2NaO_4^+$ $[M+Na]^+$ 429.1956, found 429.1960

***(S)-2-(2-isocyano-2-phenylethyl)-4,4,5,5-tetramethyl-1,3,2-dioxaborolane* (3a)**

Synthetized following the general procedure B starting from **2a**; yield: 97%; $[\alpha]^{20}_{D}$= – 28.5 (c 1.0, $CHCl_3$); ^{1}H NMR (400 MHz, $CDCl_3$) δ 7.42-7.26 (m, 5H), 4.94 (app. t, 1H), 1.69 (dd, J_2 = 16.0 Hz, J_3 = 8.0 Hz, 1H), 1.58-1.49 (m, 1H), 1.22 (s, 12H); ^{13}C NMR (101 MHz, $CDCl_3$) δ 155.9 (1C), 139.3 (1C), 128.7 (2C), 128.2 (1C), 125.9 (2C), 83.9 (2C), 55.2 (1C), 24.8 (2C), 24.7 (2C), (1C missing due to boron-quadrupole-induced relaxation); ^{11}B NMR (128 MHz, $CDCl_3$) δ 32.4; HRMS (ESI) calcd for $C_{15}H_{20}BNNaO_2^+$ $[M+Na]^+$ 280.1479, found 280.1470

***(S)-2-(2-isocyano-2-(2-methoxyphenyl)ethyl)-4,4,5,5-tetramethyl-1,3,2-dioxaborolane* (3b)**

Synthetized following the general procedure B starting from **2b**; yield: 97%; $[\alpha]^{20}_{D}$= - 40.2 (c 1.0, $CHCl_3$); ^{1}H NMR (400 MHz, $CDCl_3$) δ 7.48 (d, *J* = 7.8 Hz, 1H), 7.30 (t, *J* = 7.4 Hz, 1H), 7.00 (t, *J* = 7.4 Hz, 1H), 6.88 (d, *J* = 7.8 Hz, 1H), 5.31-5.28 (m, 1H), 3.86 (s, 3H), 1.55 (app. d, 2H), 1.24 (br s, 12H); ^{13}C NMR (101 MHz, $CDCl_3$) δ 155.6 (1C), 155.0 (1C), 129.3 (1C), 127.5 (1C), 126.5 (1C), 120.8 (1C), 110.6 (1C), 83.8 (2C), 55.4 (1C), 49.9 (1C), 24.9 (2C), 24.6 (2C), (1C missing due to boron-quadrupole-induced relaxation); ^{11}B NMR (128 MHz, $CDCl_3$) δ 33.7; HRMS (ESI) calcd for $C_{16}H_{22}BNNaO_3^+$ $[M+Na]^+$ 310.1585, found 310.1580

***(S)-2-(2-isocyano-2-(4-nitrophenyl)ethyl)-4,4,5,5-tetramethyl-1,3,2-dioxaborolane* (3c)**

Synthetized following the general procedure B starting from **2c**; yield: 95%; $[\alpha]^{20}_D$= - 19.2 (c 0.3, $CHCl_3$); ^1H NMR (400 MHz, $CDCl_3$) δ 8.27 (d, *J* = 8.7 Hz, 2H), 7.62 (d, *J* = 8.7 Hz, 2H), 5.10-5.06 (m, 1H), 1.60-1.53 (m, 2H), 1.24 (s, 12H); ^{13}C NMR (101 MHz, $CDCl_3$) δ 146.9 (1C), 132.1 (1C), 131.5 (1C), 127.0 (2C), 124.1 (2C), 84.3 (2C), 54.8 (1C), 24.8 (2C), 24.7 (2C), (1C missing due to boron-quadrupole-induced relaxation); ^{11}B NMR (128 MHz, $CDCl_3$) δ 32.9; HRMS (ESI) calcd for $C_{15}H_{19}BN_2NaO_4^+$ $[M+Na]^+$ 325.1330, found 325.1320

***(S)-2-(2-(3-bromophenyl)-2-isocyanoethyl)-4,4,5,5-tetramethyl-1,3,2-dioxaborolane* (3d)**

Synthetized following the general procedure B starting from **2d**; yield: 98%; $[\alpha]^{20}_D$= - 34.9 (c 0.6, $CHCl_3$); ^1H NMR (400 MHz, $CDCl_3$) δ 7.57 (s, 1H), 7.48 (d, *J* = 8.0 Hz, 1H), 7.37 (d, *J* = 8.0 Hz, 1H), 7.27 (t, *J* = 8.0 Hz, 1H), 4.91 (app. t, 1H), 1.69 (dd, J_2 = 15.6 Hz, J_3 = 6.7 Hz, 1H), 1.53 (dd, J_2 = 15.6 Hz, J_3 = 6.7 Hz, 1H), 1.24 (s, 12H); ^{13}C NMR (101 MHz, $CDCl_3$) δ 157.3 (1C), 142.0 (1C), 132.0 (1C), 131.1 (1C), 129.9 (1C), 125.1 (1C), 123.3 (1C), 84.7 (2C), 55.2 (1C), 25.4 (4C), (1C missing due to boron-quadrupole-induced relaxation); ^{11}B NMR (128 MHz, $CDCl_3$) δ 32.3; HRMS (ESI) calcd for $C_{15}H_{19}BBrNNaO_2^+$ $[M+Na]^+$ 358.0584, found 358.0583

***(S)-2-(2-(3-fluorophenyl)-2-isocyanoethyl)-4,4,5,5-tetramethyl-1,3,2-dioxaborolane* (3e)**

Synthetized following the general procedure B starting from **2e**; yield: 85%; $[\alpha]^{20}_D$= - 21.1 (c 1.0, $CHCl_3$); ^1H NMR (400 MHz, $CDCl_3$) δ 7.38-7.32 (m, 1H), 7.19 (d, *J* = 7.9 Hz, 1H), 7.15-7.12 (m, 1H), 7.05-7.00 (m, 1H), 4.93 (app t, 1H), 1.68 (dd, J_2 = 16.0 Hz, J_3 = 7.8 Hz, 1H), 1.52 (dd, J_2 = 16.0 Hz, J_3 = 7.8 Hz, 1H), 1.23 (s, 12H); ^{13}C NMR (101 MHz, $CDCl_3$) δ 162.8 (d, *J* = 247.6 Hz, 1H), 156.5 (1C), 141.7-141.6 (1C), 130.5-130.4 (1C), 121.5 (1C), 115.3-115.1 (1C), 113.3-113.1 (1C), 84.1 (2C), 54.8 (1C), 24.8 (2C), 24.7 (2C), (1C missing due to boron-quadrupole-induced relaxation); ^{11}B NMR (128 MHz, $CDCl_3$) δ 32.3; HRMS (ESI) calcd for $C_{15}H_{19}BFNNaO_2^+$ $[M+Na]^+$ 298.1385, found 298.1398

***(S)-2-(2-isocyano-2-(naphthalen-2-yl)ethyl)-4,4,5,5-tetramethyl-1,3,2-dioxaborolane* (3f)**

Synthetized following the general procedure B starting from **2f**; yield: 98%; $[\alpha]^{20}_D$= - 18.1 (c 1.0, $CHCl_3$); ^1H NMR (400 MHz, $CDCl_3$) δ 7.87-7.83 (m, 4H), 7.53-7.48 (m, 3H), 5.11 (app. t, 1H), 1.77 (dd, J_2 = 15.6 Hz, J_3 = 7.8 Hz, 1H), 1.63 (dd, J_2 = 15.6 Hz, J_3 = 6.7 Hz, 1H), 1.22 (s, 6H), 1.21 (s, 6H); ^{13}C NMR (101 MHz, $CDCl_3$) δ 156.8 (1C), 137.2 (1C), 133.7 (1C), 133.6 (1C), 129.5 (1C), 128.6 (1C), 128.4 (1C), 127.2 (1C), 127.1 (1C), 125.5 (1C), 124.2 (1C), 84.6 (2C), 56.0 (1C), 25.4 (4C), (1C missing due to boron-quadrupole-induced relaxation); ^{11}B NMR (128 MHz, $CDCl_3$) δ 32.5; HRMS (ESI) calcd for $C_{19}H_{22}BNNaO_2^+$ $[M+Na]^+$ 330.1636, found 330.1627

***(S)-2-(2-isocyano-2-(thiophen-2-yl)ethyl)-4,4,5,5-tetramethyl-1,3,2-dioxaborolane* (3g)**

Synthetized following the general procedure B starting from **2g**; yield: 98%; $[\alpha]^{20}_D$= - 18.2 (c 1.0, $CHCl_3$); ^1H NMR (400 MHz, $CDCl_3$) δ 7.27 (dd, J_3 = 5.2 Hz, J_4 = 1.3 Hz, 1H), 7.08-7.07 (m, 1H), 6.97-6.95 (m, 1H), 5.18 (app. t, 1H), 1.78 (dd, J_2 = 15.7 Hz, J_3 = 7.6 Hz, 1H), 1.66 (dd, J_2 = 15.7 Hz, J_3 = 7.6 Hz, 1H), 1.24 (s, 6H), 1.22 (s, 6H); ^{13}C NMR (101 MHz, $CDCl_3$) δ 156.6 (1C), 142.9 (1C), 127.3 (1C), 126.0 (1C), 125.8 (1C), 84.7 (2C), 51.2 (1C), 25.4 (2C), 25.3 (2C), (1C missing due to boron-quadrupole-induced relaxation); ^{11}B NMR (128 MHz, $CDCl_3$) δ 32.3; HRMS (ESI) calcd for $C_{13}H_{18}BNNaO_2S^+$ $[M+Na]^+$ 286.1043, found 286.1049

(S)-2-(2-isocyano-2-(3,4,5-trimethoxyphenyl)ethyl)-4,4,5,5-tetramethyl-1,3,2-dioxaborolane **(3h)**

Synthetized following the general procedure B starting from **2h**; yield: 98%; $[\alpha]^{20}_D$= - 16.8 (c 1.0, $CHCl_3$); ^{1}H NMR (400 MHz, $CDCl_3$) δ 6.60 (s, 2H), 4.84 (app. t, 1H), 3.87 (s, 6H), 3.83 (s, 3H), 1.66 (dd, J_2 = 15.7 Hz, J_3 = 8.0 Hz, 1H), 1.51 (dd, J_2 = 15.7 Hz, J_3 = 8.0 Hz, 1H), 1.22 (s, 12H); ^{13}C NMR (101 MHz, $CDCl_3$) δ 156.6 (1C), 154.0 (2C), 138.4 (1C), 135.6 (1C), 103.7 (2C), 84.6 (2C), 61.5 (1C), 56.8 (2C), 46.6 (1C), 25.5 (2C), 25.3 (2C), (1C missing due to boron-quadrupole-induced relaxation); ^{11}B NMR (128 MHz, $CDCl_3$) δ 32.6; HRMS (ESI) calcd for $C_{18}H_{26}BNNaO_5^+$ $[M+Na]^+$ 370.1796, found 370.1799

(R)-1-benzyl-3-isocyano-3-((4,4,5,5-tetramethyl-1,3,2-dioxaborolan-2-yl)methyl)indolin-2-one **(3i)**

Synthetized following a modified general procedure B ($POCl_3$ 1.2eq instead of 1.5 eq and DCM 0.05M instead of 0.1M for 1 hour instead of 1.5 hours) starting from **2i**; yield: 93%; $[\alpha]^{20}_D$= + 7.4 (c 1.0, $CHCl_3$); ^{1}H NMR (400 MHz, $CDCl_3$) δ 7.54 (app d, J_2= 7.6 Hz, J_3= 1.5 Hz, 1H), 7.41-7.27 (m, 5H), 7.25 (app t, J_2= 7.6 Hz, J_3= 1.5 Hz, 1H), 7.09 (app t, J_2= 7.6 Hz, J_3= 1.5 Hz, 1H), 6.73 (d, J= 7.6 Hz, 1H), 4.98 (d, J= 15.6 Hz, 1H), 4.83 (d, J= 15.6 Hz, 1H), 2.05 (d, J= 16.3 Hz, 1H), 1.96 (d, J= 16.3 Hz, 1H), 1.07 (s, 6H), 0.94 (s, 6H); ^{13}C NMR (101 MHz, $CDCl_3$) δ 171.5 (1C), 157.9 (1C), 142.4 (1C), 135.0 (1C), 130.4 (1C), 128.9 (2C), 128.7 (1C), 127.9 (1C), 127.5 (2C), 123.8 (1C), 123.5 (1C), 109.7 (1C), 83.9 (2C), 60.9 (1C), 44.7 (1C), 24.7 (2C), 24.2 (2C), (1C missing due to boron-quadrupole-induced relaxation); ^{11}B NMR (128 MHz, $CDCl_3$) δ 31.5; HRMS (ESI) calcd for $C_{23}H_{25}BN_2NaO_3^+$ $[M+Na]^+$ 411.1850, found 411.1858

(S)-N-isopropyl-N-(2-oxo-2-((1-phenyl-2-(4,4,5,5-tetramethyl-1,3,2-dioxaborolan-2-yl)ethyl)amino)ethyl)cinnamamide **(4a)**

Synthesized following the General procedure C starting from paraformaldehyde, cinnamic acid, propan-2-amine and **3a**; yield: 42%; FC (dichlorometane:ethyl acetate=8:2); $[\alpha]^{20}_D$= - 30.5 (c 1.0, $CHCl_3$); ^{1}H NMR (400 MHz, $CDCl_3$, rotamers mixture 65:35) δ 7.82-7.78 (m, 2H), 7.58-7.07 (m, 10H), 6.97 (d, J_3 =15.4 Hz, 0.35H), 6.65 (d, J_3 =15.4 Hz, 0.65H), 5.43 (br s, 0.65H), 5.30-5.28 (m, 0.35H), 5.10-5.04 (m, 0.65H), 4.41-4.35 (m, 0.35H), 4.11-3.95 (m, 2H), 1.34 (d, J_3 =6.3 Hz, 2H), 1.26-1.20 (m, 6H), 1.16-1.10 (m, 12H); ^{13}C NMR (101 MHz, $CDCl_3$, rotamers mixture 65:35) δ 169.9 and 169.0 (1C), 168.0 and 167.5 (1C), 145.0 and 144.4 (1C), 144.2 and 143.9 (1C), 135.7 and 135.4 (1C), 130.6 (1C), 129.5 (2C), 129.0 and 128.9 (2C), 128.7 and 128.5 (2C), 127.6 and 127.4 (1C), 126.9 and 126.5 (2C), 118.0 and 117.7 (1C), 84.6 and 84.0 (2C), 50.1 and 49.9 (1C), 47.0 and 46.7 (1C), 45.9 (1C), 25.4 (2C), 25.1 (2C), 21.8 (1C), 20.7-20.4 (1C), (1C missing due to boron-quadrupole-induced relaxation); ^{11}B NMR (128 MHz, $CDCl_3$) δ 33.7; HRMS (ESI) calcd for $C_{28}H_{37}BN_2NaO_4^+$ $[M+Na]^+$ 492.2738, found 492.2743

(S)-N-(2-oxo-2-((1-phenyl-2-(4,4,5,5-tetramethyl-1,3,2-dioxaborolan-2-yl)ethyl)amino)ethyl)-N-phenylcinnamamide **(4b)**

Synthesized following the General procedure C starting from paraformaldehyde, cinnamic acid, aniline and **3a**; yield: 37%; FC (dichlorometane:ethyl acetate=8:2); $[\alpha]^{20}_D$= + 15.9 (c 1.0, $CHCl_3$); ^{1}H NMR (400 MHz, $CDCl_3$) δ 7.79 (d, J_3 =15.7 Hz, 1H), 7.46-7.30 (m, 14H), 7.28-7.19 (m, 2H), 6.42 (d, J_3 =15.7 Hz, 1H), 5.40-5.35 (m, 1H), 4.57-4.39 (m, 2H), 1.55 (dd, J_2 = 15.6 Hz, J_3 = 5.8 Hz, 1H), 1.42 (dd, J_2 = 15.6 Hz, J_3 = 5.8 Hz, 1H), 1.11 (s, 12H); ^{13}C NMR (101 MHz, $CDCl_3$) δ

168.2 (1C), 167.3 (1C), 144.0 (1C), 143.8 (1C), 142.9 (1C), 135.5 (1C), 130.5 (1C), 130.4 (2C), 129.4 (2C), 129.0 (2C), 128.8 (1C), 128.6-128.5 (4C), 127.5 (1C), 126.8 (2C), 118.6 (1C), 84.2 (2C), 55.0 (1C), 50.1 (1C), 25.4 (2C), 25.2 (2C), (1C missing due to boron-quadrupole-induced relaxation); ^{11}B NMR (128 MHz, $CDCl_3$) δ 33.5; HRMS (ESI) calcd for $C_{31}H_{35}BN_2NaO_4^+$ [M+Na]$^+$ 533.2582, found 533.2589

(methyl N-cinnamoyl-N-(2-oxo-2-(((S)-1-phenyl-2-(4,4,5,5-tetramethyl-1,3,2-dioxaborolan-2-yl)ethyl)amino)ethyl)-D-valinate **(4c)**

Synthesized following the General procedure C starting from paraformaldehyde, cinnamic acid, methyl L-valinate hydrochloride, triethylamine (1eq) and **3a**; yield: 50%; FC (dichlorometane:ethyl acetate=9:1); $[\alpha]^{20}_{D}$= - 64.7 (c 1.0, $CHCl_3$); ^{1}H NMR (400 MHz, $CDCl_3$) δ 8.17 (d, J =8.0 Hz, 1H), 7.73 (d, J =15.2 Hz, 1H), 7.42-7.40 (m, 2H), 7.38-7.35 (m, 3H), 7.31-7.30 (m, 2H), 7.18-7.06 (m, 3H), 6.61 (d, J =15.2 Hz, 1H), 5.37-5.32 (m, 1H), 4.36-4.29 (m, 2H), 4.05 (d, J =18.2 Hz, 1H), 3.64 (s, 3H), 2.54-2.45 (m, 1H), 1.44 (dd, J_2 = 15.7 Hz, J_3 = 5.9 Hz, 1H), 1.34 (dd, J_2 = 15.7 Hz, J_3 = 5.9 Hz, 1H), 1.14-1.10 (m, 15H), 0.96-0.95 (d, J = 6.7 Hz, 3H); ^{13}C NMR (101 MHz, $CDCl_3$) δ 171.7 (1C), 167.5 (1C), 167.3 (1C), 145.3 (1C), 143.4 (1C), 134.5 (1C), 130.2 (1C), 128.9 (2C), 128.3 (2C), 128.2 (2C), 127.0 (1C), 126.4 (2C), 115.9 (1C), 83.5 (2C), 66.3 (1C), 52.2 (2C), 49.8 (1C), 28.7 (1C), 24.7 (2C), 24.5 (2C), 20.8 (1C), 19.5 (1C), (1C missing due to boron-quadrupole-induced relaxation); ^{11}B NMR (128 MHz, $CDCl_3$) δ 33.1; HRMS (ESI) calcd for $C_{31}H_{41}BN_2NaO_6^+$ [M+Na]$^+$ 571.2950, found 571.2959

(S)-N-isopropyl-N-(2-oxo-2-((1-phenyl-2-(4,4,5,5-tetramethyl-1,3,2-dioxaborolan-2-yl)ethyl)amino)ethyl)pent-4-enamide **(4d)**

Synthesized following the General procedure C starting from paraformaldehyde, pent-4-enoic acid, propan-2-amine and **3a**; yield: 36%; FC (dichlorometane:ethyl acetate=8:2); $[\alpha]^{20}_{D}$= - 8.0 (c 1.0, $CHCl_3$); ^{1}H NMR (400 MHz, $CDCl_3$, rotamers mixture 55:45) δ 7.50 (d, J_3 =8.5 Hz, 1H) 7.30-7.20 (m, 5H), 5.93-5.74 (m, 1H), 5.44-5.39 (m, 0.45H), 5.29-5.23 (m, 0.55H), 5.11-4.92 (m, 2.45H), 4.17-4.10 (m, 0.55H), 3.99-3.86 (m, 2H), 2.55-2.30 (m, 4H), 1.50 (dd, J_2 = 15.6 Hz, J_3 = 6.1 Hz, 1H), 1.34 (dd, J_2 = 15.6 Hz, J_3 = 6.1 Hz, 1H), 1.19-1.12 (m, 18H); ^{13}C NMR (101 MHz, $CDCl_3$, rotamers mixture 55:45) δ 173.5 (1C), 169.8 and 168.8 (1C), 144.1 and 143.7 (1C), 137.9 and 137.7 (1C), 129.1 (1C), 128.9 (1C), 127.9 and 127.5 (1C), 126.9 (1C), 126.7 (1C), 116.2 (1C), 84.6 and 84.0 (2C), 50.1 and 47.0 (1C), 49.8 and 49.5 (1C), 46.1 and 45.7 (1C), 34.0 and 33.4 (1C), 30.0 and 29.9 (1C), 25.4 (2C), 25.2 (2C), 21.6 (1C), 20.7-20.5 (1C), (1C missing due to boron-quadrupole-induced relaxation); ^{11}B NMR (128 MHz, $CDCl_3$) δ 33.3; HRMS (ESI) calcd for $C_{24}H_{37}BN_2NaO_4^+$ [M+Na]$^+$ 451.2738, found 451.2739

tert-butyl ((S)-1-oxo-1-((2-oxo-2-(((S)-1-phenyl-2-(4,4,5,5-tetramethyl-1,3,2-dioxaborolan-2-yl)ethyl)amino)ethyl)(phenyl)amino)propan-2-yl)carbamate **(4e)**

Synthesized following the General procedure C starting from paraformaldehyde, (tert-butoxycarbonyl)-L-alanine, aniline and **3a**; yield: 43%; FC (from dichlorometane:ethyl acetate=8:2 to dichlorometane:ethyl acetate=7:3); $[\alpha]^{20}_{D}$= + 41.5 (c 1.0, $CHCl_3$); ^{1}H NMR (400 MHz, $CDCl_3$) δ 7.40-7.21 (m, 10H), 6.97 (br d, J =8.0 Hz, 1H), 5.37-5.31 (m, 1H), 5.18 (br. s, 1H), 4.47 (br d, J =15.7 Hz, 1H), 4.36-4.29 (m, 1H), 4.22 (br d, J =15.7 Hz, 1H), 1.56 (dd, J_2 = 15.6 Hz, J_3 = 5.9 Hz, 1H), 1.46 (s, 9H), 1.43-1.41 (m, 1H), 1.27 (d, J= 7.4 Hz, 3H), 1.14 (s, 12H); ^{13}C NMR (101 MHz, $CDCl_3$) δ 173.9 (1C), 167.0 (1C), 155.1 (1C), 143.5 (1C), 141.6 (1C), 130.0 (2C), 128.6 (1C), 128.3

(2C), 128.0 (2C), 127.0 (1C), 126.4 (2C), 83.4 (2C), 79.6 (1C), 54.2 (1C), 49.8 (1C), 47.1 (1C), 28.4 (3C), 24.7 (2C), 24.6 (2C), 18.4 (1C), (1C missing due to boron-quadrupole-induced relaxation); ^{11}B NMR (128 MHz, $CDCl_3$) δ 33.0; HRMS (ESI) calcd for $C_{30}H_{42}BN_3NaO_6^+$ [M+Na]$^+$ 574.3059, found 574.3070

***2-(N-isopropylcinnamamido)-4-methyl-N-((S)-1-phenyl-2-(4,4,5,5-tetramethyl-1,3,2-dioxaborolan-2-yl)ethyl)pentanamide* (4fa)**

Synthesized following the General procedure C starting from 3-methylbutanal, cinnamic acid , propan-2-amine and **3a**; yield: 24%; FC (hexane:ethyl acetate=8:2); $[\alpha]^{20}_D$= + 62.3 (c 0.6, $CHCl_3$); ^{1}H NMR (400 MHz, $CDCl_3$) δ 7.69 (d, *J*= 15.3 Hz, 1H), 7.56-7.30 (m, 9H), 7.26-7-18 (m, 2H), 6.86 (d, *J*= 15.3 Hz, 1H), 5.42 (br. s, 1H), 5.22-5.17 (m, 1H), 4.18-4.12 (m, 1H), 2.24-2.13 (m, 1H), 1.82-1.81 (m, 1H), 1.64 (br s, 1H), 1.38-1.36 (m, 5H), 1.26-1.22 (m, 3H), 1.13 (s, 12H), 1.00-0.97 (m, 6H); ^{13}C NMR (101 MHz, $CDCl_3$) δ 172.2 (1C), 168.6 (1C), 144.7 (1C), 143.2 (1C), 135.8 (1C), 130.4 (1C), 129.6 (2C), 128.9 (2C), 128.4 (2C), 127.4 (1C), 127.1-126.7 (2C), 120.4 (1C), 84.0 (2C), 59.0 (1C), 50.6 (1C), 50.0 (1C), 39.3 (1C), 26.0 (1C), 25.3 (4C), 23.5 (1C), 23.4 (1C), 22.5 (1C), 21.5 (1C), (1C missing due to boron-quadrupole-induced relaxation); ^{11}B NMR (128 MHz, $CDCl_3$) δ 33.5; HRMS (ESI) calcd for $C_{32}H_{45}BN_2NaO_4^+$ [M+Na]$^+$ 555.3364, found 555.3381

***2-(N-isopropylcinnamamido)-4-methyl-N-((S)-1-phenyl-2-(4,4,5,5-tetramethyl-1,3,2-dioxaborolan-2-yl)ethyl)pentanamide* (4fb)**

Synthesized following the General procedure C starting from 3-methylbutanal, cinnamic acid , propan-2-amine and **3a**; yield: 24%; FC (hexane:ethyl acetate=8:2); $[\alpha]^{20}_D$= + 6.2 (c 0.6, $CHCl_3$); ^{1}H NMR (400 MHz, $CDCl_3$) δ 7.74 (d, *J*= 15.6 Hz, 1H), 7.55-7.33 (m, 8H), 7.27-7-18 (m, 3H), 6.84 (d, *J*= 15.6 Hz, 1H), 5.30 (br. s, 1H), 5.23-5.18 (m, 1H), 4.10-4.07 (m, 1H), 2.13-2.08 (m, 1H), 1.67-1.63 (m, 1H), 1.60-1.55 (m, 1H), 1.40-1.36 (m, 6H), 1.29-1.28 (m, 2H), 1.14 (s, 12H), 0.97-0.95 (m, 6H); ^{13}C NMR (101 MHz, $CDCl_3$) δ 171.9 (1C), 168.6 (1C), 144.7 (1C), 143.3 (1C), 135.9 (1C), 130.4 (1C), 129.6 (2C), 128.9 (2C), 128.5 (2C), 127.4 (1C), 127.1-126.7 (2C), 120.5 (1C), 83.9 (2C), 58.5 (1C), 50.6 (1C), 49.5 (1C), 39.0 (1C), 25.9 (1C), 25.3 (4C), 23.5 (1C), 23.4 (1C), 22.7 (1C), 22.5 (1C), (1C missing due to boron-quadrupole-induced relaxation); ^{11}B NMR (128 MHz, $CDCl_3$) δ 33.3; HRMS (ESI) calcd for $C_{32}H_{45}BN_2NaO_4^+$ [M+Na]$^+$ 555.3364, found 555.3375

***(S)-N-isopropyl-N-(2-((1-(2-methoxyphenyl)-2-(4,4,5,5-tetramethyl-1,3,2-dioxaborolan-2-yl)ethyl)amino)-2-oxoethyl)cinnamamide* (4g)**

Synthesized following the General procedure C starting from paraformaldehyde, cinnamic acid, propan-2-amine and **3b**; yield: 61%; FC (hexane:ethyl acetate= 8:2 to 9:1); $[\alpha]^{20}_D$= – 48.5 (c 1.0, $CHCl_3$); ^{1}H NMR (400 MHz, $CDCl_3$, rotamers mixture 70:30) δ 7.89-7.68 (m, 2H), 7.57 (s, 1H), 7.41-7.32 (m, 4H), 7.18-7.17 (m, 1H), 7.05-6.97 (m, 1H), 6.86-6.80 (m, 1H), 6.73-6.55 (m, 2H), 5.58-5.52 (m, 0.7H), 5.45-5.40 (m, 0.3H), 5.09-5.02 (m, 0.7H), 4.39-4.36 (m, 0.3H), 4.06-3.91 (m, 2H), 3.83 (s, 1H), 3.81 (s, 2H), 1.47 (dd, J_2 = 16.6 Hz, J_3 = 9.1 Hz, 1H), 1.36 (dd, J_2 = 16.6 Hz, J_3 = 9.1 Hz, 1H), 1.28-1.24 (m, 3H), 1.20-1.18 (m, 3H), 1.14-1.11 (m, 12H); ^{13}C NMR (101 MHz, $CDCl_3$, rotamers mixture 70:30) δ 167.8 (1C), 167.0 (1C), 156.5 (1C), 144.1 and 143.6 (1C), 134.7 (1C), 130.7 (1C), 129.9 (1C), 128.9 (1C), 128.8 (1C), 128.2 (1C), 128.1 (1C), 127.8 (1C), 127.3 (1C), 120.2 (1C), 117.4 and 117.1 (1C), 110.5 (1C), 83.6 and 83.1 (2C), 55.1 (1C), 49.2 and 45.4 (1C), 47.5 and 47.1 (1C), 46.5 and 46.0 (1C), 24.8 (2C), 24.6 (2C), 20.0 and 19.9 (2C), (1C missing

due to boron-quadrupole-induced relaxation); ^{11}B NMR (128 MHz, $CDCl_3$) δ 33.7; HRMS (ESI) calcd for $C_{29}H_{39}BN_2NaO_5^+$ $[M+Na]^+$ 529.4392, found 529.4405

***(S)-N-isopropyl-N-(2-((1-(4-nitrophenyl)-2-(4,4,5,5-tetramethyl-1,3,2-dioxaborolan-2-yl)ethyl)amino)-2-oxoethyl)cinnamamide* (4h)**

Synthesized following the General procedure C starting from paraformaldehyde, cinnamic acid, propan-2-amine and **3c**; yield: 27%; FC (dichloromethane:ethyl acetate=6:4 to 1:1); $[\alpha]^{20}_D$= - 24.1 (c 0.1, $CHCl_3$); ^{1}H NMR (400 MHz, $CDCl_3$, rotamers mixture 55:45) δ 8.18 (d, J = 8.5 Hz, 1H), 7.85-7.79 (m, 3H), 7.58-7.52 (m, 3H), 7.40-7.37 (m, 3H), 6.99 (d, J = 15.7 Hz, 0.55H), 6.58 (d, J = 15.7 Hz, 0.45H), 5.43 (br. s, 0.55H), 5.34 (br. s, 0.45H), 5.14 (app. q, 0.45H), 4.42 (app. q, 0.55H), 4.15-3.96 (m, 2H), 1.52-1.43 (m, 2H), 1.28 (m, 6H), 1.18 (m, 6H), 1.12 (m, 6H); ^{13}C NMR (101 MHz, $CDCl_3$, rotamers mixture 55:45) δ 170.2 (1C), 169.4 (1C), 152.1 (1C), 151.4 (1C), 145.4 and 144.8 (1C), 131.2 (1C), 130.7 (1C), 129.6 (2C), 128.6 (2C), 127.8 (1C), 127.5 (1C), 124.3 (2C), 117.5 and 117.3 (1C), 85.0 and 84.4 (2C), 50.0 (1C), 49.9 (1C), 46.8 and 45.8 (1C), 25.4 (2C), 25.2 (2C), 21.9 and 21.8 (1C), 20.8 and 20.5 (1C), (1C missing due to boron-quadrupole-induced relaxation); ^{11}B NMR (128 MHz, $CDCl_3$) δ 31.6; HRMS (ESI) calcd for $C_{28}H_{36}BN_3NaO_6^+$ $[M+Na]^+$ 544.2589, found 544.2581

***(S)-N-isopropyl-N-(2-oxo-2-((2-(4,4,5,5-tetramethyl-1,3,2-dioxaborolan-2-yl)-1-(thiophen-2-yl)ethyl)amino)ethyl)cinnamamide* (4i)**

Synthesized following the General procedure C starting from paraformaldehyde, cinnamic acid, propan-2-amine and **3g**; yield: 37%; FC (hexane:ethyl acetate=6:4); $[\alpha]^{20}_D$= - 24.2 (c 0.6, $CHCl_3$); ^{1}H NMR (400 MHz, $CDCl_3$, rotamers mixture 60:40) δ 7.88 (d, J= 8.8 Hz, 1H), 7.75 (d, J = 13.8 Hz, 1H), 7.55 (s, 1H), 7.48-7.38 (m, 4H), 7.14-6.68 (m, 3.4H), 6.62 (d, J = 13.8 Hz, 0.6H), 5.67 (s, 0.6H), 5.58 (s, 0.4H), 5.06-5.00 (m, 0.6H), 4.43-4.36 (m, 0.4H), 4.15-3.96 (m, 2H), 1.57 (dd, J_2 = 16.0 Hz, J_3 = 5.8 Hz, 1H), 1.45 (dd, J_2 = 16.0 Hz, J_3 = 5.8 Hz, 1H), 1.27 (s, 6H), 1.19 (s, 6H), 1.13 (s, 6H); ^{13}C NMR (101 MHz, $CDCl_3$, rotamers mixture 60:40) δ 169.0 (1C), 167.7 (1C), 144.9 and 144.4 (1C), 130.6 (2C), 129.5 (3C), 128.7 (1C), 128.5 (1C), 127.2 (1C). 124.6 and 124.4 (2C), 118.2 and 117.6 (1C), 84.7 and 84.2 (2C), 49.9 and 46.6 (1C), 47.4 and 46.1 (1C), 46.6 and 46.1 (1C), 25.3 (2C), 25.0 (2C), 21.83 and 20.6 (1C), 21.77 and 20.4 (1C), (1C missing due to boron-quadrupole-induced relaxation); ^{11}B NMR (128 MHz, $CDCl_3$) δ 33.3; HRMS (ESI) calcd for $C_{26}H_{35}BN_2NaO_4S^+$ $[M+Na]^+$ 505.2303, found 505.2296

***(S)-N-isopropyl-N-(2-((1-(naphthalen-1-yl)-2-(4,4,5,5-tetramethyl-1,3,2-dioxaborolan-2-yl)ethyl)amino)-2-oxoethyl)cinnamamide* (4j)**

Synthesized following the General procedure C starting from paraformaldehyde, cinnamic acid, propan-2-amine and **3f**; yield: 55%; FC (hexane:ethyl acetate=7:3); $[\alpha]^{20}_D$= - 30.8 (c 1.0, $CHCl_3$); ^{1}H NMR (400 MHz, $CDCl_3$, rotamers mixture 65:35) δ 8.01-7.23 (m, 13H), 6.97 (d, J = 15.6 Hz, 0.35H), 6.65 (d, J = 15.6 Hz, 0.65H), 5.60 (br. s, 0.65H), 5.49-5.45 (m, 0.35H), 5.12-5.07 (m, 0.65H), 4.39 (br. s, 0.35H), 4.17-3.96 (m, 2H), 1.57-1.43 (m, 2H), 1.27-1.26 (m, 3H), 1.21-1.16 (m, 3H), 1.16-1.08 (m, 12H); ^{13}C NMR (101 MHz, $CDCl_3$, rotamers mixture 65:35) δ 170.0 and 169.1 (1C), 168.1 and 167.6 (1C), 145.1 and 144.5 (1C), 141.8 and 141.5 (1C), 135.7 and 135.4 (1C), 133.8 and 133.2 (1C), 130.6 (1C), 129.5 (2C), 129.1 (1C), 128.7-128.5 (4C), 128.1 (1C), 126.6 and 126.5 (1C), 126.3 and 126.1 (1C), 125.6 and 124.9 (1C), 125.2 (1C), 118.0 and 117.7 (1C), 84.7 and 84.1 (2C), 50.1 and 49.9 (1C), 47.1 and 46.8 (1C), 46.0 (1C), 25.4 (2C), 25.1 (2C), 21.9 and 20.7

(1C), 21.8 and 20.4 (1C), (1C missing due to boron-quadrupole-induced relaxation); ^{11}B NMR (128 MHz, $CDCl_3$) δ 34.3; HRMS (ESI) calcd for $C_{32}H_{39}BN_2NaO_4^+$ $[M+Na]^+$ 549.2895, found 549.2906

(S)-2-(2-oxoazetidin-1-yl)-N-(1-phenyl-2-(4,4,5,5-tetramethyl-1,3,2-dioxaborolan-2-yl)ethyl)acetamide **(5a)**

Synthesized following a modified General procedure C (heating the reaction at 60°C for 8 hours instead of room temperature for 48 hours) starting from paraformaldehyde, 3-aminopropanoic acid and **3a**; yield: 36%; FC (ethyl acetate); $[\alpha]^{20}_D$= - 16.1 (c 1.0, $CHCl_3$); ^{1}H NMR (400 MHz, $CDCl_3$) δ 7.34-7.29 (m, 4H), 7.26.7.21 (m, 1H), 6.99 (d, *J* =8.0 Hz, 1H), 5.36-5.31 (m, 1H), 3.97 (d, *J* =16.9 Hz, 1H), 3.92 (d, *J* =16.9 Hz, 1H), 3.46 (dd, J_2 = 9.5 Hz, J_3 = 3.4 Hz, 1H), 3.41 (dd, J_2 = 9.5 Hz, J_3 = 3.4 Hz, 1H), 3.08 (app. t, 2H), 1.52 (dd, J_2 = 16.1 Hz, J_3 = 5.6 Hz, 1H), 1.41 (dd, J_2 = 16.1 Hz, J_3 = 5.6 Hz, 1H), 1.17 (s, 12H); ^{13}C NMR (101 MHz, $CDCl_3$) δ 168.8 (1C), 166.9 (1C), 143.7 (1C), 129.1 (2C), 127.8 (1C), 126.7 (2C), 84.3 (2C), 50.1 (1C), 46.9 (1C), 41.2 (1C), 38.3 (1C), 25.5 (2C), 25.3 (2C), (1C missing due to boron-quadrupole-induced relaxation); ^{11}B NMR (128 MHz, $CDCl_3$) δ 33.7; HRMS (ESI) calcd for $C_{19}H_{27}BN_2NaO_4^+$ $[M+Na]^+$ 381.1956, found 381.1950

(S)-N-(1-(2-methoxyphenyl)-2-(4,4,5,5-tetramethyl-1,3,2-dioxaborolan-2-yl)ethyl)-2-(2-oxoazetidin-1-yl)acetamide **(5b)**

Synthesized following a modified General procedure C (heating the reaction at 60°C for 8 hours instead of room temperature for 48 hours) starting from paraformaldehyde, 3-aminopropanoic acid and **3b**; yield: 39%; FC (dichloromethane:methanol=98:2 to 95:5); $[\alpha]^{20}_D$= – 23.6 (c 0.4, $CHCl_3$); ^{1}H NMR (400 MHz, $CDCl_3$) δ 7.25-7.18 (m, 3H), 6.91-6.87 (m, 2H), 5.44 (dd, J_2 = 14.6 Hz, J_3 = 7.0 Hz, 1H), 3.91-3.89 (m, 5H), 3.45-3.44 (m, 1H), 3.43-3.38 (m, 1H), 3.12-3.03 (m, 2H), 1.52 (dd, J_2 = 15.6 Hz, J_3 = 7.2 Hz, 1H), 1.44 (dd, J_2 = 15.6 Hz, J_3 = 7.2 Hz, 1H), 1.17 (s, 12H); ^{13}C NMR (101 MHz, $CDCl_3$) δ 168.6 (1C), 166.3 (1C), 157.4 (1C), 131.4 (1C), 129.0 (1C), 128.5 (1C), 121.1 (1C), 111.5 (1C), 84.1 (2C), 55.9 (1C), 48.4 (1C), 46.9 (1C), 41.1 (1C), 38.3 (1C), 25.5 (2C), 25.4 (2C), (1C missing due to boron-quadrupole-induced relaxation); ^{11}B NMR (128 MHz, $CDCl_3$) δ 33.7; HRMS (ESI) calcd for $C_{20}H_{29}BN_2NaO_5^+$ $[M+Na]^+$ 411.2062, found 411.2077

(S)-2-(isopropyl(2-nitrophenyl)amino)-N-(1-(naphthalen-1-yl)-2-(4,4,5,5-tetramethyl-1,3,2-dioxaborolan-2-yl)ethyl)acetamide **(6)**

Synthesized following a modified General procedure C (2-NO_2-phenol instead of carboxylic acid and heated at 60°C instead of room temperature) starting from paraformaldehyde, 2-nitrophenol, propan-2-amine and **3f**; yield: 34%; FC (hexane:ethyl acetate=7:3); $[\alpha]^{20}_D$= - 44.5 (c 0.7, $CHCl_3$); ^{1}H NMR (400 MHz, $CDCl_3$) δ 8.08 (d, J_3 = 8.2 Hz, 1H), 7.76-7.73 (m, 1H), 7.69 (dd, J_3 = 8.2 Hz, J_4 = 1.6 Hz, 1H), 7.68-7.66 (m, 1H), 7.62 (d, J_3 = 8.5 Hz, 1H), 7.55 (app. s, 1H), 7.44-7.40 (m, 3H), 7.22 (dd, J_3 = 8.2 Hz, J_4 = 0.8 Hz, 1H), 7.18 (dd, J_3 = 8.5 Hz, J_4 = 1.6 Hz, 1H), 7.13-7-09 (m, 1H), 5.37-5.31 (m, 1H), 3.88-3.77 (m, 2H), 3.29 (sept, J_3 = 6.5 Hz, 1H), 1.44 (d, J_3 = 7.7 Hz, 2H), 1.18 (d, J_3 = 6.5 Hz, 3H), 1.07 (s, 12H), 1.04 (d, J_3 = 6.5 Hz, 3H); ^{13}C NMR (101 MHz, $CDCl_3$) δ 169.0 (1C), 146.2 (1C), 143.4 (1C), 141.1 (1C), 133.2 (1C), 132.5 (1C), 127.9 (2C), 127.6 (1C), 127.5 (1C), 125.7 (1C), 125.6 (1C), 125.4 (1C), 124.7 (1C), 124.6 (1C), 123.3 (1C), 123.1 (1C), 83.3 (2C), 57.2 (1C), 50.0 (1C), 48.1 (1C), 24.6 (4C), 19.5 (1C), 18.8 (1C), (1C missing due to boron-quadrupole-induced relaxation); ^{11}B NMR (128 MHz, $CDCl_3$) δ 34.7; HRMS (ESI) calcd for $C_{29}H_{36}BN_3NaO_5^+$ $[M+Na]^+$ 540.2640, found 540.2631

***(S)-2-oxo-2-((2-(4,4,5,5-tetramethyl-1,3,2-dioxaborolan-2-yl)-1-(3,4,5-trimethoxyphenyl)ethyl)amino)ethyl cinnamate* (7)**

Synthesized following a modified General procedure C (heating the reaction at 60°C for 8 hours instead of room temperature for 48 hours) starting from cinnamic acid, formaldehyde and **3h**; yield: 51%; FC (hexane:ethyl acetate=7:3); $[\alpha]^{20}_{D}$= + 19.6 (c 1.0, $CHCl_3$); ^1H NMR (400 MHz, $CDCl_3$) δ 7.83 (d, *J*= 16.2 Hz, 1H), 7.57-7.55 (m, 2H), 7.45-7.43 (m, 3H), 7.12 (d, *J*= 7.9 Hz, 1H), 6.58-6.54 (m, 3H), 5.38-5.33 (m, 1H), 4.77 (d, *J*= 15.3 Hz, 2H), 3.87 (s, 6H), 3.82 (s, 3H), 1.53 (dd, J_2 = 15.6 Hz, J_3 = 6.5 Hz, 1H), 1.43 (dd, J_2 = 15.6 Hz, J_3 = 6.5 Hz, 1H), 1.13 (s, 12H); ^{13}C NMR (101 MHz, $CDCl_3$) δ 167.0 (1C), 166.3 (1C), 153.8 (2C), 147.4 (1C), 139.5 (1C), 137.7 (1C), 134.5 (1C), 131.6 (1C), 129.7 (2C), 128.9 (2C), 117.2 (1C), 103.9 (2C), 84.3 (2C), 63.8 (1C), 61.4 (1C), 56.8 (2C), 49.7 (1C), 25.5 (2C), 25.3 (2C), (1C missing due to boron-quadrupole-induced relaxation); ^{11}B NMR (128 MHz, $CDCl_3$) δ 33.3; HRMS (ESI) calcd for $C_{28}H_{36}BNNaO_8^+$ $[M+Na]^+$ 548.2426, found 548.2415

***(S)-2-(4-(1H-indole-2-carbonyl)piperazin-1-yl)-N-(1-(3-bromophenyl)-2-(4,4,5,5-tetramethyl-1,3,2-dioxaborolan-2-yl)ethyl)acetamide* (8)**

Synthesized following the General procedure C starting from 1H-indole-2-carboxylic acid, formaldehyde, piperazine and **3d**; yield: 56%; FC (ethyl acetate:dichloromethane=8:2); $[\alpha]^{20}_{D}$= -12.1 (c 1.0, $CHCl_3$); ^1H NMR (400 MHz, $CDCl_3$) δ 9.58 (br. s, 1H), 7.97 (br. s, 1H), 7.68 (d, *J*= 7.8 Hz, 1H), 7.51 (s, 1H), 7.46 (d, *J*= 8.0 Hz, 1H), 7.39 (d, *J*= 7.4 Hz, 1H), 7.33-7.27 (m, 2H), 7.22-7.15 (m, 2H), 6.80 (s, 1H), 5.36-5.35 (m, 1H), 4.05 (br s, 4H), 3.15 (br s, 2H), 2.70 (br s, 4H), 1.49 (dd, J_2 = 15.1 Hz, J_3 = 5.9 Hz, 1H), 1.42 (dd, J_2 = 15.1 Hz, J_3 = 5.9 Hz, 1H), 1.22 (s, 12H); ^{13}C NMR (101 MHz, $CDCl_3$) δ 168.7 (1C), 162.6 (1C), 146.3 (1C), 141.8 (1C), 135.8 (1C), 130.1 (2C), 129.1-129.0 (1C), 127.4 (1C), 125.2 (1C), 124.6 (1C), 122.5 (1C), 121.9 (1C), 120.7 (1C), 111.9 (1C), 105.4 (1C), 83.7 (2C), 61.6 (1C), 53.5 (4C), 48.5 (1C), 25.0 (2C), 24.8 (2C), (1C missing due to boron-quadrupole-induced relaxation); ^{11}B NMR (128 MHz, $CDCl_3$) δ 33.9; HRMS (ESI) calcd for $C_{29}H_{36}BBrN_4NaO_4^+$ $[M+Na]^+$ 617.1905, found 617.1915

***(S)-N-((1-(1-phenyl-2-(4,4,5,5-tetramethyl-1,3,2-dioxaborolan-2-yl)ethyl)-1H-tetrazol-5-yl)methyl)propan-2-amine* (9)**

Synthesized following a modified General procedure C (TMS-N_3 instead of carboxylic acid) starting from paraformaldehyde (1.3eq), trimethylsilyl azide (1.3eq), propan-2-amine (1.3eq) and **3a** (1eq); yield: 36%; FC (ethyl acetate); $[\alpha]^{20}_{D}$= + 10.1 (c 1.0, $CHCl_3$); ^1H NMR (400 MHz, $CDCl_3$) δ 7.37-7.29 (m, 5H), 6.00-5.96 (m, 1H), 4.05 (d, *J* =14.8 Hz, 1H), 3.96 (d, *J* =14.8 Hz, 1H), 2.81-2.72 (m, 1H), 2.25 (dd, J_2 = 16.1 Hz, J_3 = 10.1 Hz, 1H), 1.86 (dd, J_2 = 16.1 Hz, J_3 = 10.1 Hz, 1H), 1.16 (s, 6H), 1.09-1.08 (m, 9H), 1.00 (d, *J* =6.1 Hz, 3H), (one exchangeable proton missing); ^{13}C NMR (101 MHz, $CDCl_3$) δ 154.0 (1C), 140.8 (1C), 129.6 (2C), 129.1 (1C), 127.3 (2C), 84.4 (2C), 60.3 (1C), 48.9 (1C), 40.7 (1C), 25.4 (2C), 25.2 (2C), 23.2 (1C), 23.1 (1C), (1C missing due to boron-quadrupole-induced relaxation); ^{11}B NMR (128 MHz, $CDCl_3$) δ 32.6; HRMS (ESI) calcd for $C_{19}H_{30}BN_5NaO_2^+$ $[M+Na]^+$ 394.2385, found 394.2380

***(S)-N-isopropyl-2-(1-(1-phenyl-2-(4,4,5,5-tetramethyl-1,3,2-dioxaborolan-2-yl)ethyl)-1H-tetrazol-5-yl)propan-2-amine* (10)**

Synthesized following a modified General procedure C (TMS-N_3 instead of carboxylic acid) starting from acetone (1.3eq), trimethylsilyl azide (1.3eq), propan-2-amine (1.3eq) and **3a** (1 eq); yield: 44%; FC (hexane:ethyl acetate=8:2); $[\alpha]^{20}_D$= + 30.6 (c 1.0, $CHCl_3$); ^{1}H NMR (400 MHz, $CDCl_3$) δ 7.46-7.45 (m, 2H), 7.34-7.25 (m, 3H), 6.90 (app. t, 1H), 3.11-3.02 (m, 1H), 2.01-1.98 (m, 2H), 1.73 (s, 3H), 1.36 (s, 3H), 1.15 (s, 6H), 1.09 (s, 6H), 0.92 (d, *J*= 6.2 Hz, 3H), 0.86 (d, *J*= 6.2 Hz, 3H), (one exchangeable proton missing); ^{13}C NMR (101 MHz, $CDCl_3$) δ 160.3 (1C), 142.0 (1C), 129.2 (2C), 128.5 (1C), 127.9 (2C), 84.2 (2C), 61.0 (1C), 53.6 (1C), 44.5 (1C), 29.1 (2C), 28.6 (2C), 25.3-25.1 (4C), (1C missing due to boron-quadrupole-induced relaxation); ^{11}B NMR (128 MHz, $CDCl_3$) δ 32.8; HRMS (ESI) calcd for $C_{21}H_{34}BN_5NaO_2^+$ $[M+Na]^+$ 422.2698, found 422.2690

***(S)-2-phenyl-N-(1-phenyl-2-(4,4,5,5-tetramethyl-1,3,2-dioxaborolan-2-yl)ethyl)imidazo[1,2-a]pyridin-3-amine* (11a)**

Synthesized following a modified General procedure C (BDMSB as catalyst and no carboxylic acid) starting from pyridin-2-amine, benzaldehyde, BDMSB (0.1eq) and **3a**; yield: 50%; FC (ethyl acetate:hexane=7:3); $[\alpha]^{20}_D$= - 34.5 (c 1.0, $CHCl_3$); ^{1}H NMR (400 MHz, $CDCl_3$) δ 8.23 (d, *J*= 6.9 Hz, 1H), 7.87 (d, *J*= 7.5 Hz, 2H), 7.53 (d, *J*= 8.8 Hz, 1H), 7.44-7.40 (m, 2H), 7.35-7.31 (m, 1H), 7.16-7.10 (m, 6H), 6.73 (t, *J*= 6.9 Hz, 1H), 4.49-4.43 (m, 1H), 3.95 (d, *J*= 4.7 Hz, 1H), 1.52 (dd, J_2 = 15.6 Hz, J_3 = 8.0 Hz, 1H), 1.42 (dd, J_2 = 15.6 Hz, J_3 = 8.0 Hz, 1H), 1.18 (s, 6H), 1.17 (s, 6H); ^{13}C NMR (101 MHz, $CDCl_3$) δ 144.2 (2C), 142.0 (1C), 137.4 (1C), 134.9 (1C), 129.0 (2C), 128.8 (2C), 128.1 (2C), 128.0 (1C), 127.9 (1C), 127.5 (2C), 124.5 (1C), 123.7 (1C), 117.8 (1C), 112.0 (1C), 84.1 (2C), 58.8 (1C), 25.5 (2C), 25.3 (2C), (1C missing due to boron-quadrupole-induced relaxation); ^{11}B NMR (128 MHz, $CDCl_3$) δ 33.2; HRMS (ESI) calcd for $C_{27}H_{30}BN_3NaO_2^+$ $[M+Na]^+$ 462.2323, found 462.2332

(*S*)*-2-phenyl-N-(2-(4,4,5,5-tetramethyl-1,3,2-dioxaborolan-2-yl)-1-(3,4,5-trimethoxyphenyl)ethyl)imidazo[1,2-a]pyridin-3-amine* (11h)

Synthesized following a modified General procedure C (BDMSB as catalyst and no carboxylic acid) starting from pyridin-2-amine (1eq), benzaldehyde (1eq), BDMSB (0.1eq) and **3h**; yield: 59%; FC (ethyl acetate:hexane=1:1); $[\alpha]^{20}_D$= - 31.7 (c 1.0, $CHCl_3$); ^{1}H NMR (400 MHz, $CDCl_3$) δ 8.23 (d, *J*= 6.9 Hz, 1H), 7.84 (d, *J*= 7.2 Hz, 2H), 7.53 (d, *J*= 9.1 Hz, 1H), 7.43-7.39 (m, 1H), 7.33-7.31 (m, 1H), 7.14-7.10 (m, 1H), 6.76-6.73 (m, 1H), 6.25 (s, 2H), 4.42-4.37 (m, 1H), 3.99 (br.s, 1H), 3.72 (s, 3H), 3.63 (s, 6H), 1.53 (dd, J_2 = 15.5 Hz, J_3 = 8.3 Hz, 1H), 1.43 (dd, J_2 = 15.5 Hz, J_3 = 8.3 Hz, 1H), 1.23 (s, 6H), 1.22 (s, 6H); ^{13}C NMR (101 MHz, $CDCl_3$) δ 153.4 (2C), 142.0 (1C), 139.8 (2C), 137.8 (1C), 137.5 (1C), 134.9 (1C), 129.0 (2C), 128.2 (2C), 128.0 (1C), 124.5 (1C), 123.7 (1C), 117.9 (1C), 112.1 (1C), 104.4 (2C), 84.2 (2C), 61.4 (1C), 58.7 (1C), 56.6 (2C), 25.6 (2C), 25.4 (2C), (1C missing due to boron-quadrupole-induced relaxation); ^{11}B NMR (128 MHz, $CDCl_3$) δ 33.7; HRMS (ESI) calcd for $C_{30}H_{36}BN_3NaO_5^+$ $[M+Na]^+$ 552.2640, found 552.2649

***((S)-2-(2-(N-((S)-1-methoxy-3-methyl-1-oxobutan-2-yl)cinnamamido)acetamido)-2-phenylethyl)boronic acid* (12)**

Prepared following the General Procedure D starting from β-amido boronic ester **4c**; yield: 97%; $[\alpha]^{20}_D$= - 78.6 (c 0.3, Acetone); m.p.= 123-126 °C; ^{1}H NMR (400 MHz, Acetone d-6 + 1 drop of H_2O) δ 8.00 (br d, *J*= 6.9 Hz, 1H), 7.71 (br s, 1H), 7.58 (d, *J*= 15.4 Hz, 1H), 7.46-7.30 (m, 8H), 7.22-7.15 (m, 2H), 6.85 (app. d, 2H), 5.28-5.22 (app. q, 1H), 4.66 (d, *J*= 9.4 Hz, 1H), 4.40 (d, *J*=

17.5 Hz, 1H), 4.30 (d, *J*= 17.5 Hz, 1H), 3.68 (s, 3H), 2.37-2.30 (m, 1H), 1.41-1.36 (m, 2H), 1.03 (d, *J*= 6.7 Hz, 3H), 0.90 (d, *J*= 6.7 Hz, 3H); ^{13}C NMR (101 MHz, Acetone d-6 + 1 drop of H_2O) δ 171.9 (1C), 168.5 (1C), 167.4 (1C), 146.7 (1C), 143.3 (1C), 136.0 (1C), 130.2 (1C), 129.4 (2C), 129.0 (1C), 128.8 (2C), 128.6 (2C), 127.2 (1C), 127.0 (1C), 118.8 (1C), 64.6 (1C), 52.0 (1C), 51.1 (1C), 50.2 (1C), 28.3 (1C), 20.2 (1C), 19.2 (1C), (1C missing due to boron-quadrupole-induced relaxation); ^{11}B NMR (128 MHz, Acetone d-6 + 1 drop of H_2O) δ 33.3 ; HRMS (ESI) calcd for $C_{27}H_{35}BN_2NaO_6^+$ $[MB(OMe)_2+Na]^+$ 517.2486, found 517.2491.

***(S)-(2-(2-(cinnamoyloxy)acetamido)-2-(3,4,5-trimethoxyphenyl)ethyl)boronic acid* (13)**

Prepared following the General Procedure D starting from β-amido boronic ester **7**; yield: 98%; $[\alpha]^{20}_D$= + 13.1 (c 0.2, $CHCl_3$); ^{1}H NMR (400 MHz, $CDCl_3$, complex rotameric mixture: the section by section integration proves the overall number of protons) δ 7.82-7.39 (m, 7H), 6.61-6.54 (m, 2H), 6.50-6.44 (m, 1H), 5.24-4.67 (m, 3H), 3.97-3.82 (m, 9H), 1.28-0.91 (m, 2H), (two exchangeable protons missing); ^{13}C NMR (101 MHz, $CDCl_3$, complex rotameric mixture) δ 171.5-170.6 (1C), 165.9-165.6 (1C), 154.4-154.1 (2C), 149.1-148.5 (1C), 138.5-138.0 (1C), 134.2-134.1 (1C), 132.1-131.7 (2C), 129.7 (2C), 129.1 (2C), 116.2-115.6 (1C), 104.2-104.0 (2C), 63.5-62.6 (1C), 61.9-61.5 (1C), 58.6 (1C), 56.9-56.2 (2C), (1C missing due to boron-quadrupole-induced relaxation); ^{11}B NMR (128 MHz, $CDCl_3$) δ 33.0; HRMS (ESI) calcd for $C_{24}H_{30}BNNaO_8^+$ $[MB(OMe)_2+Na]^+$ 494.1956, found 494.1943.

***(S)-2-hydroxy-4-(3,4,5-trimethoxyphenyl)-1,5,2-oxazaborepan-6-one* (14)**

Prepared in a two-step procedure: In a round-bottom flask, compound **13** (45 mg, 0.085 mmol, 1eq) was dissolved in dry THF (1.7 mL, 0.05M), then aqueous NaOH 1M (172µL, 2.1eq) was added and the reaction stirred for 20 minutes at room temperature. The solvents were removed under reduced pressure, then the crude was dissolved in ethyl acetate (5 mL) and filtered through a pad of celite, washing with a small amount of ethyl acetate. The solvent was removed under reduced pressure and the product obtained treated following the General Procedure D for 2.5 hours to afford pure compound **14** after lyophilisation; yield: 34%; $[\alpha]^{20}_D$= + 32.3 (c 0.1, H_2O:DMSO 9:1); ^{1}H NMR (400 MHz, D_2O+3 drops of DMSO d_6) δ 6.73 (s, 2H), 5.01 (app. t, 1H), 4.06 (d, *J*= 16.7 Hz, 1H), 4.00 (d, *J*= 16.7 Hz, 1H), 3.84 (s, 6H), 3.73 (s, 3H), 1.38 (app. d, 2H), (two exchangeable protons missing); ^{13}C NMR (101 MHz, D_2O+3 drops of DMSO d_6) δ 174.1 (1C), 153.2 (2C), 141.3 (2C), 104.2 (2C), 61.5 (2C), 56.7 (2C), 51.5 (1C), (1C missing due to boron-quadrupole-induced relaxation); ^{11}B NMR (128 MHz, D_2O+3 drops of DMSO d_6) δ 19.6; HRMS (ESI+ dissolved in MeOH) calcd for $C_{15}H_{24}BNNaO_6^+$ $[MB(OMe)+Na+MeOH]^+$ 364.1544, found 364.1553; (ESI- dissolved in H_2O) calcd for $C_{13}H_{19}BNO_7^-$ $[MB(OH)_2]^-$ 312.1255, found 312, 1248.

Copies of ^{1}H, ^{13}C and ^{11}B NMR spectra

^{1}H NMR (400 MHz, $CDCl_3$, rotamers mixture 75:25) of compound **2a**

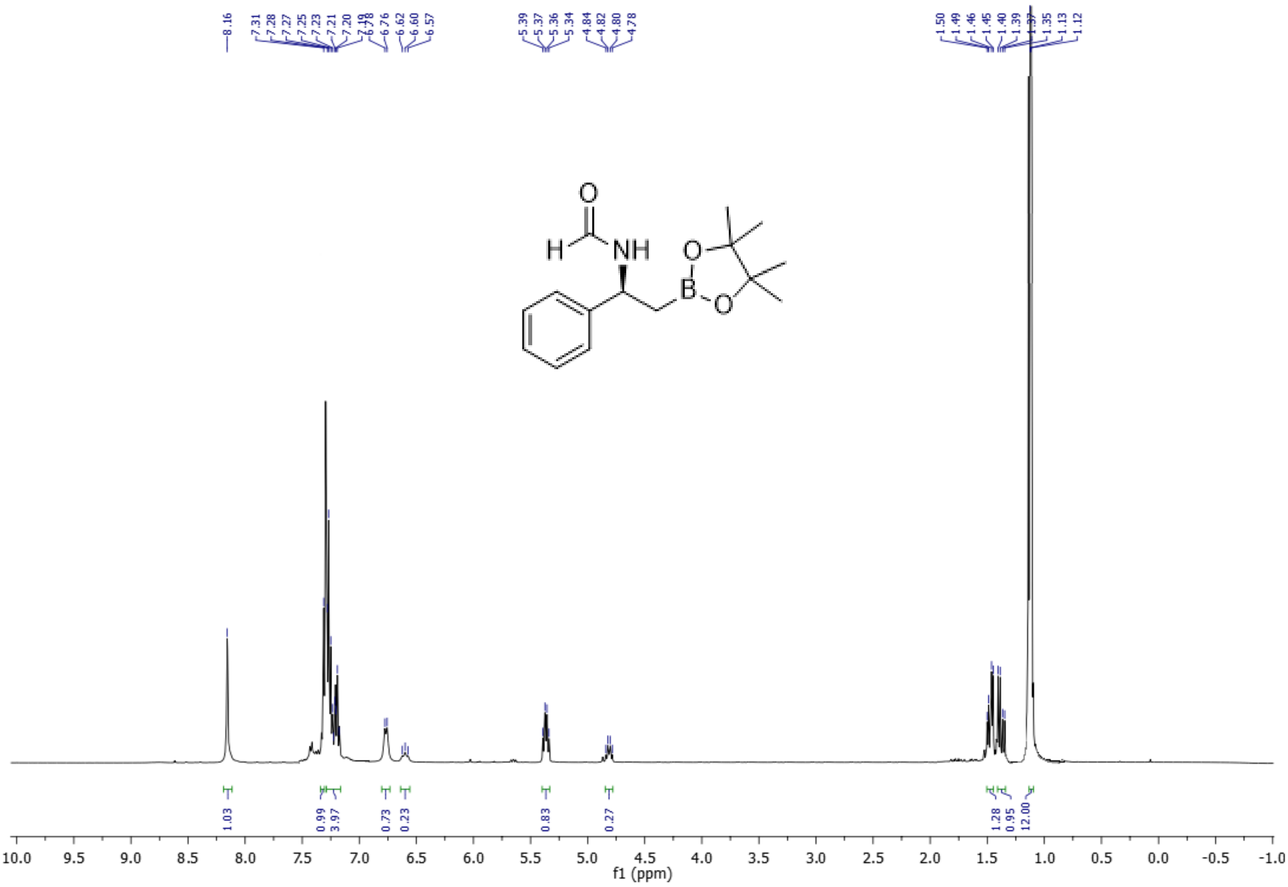

^{13}C NMR (101 MHz, $CDCl_3$, rotamers mixture 75:25) of compound **2a**

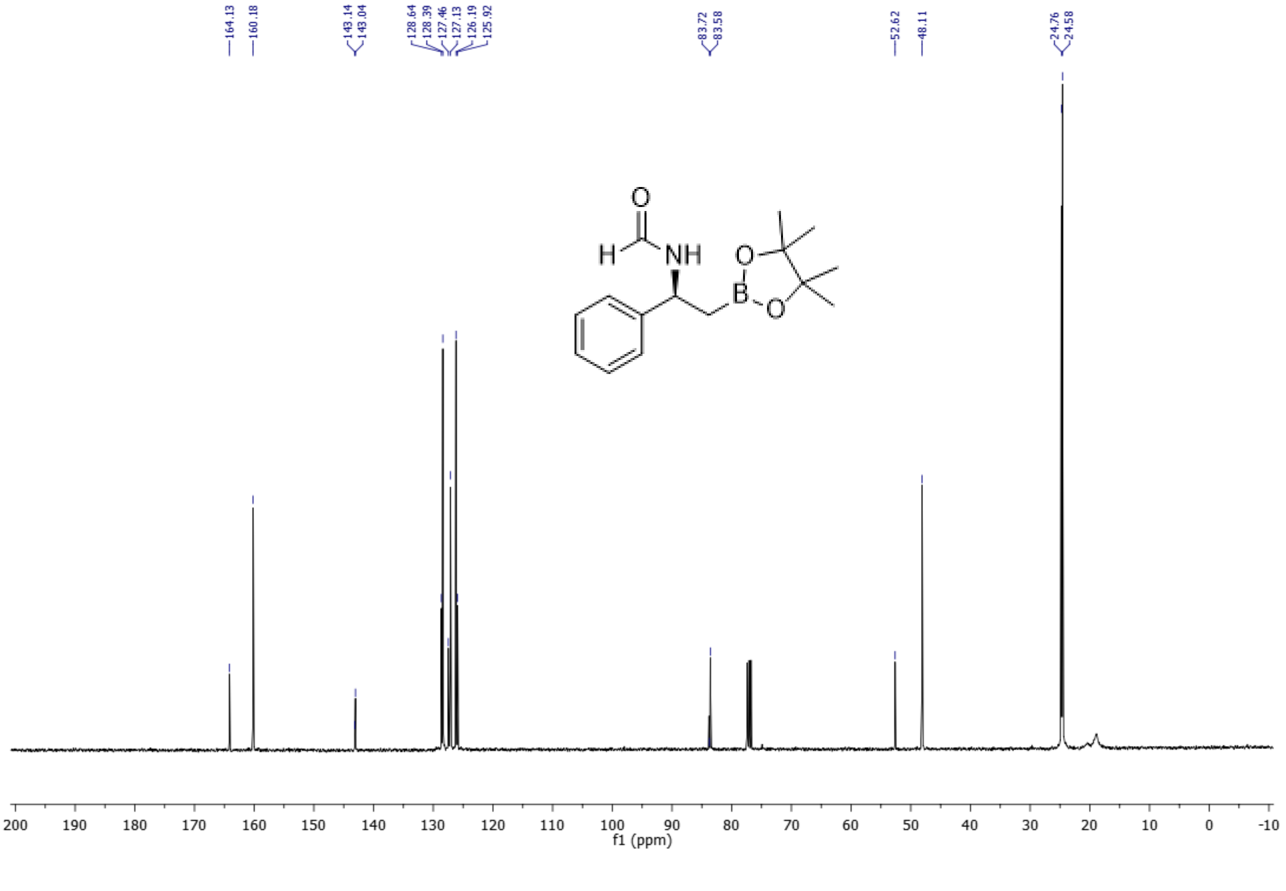

^{11}B NMR (128 MHz, $CDCl_3$) of compound **2a**

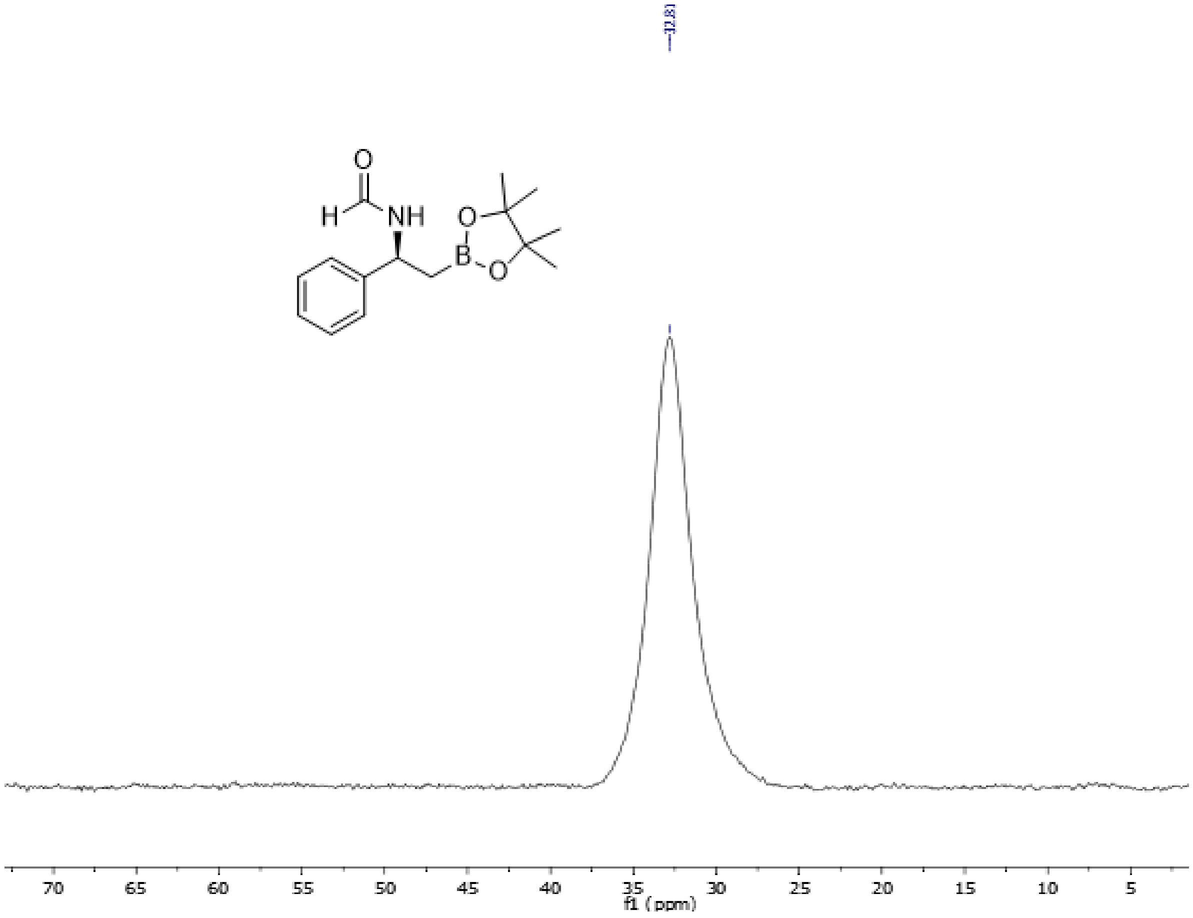

^{1}H NMR (400 MHz, $CDCl_3$, rotamers mixture 60:40) of compound **2b**

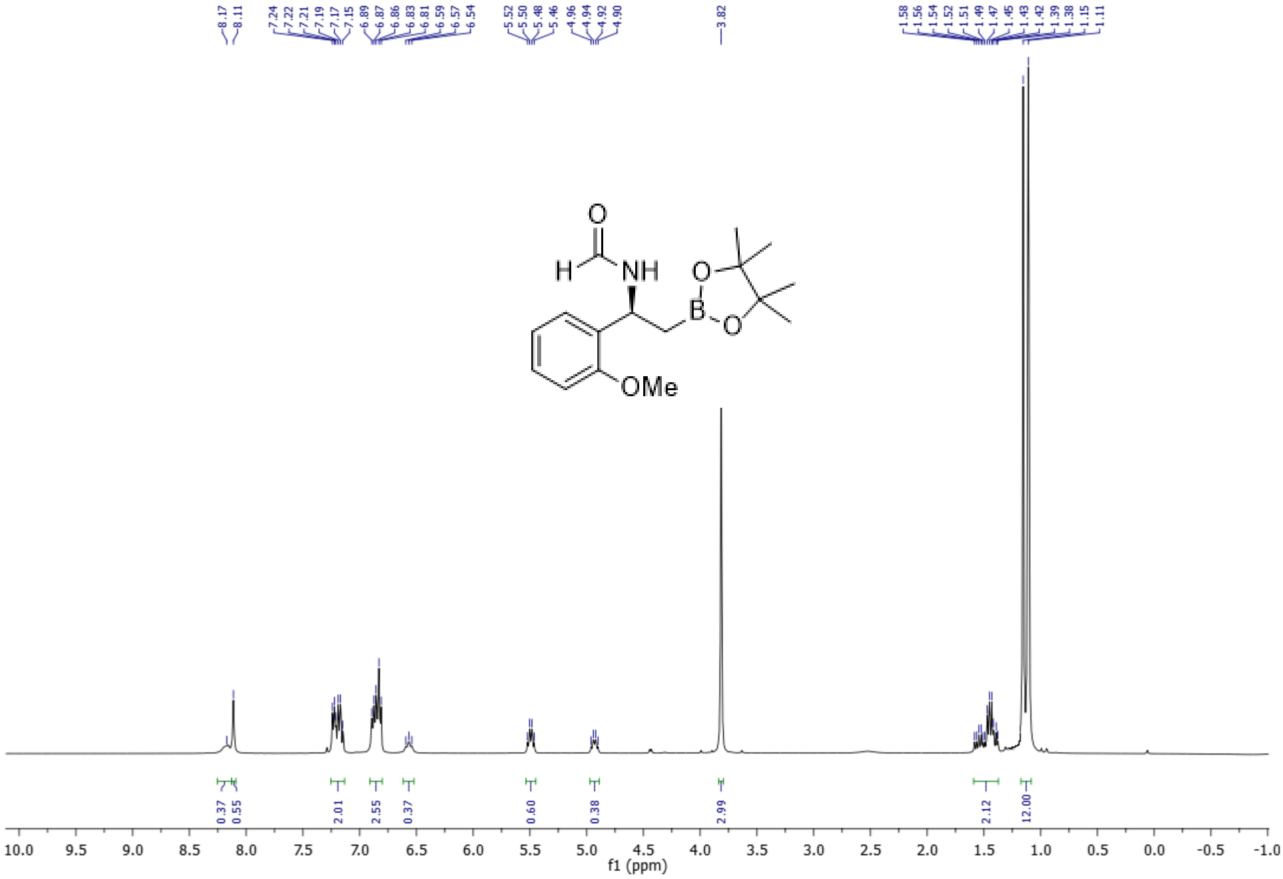

^{13}C NMR (101 MHz, $CDCl_3$, rotamers mixture 60:40) of compound **2b**

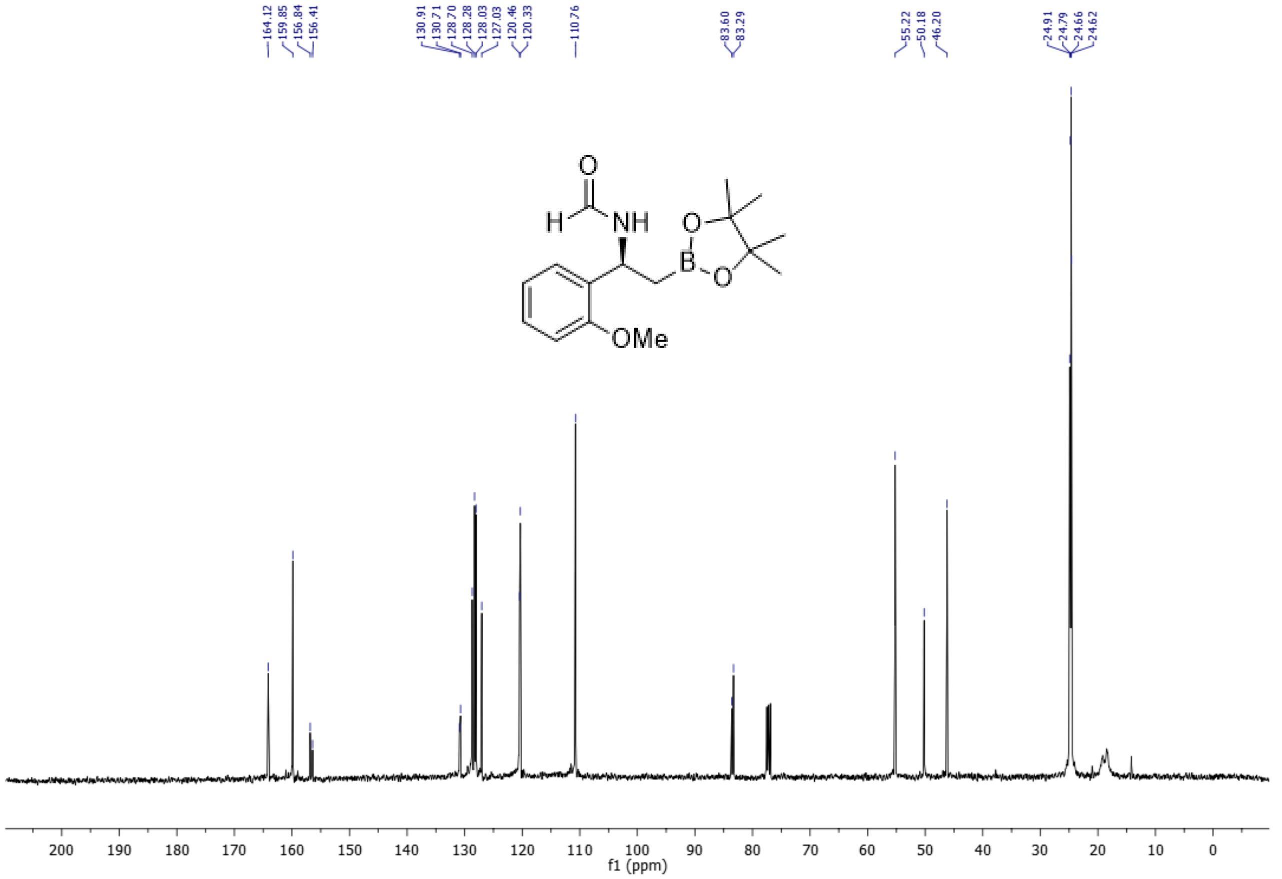

^{11}B NMR (128 MHz, $CDCl_3$) of compound **2b**

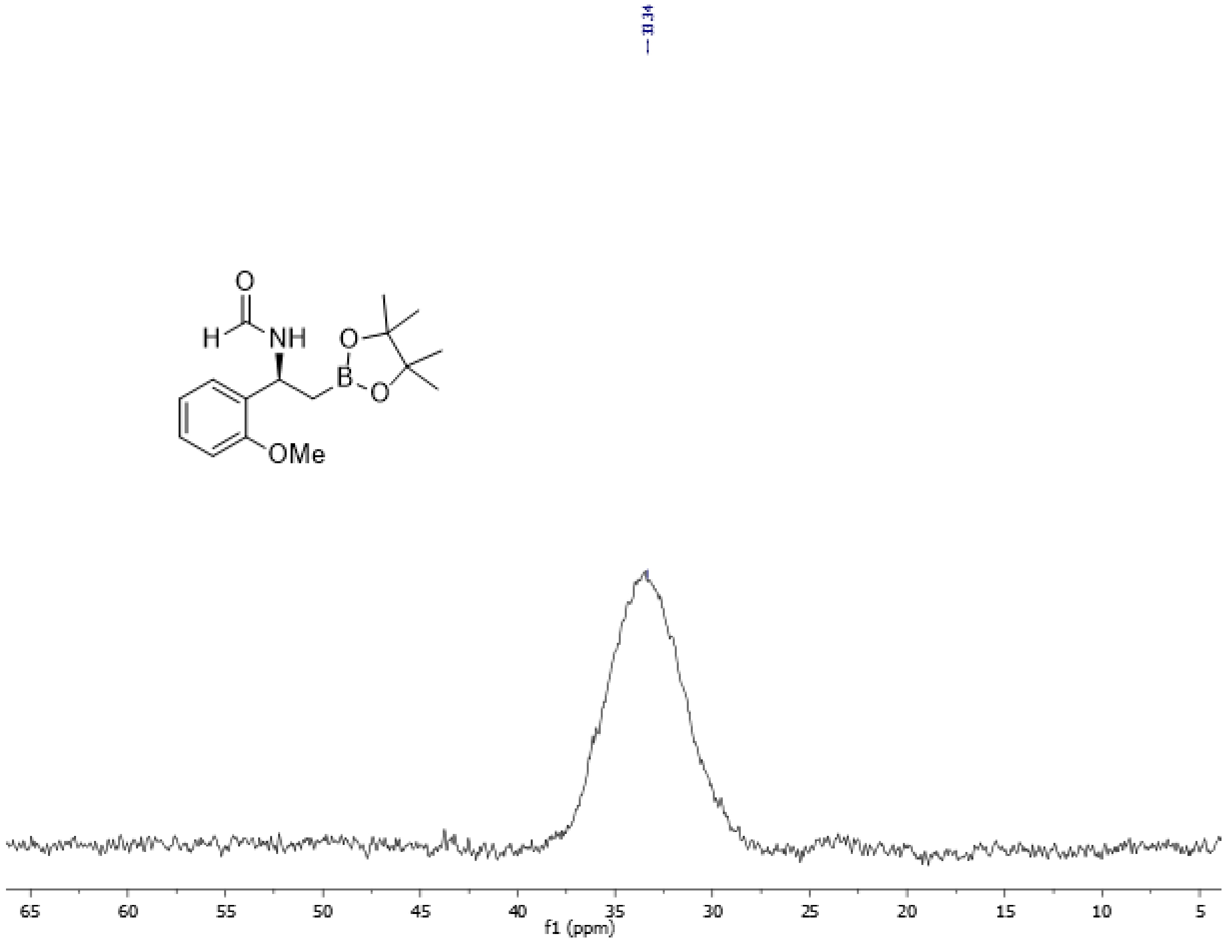

^{1}H NMR (400 MHz, $CDCl_3$) of compound **2c**

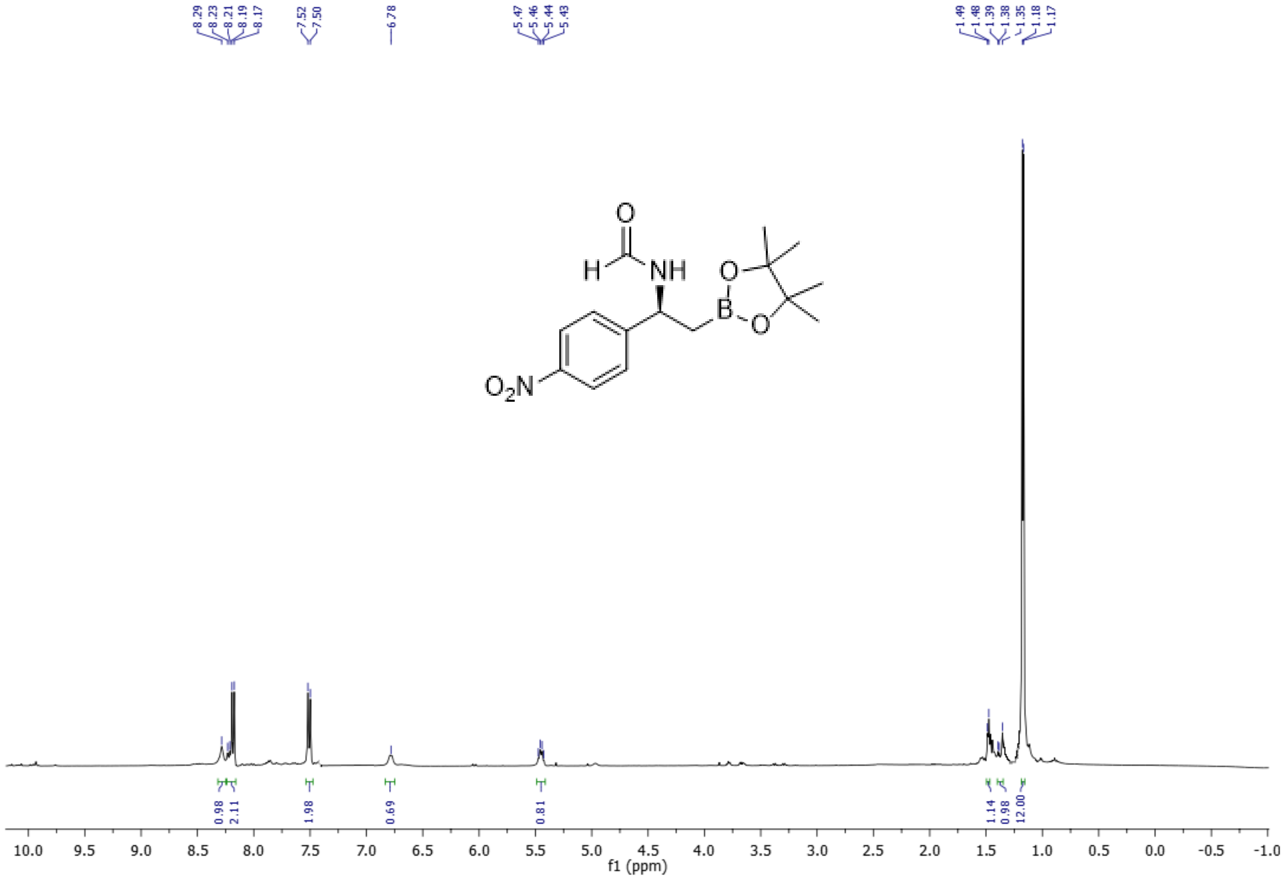

^{13}C NMR (101 MHz, $CDCl_3$) of compound **2c**

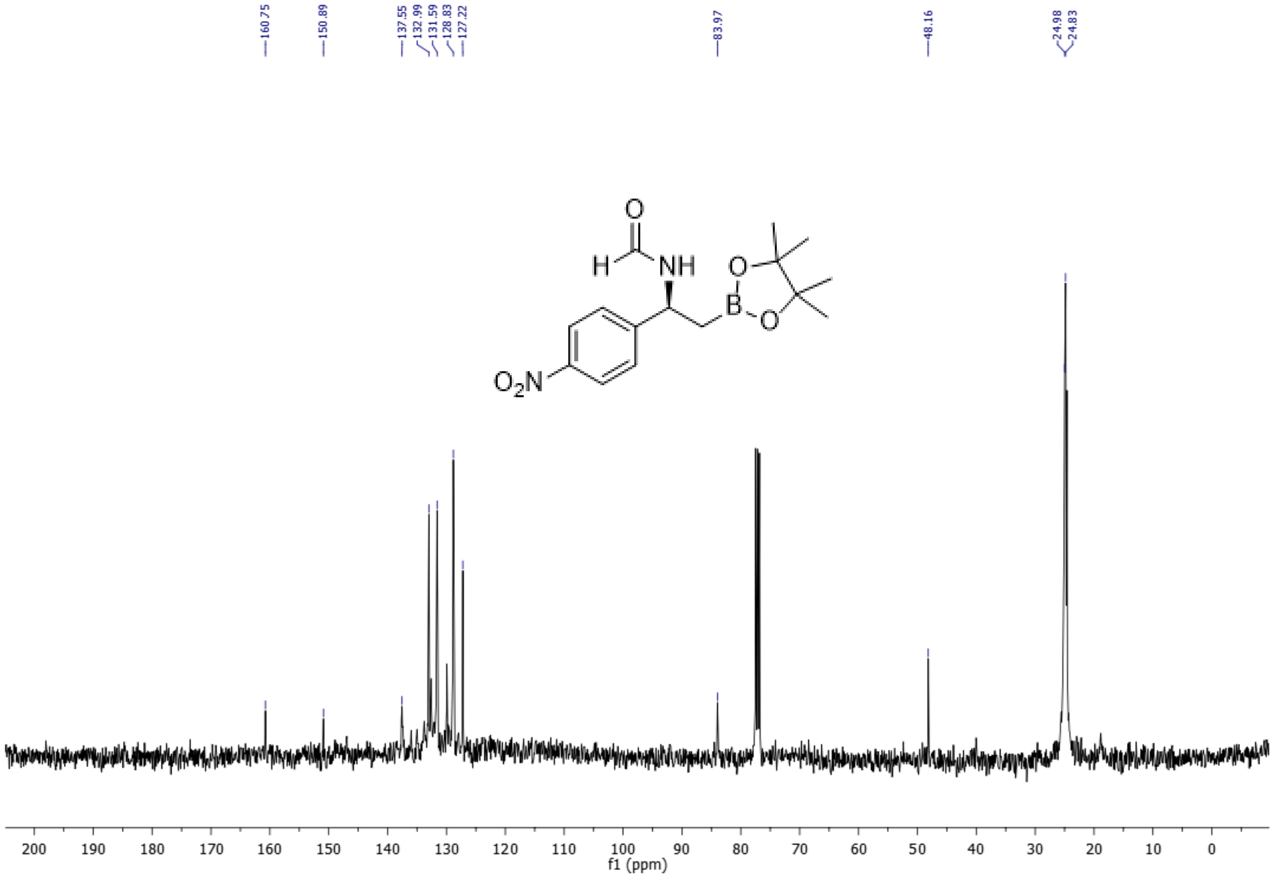

^{11}B NMR (128 MHz, $CDCl_3$) of compound **2c**

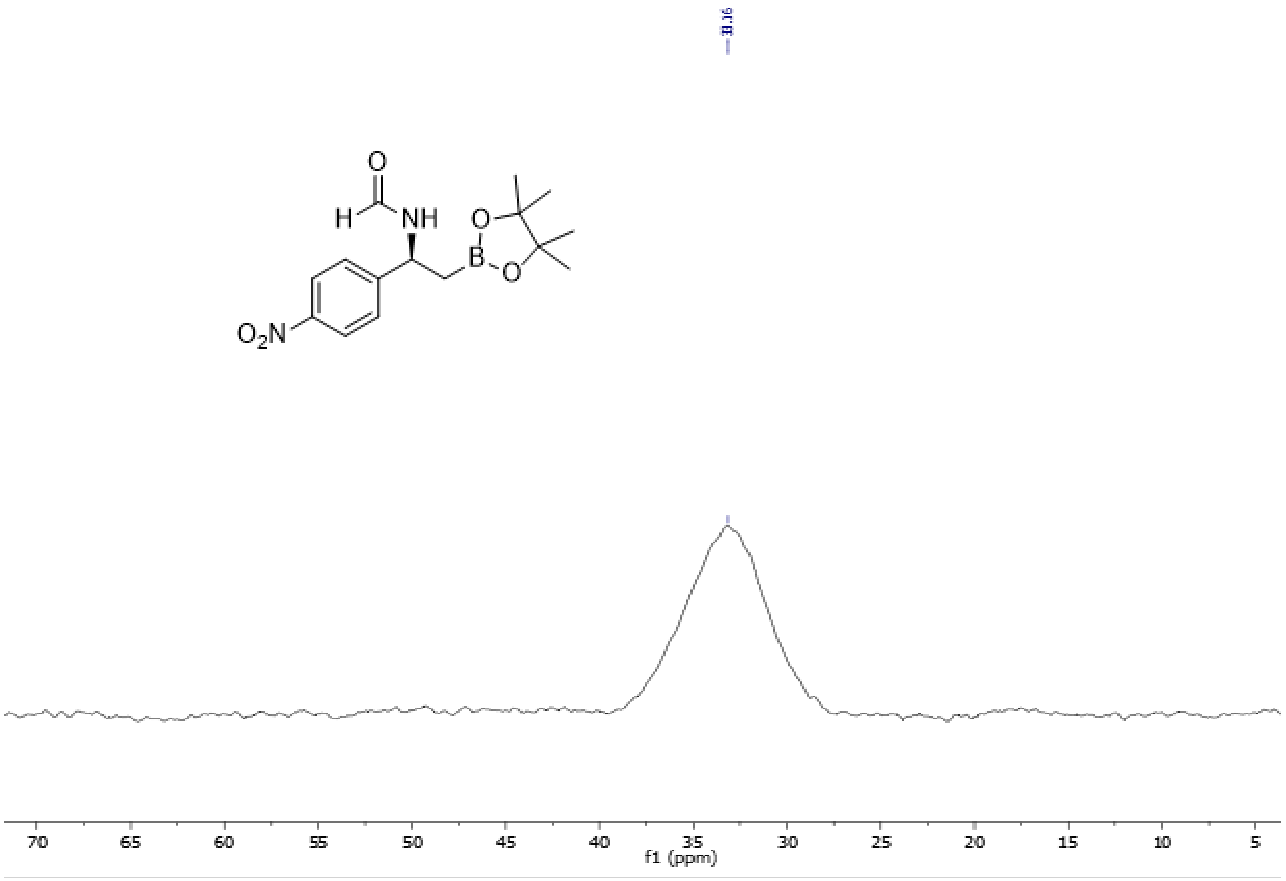

^{1}H NMR (400 MHz, $CDCl_3$. rotamers mixture 80:20) of compound **2d**

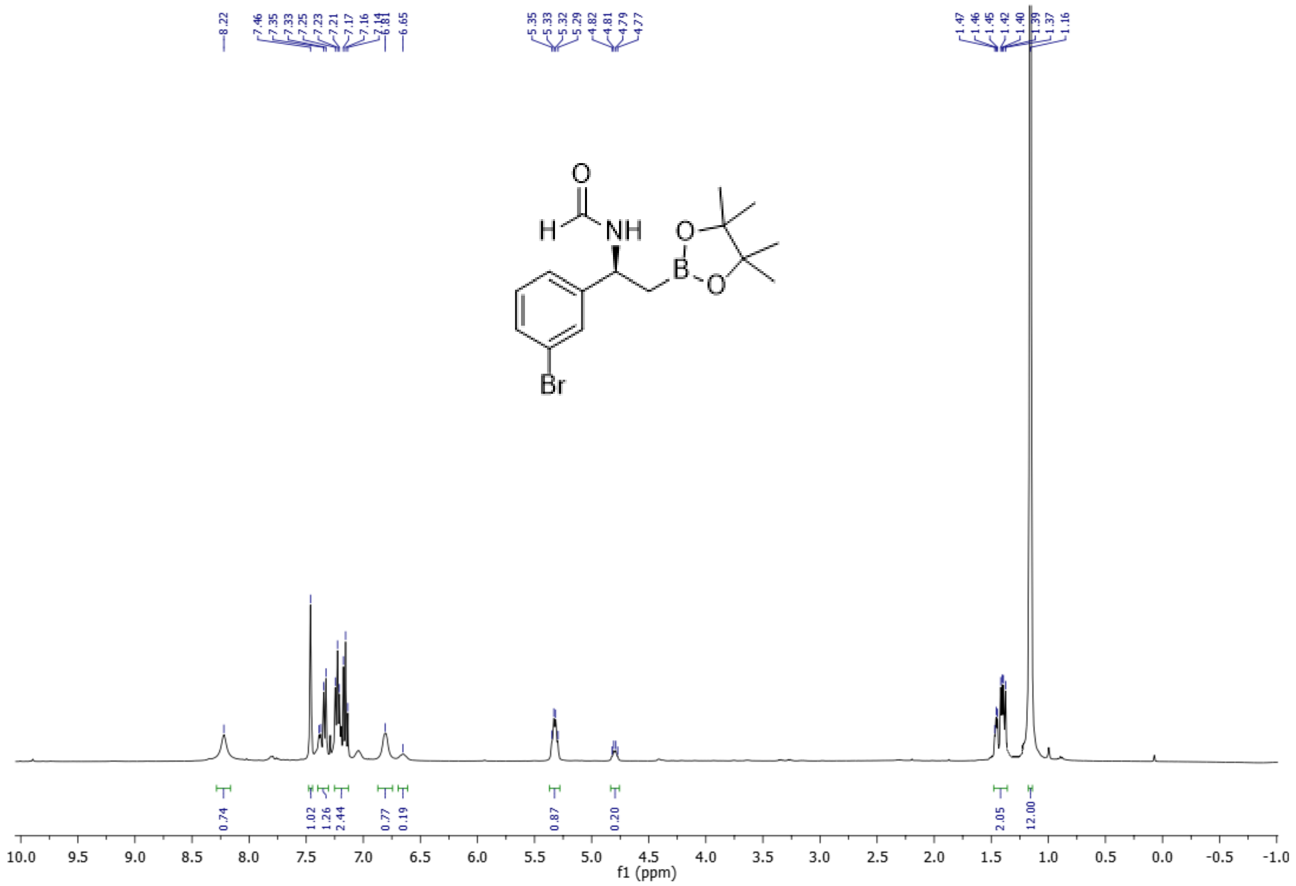

^{13}C NMR (101 MHz, $CDCl_3$, rotamers mixture 80:20) of compound **2d**

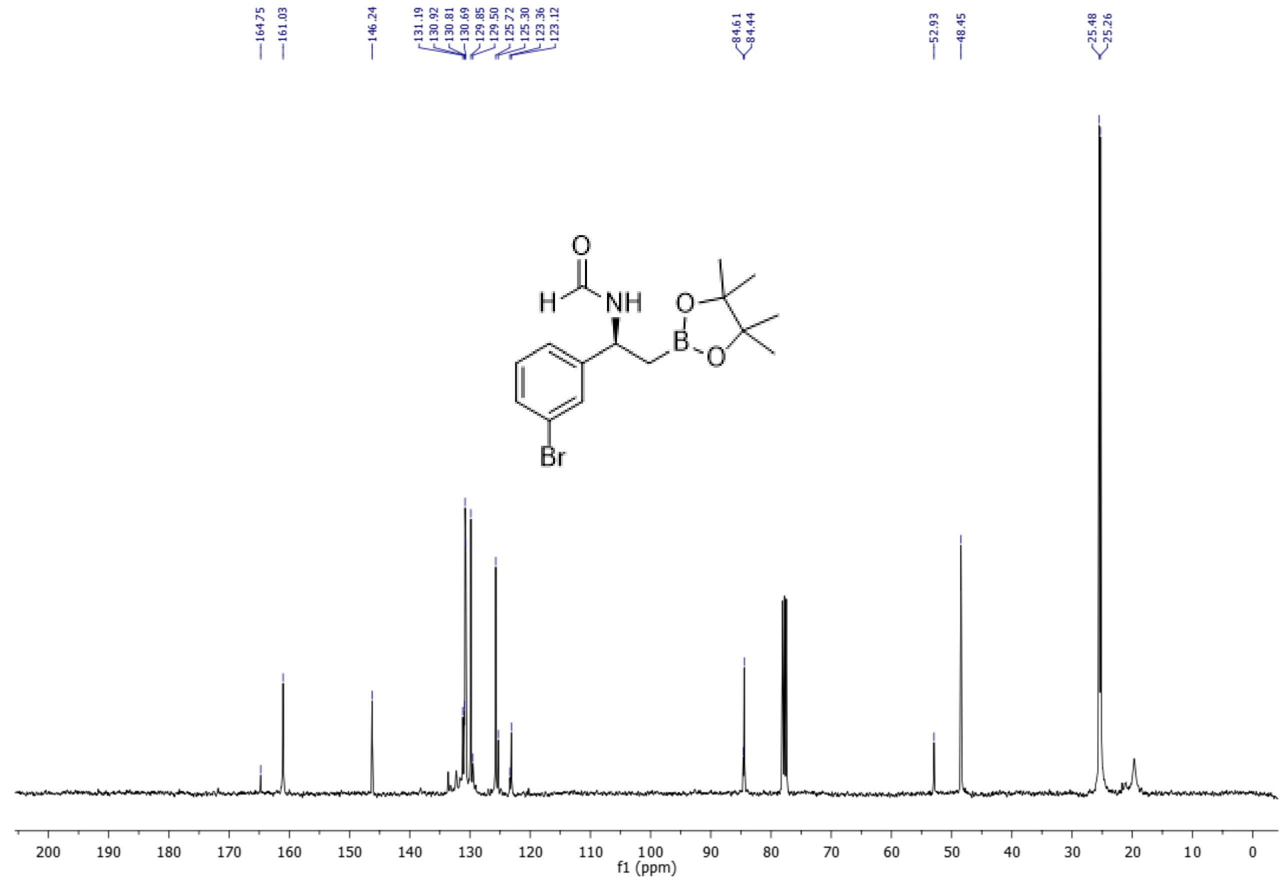

^{11}B NMR (128 MHz, $CDCl_3$) of compound **2d**

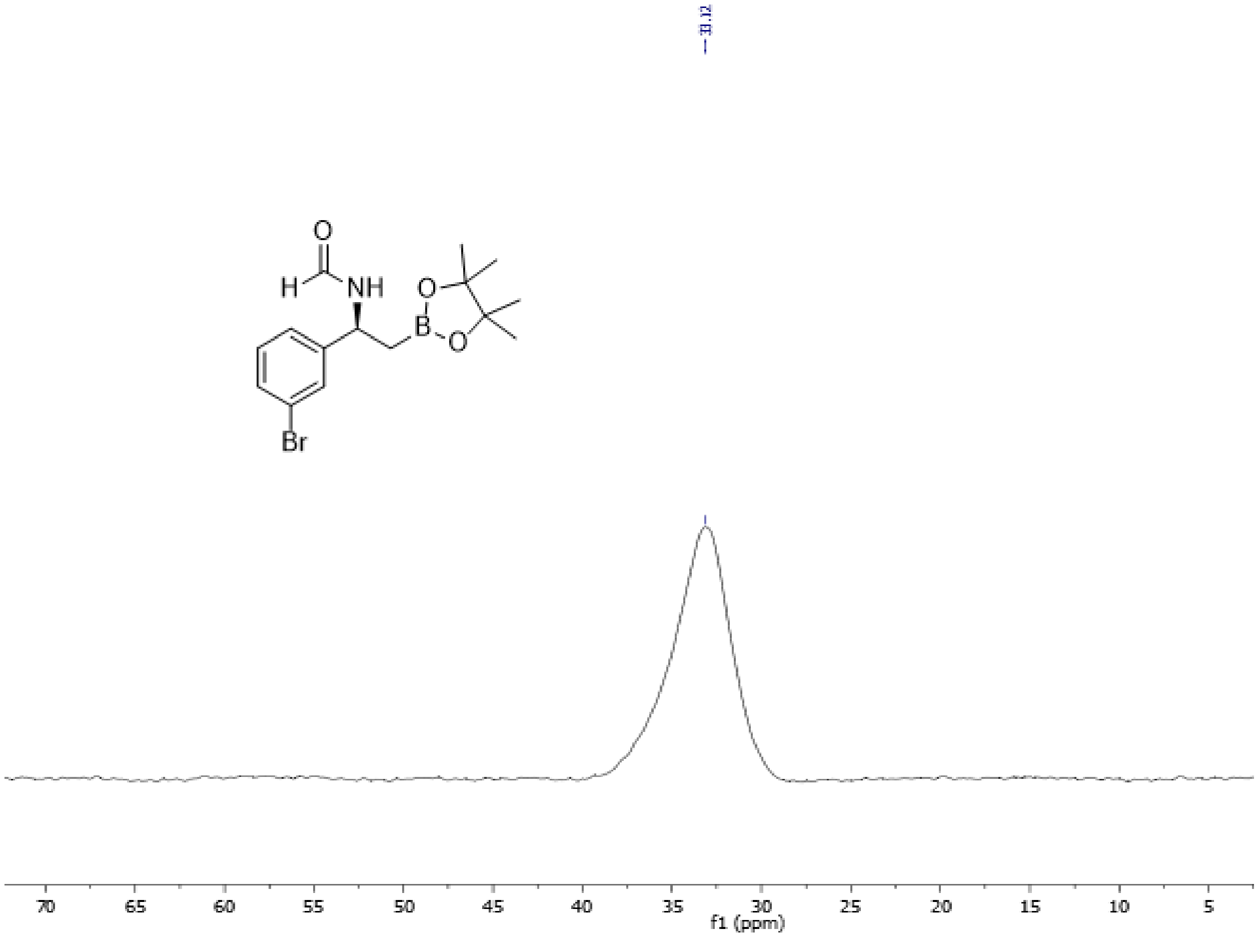

^{1}H NMR (400 MHz, $CDCl_3$, rotamers mixture 80:20) of compound **2e**

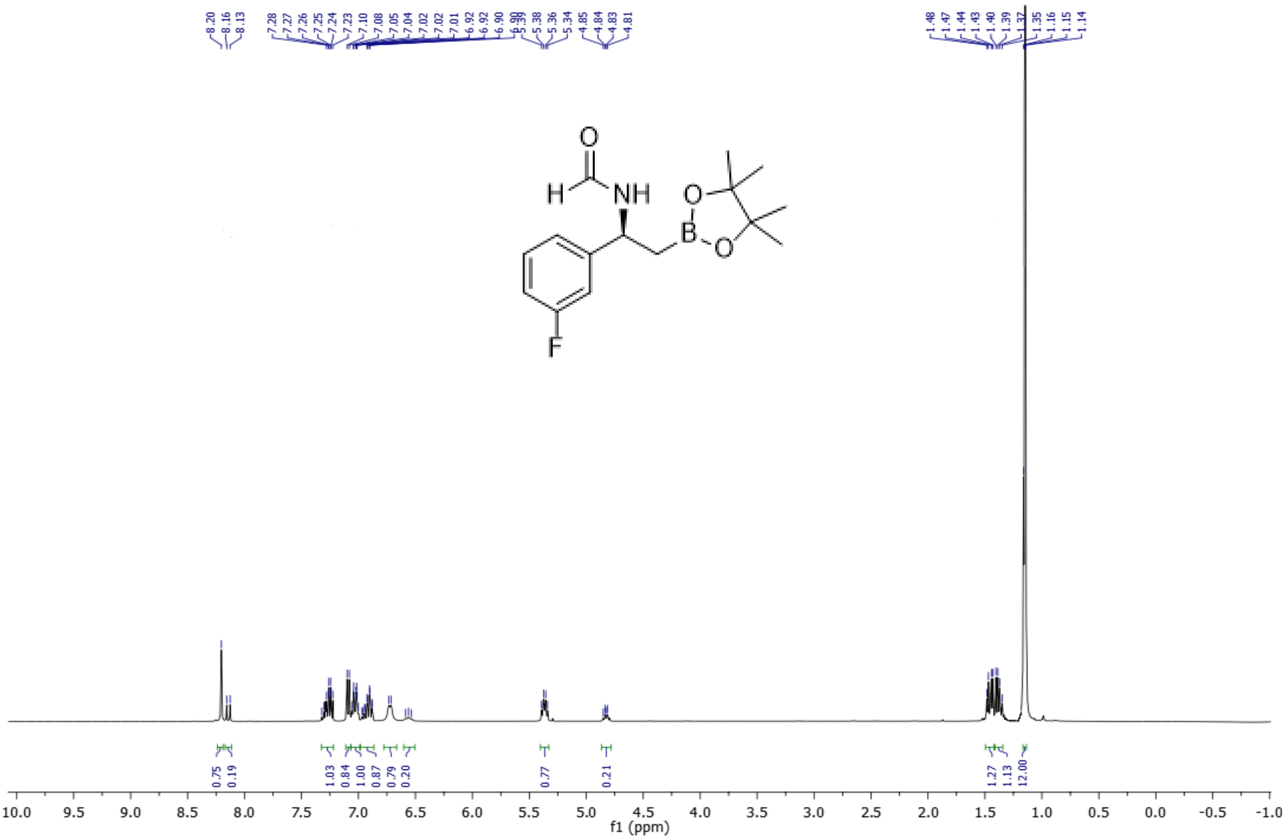

^{13}C NMR (101 MHz, $CDCl_3$, rotamers mixture 80:20) of compound **2e**

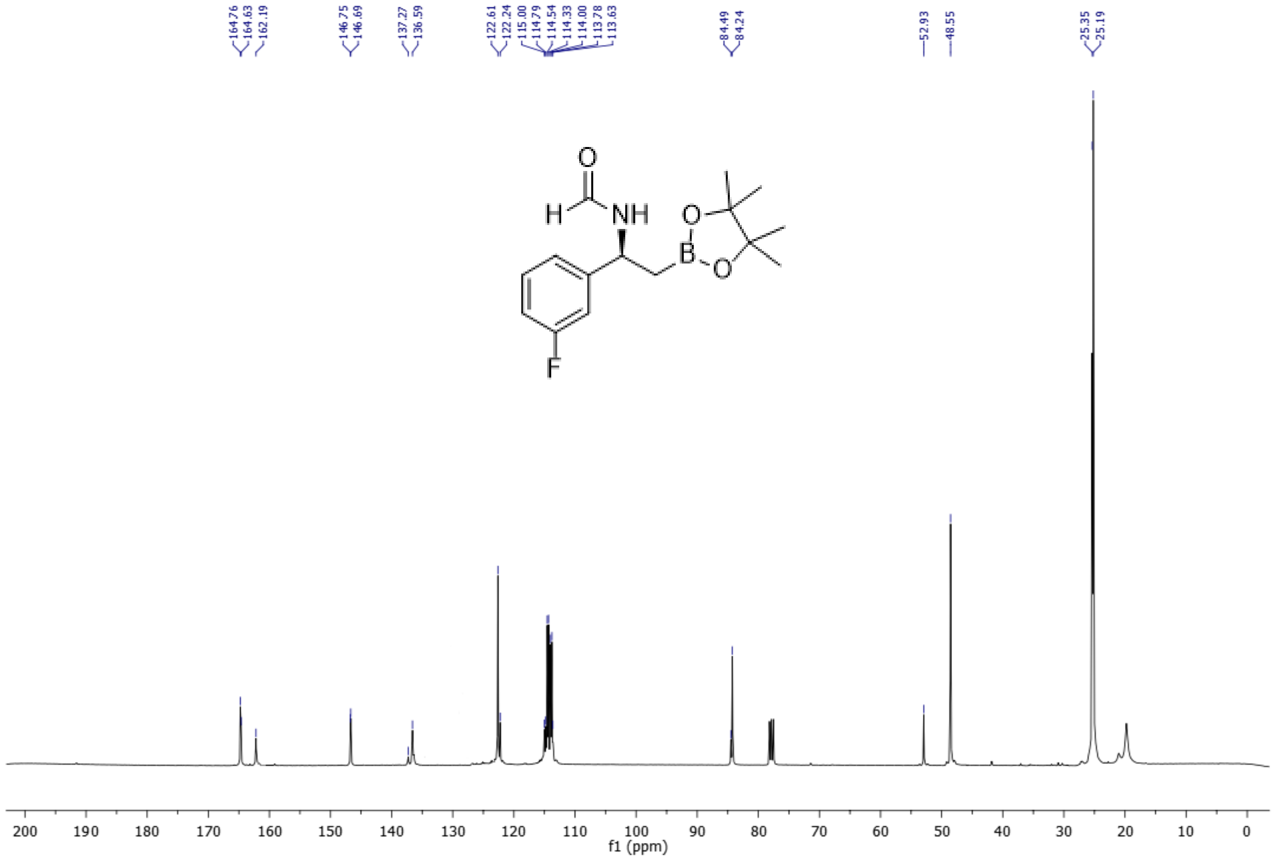

^{11}B NMR (128 MHz, $CDCl_3$) of compound **2e**

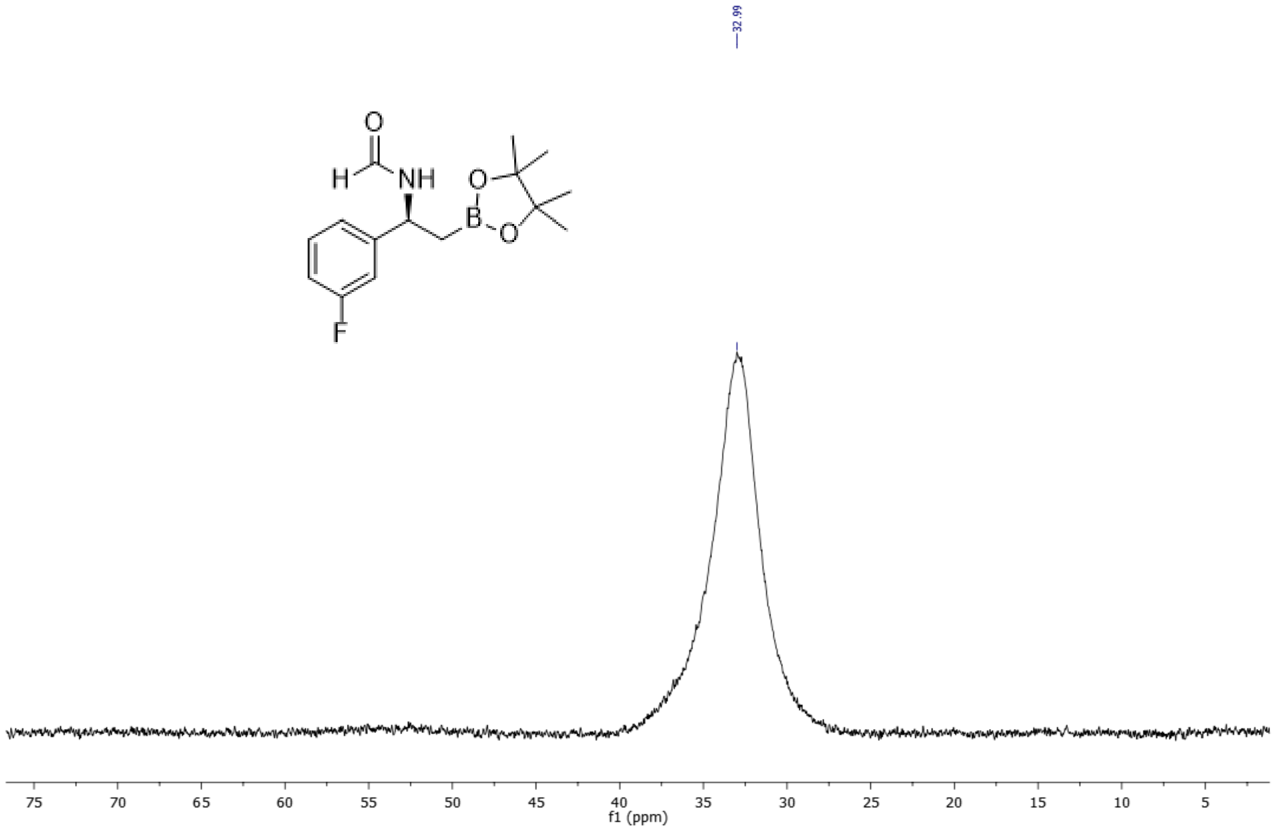

^{1}H NMR (400 MHz, $CDCl_3$, rotamers mixture 75:25) of compound **2f**

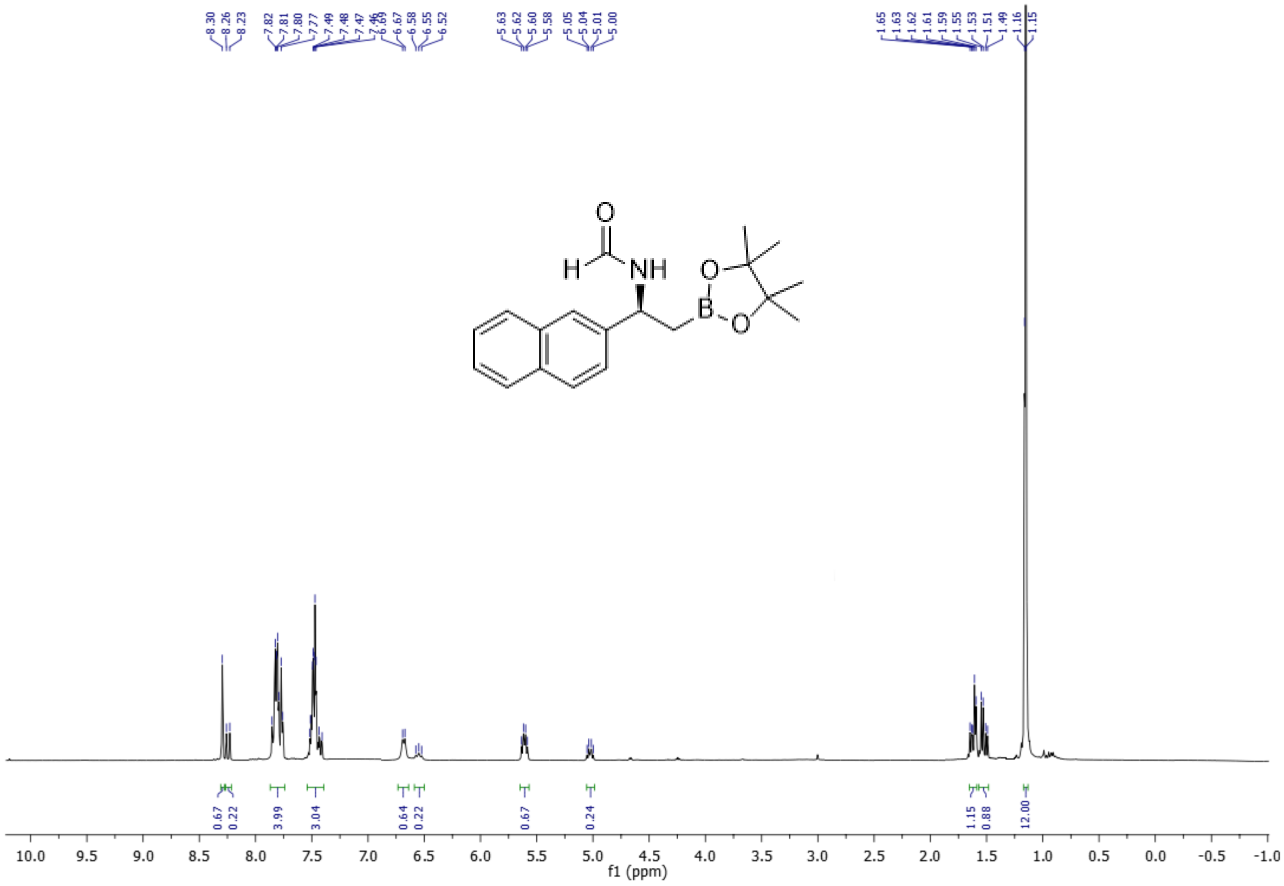

^{13}C NMR (101 MHz, $CDCl_3$, rotamers mixture 75:25) of compound **2f**

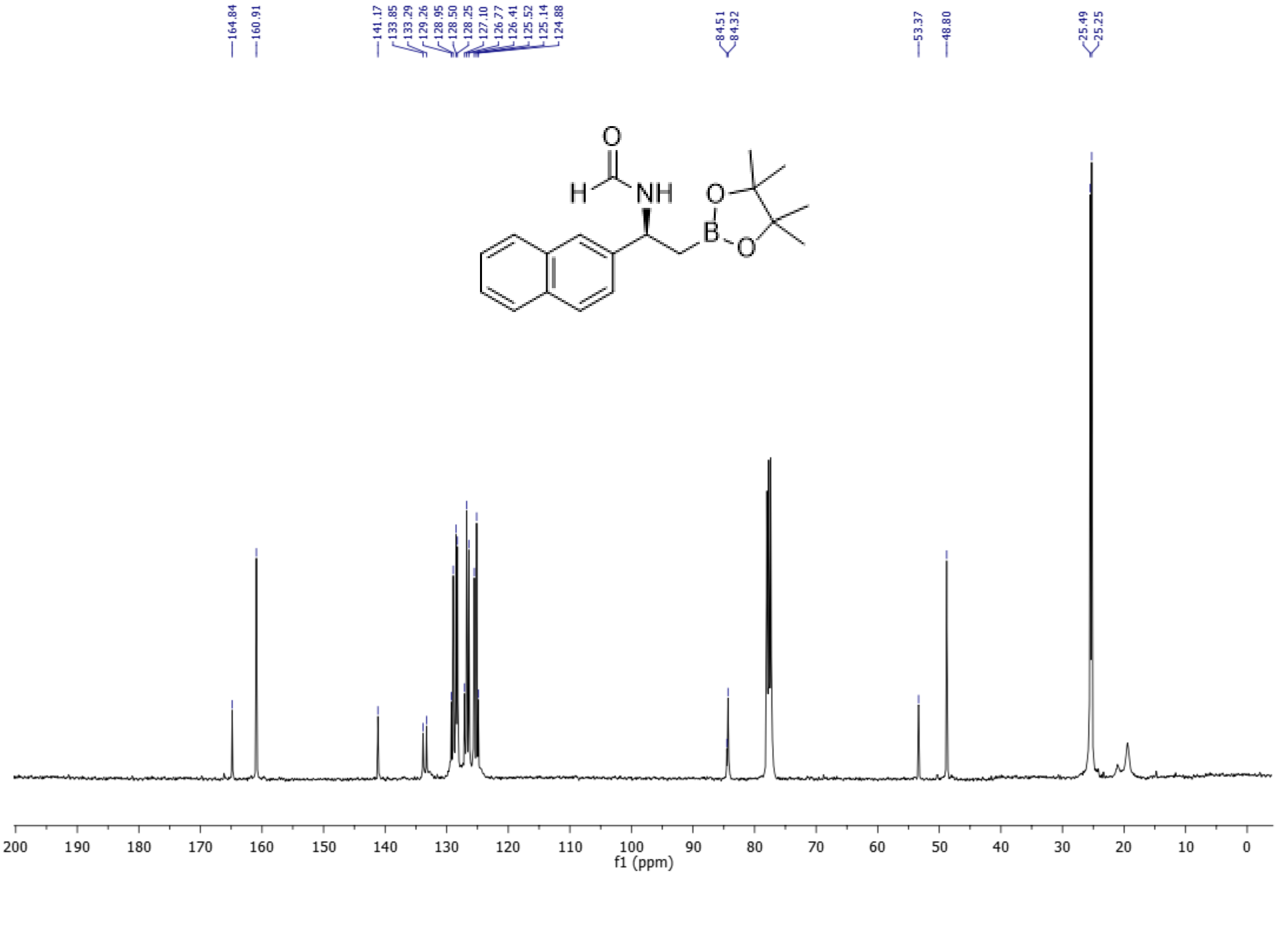

^{11}B NMR (128 MHz, $CDCl_3$) of compound **2f**

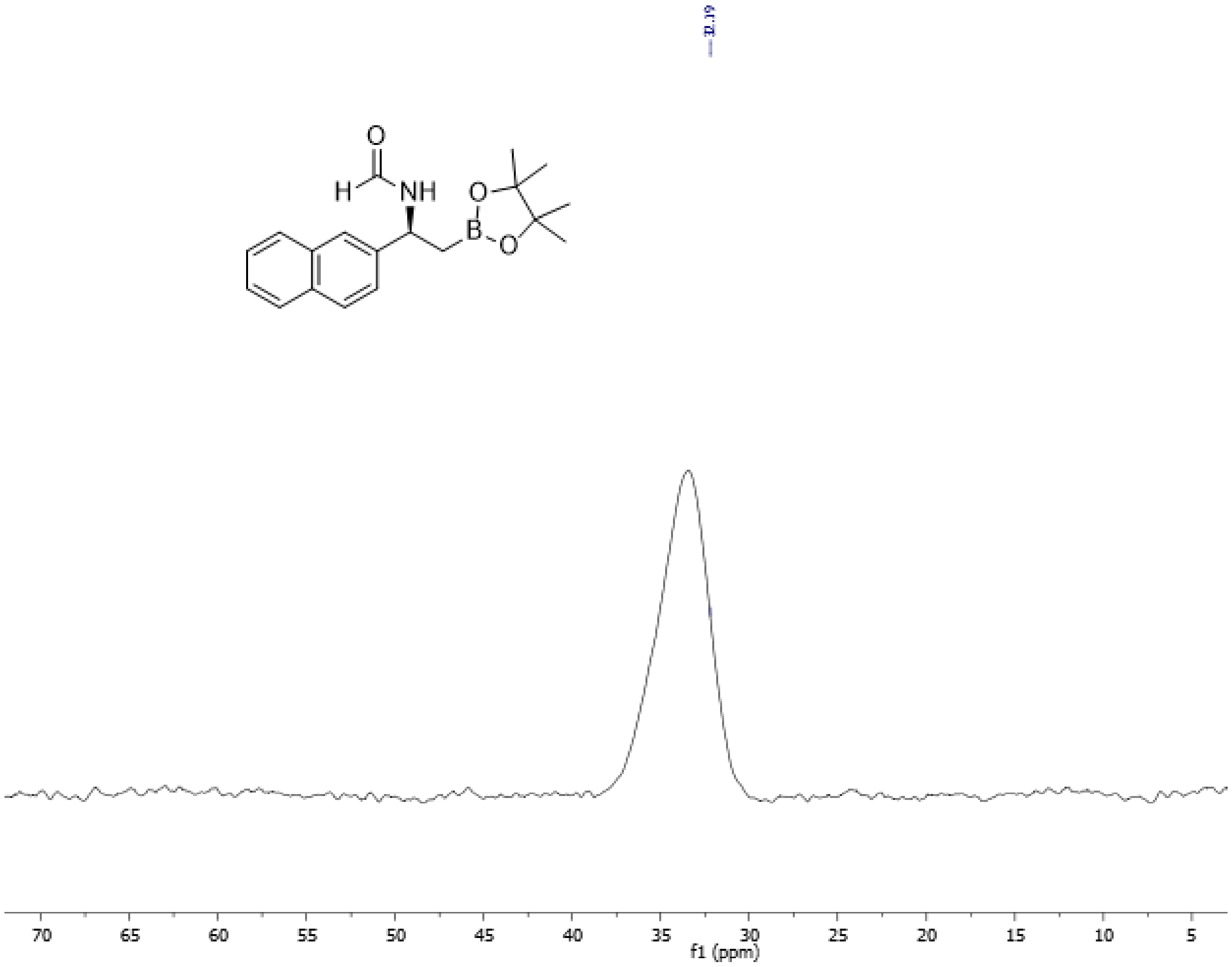

^{1}H NMR (400 MHz, $CDCl_3$, rotamers mixture 75:25) of compound **2g**

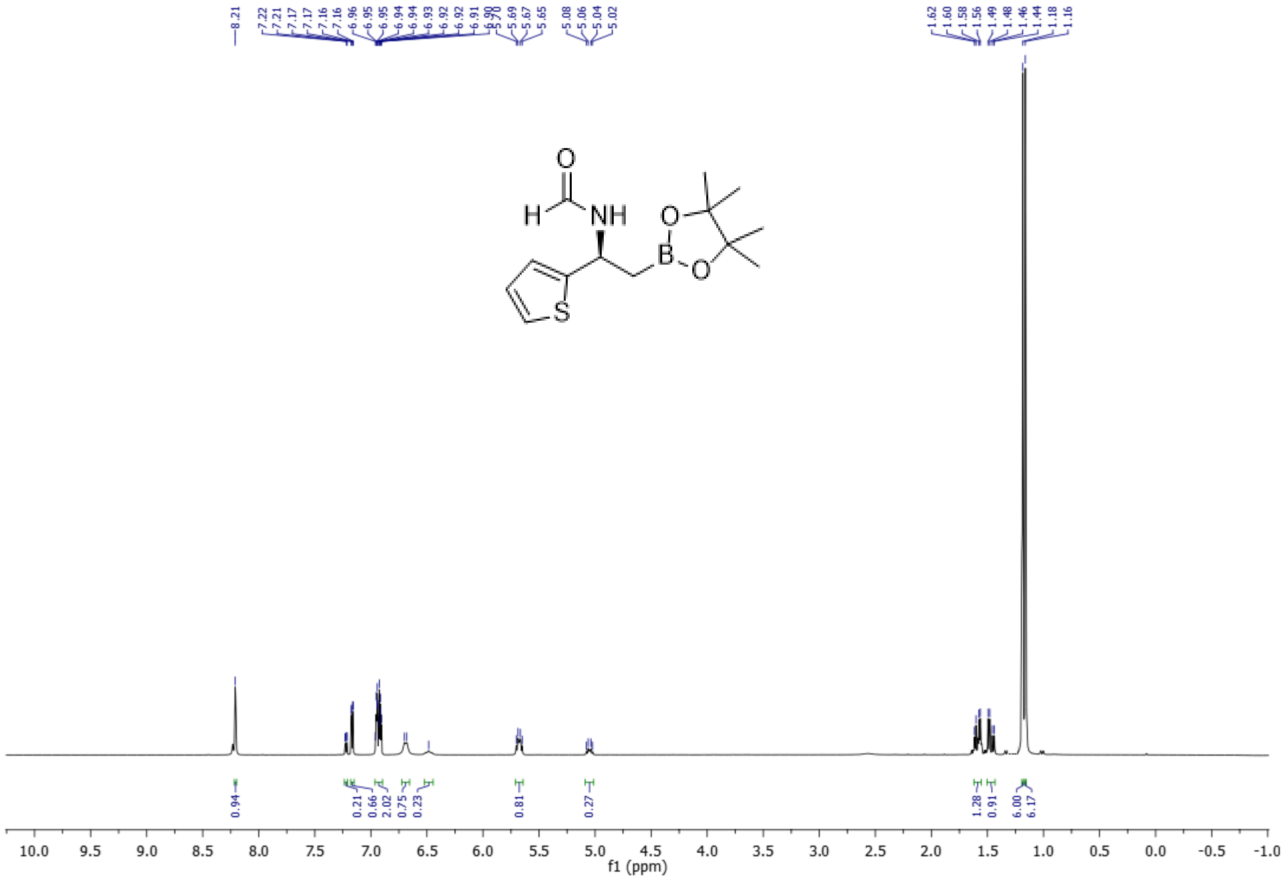

^{13}C NMR (101 MHz, $CDCl_3$, rotamers mixture 75:25) of compound **2g**

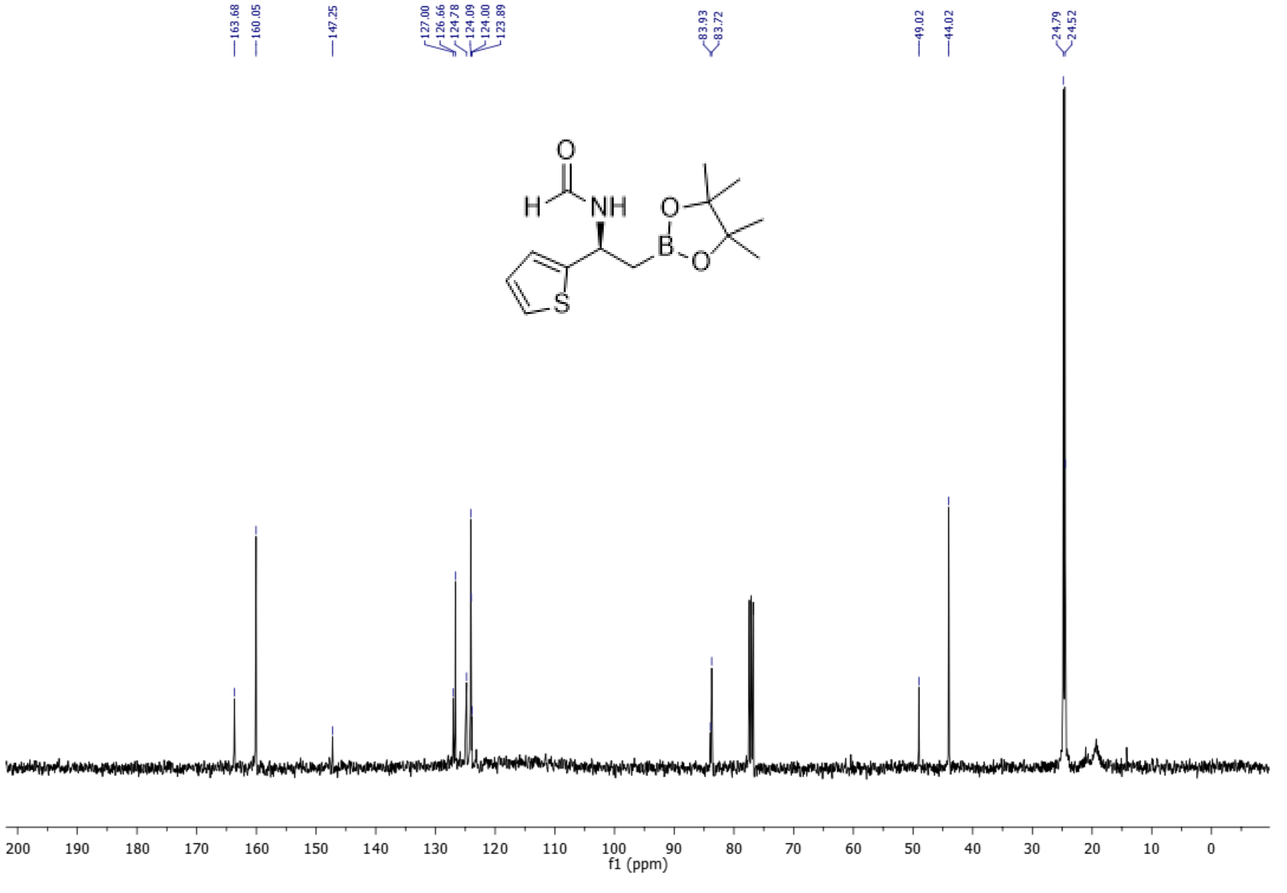

^{11}B NMR (128 MHz, $CDCl_3$) of compound **2g**

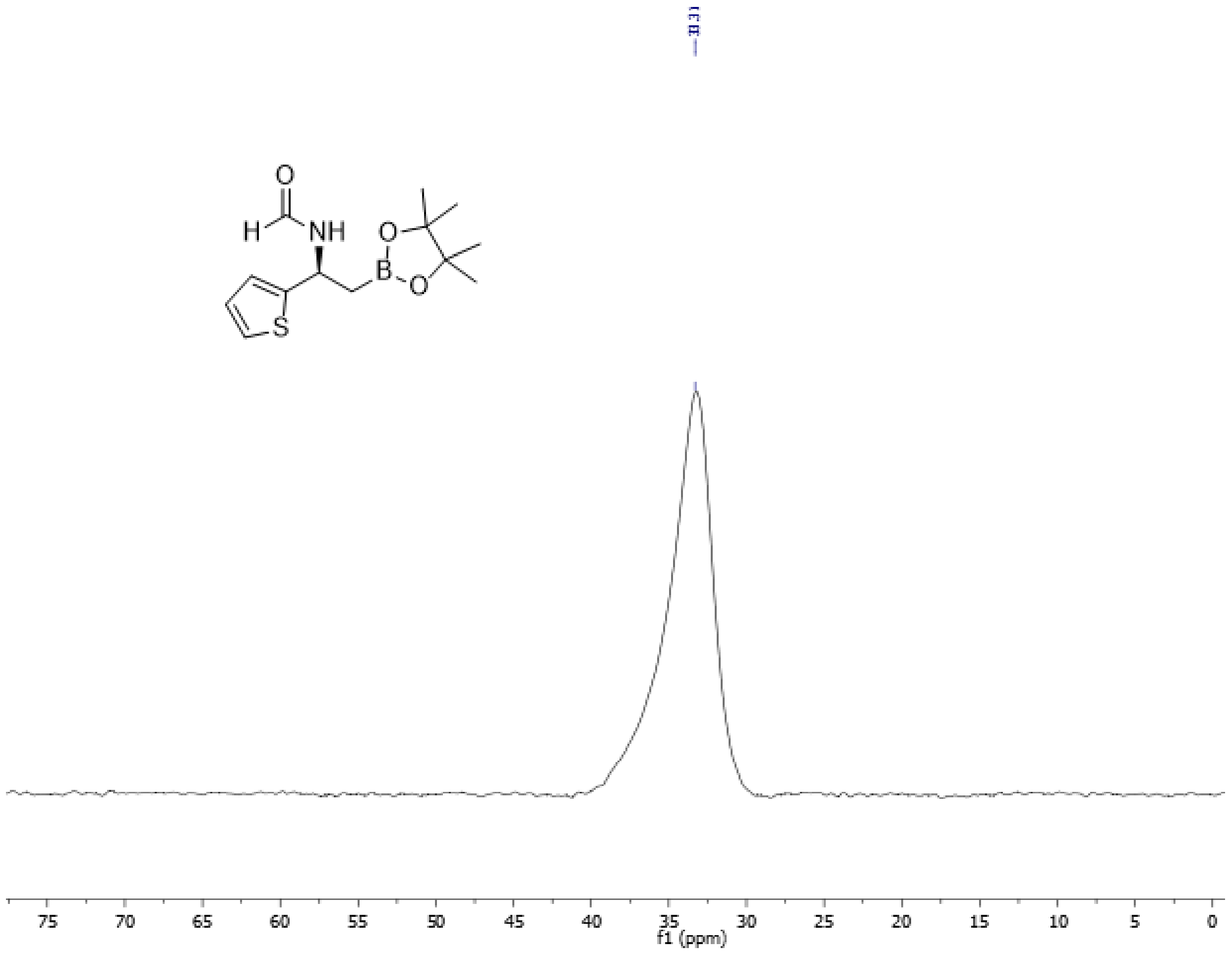

^{1}H (400 MHz, $CDCl_3$, rotamers mixture 70:30) of compound **2h**

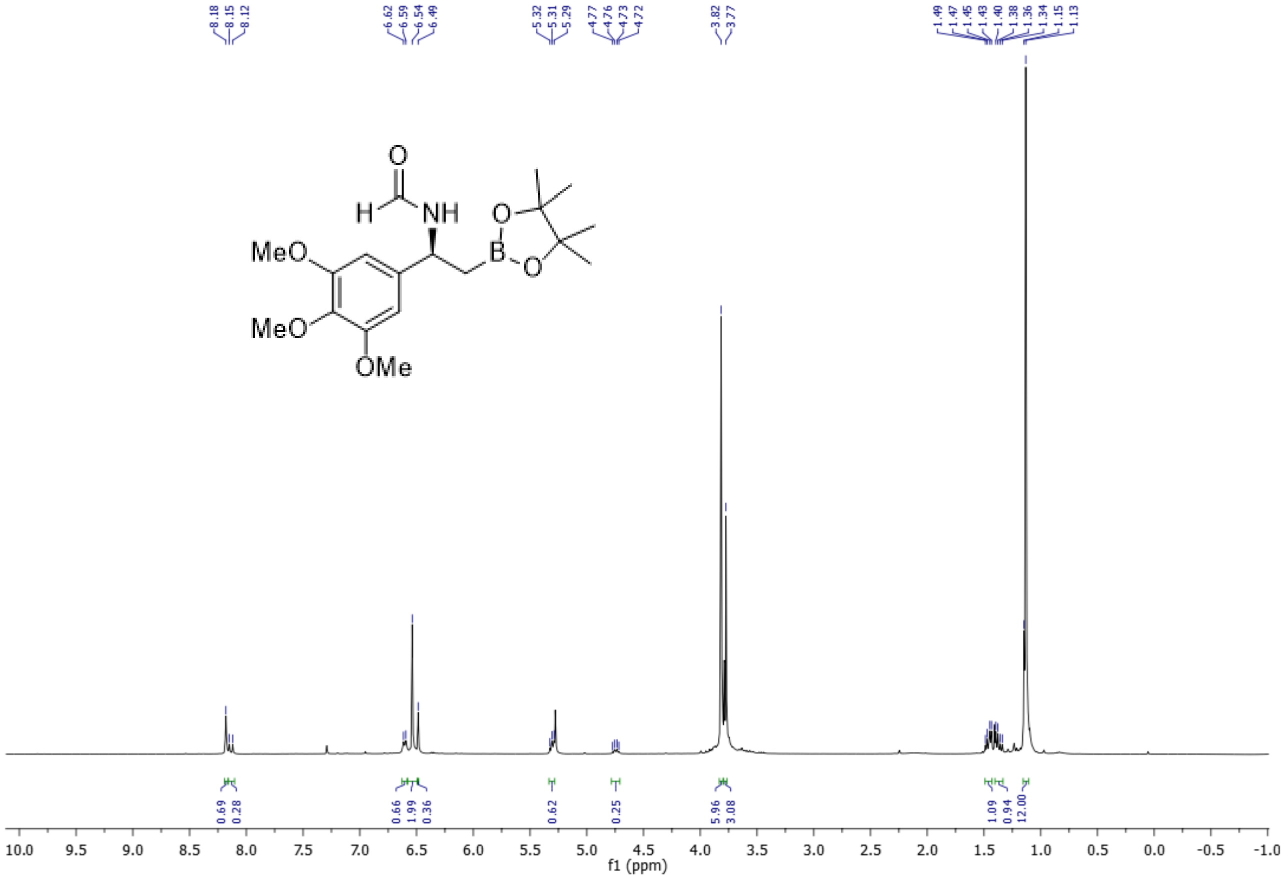

^{13}C NMR (101 MHz, $CDCl_3$, rotamers mixture 70:30) of compound **2h**

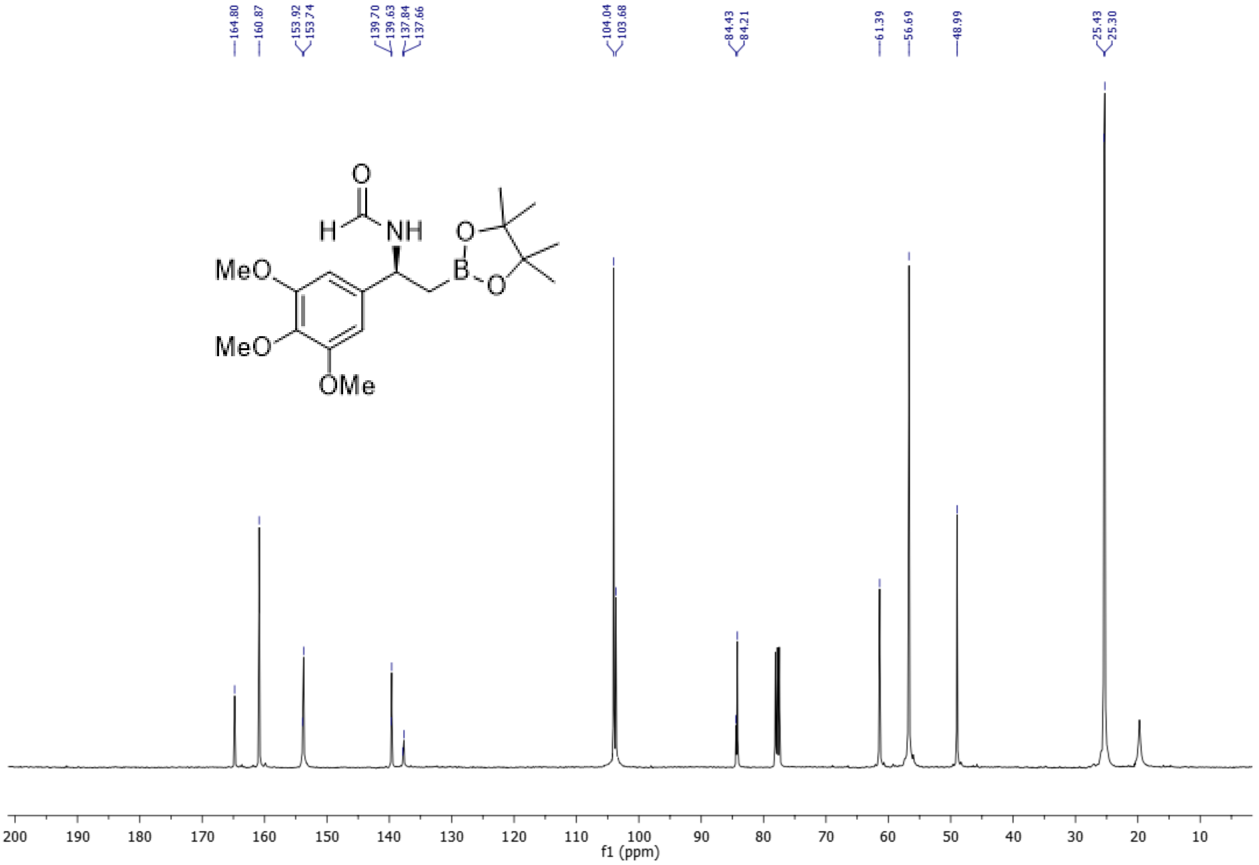

^{11}B NMR (128 MHz, $CDCl_3$) of compound **2h**

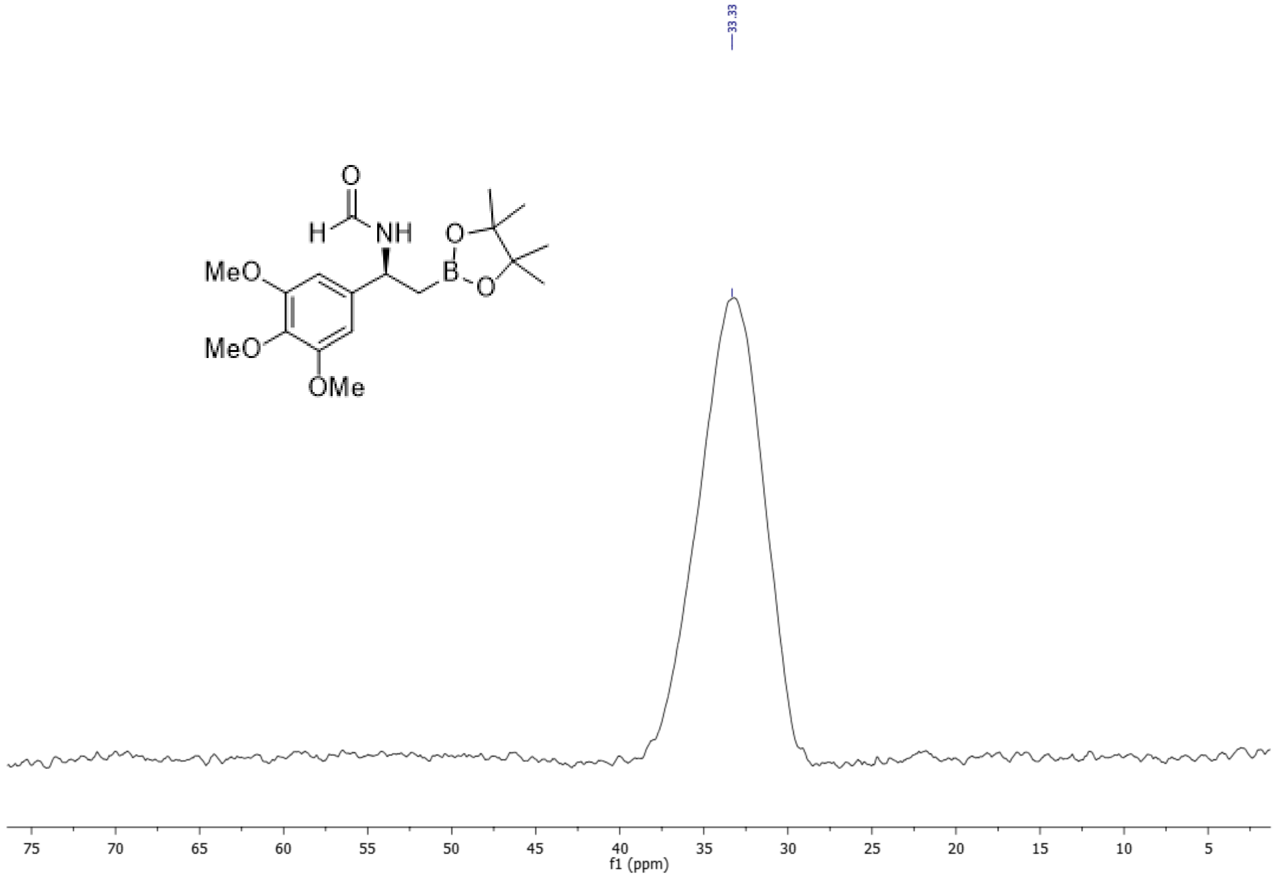

^{1}H NMR (400 MHz, $CDCl_3$) of compound **2i**

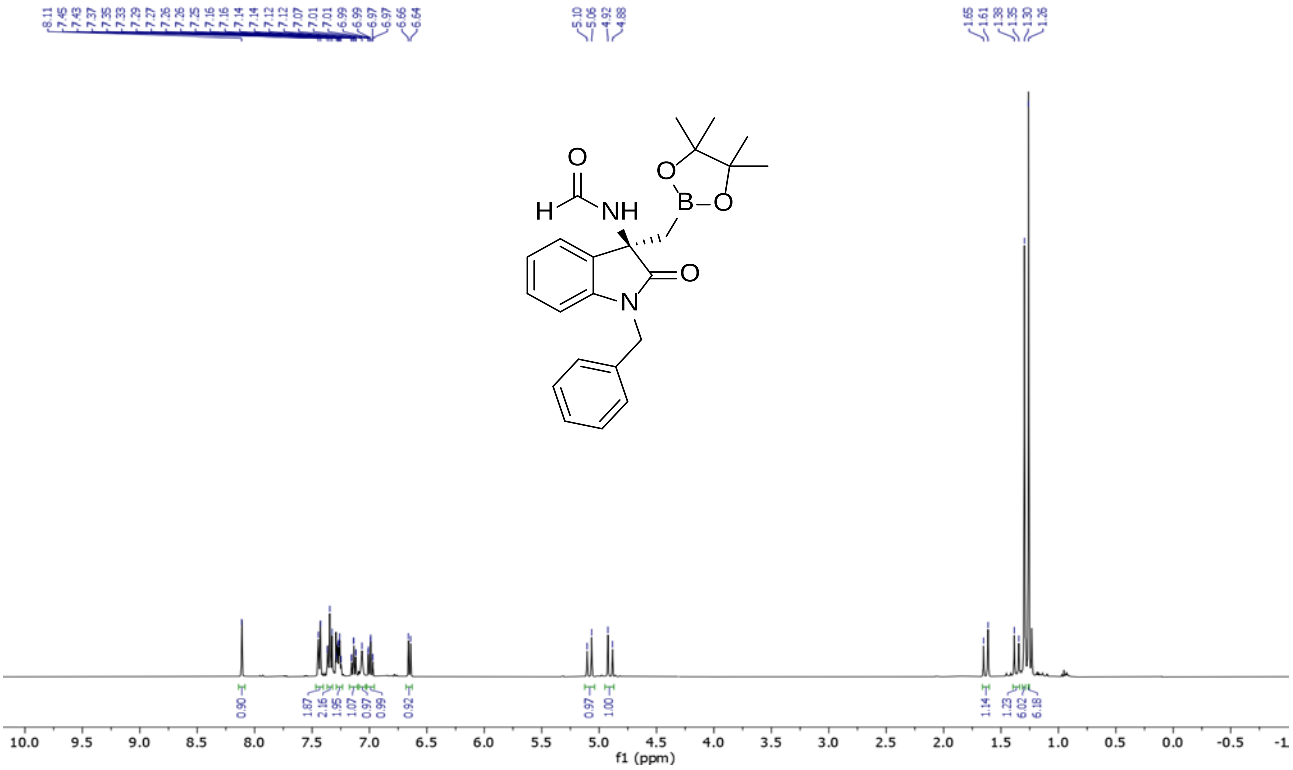

^{13}C NMR (101 MHz, $CDCl_3$) of compound **2i**

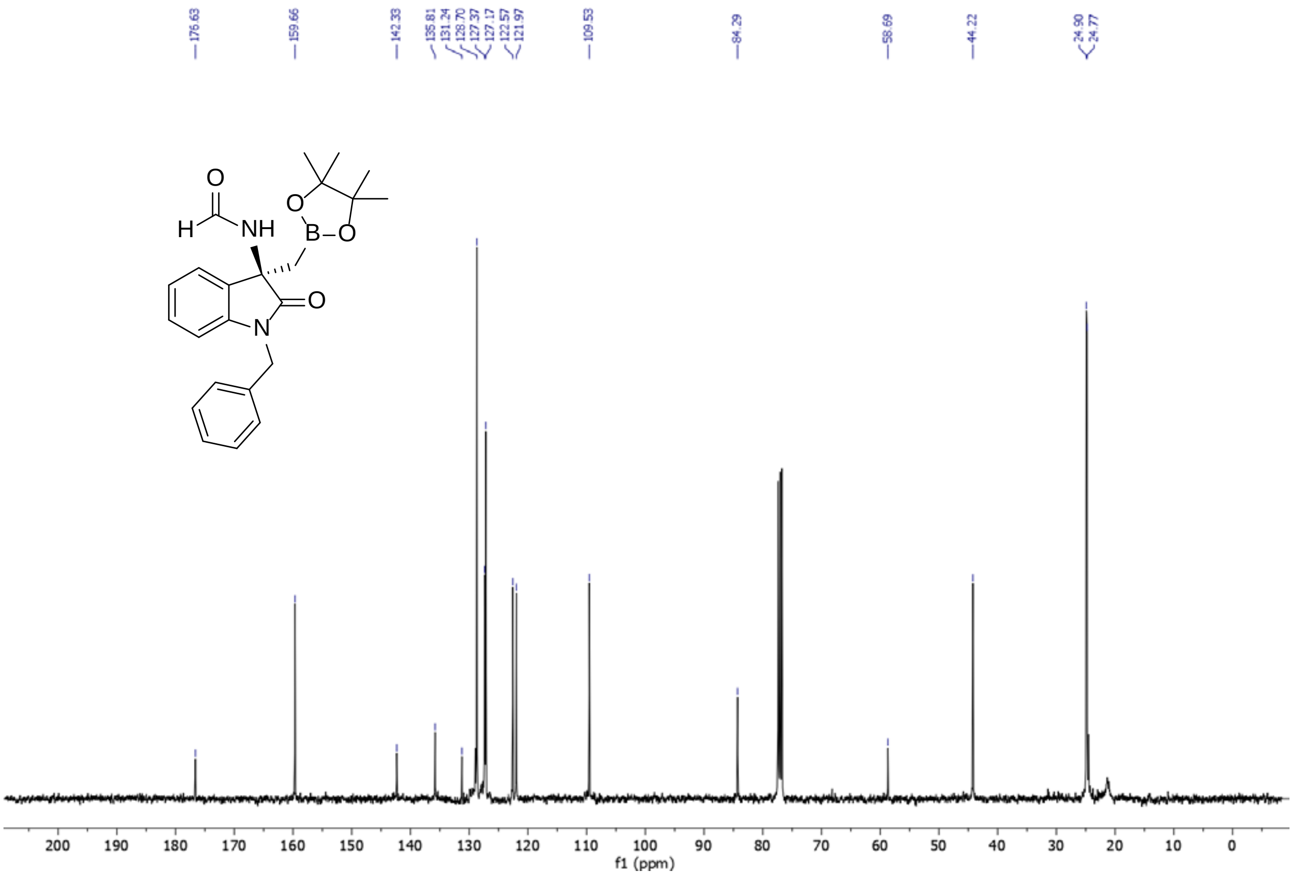

^{11}B NMR (128 MHz, $CDCl_3$) of compound **2i**

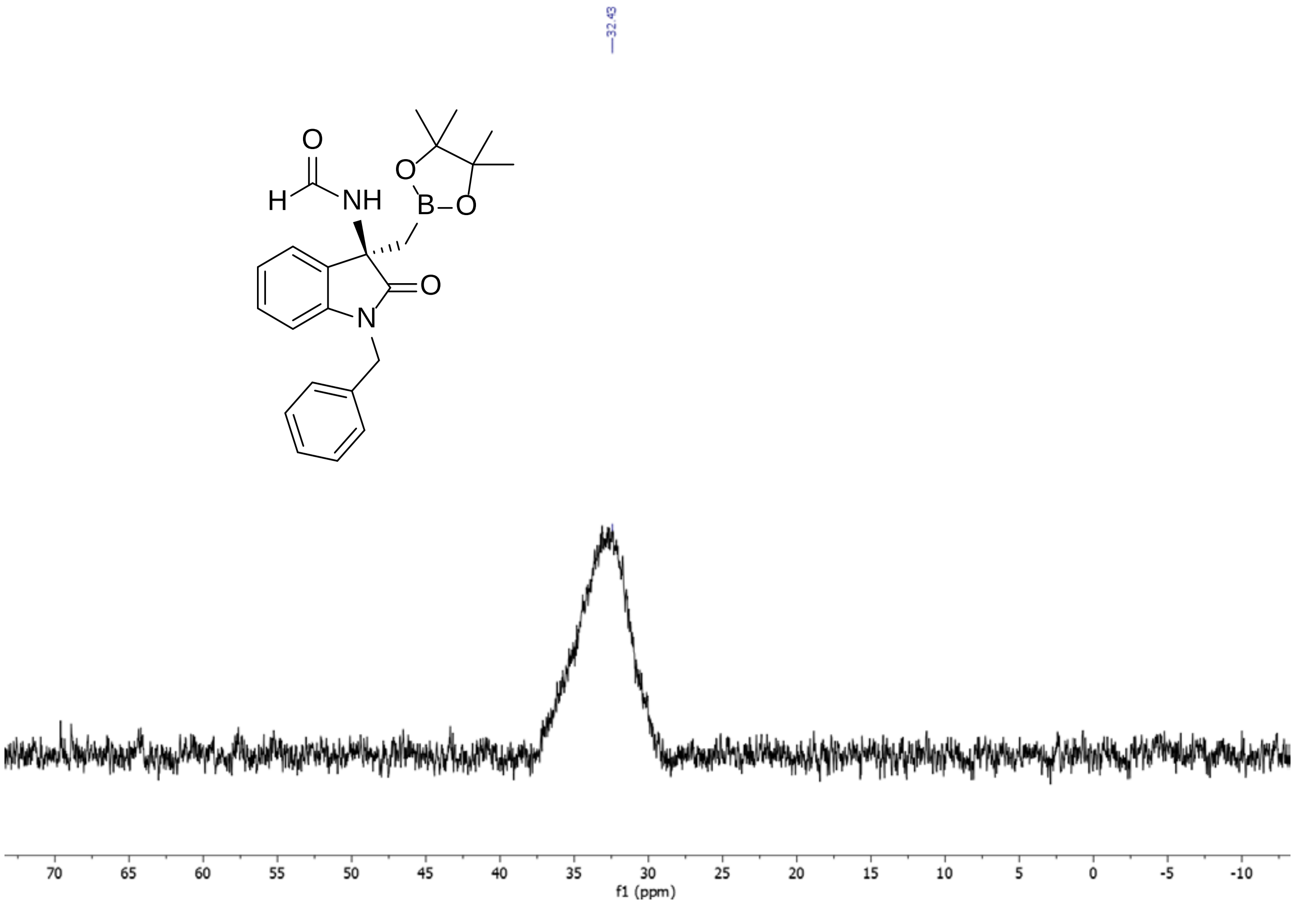

^{1}H NMR (400 MHz, $CDCl_3$) of compound **3a**

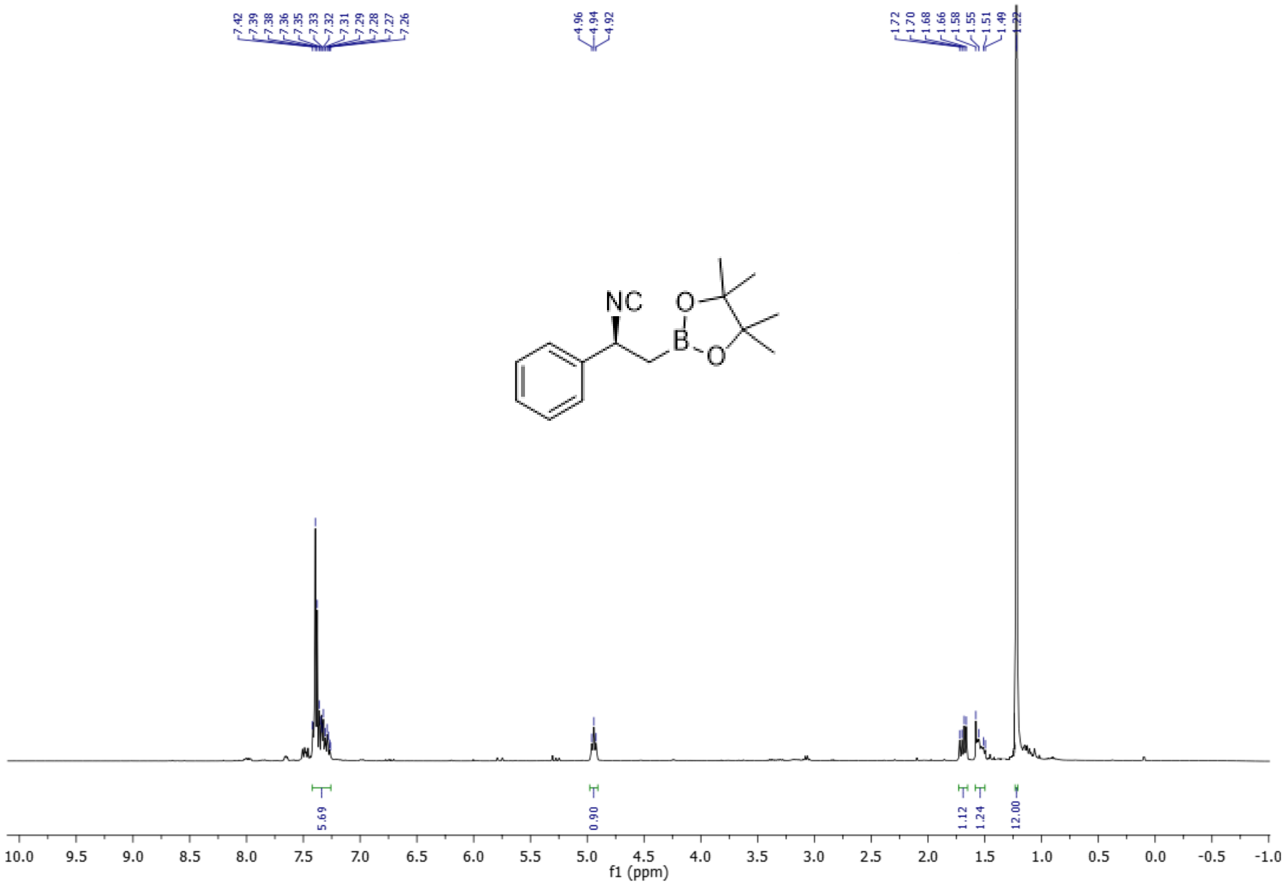

^{13}C NMR (101 MHz, $CDCl_3$) of compound **3a**

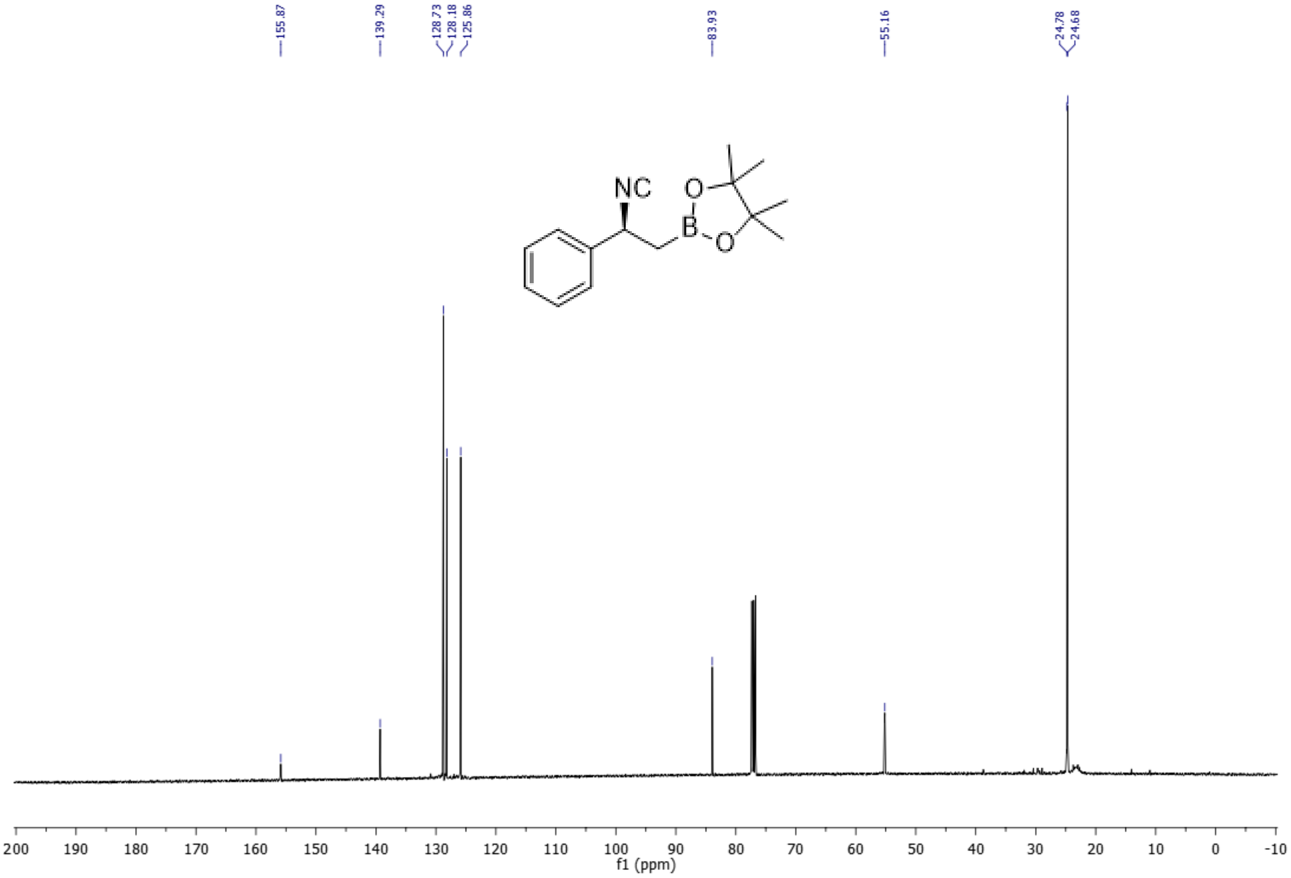

^{11}B NMR (128 MHz, $CDCl_3$) of compound **3a**

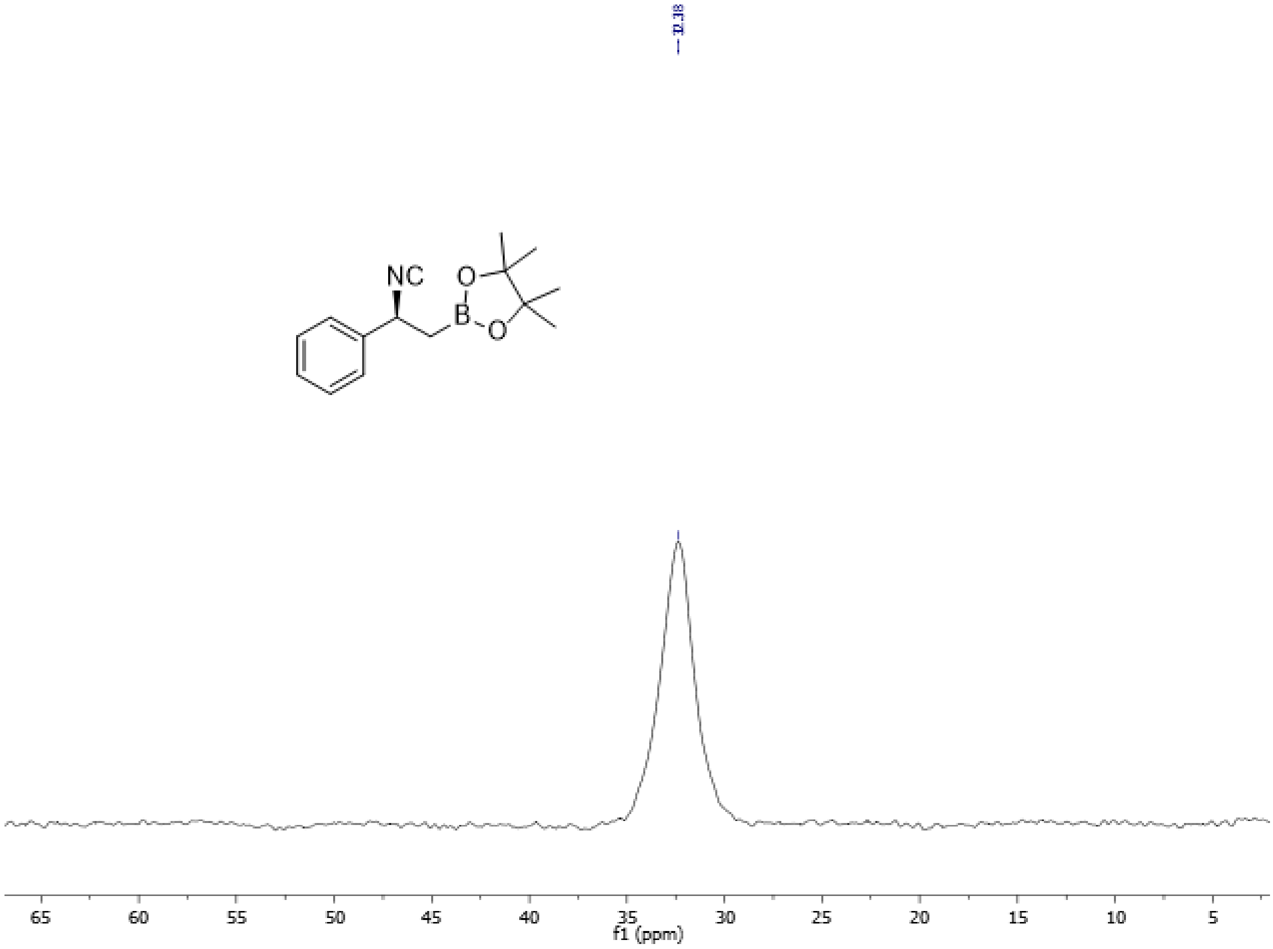

^{1}H NMR (400 MHz, $CDCl_3$) of compound **3b**

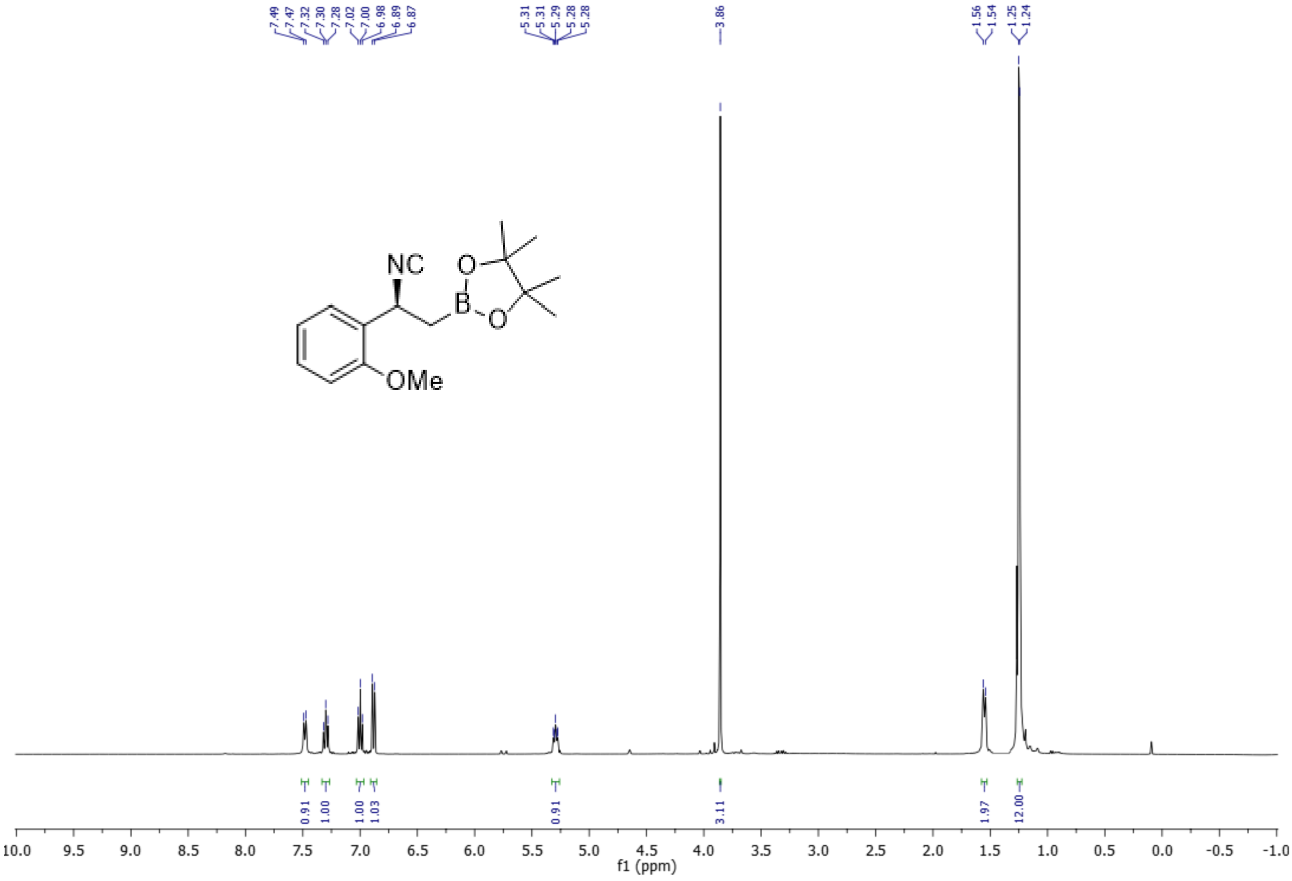

^{13}C NMR (101 MHz, $CDCl_3$) of compound **3b**

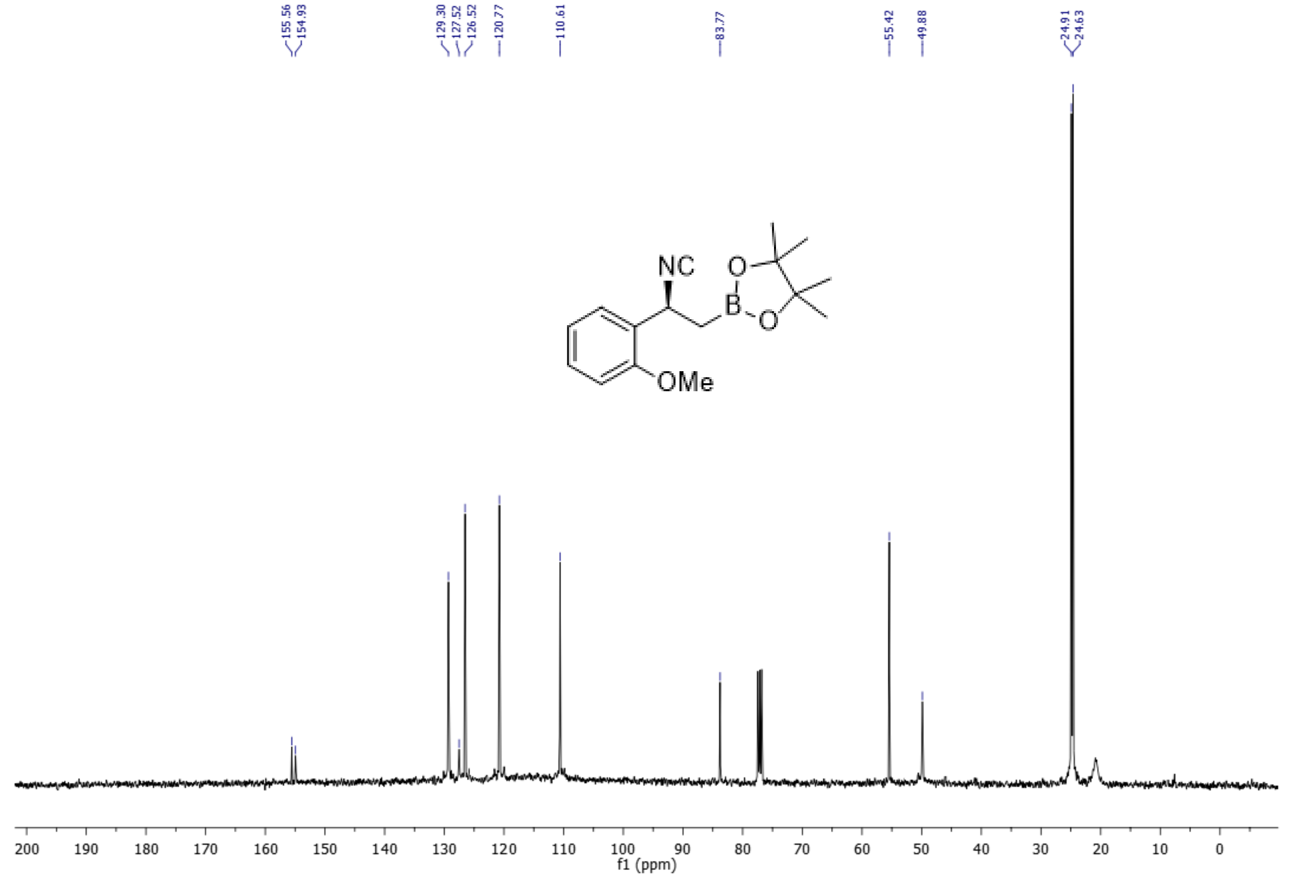

^{11}B NMR (128 MHz, $CDCl_3$) of compound **3b**

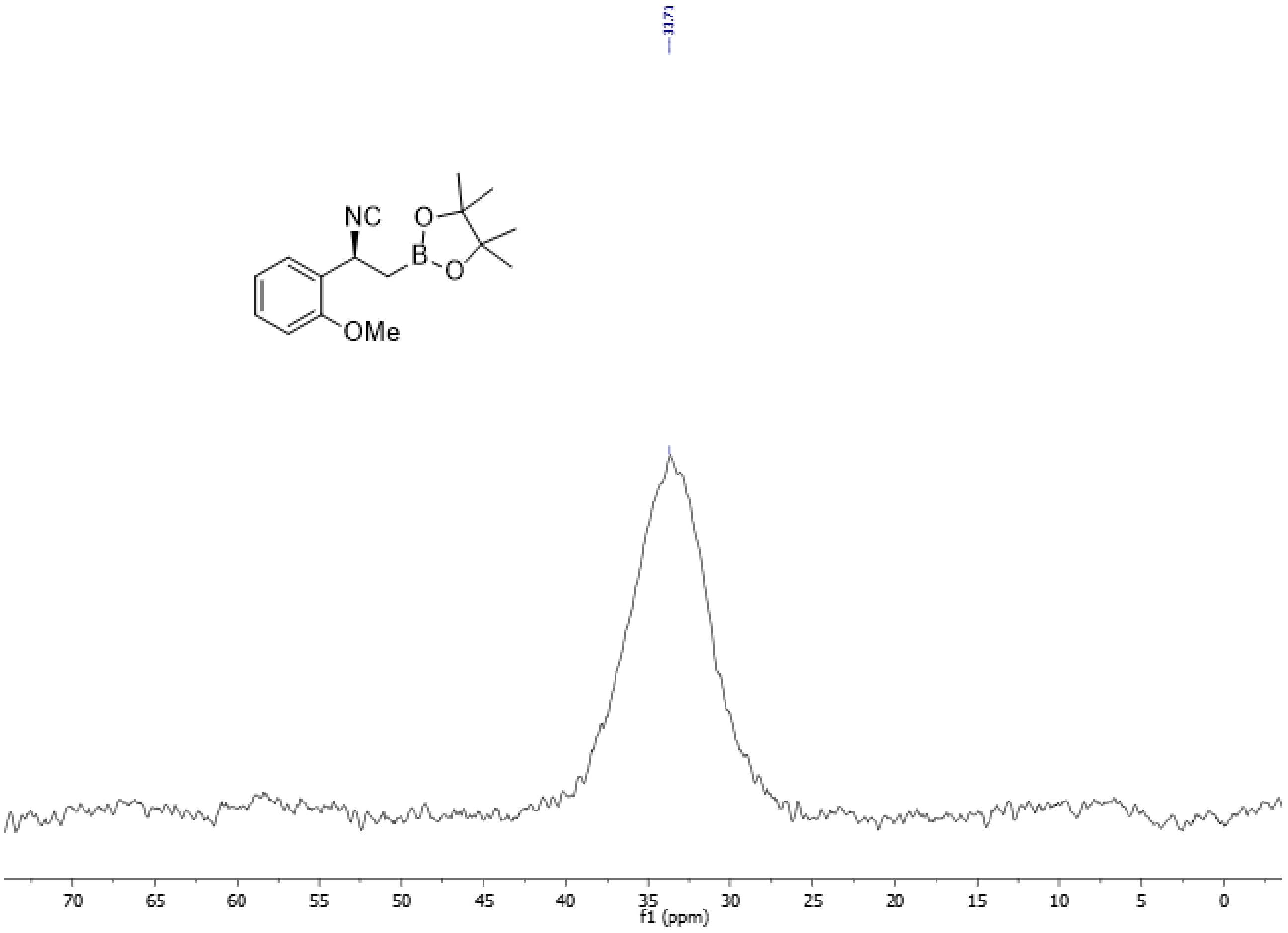

^{1}H NMR (400 MHz, $CDCl_3$) of compound **3c**

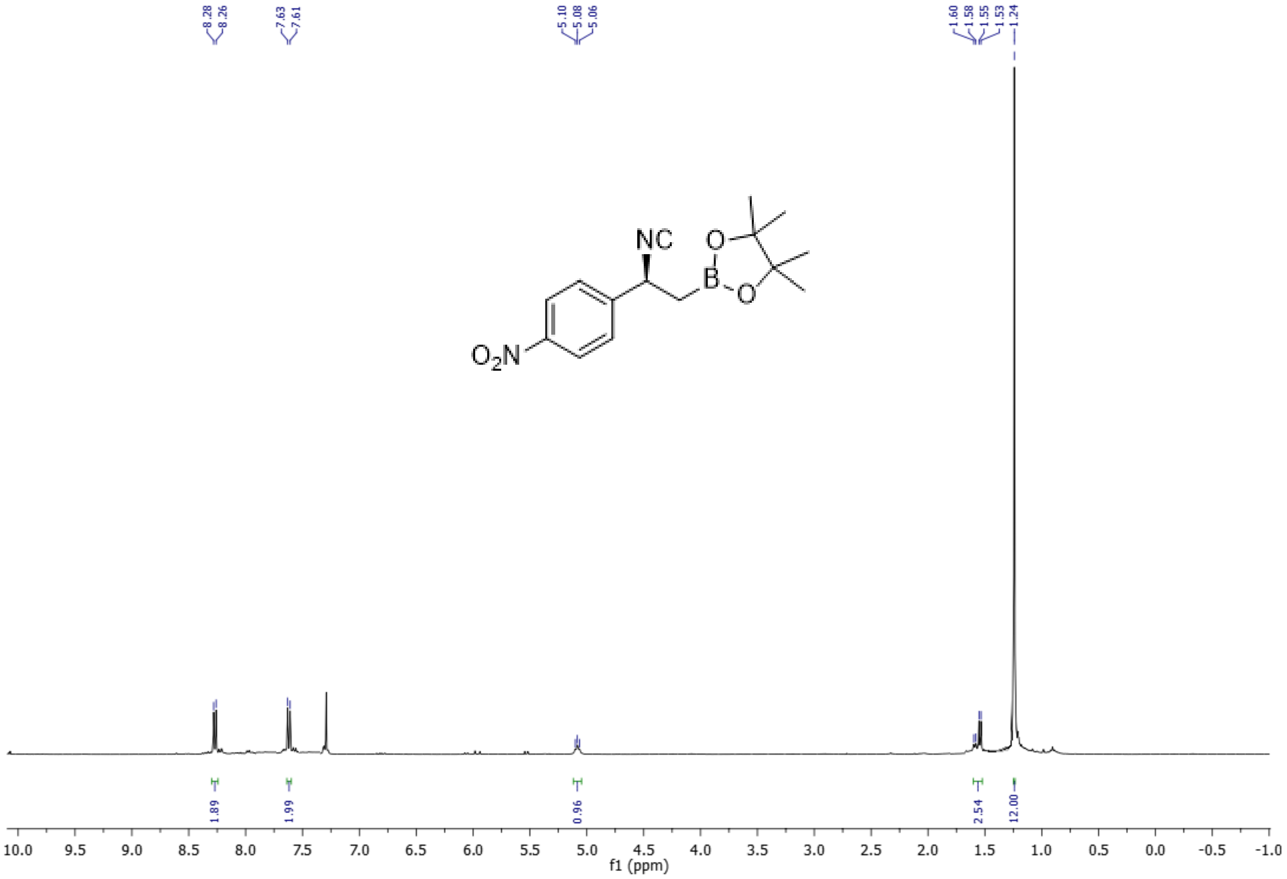

^{13}C NMR (101 MHz, $CDCl_3$) of compound **3c**

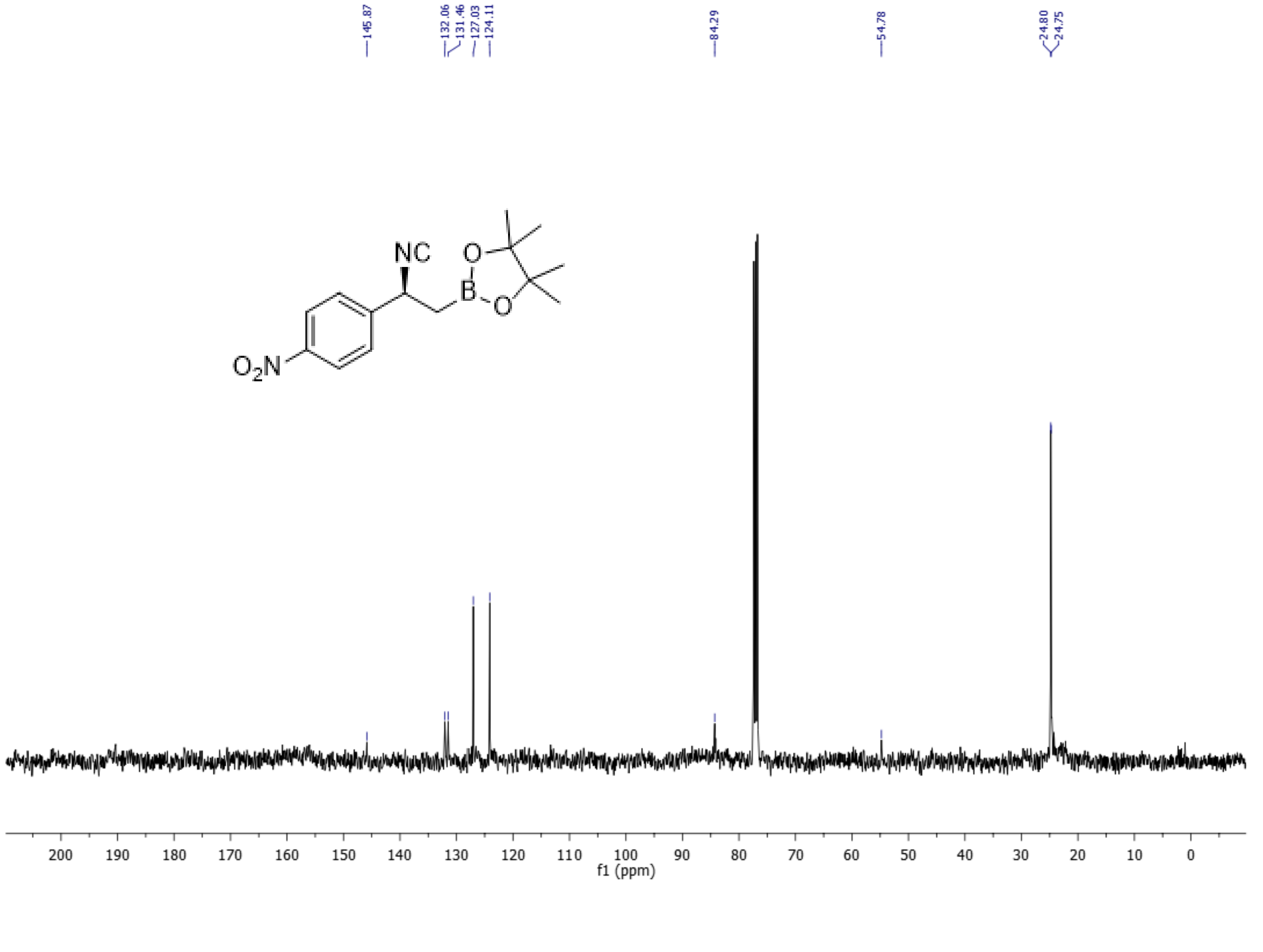

^{11}B NMR (128 MHz, $CDCl_3$) of compound **3c**

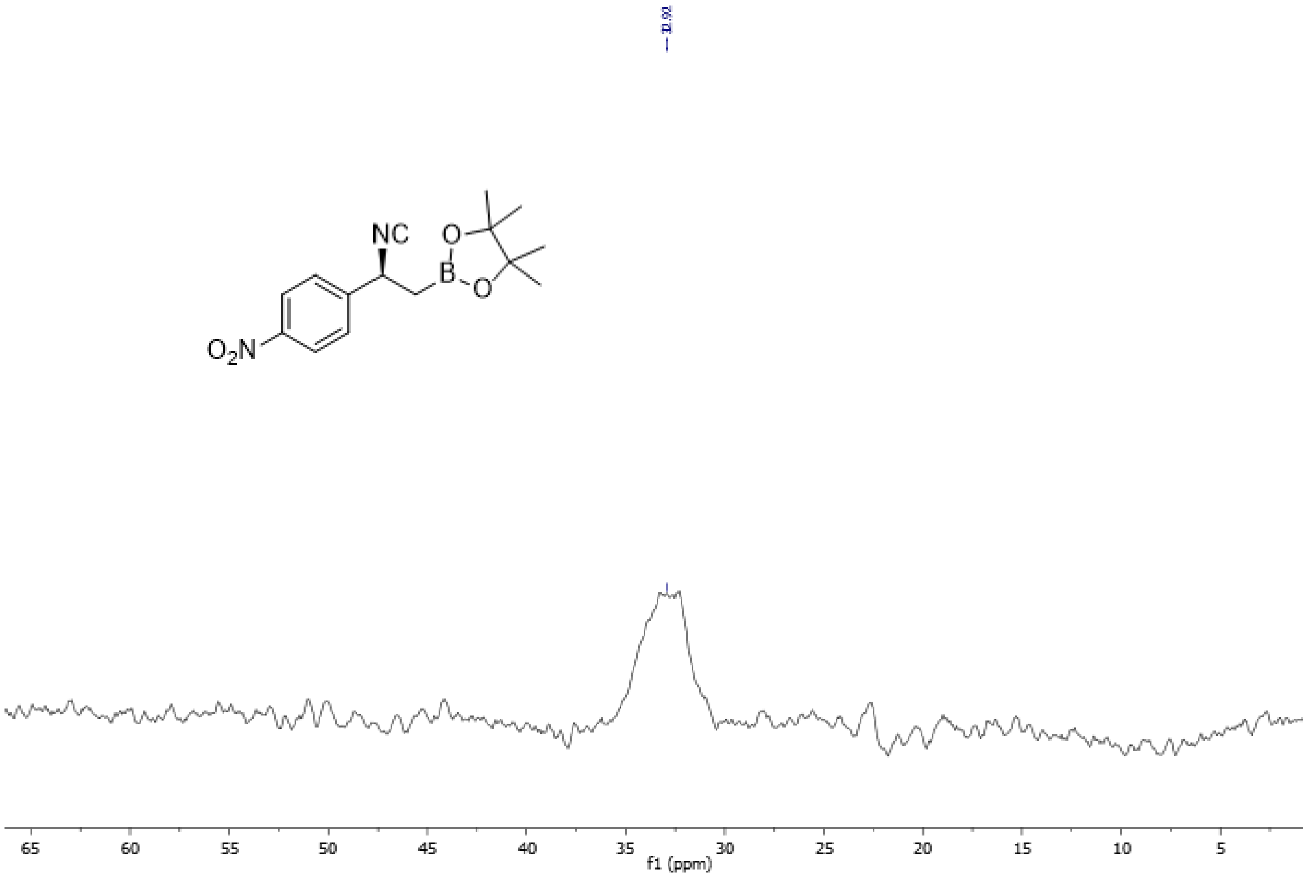

^{1}H NMR (400 MHz, $CDCl_3$) of compound **3d**

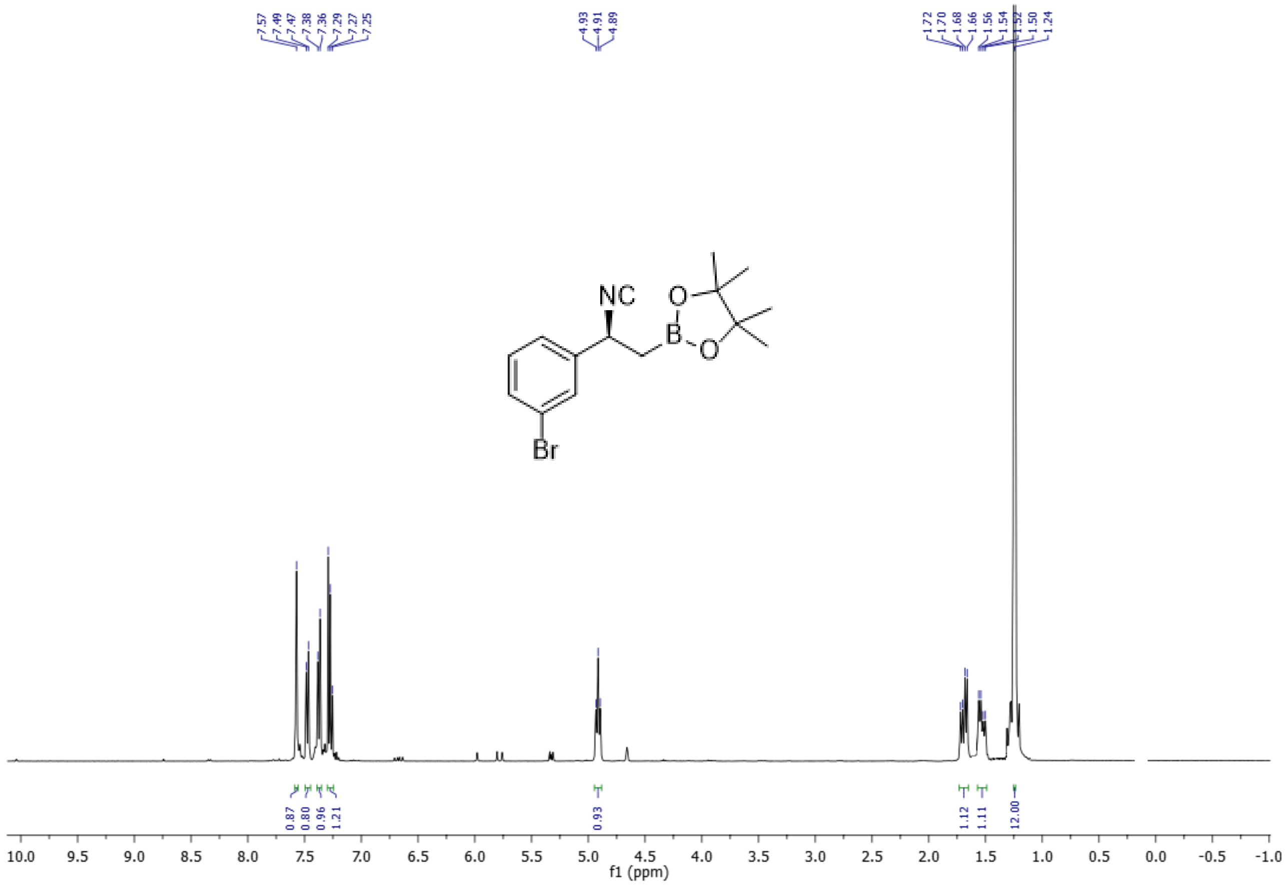

^{13}C NMR (101 MHz, $CDCl_3$) of compound **3d**

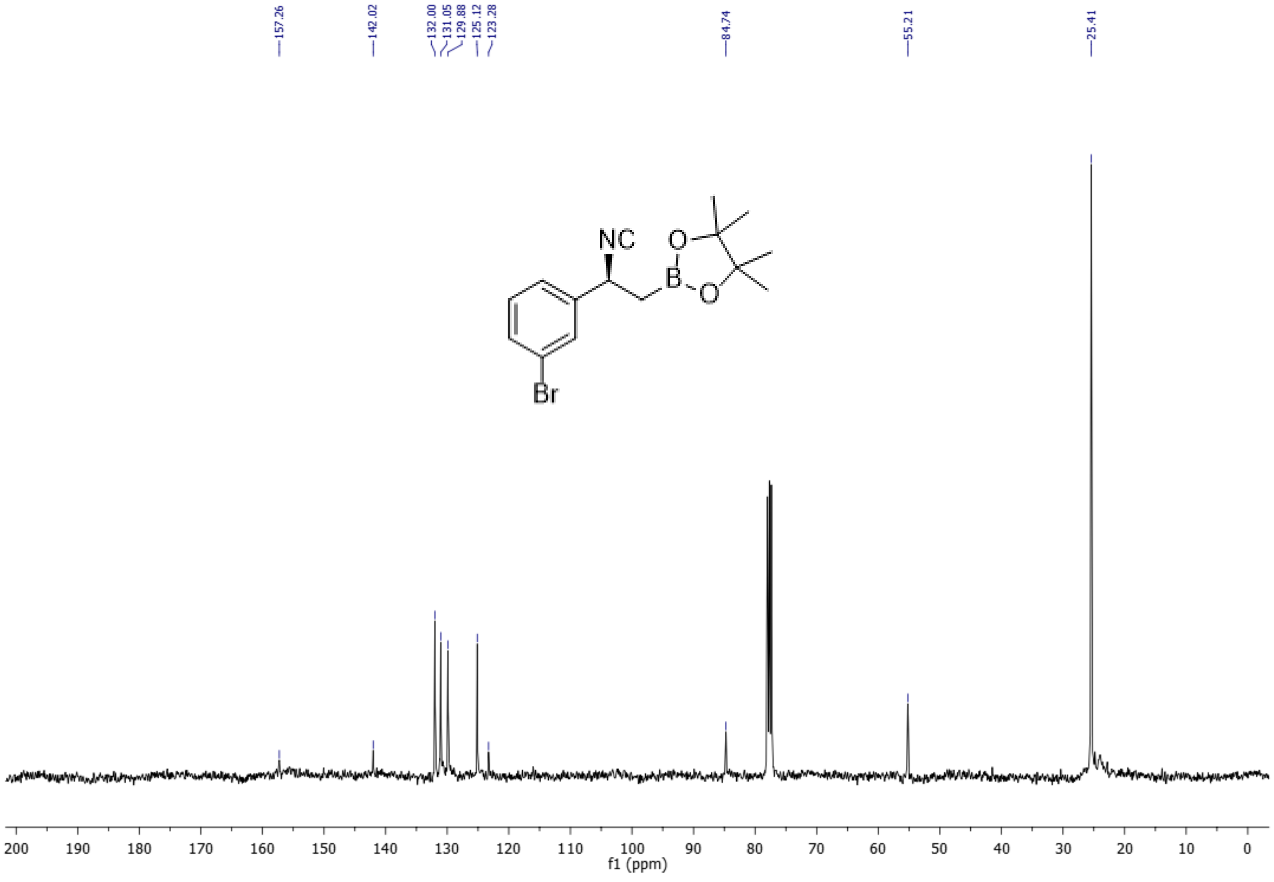

^{11}B NMR (128 MHz, $CDCl_3$) of compound **3d**

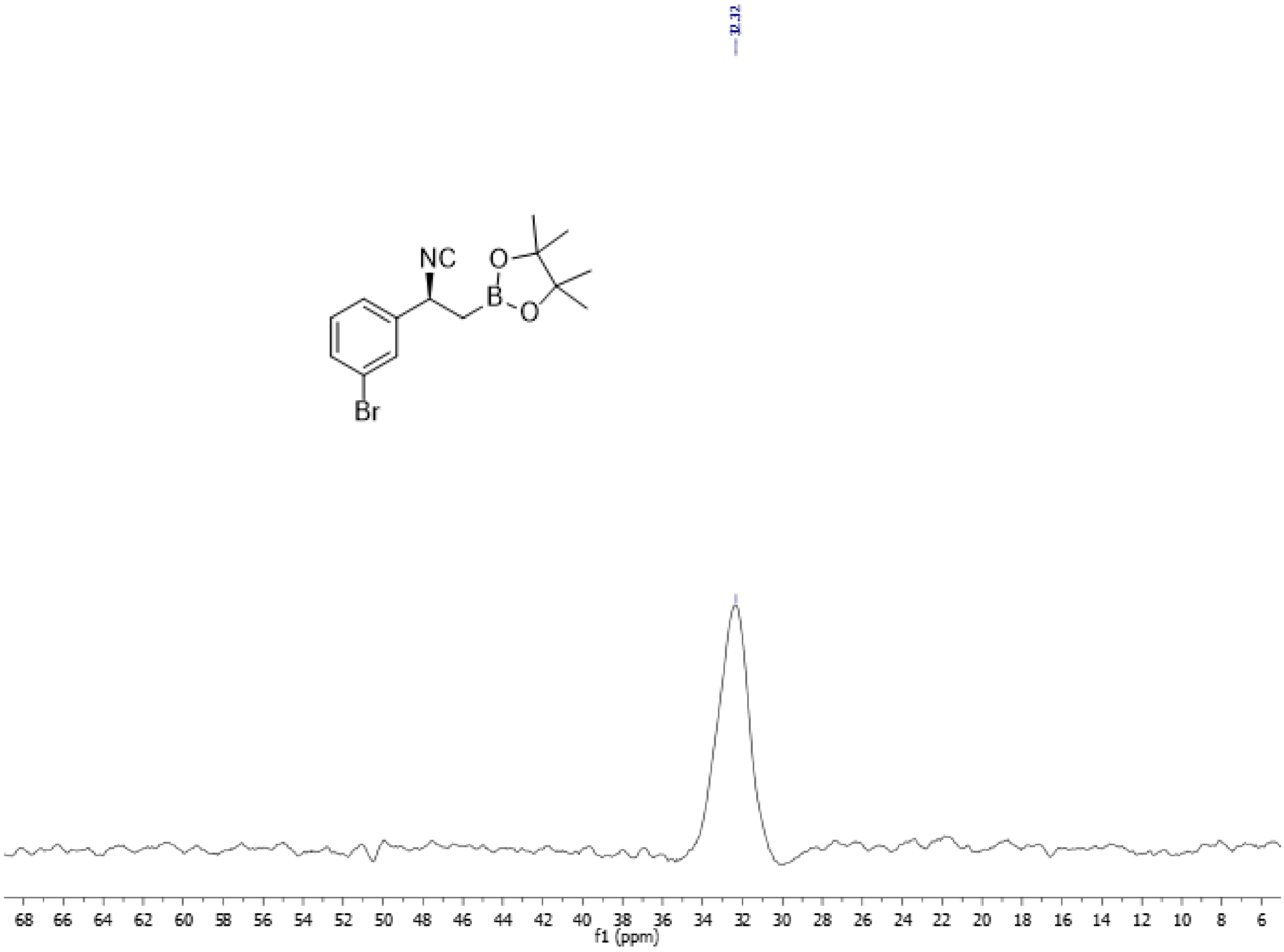

^{1}H NMR (400 MHz, $CDCl_3$) of compound **3e**

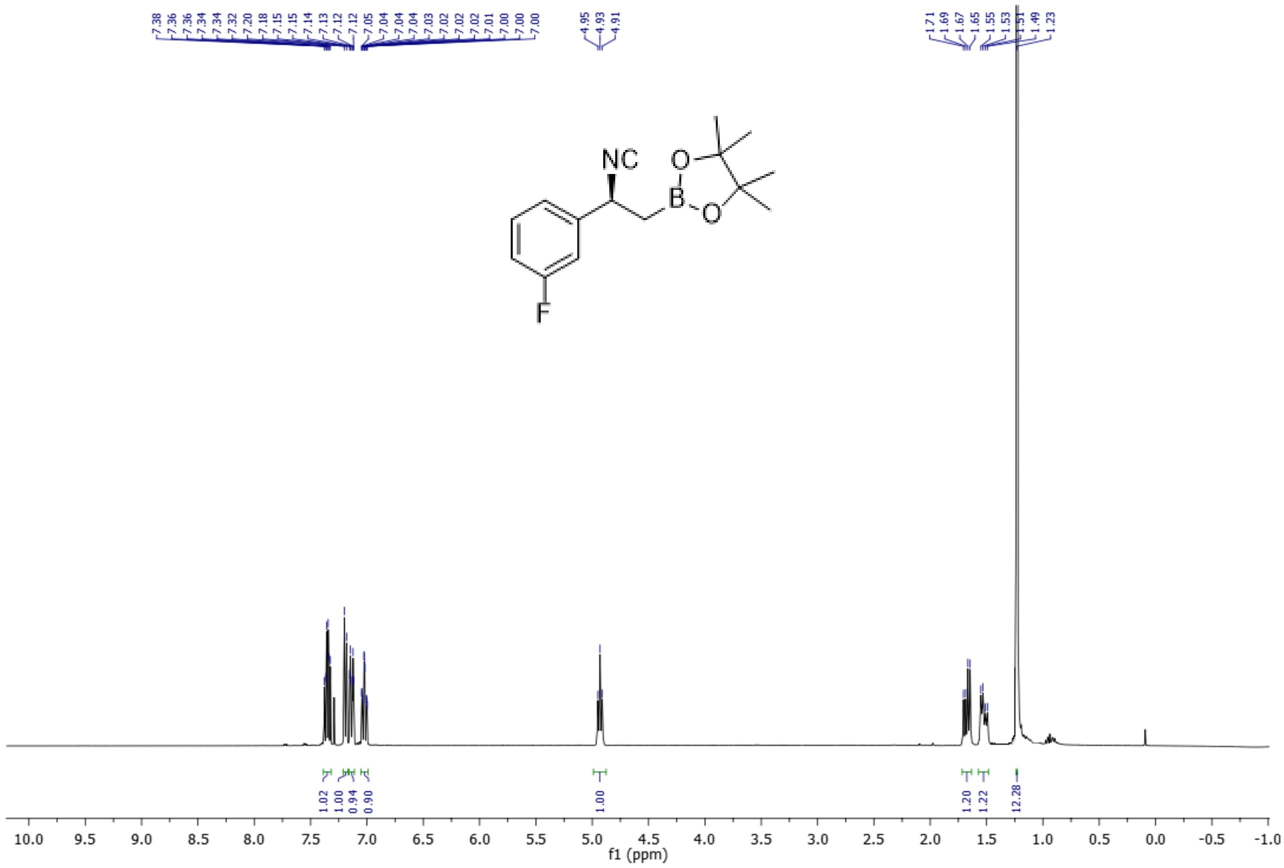

^{13}C NMR (101 MHz, $CDCl_3$) of compound **3e**

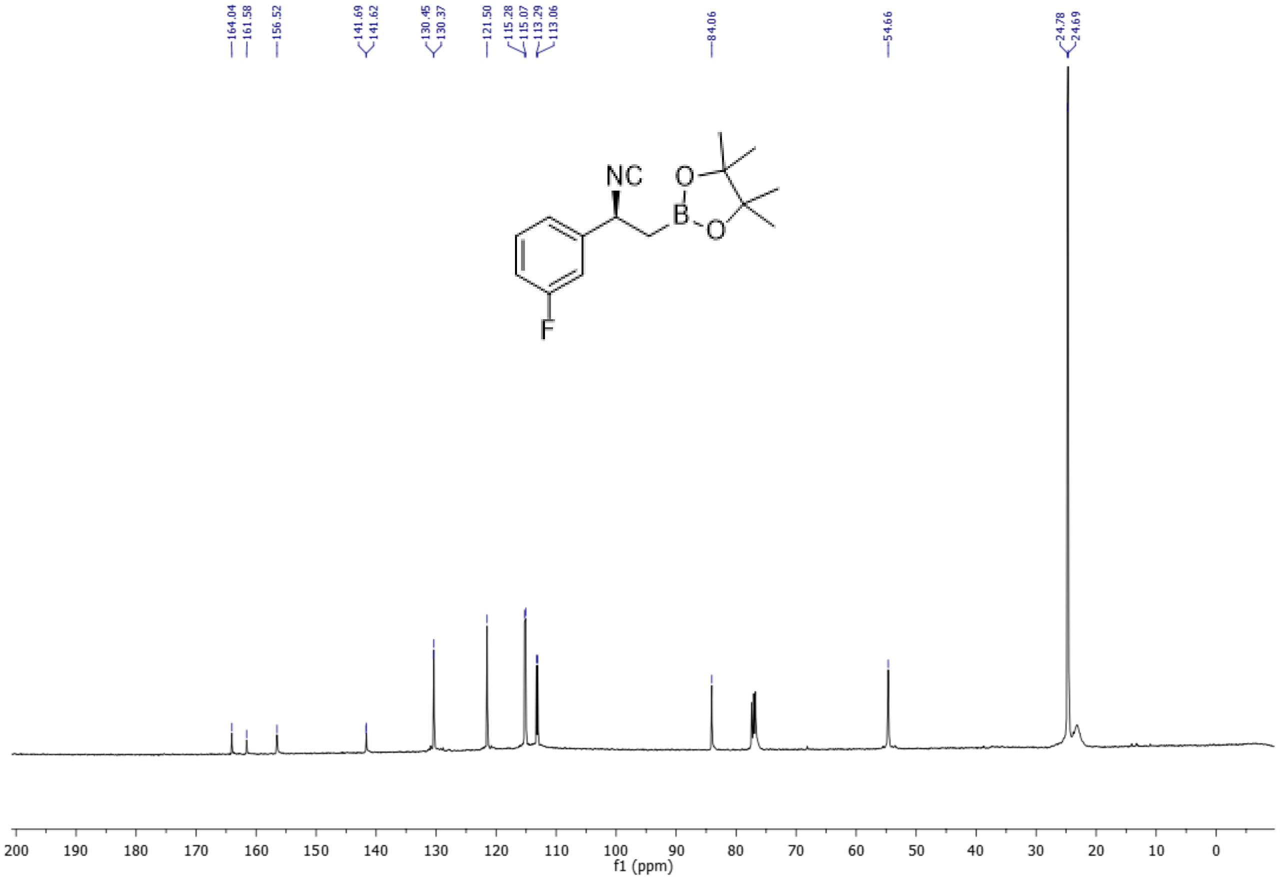

^{11}B NMR (128 MHz, $CDCl_3$) of compound **3e**

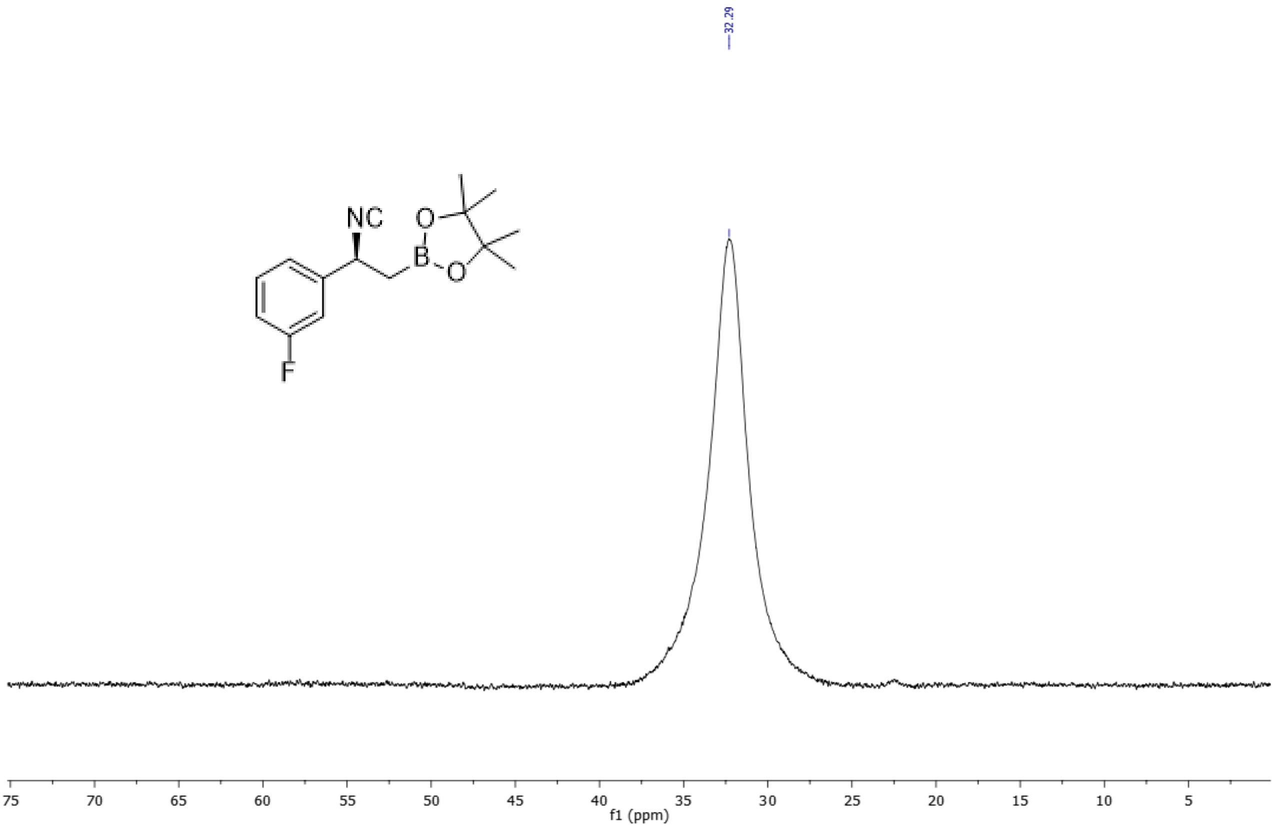

^{1}H NMR (400 MHz, CDCl$_3$) of compound **3f**

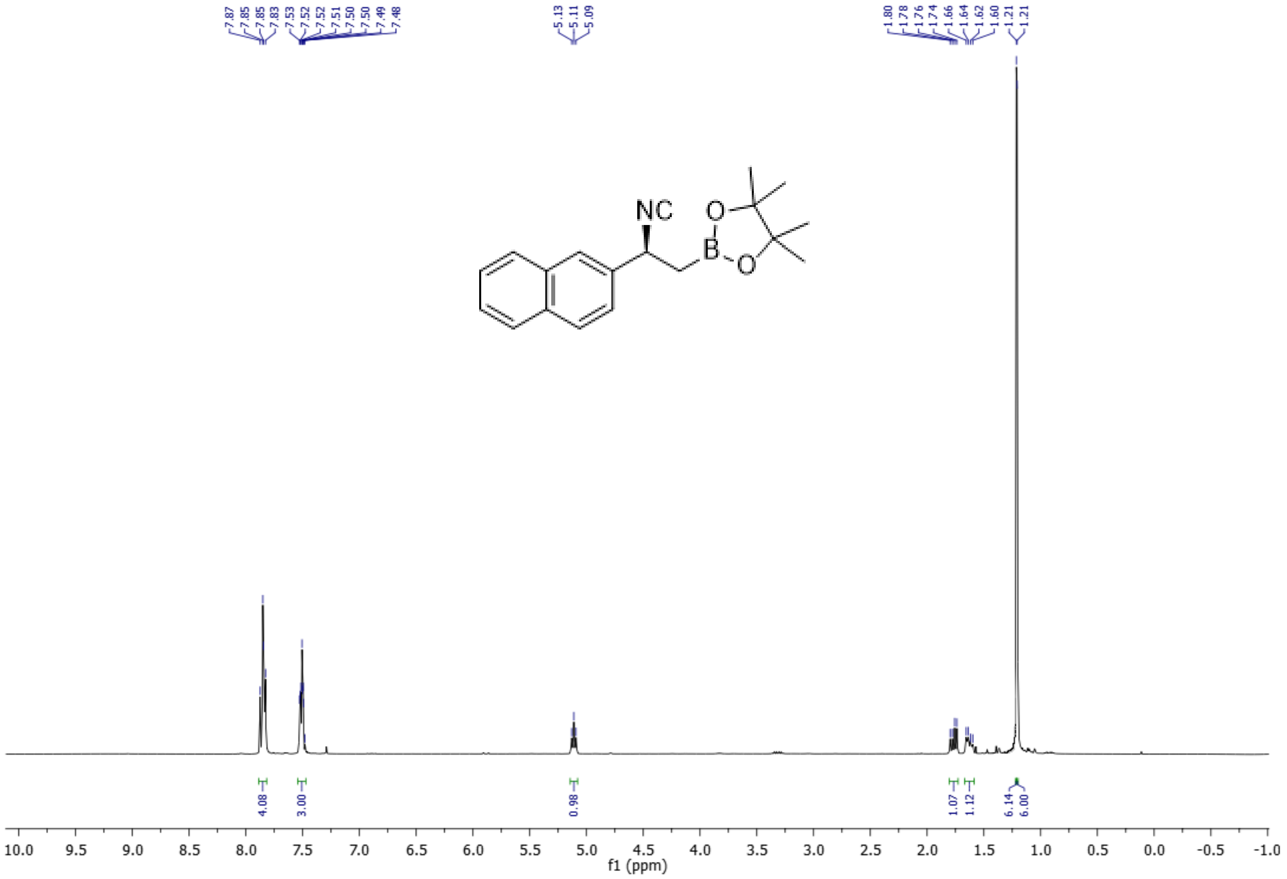

^{13}C NMR (101 MHz, CDCl$_3$) of compound **3f**

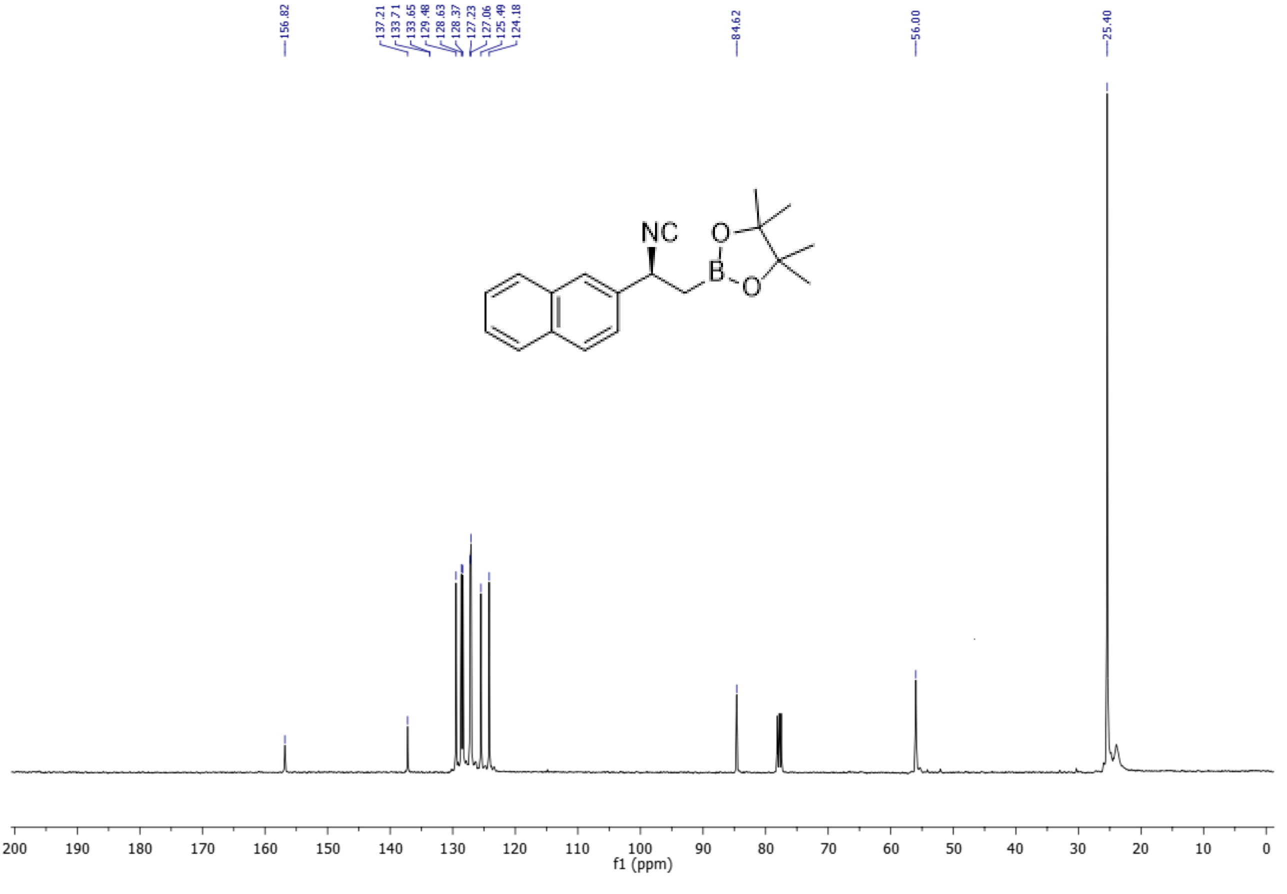

^{11}B NMR (128 MHz, $CDCl_3$) of compound **3f**

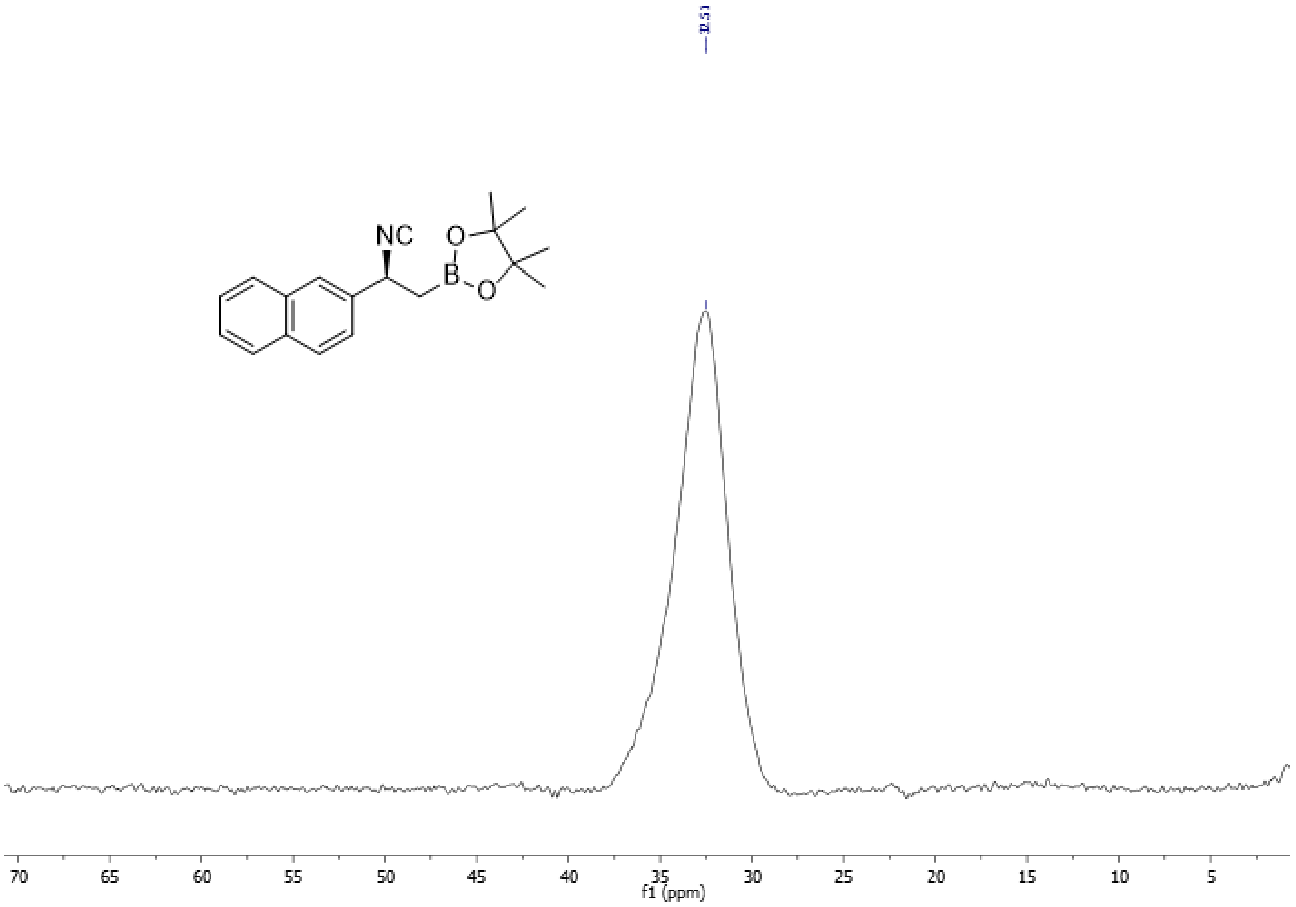

^{1}H NMR (400 MHz, $CDCl_3$) of compound **3g**

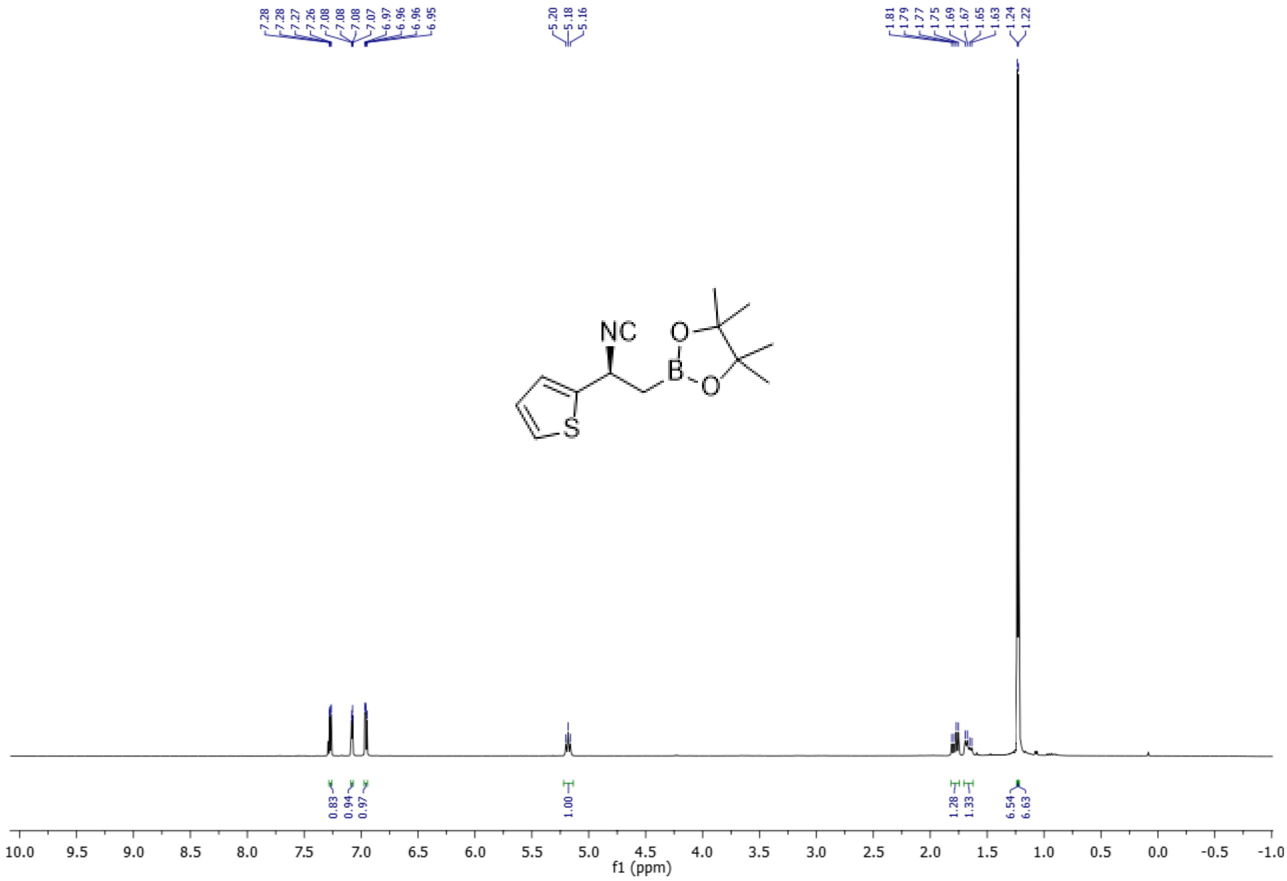

^{13}C NMR (101 MHz, $CDCl_3$) of compound **3g**

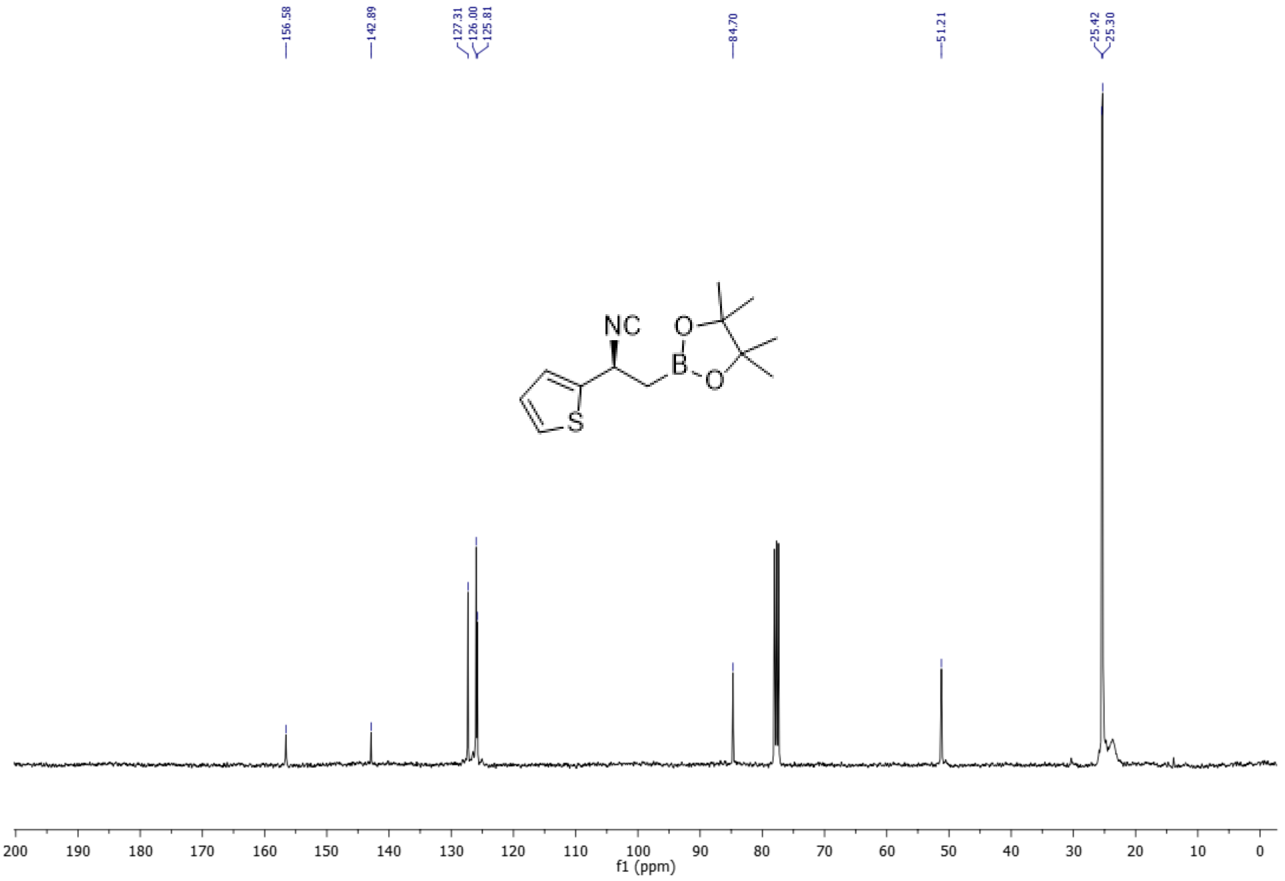

^{11}B NMR (128 MHz, $CDCl_3$) of compound **3g**

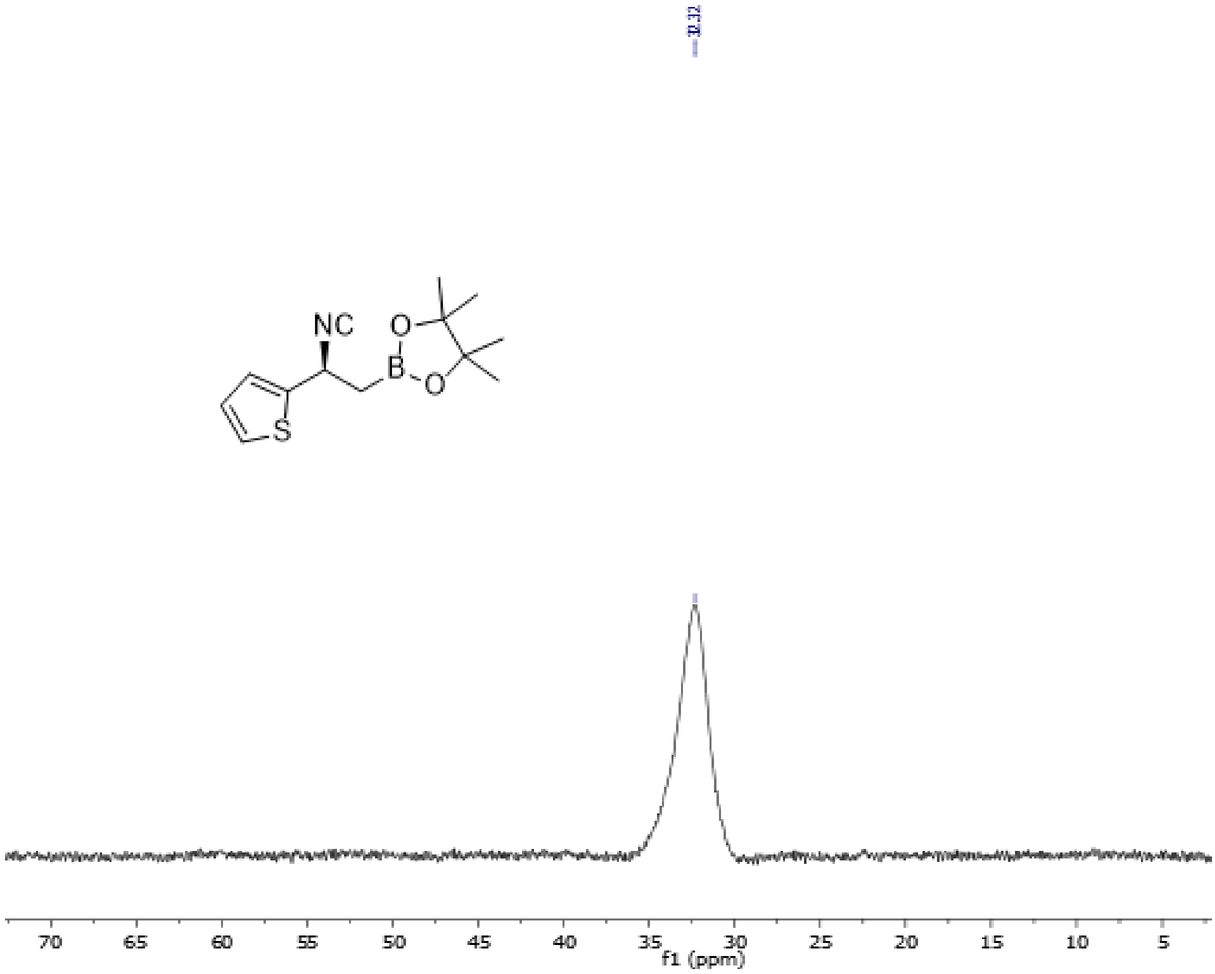

^{1}H NMR (400 MHz, $CDCl_3$) of compound **3h**

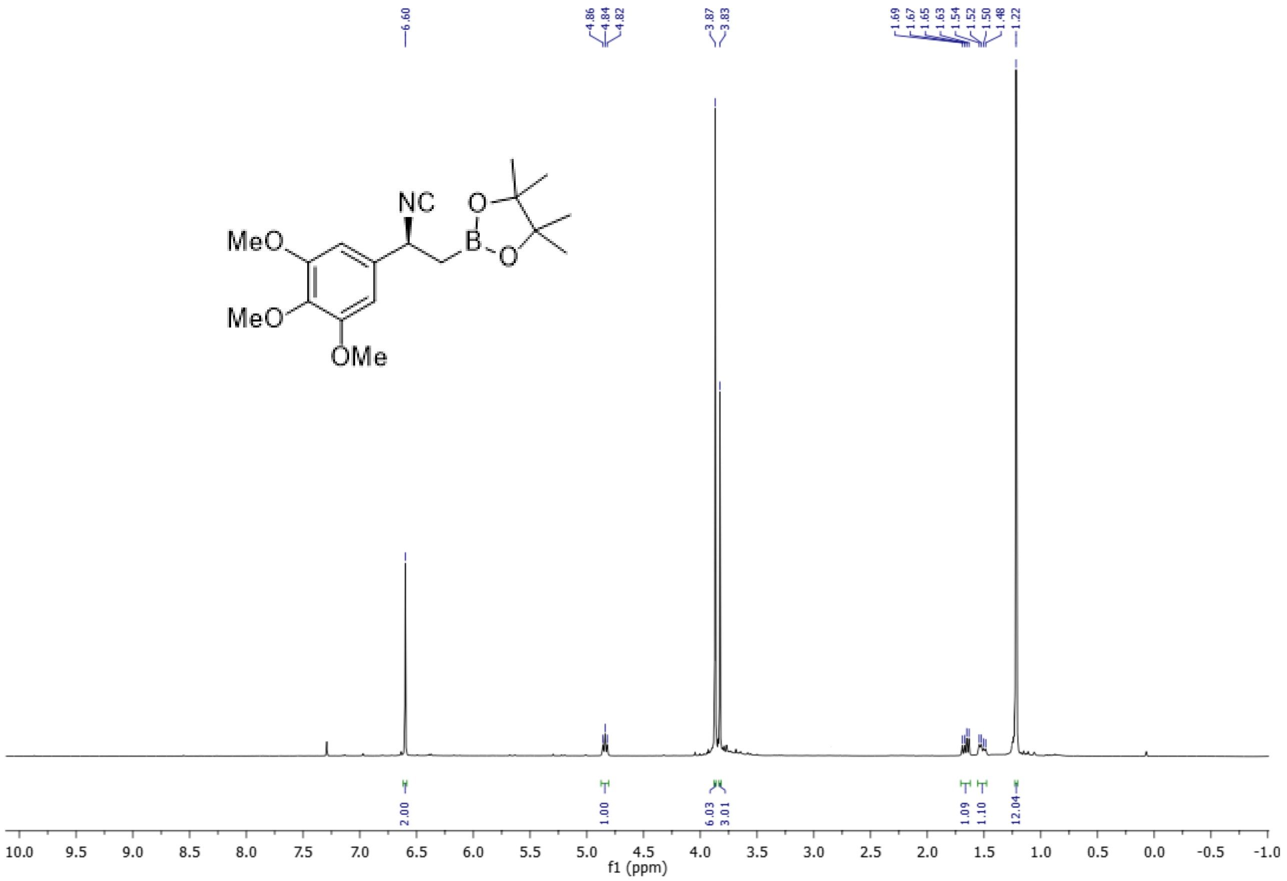

^{13}C NMR (101 MHz, $CDCl_3$) of compound **3h**

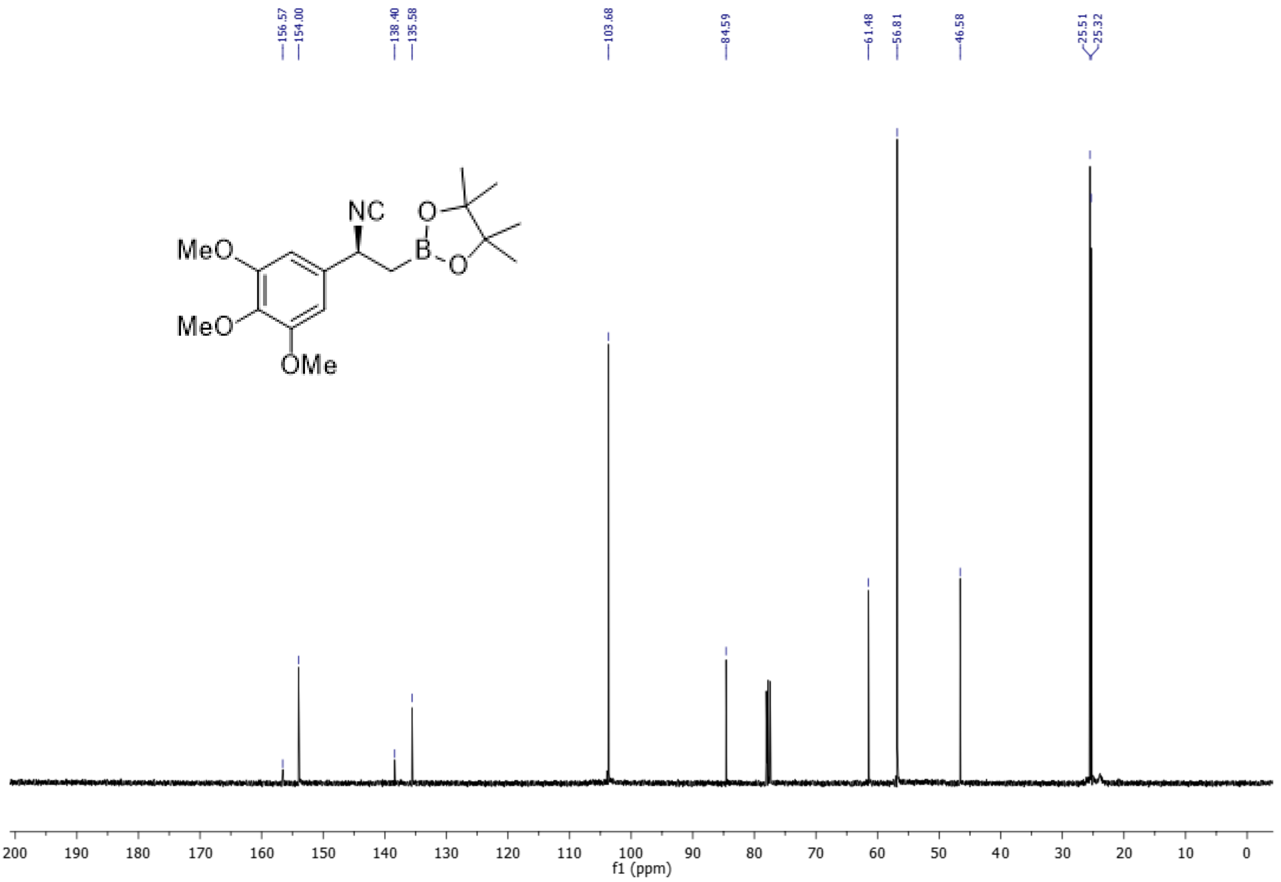

^{11}B NMR (128 MHz, $CDCl_3$) of compound **3h**

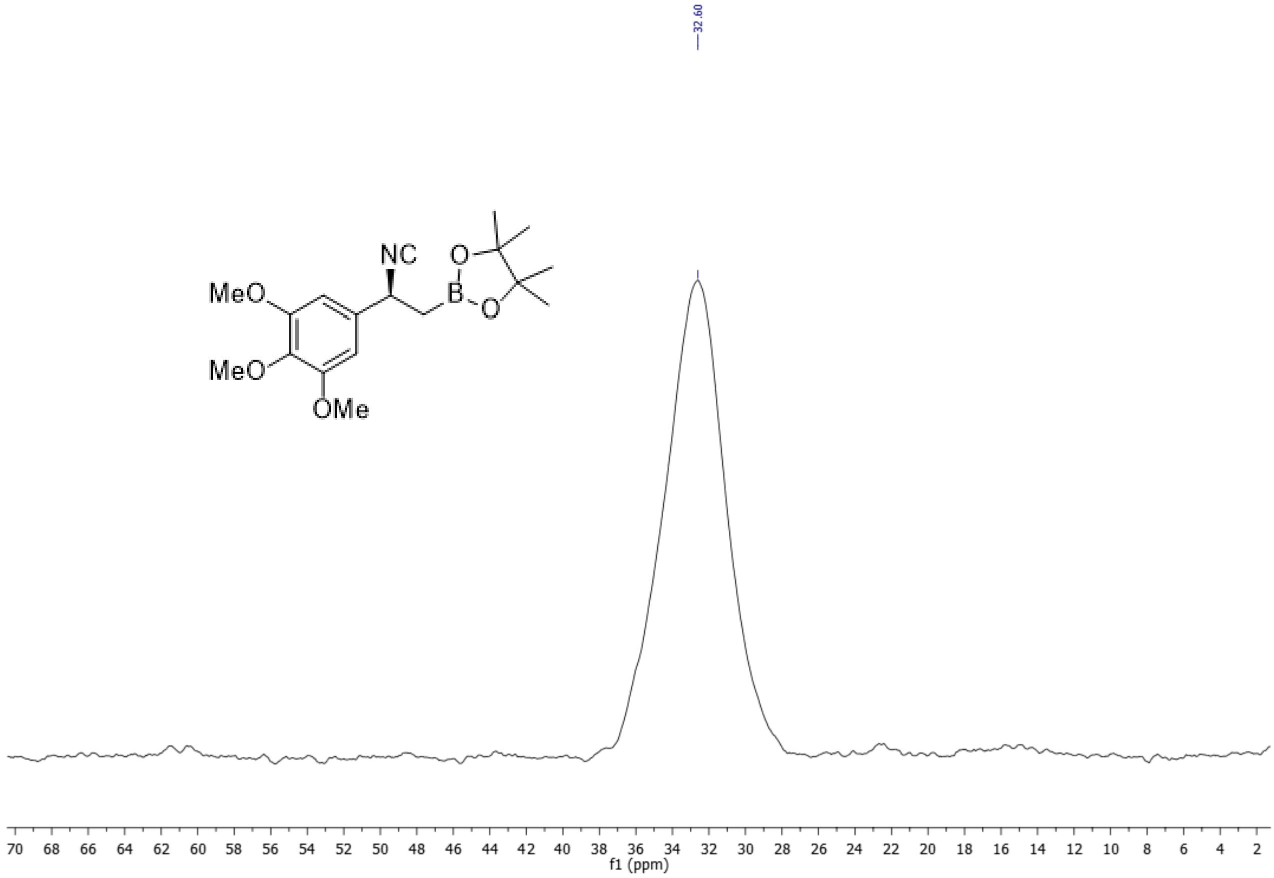

^{1}H NMR (400 MHz, $CDCl_3$) of compound **3i**

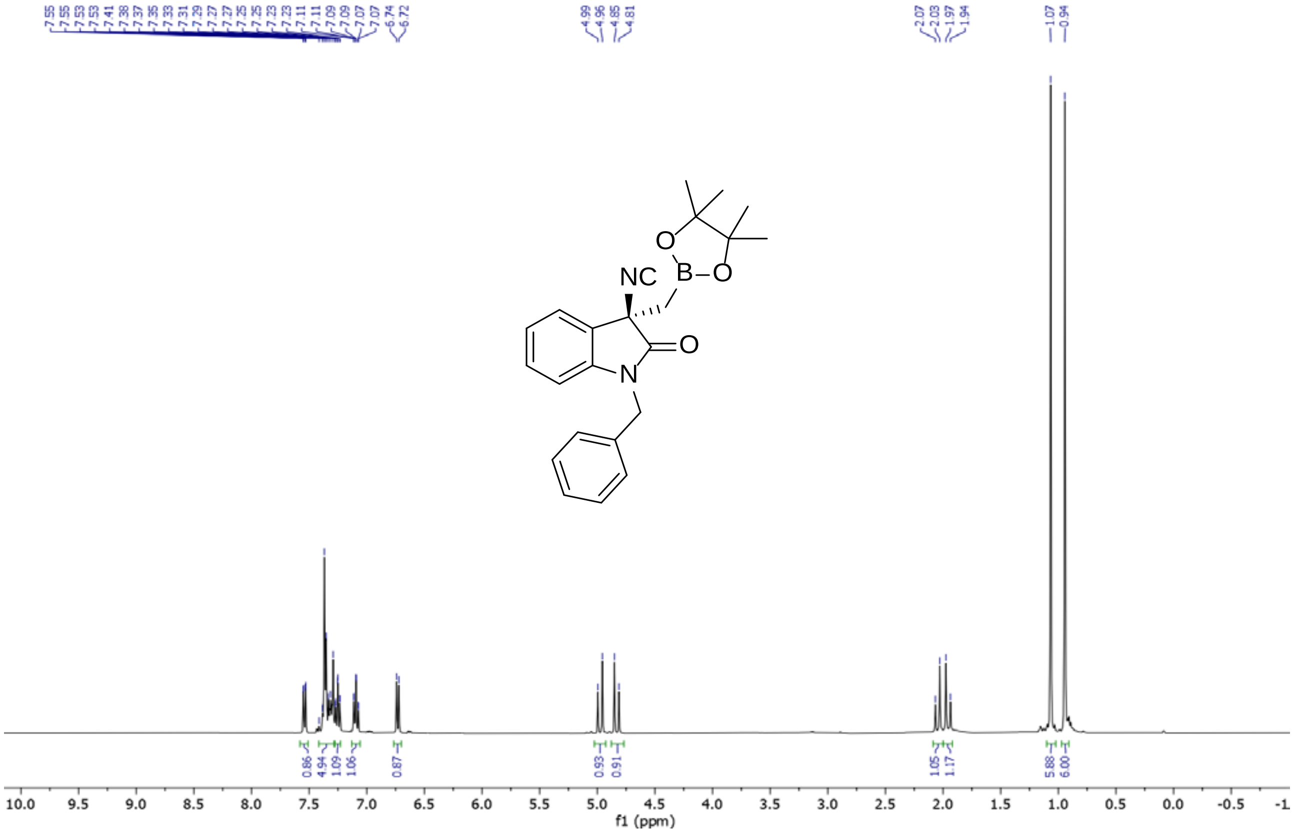

^{13}C NMR (101 MHz, $CDCl_3$) of compound **3i**

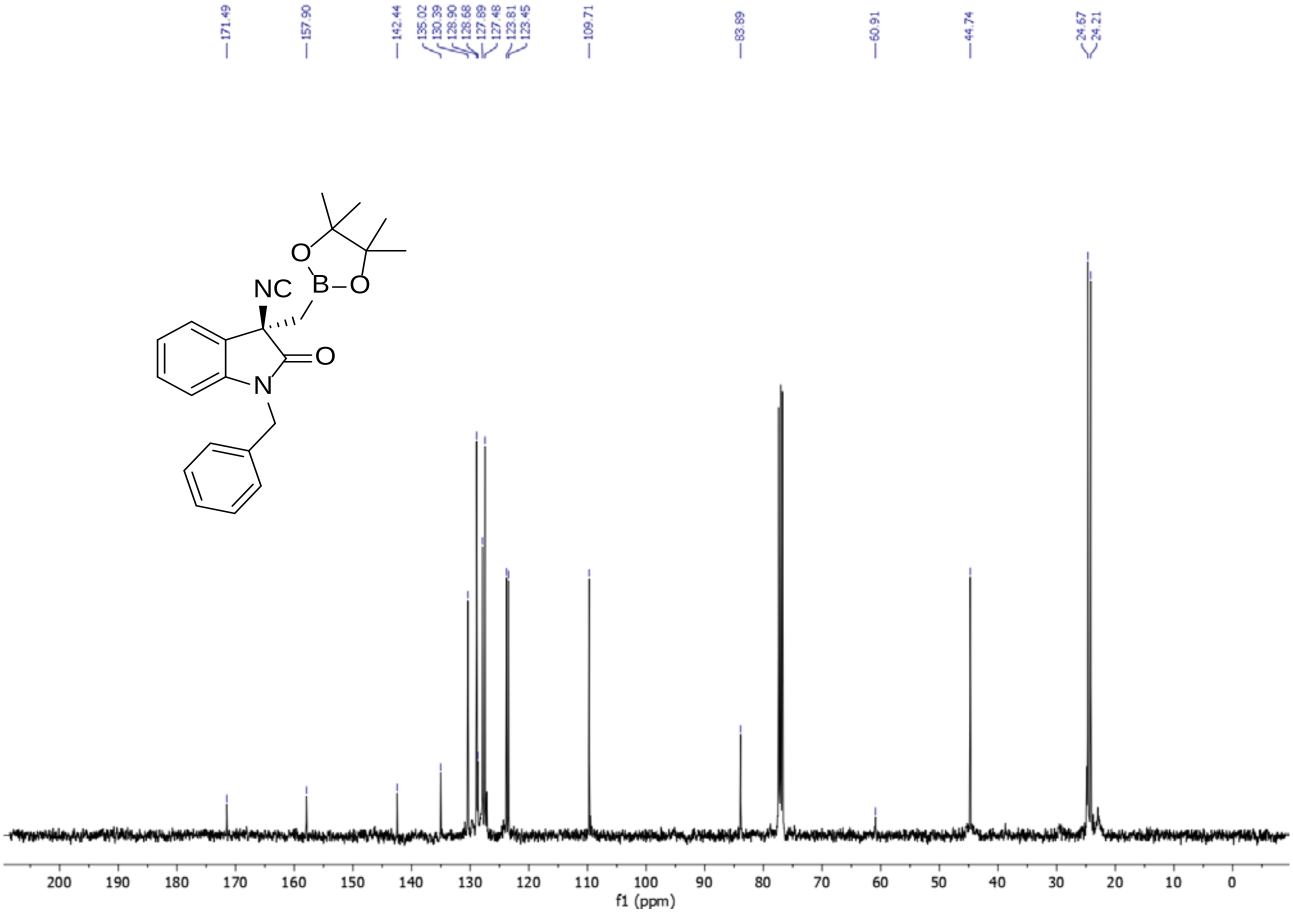

^{11}B NMR (128 MHz, $CDCl_3$) of compound **3i**

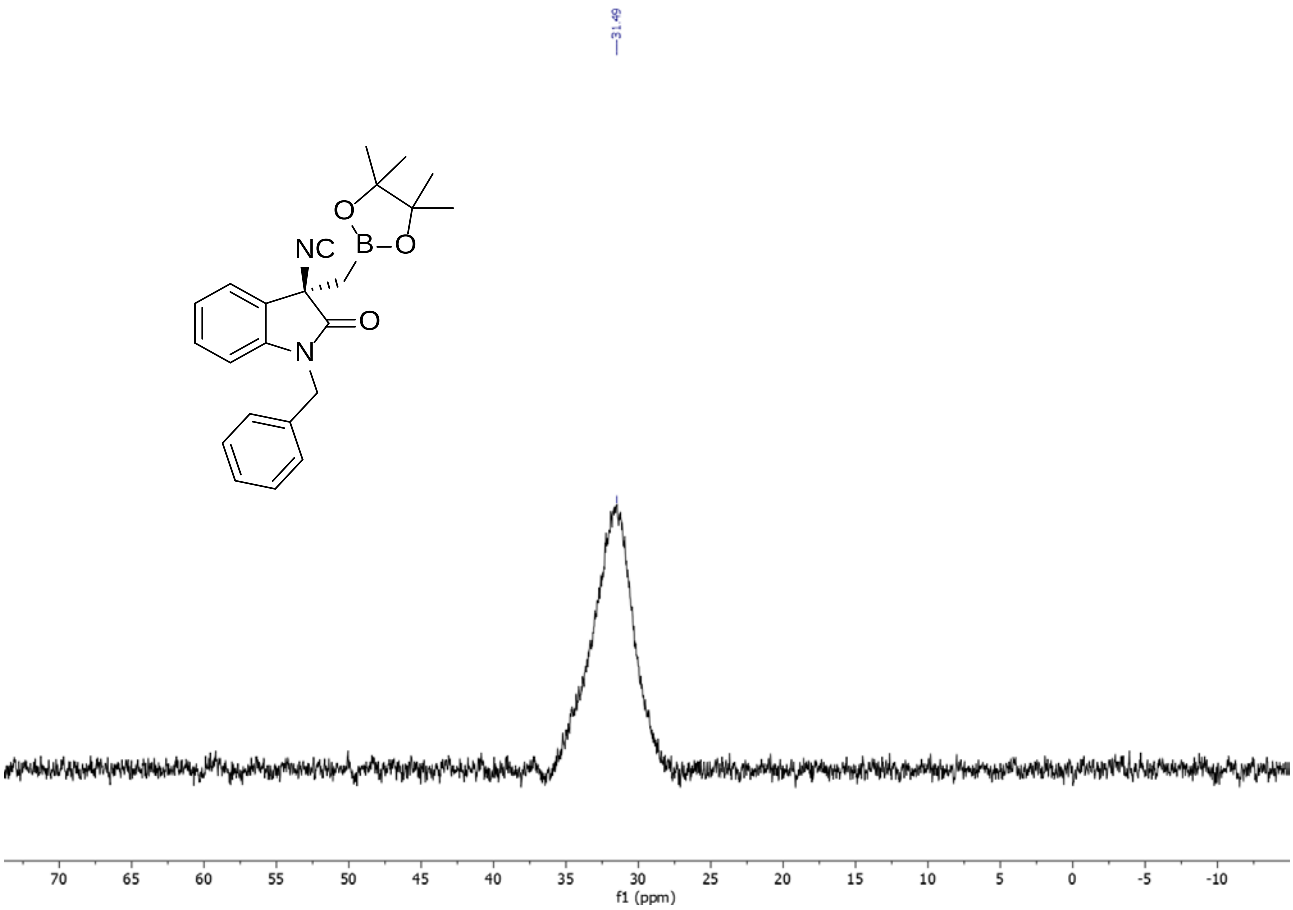

^{1}H NMR (400 MHz, $CDCl_3$, rotamers mixture 65:35) of compound **4a**

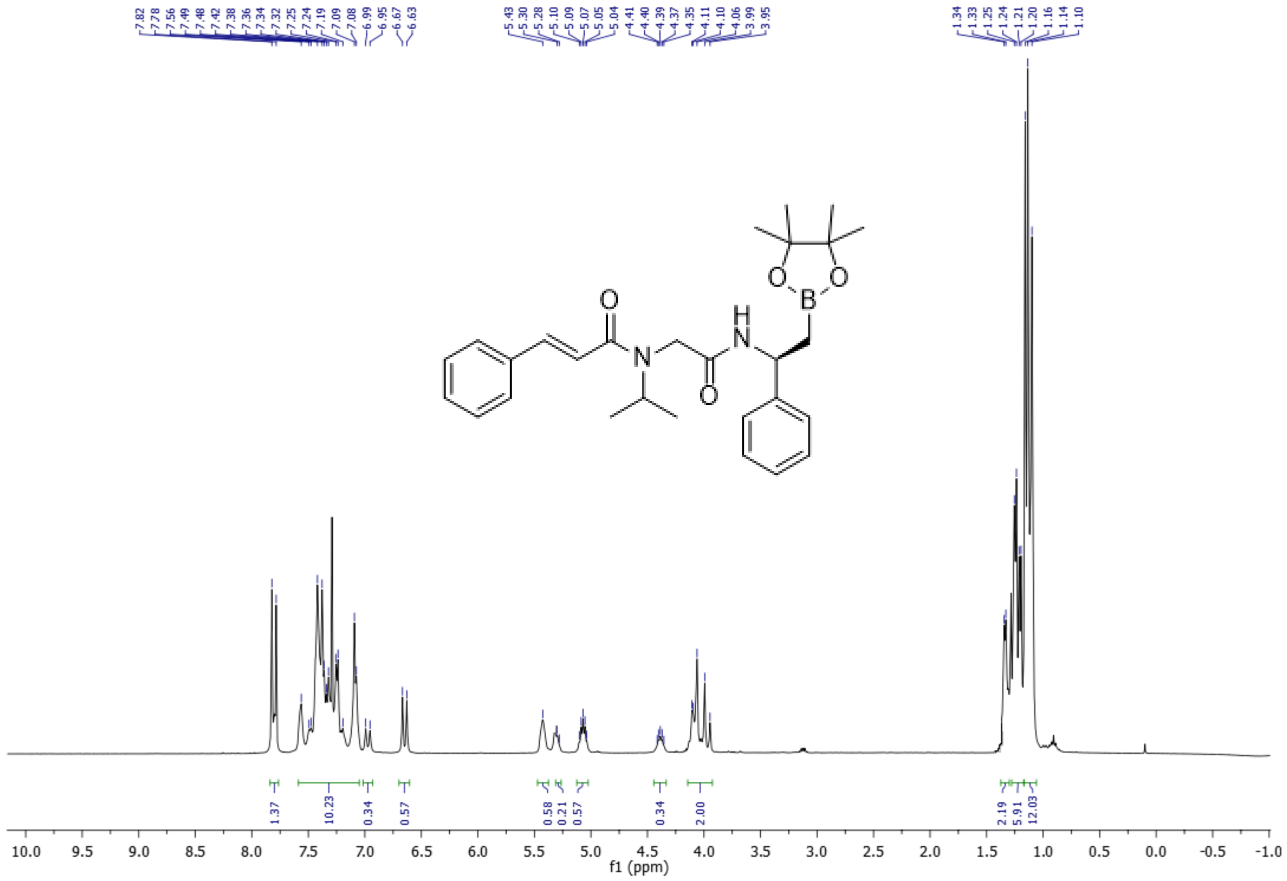

^{13}C NMR (101 MHz, $CDCl_3$, rotamers mixture 65:35) of compound **4a**

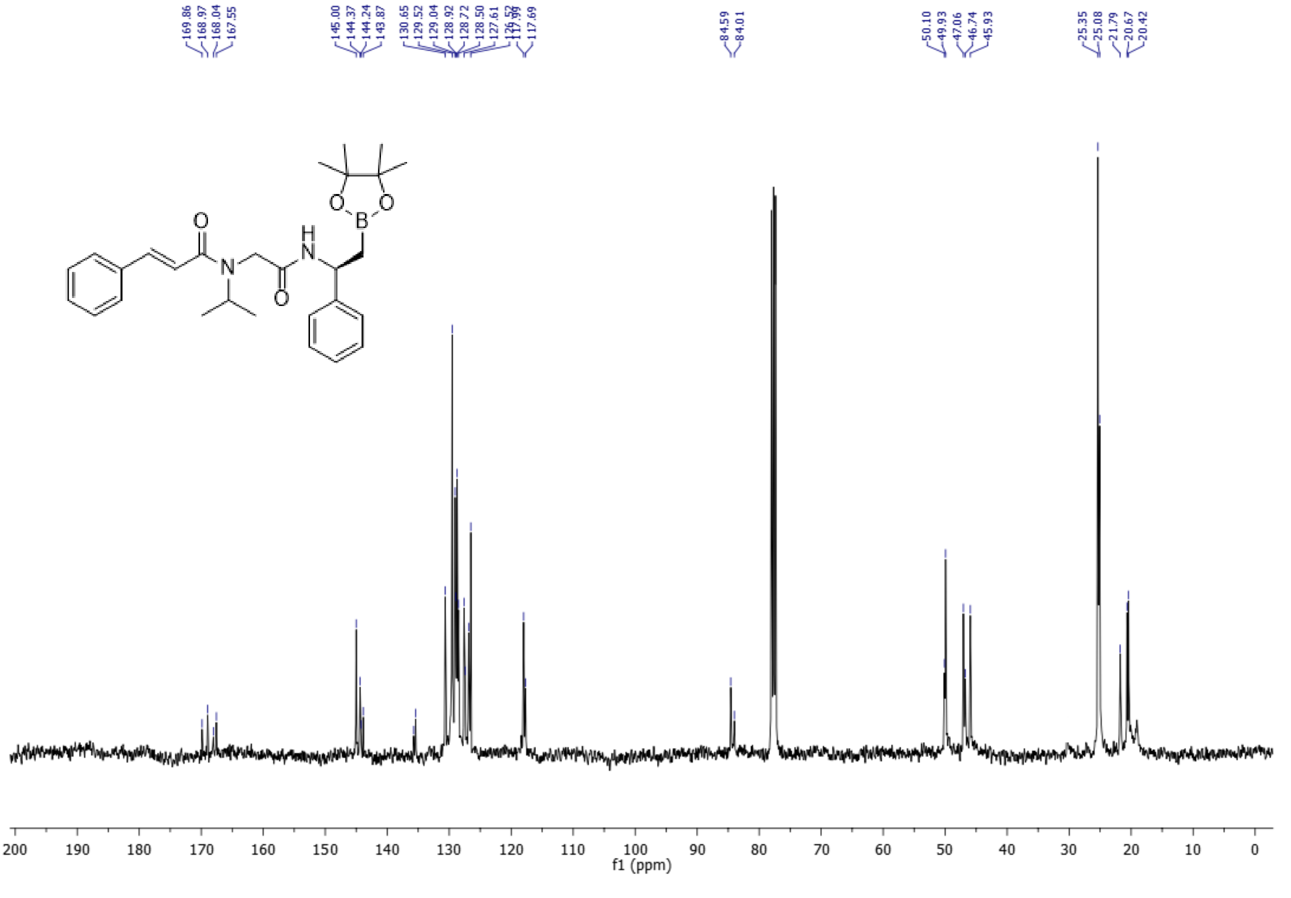

^{11}B NMR (128 MHz, $CDCl_3$) of compound **4a**

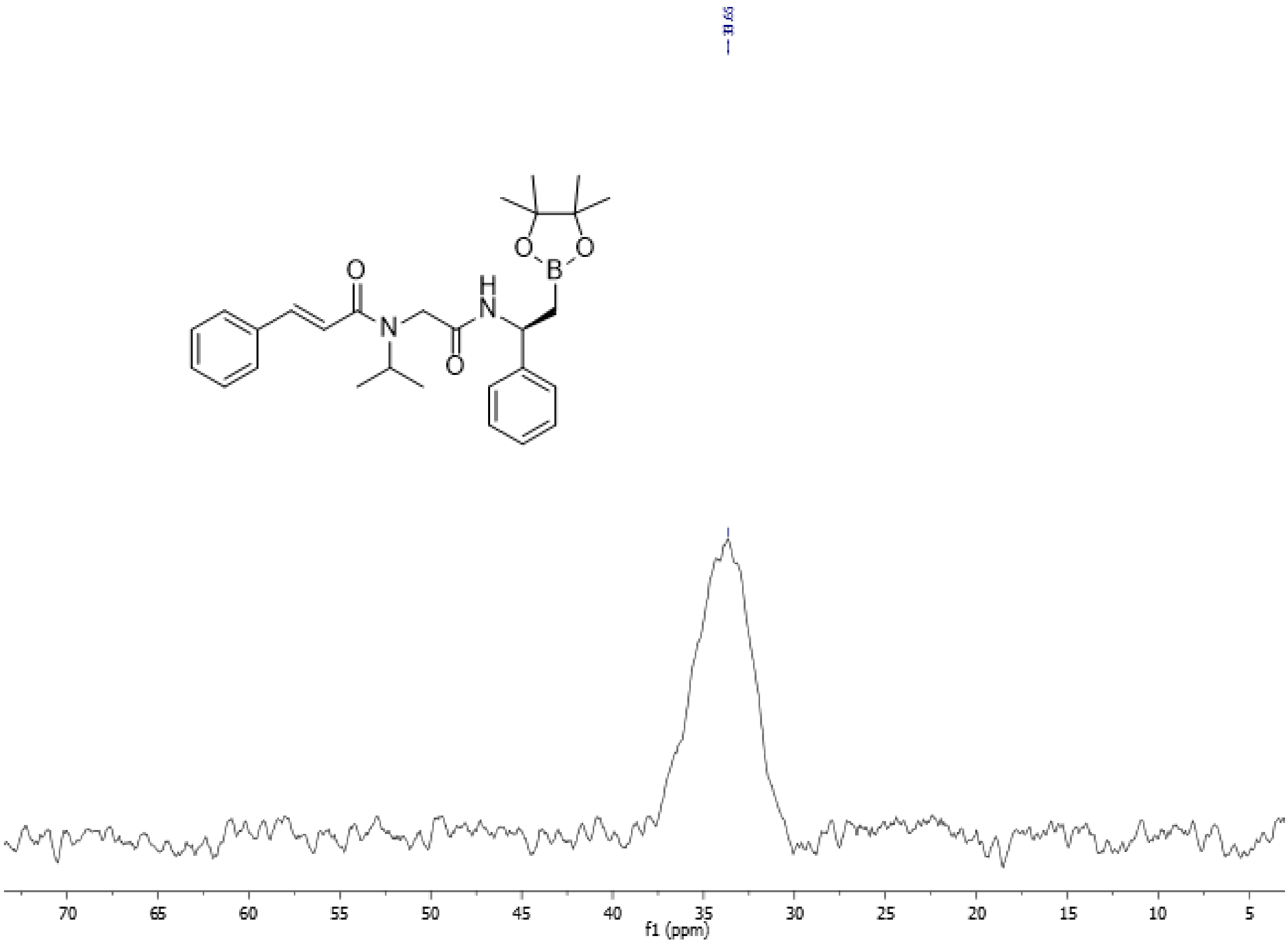

^{1}H NMR (400 MHz, $CDCl_3$) of compound **4b**

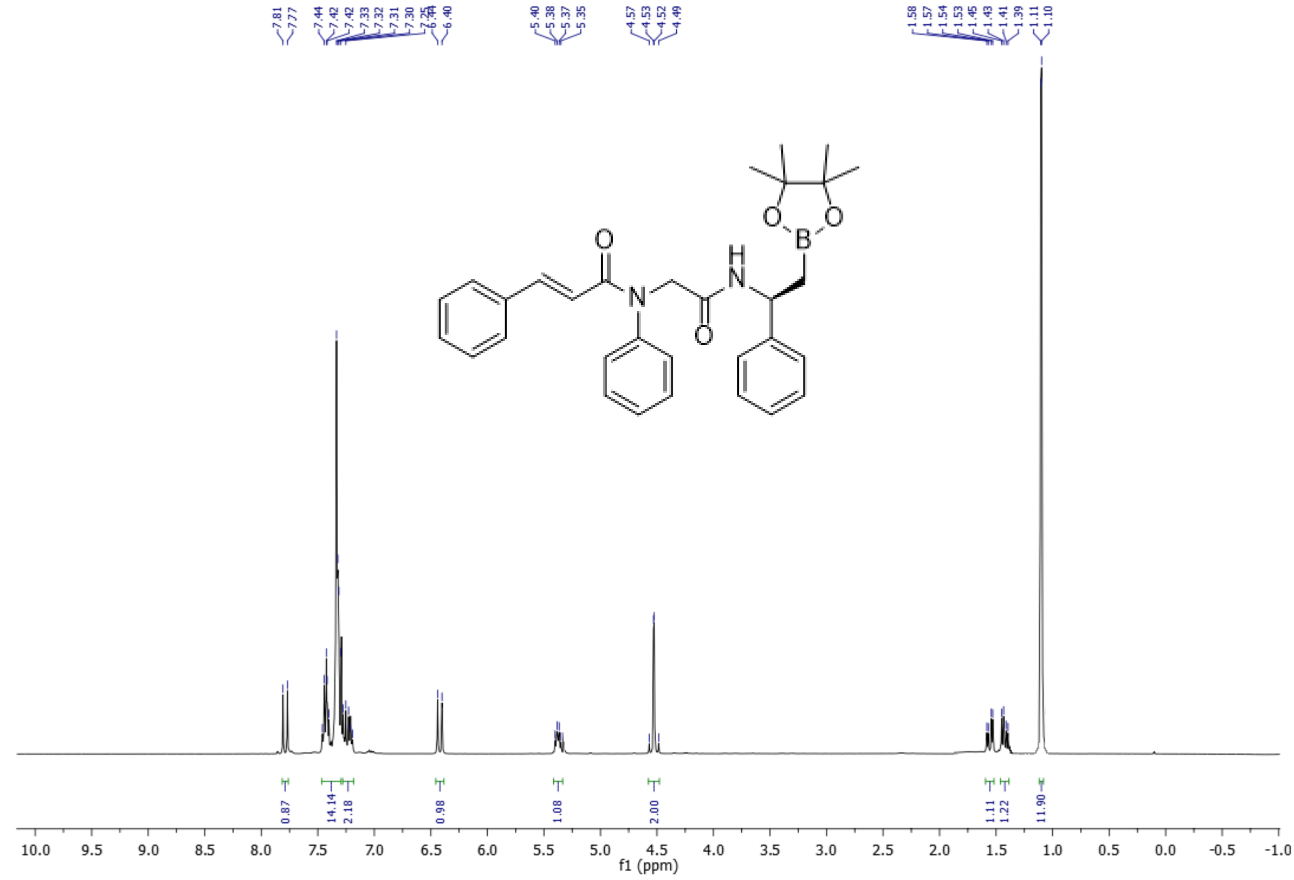

^{13}C NMR (101 MHz, $CDCl_3$) of compound **4b**

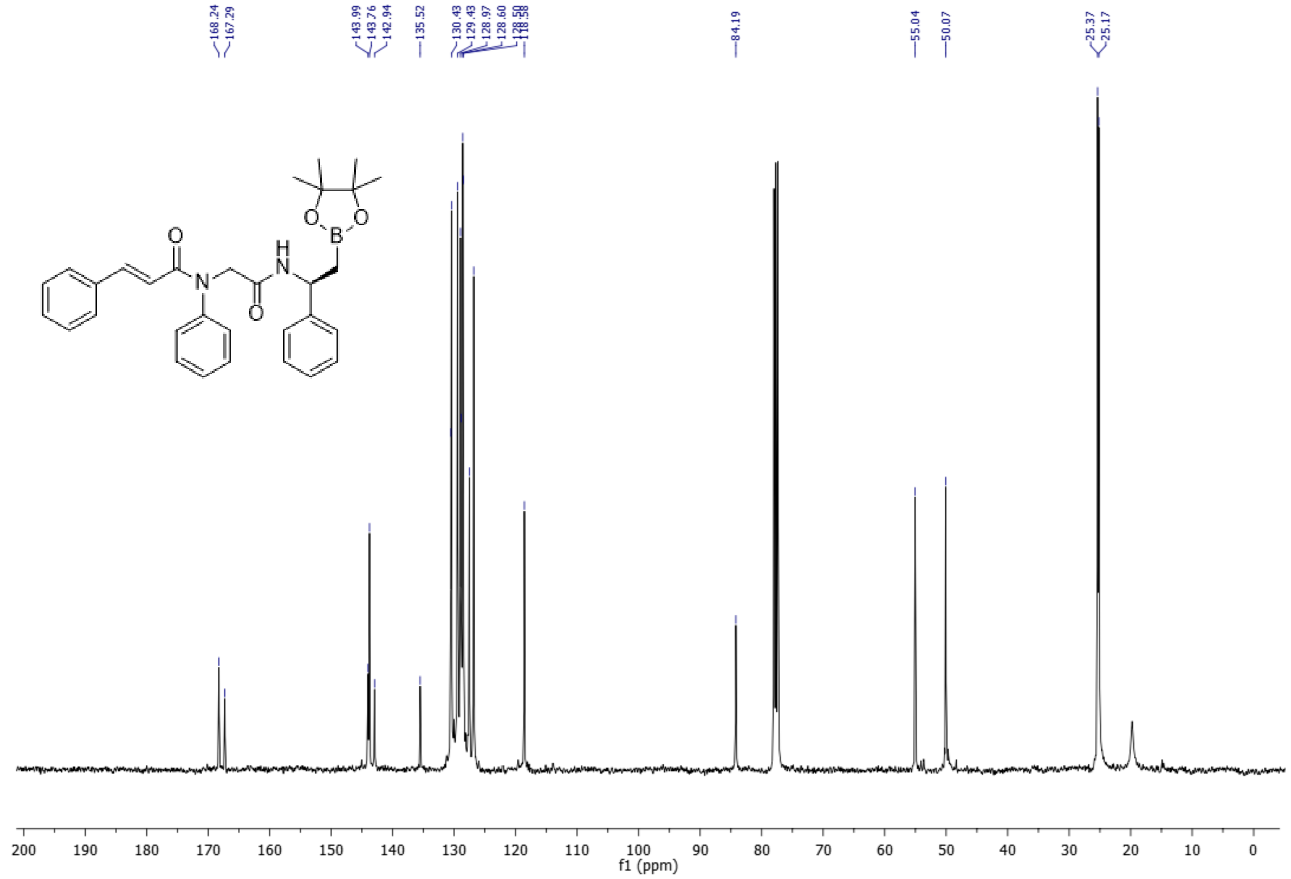

^{11}B NMR (128 MHz, $CDCl_3$) of compound **4b**

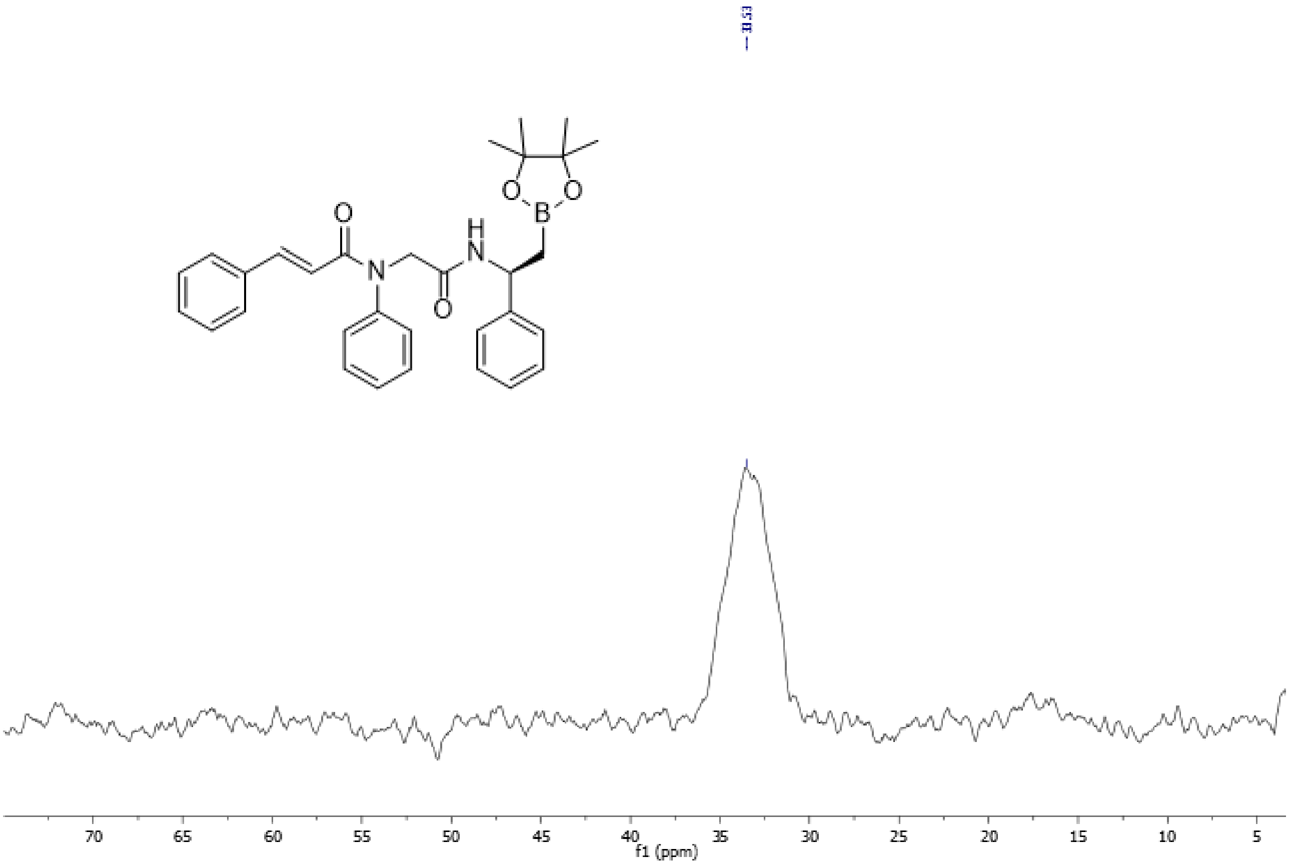

^{1}H NMR (400 MHz, $CDCl_3$) of compound **4c**

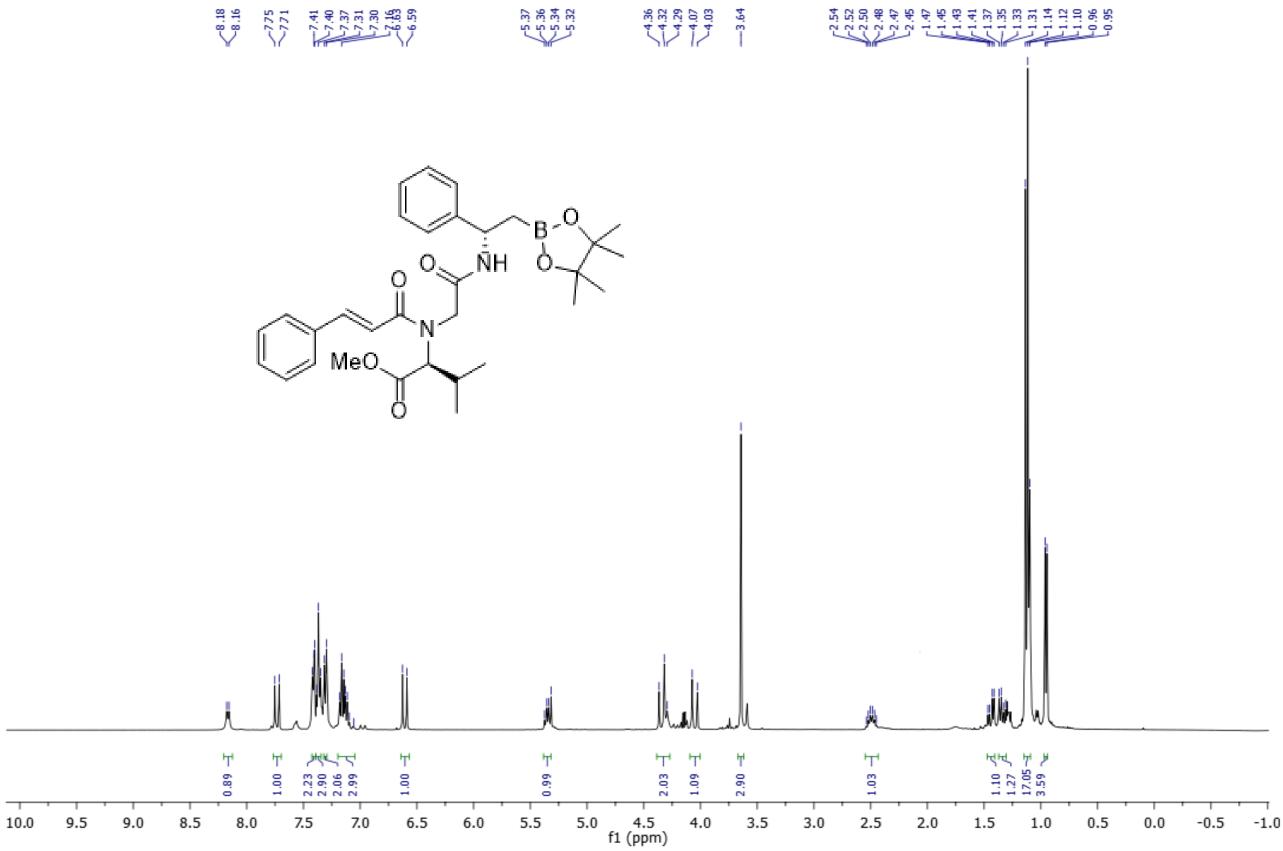

^{13}C NMR (101 MHz, $CDCl_3$) of compound **4c**

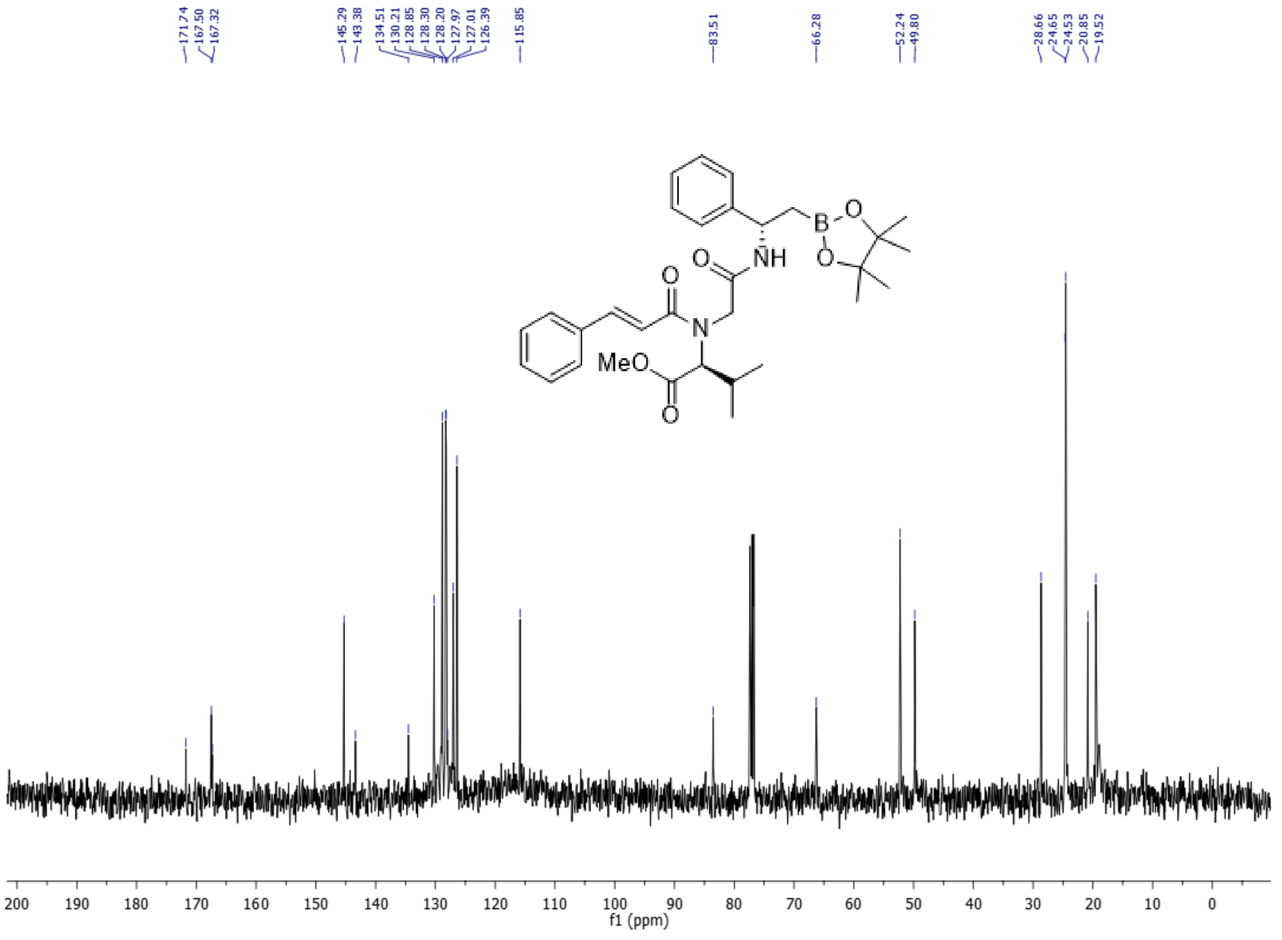

^{11}B NMR (128 MHz, $CDCl_3$) of compound **4c**

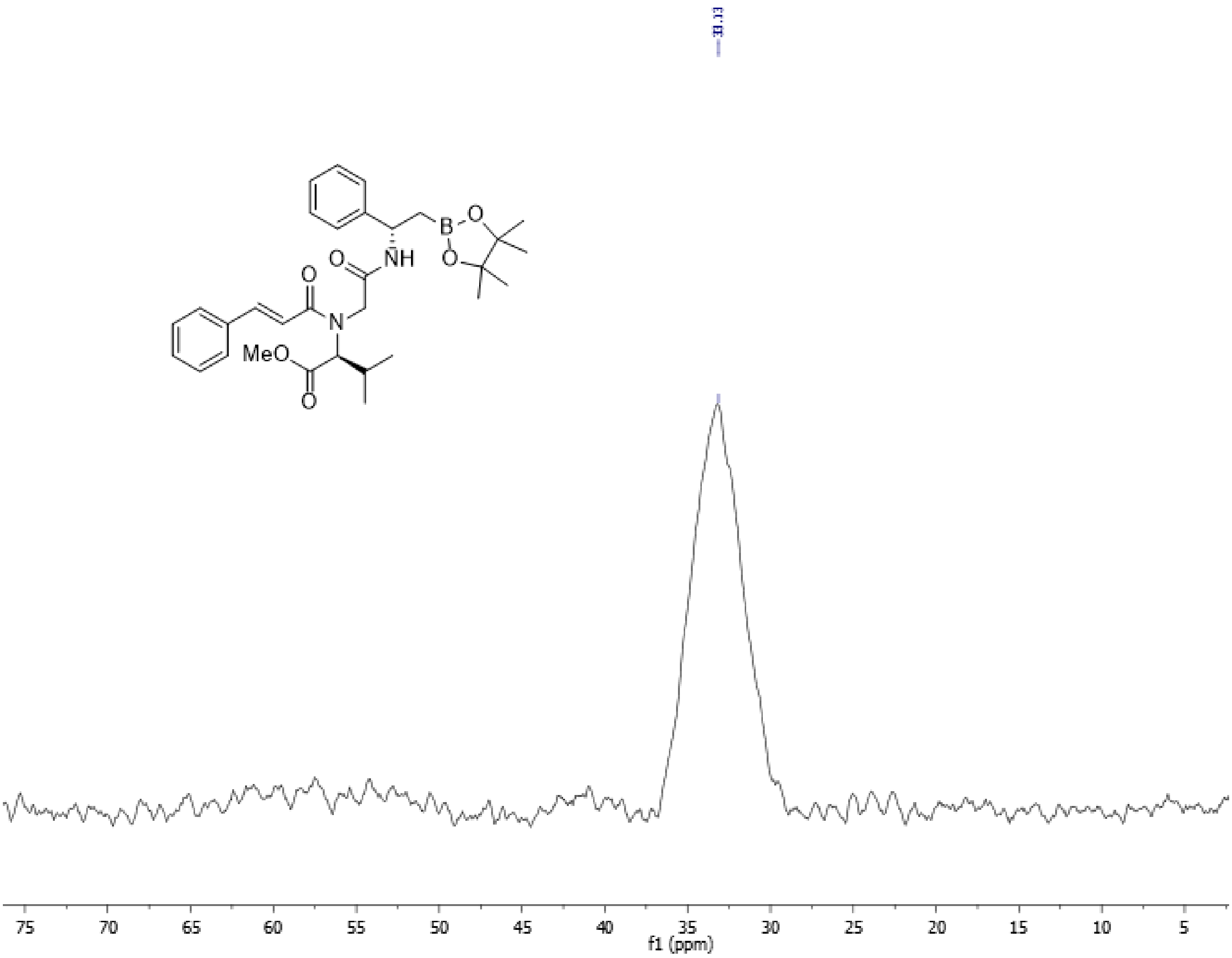

^{1}H NMR (400 MHz, $CDCl_3$, rotamers mixture 55:45) of compound **4d**

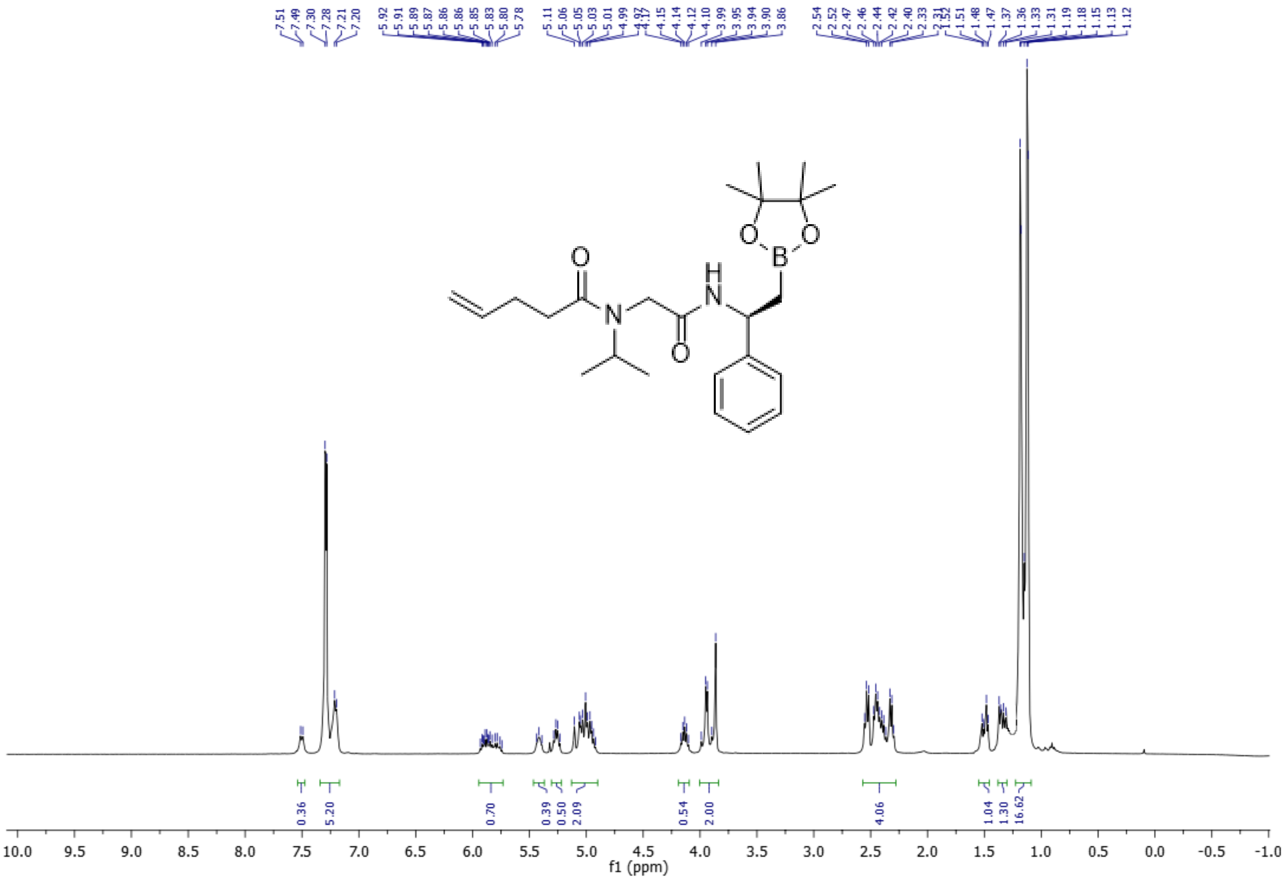

^{13}C NMR (101 MHz, $CDCl_3$) of compound **4d**

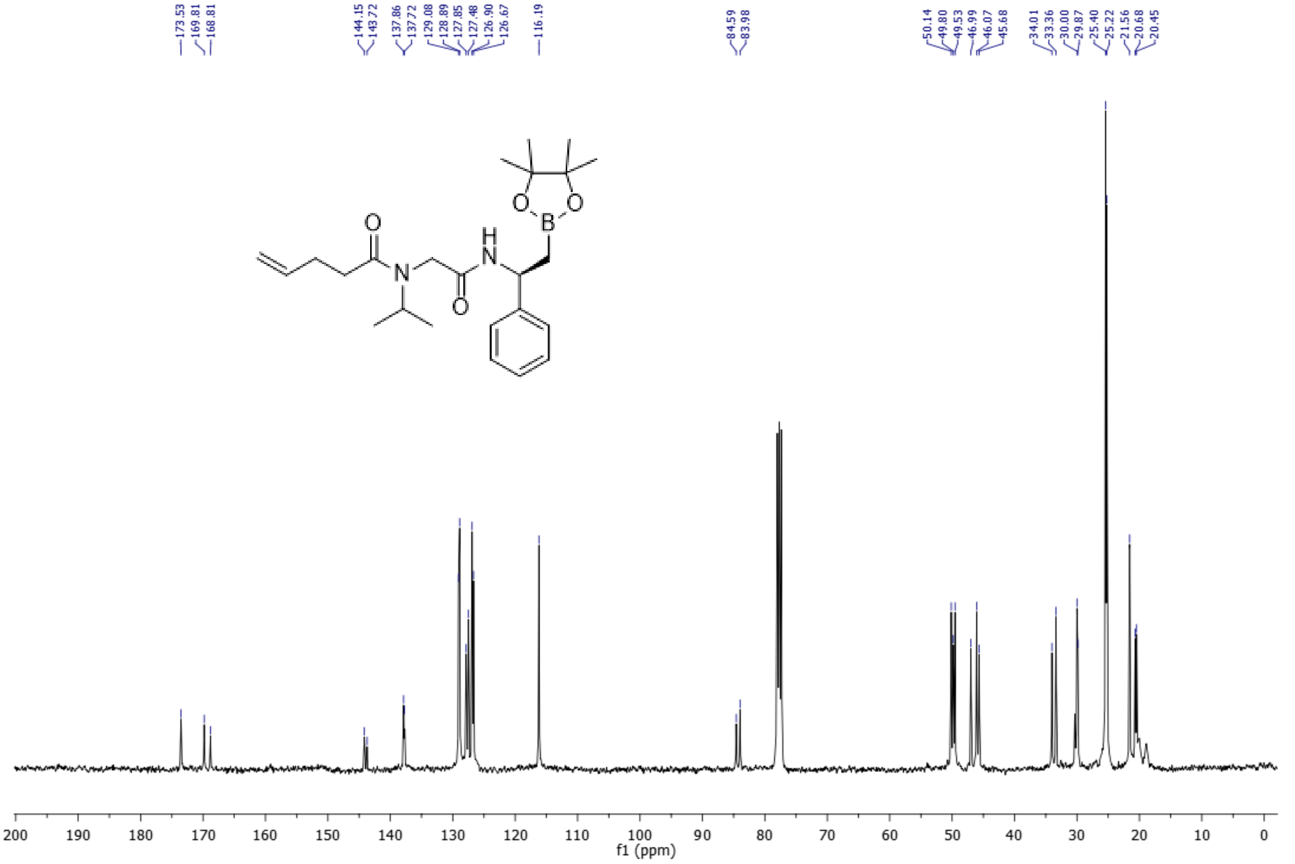

^{11}B NMR (128 MHz, $CDCl_3$) of compound **4d**

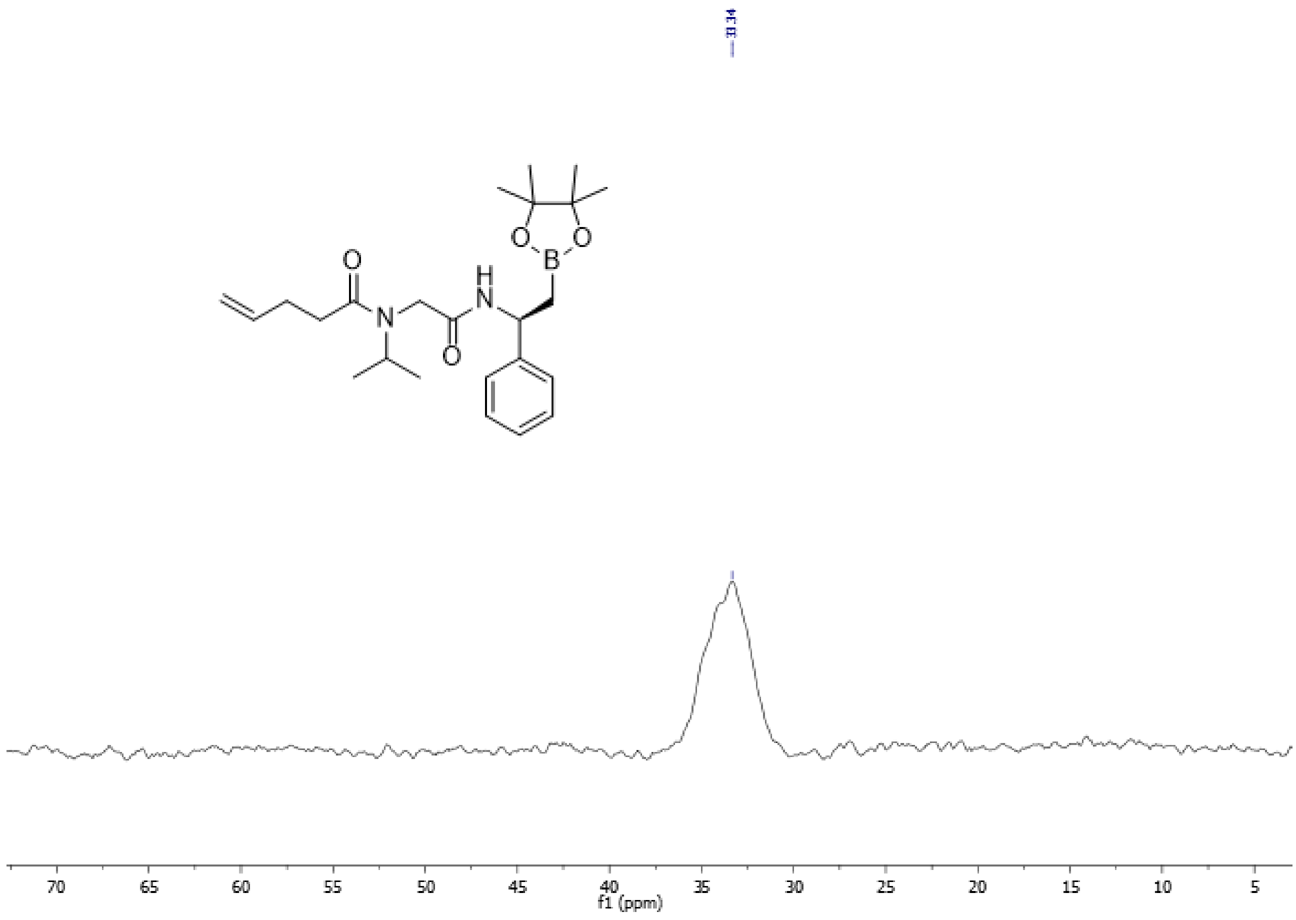

^{1}H NMR (400 MHz, $CDCl_3$) of compound **4e**

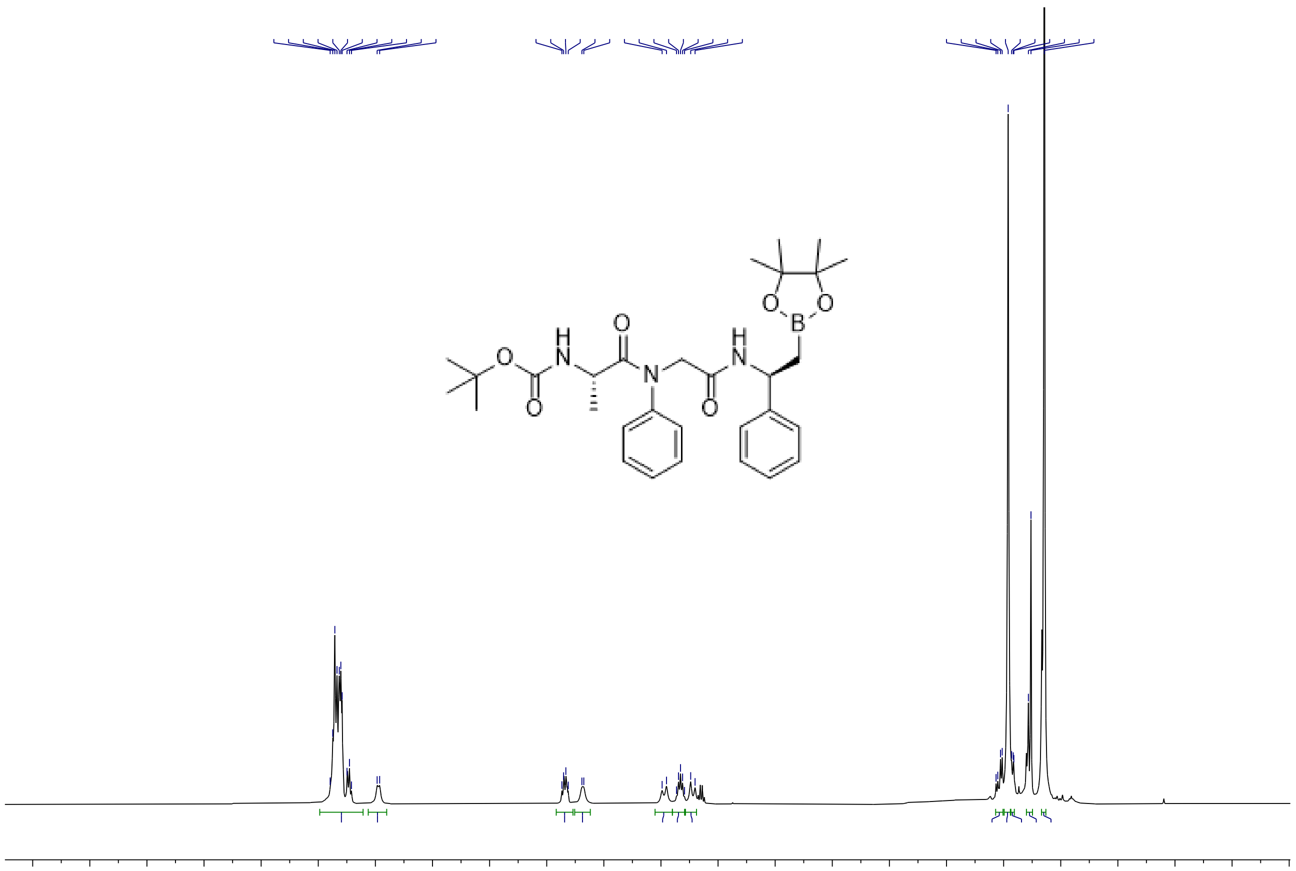

^{13}C NMR (101 MHz, $CDCl_3$) of compound **4e**

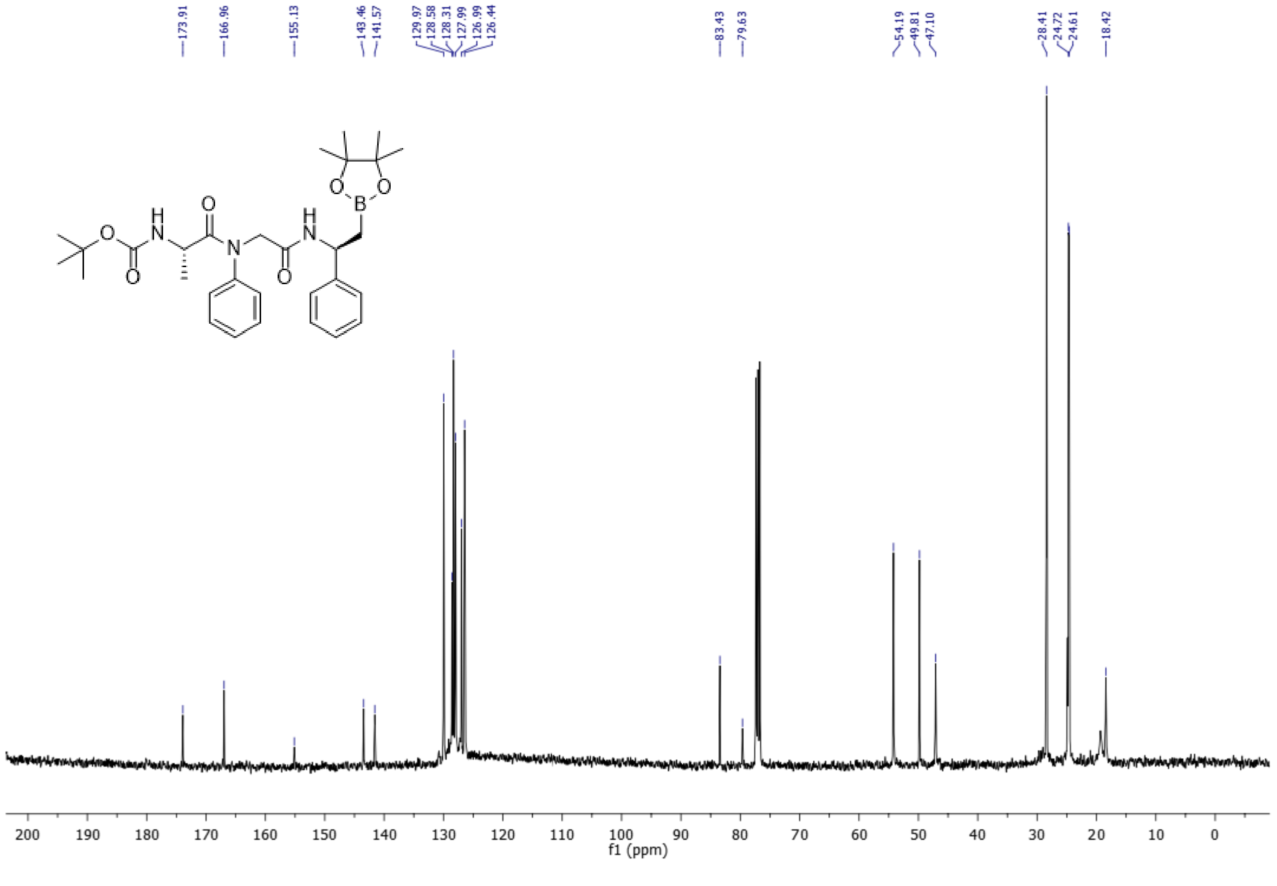

^{11}B NMR (128 MHz, $CDCl_3$) of compound **4e**

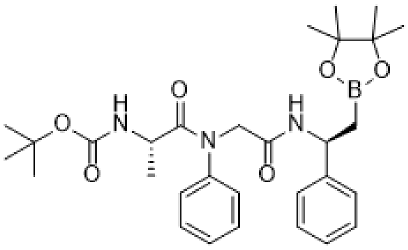

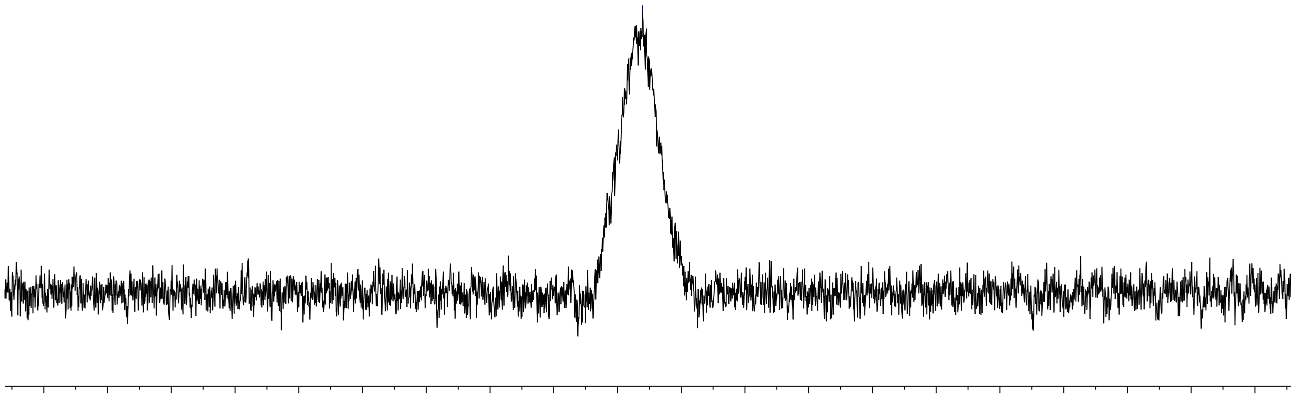

^{1}H NMR (400 MHz, $CDCl_3$) of compound **4fa**

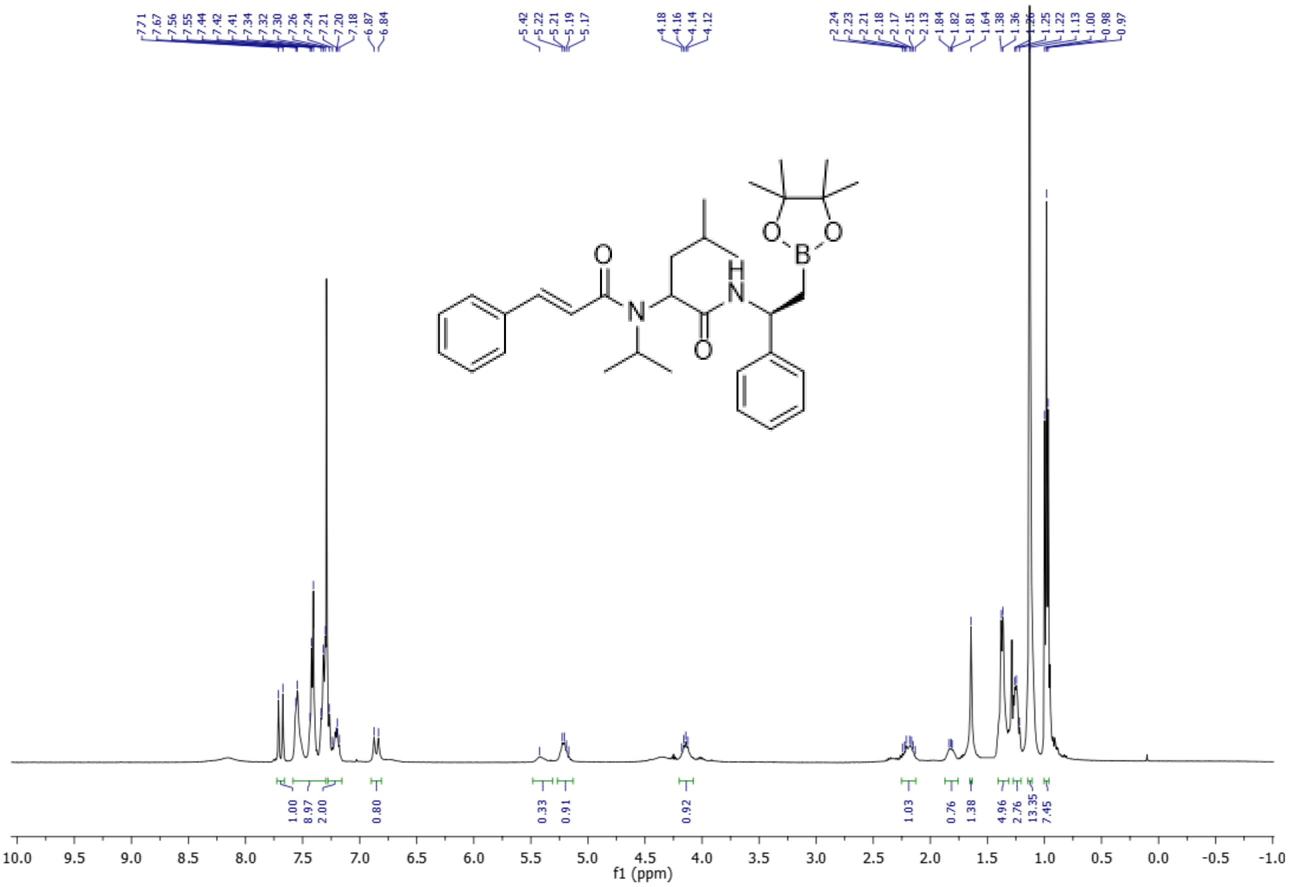

^{13}C NMR (101 MHz, $CDCl_3$) **4fa**

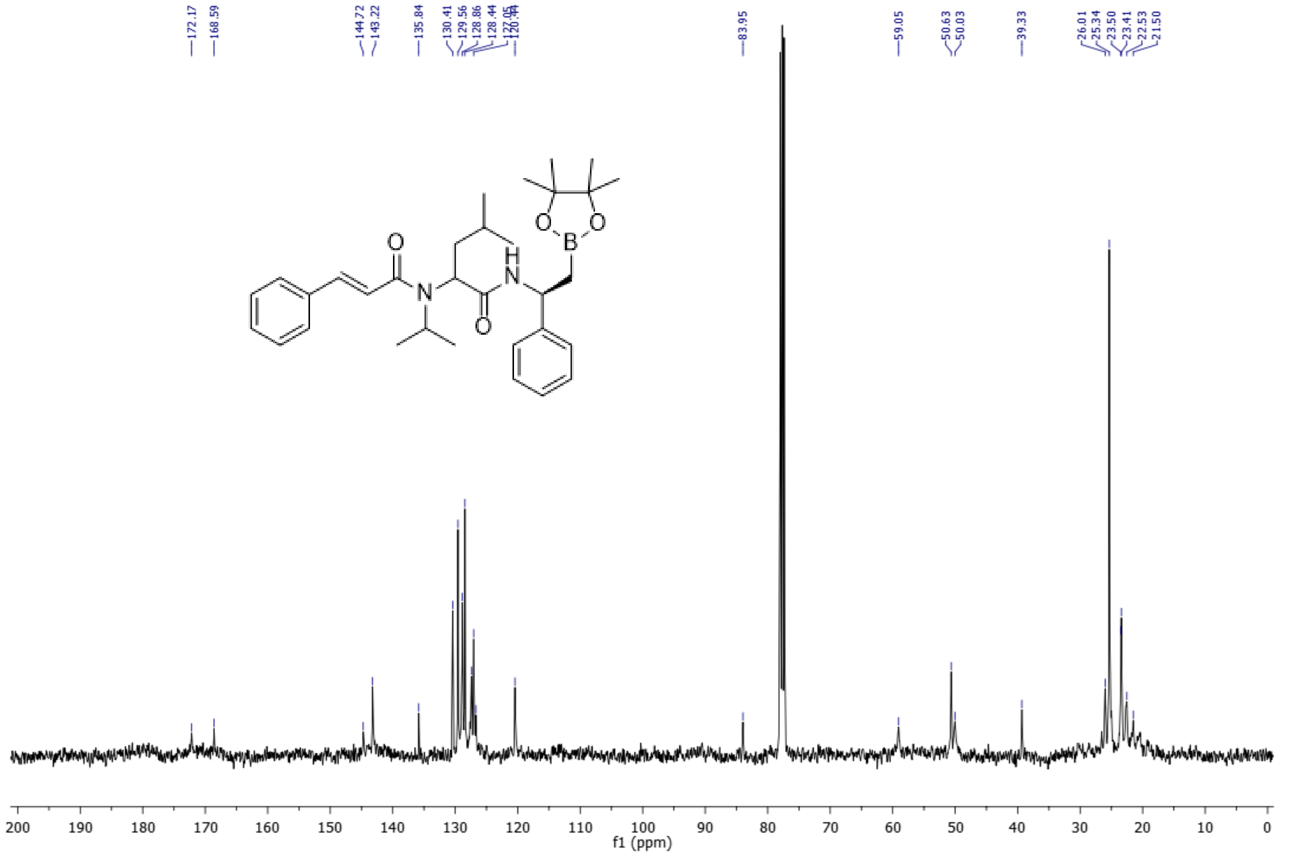

^{11}B NMR (128 MHz, $CDCl_3$) **4fa**

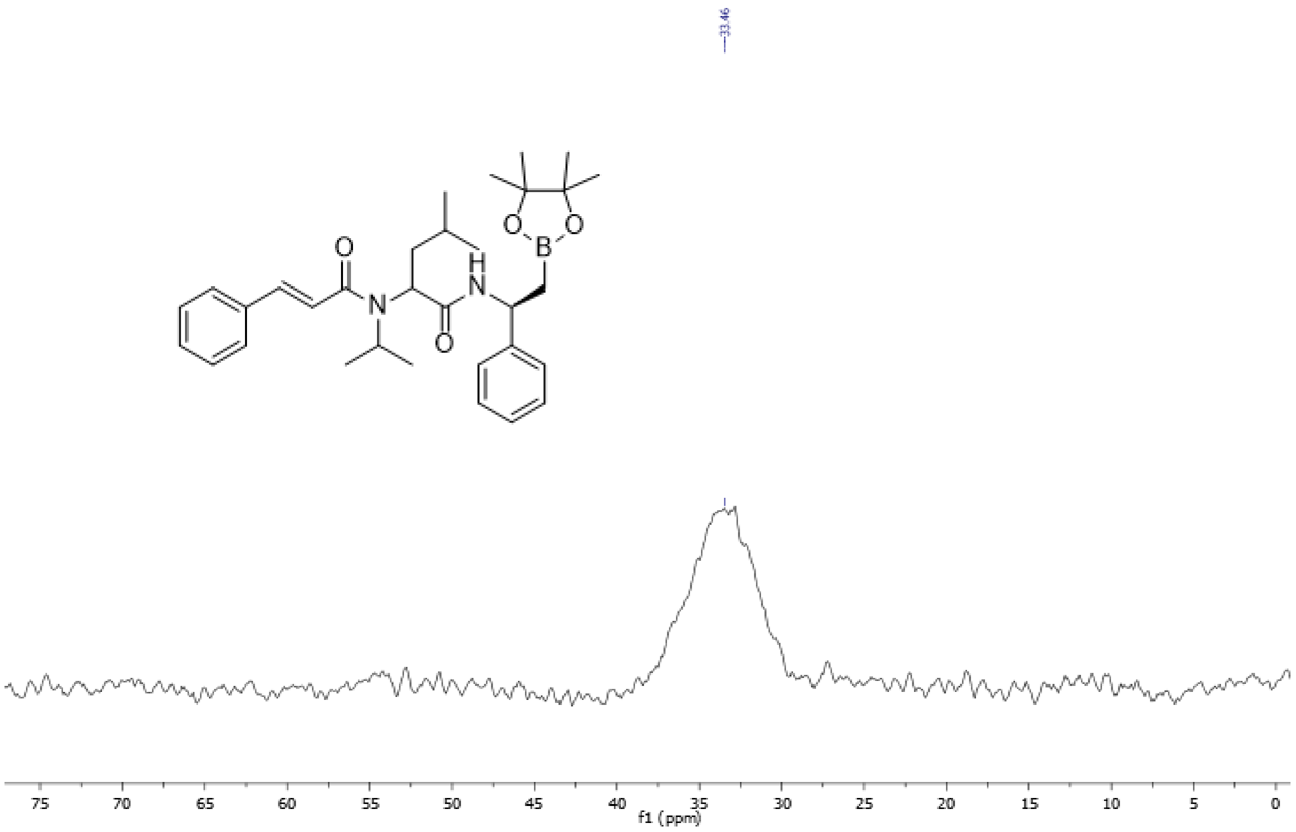

^{1}H NMR (400 MHz, $CDCl_3$) of compound **4fb**

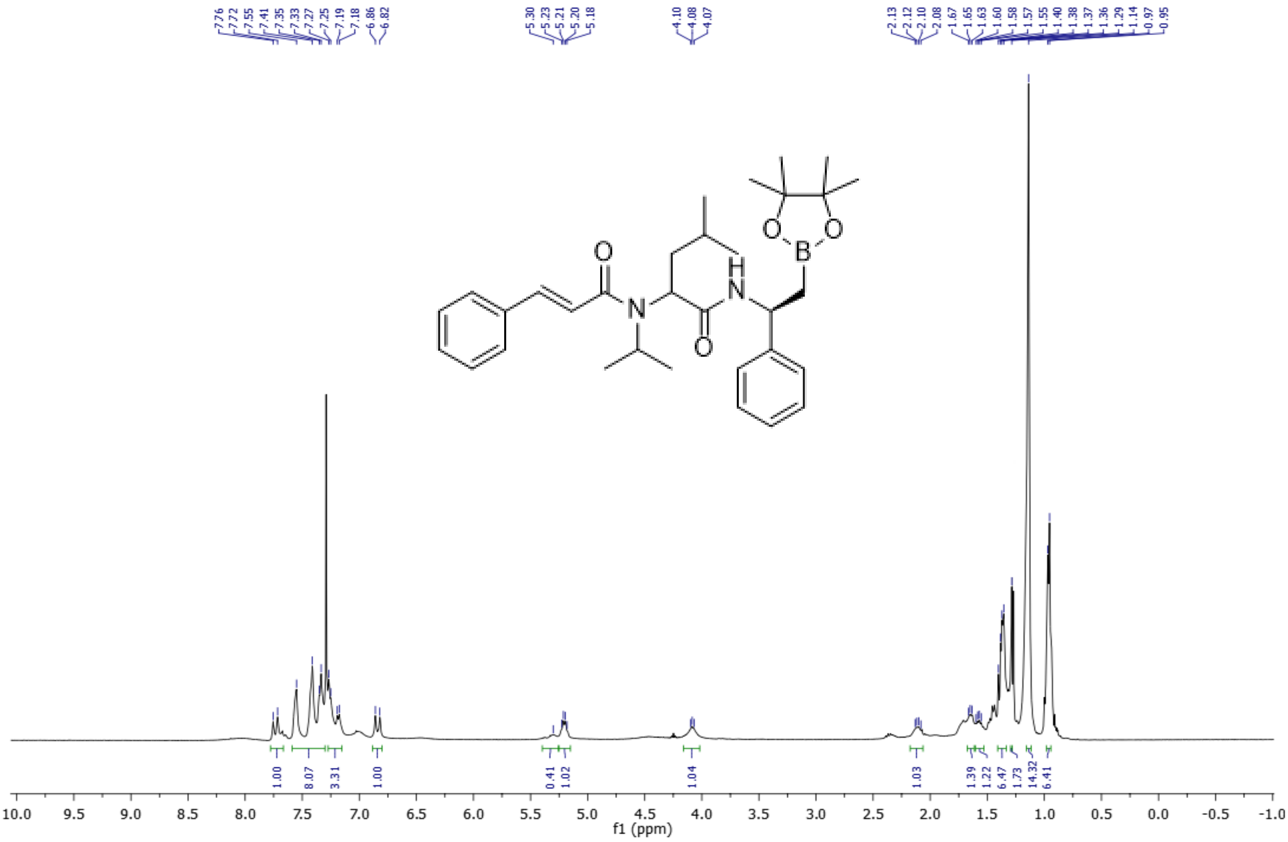

^{13}C NMR (101 MHz, $CDCl_3$) **4fb**

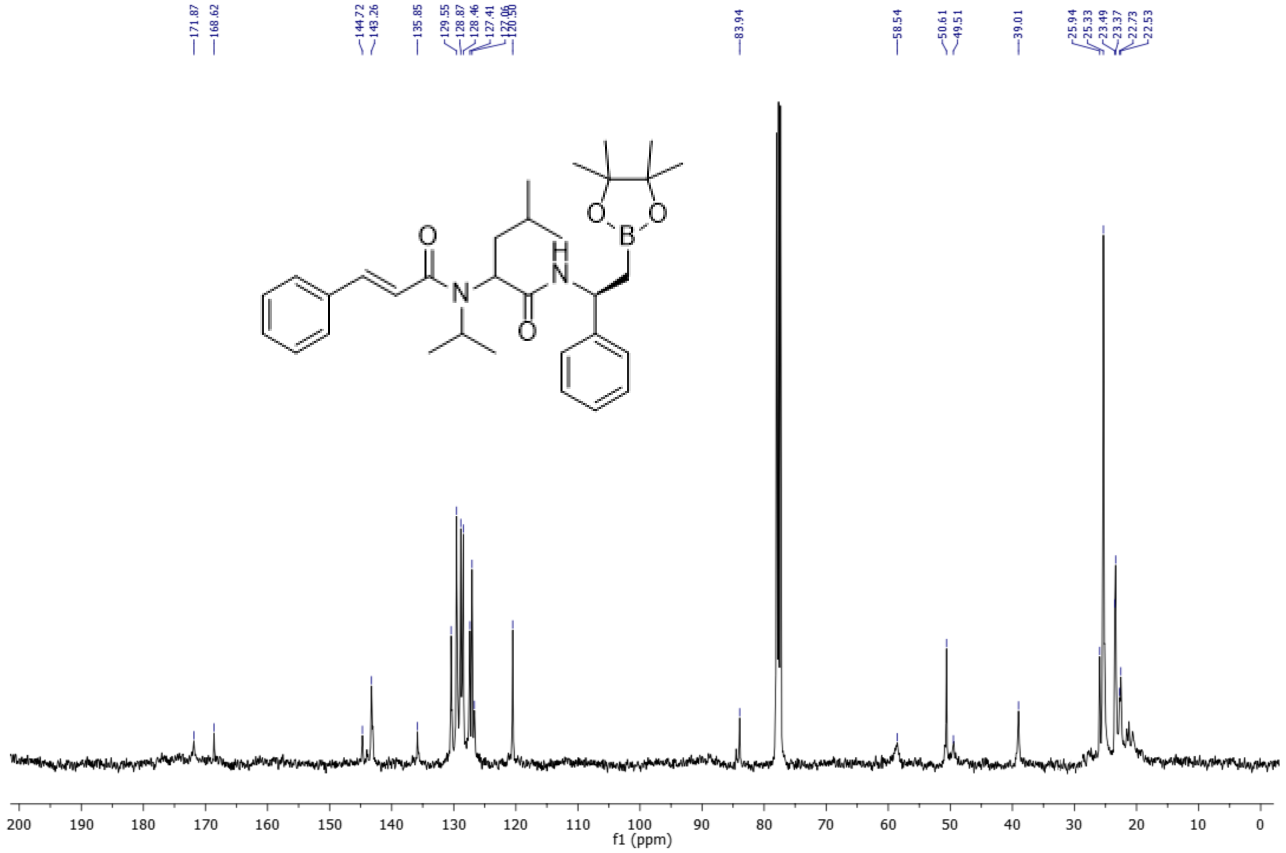

^{11}B NMR (128 MHz, $CDCl_3$) **4fb**

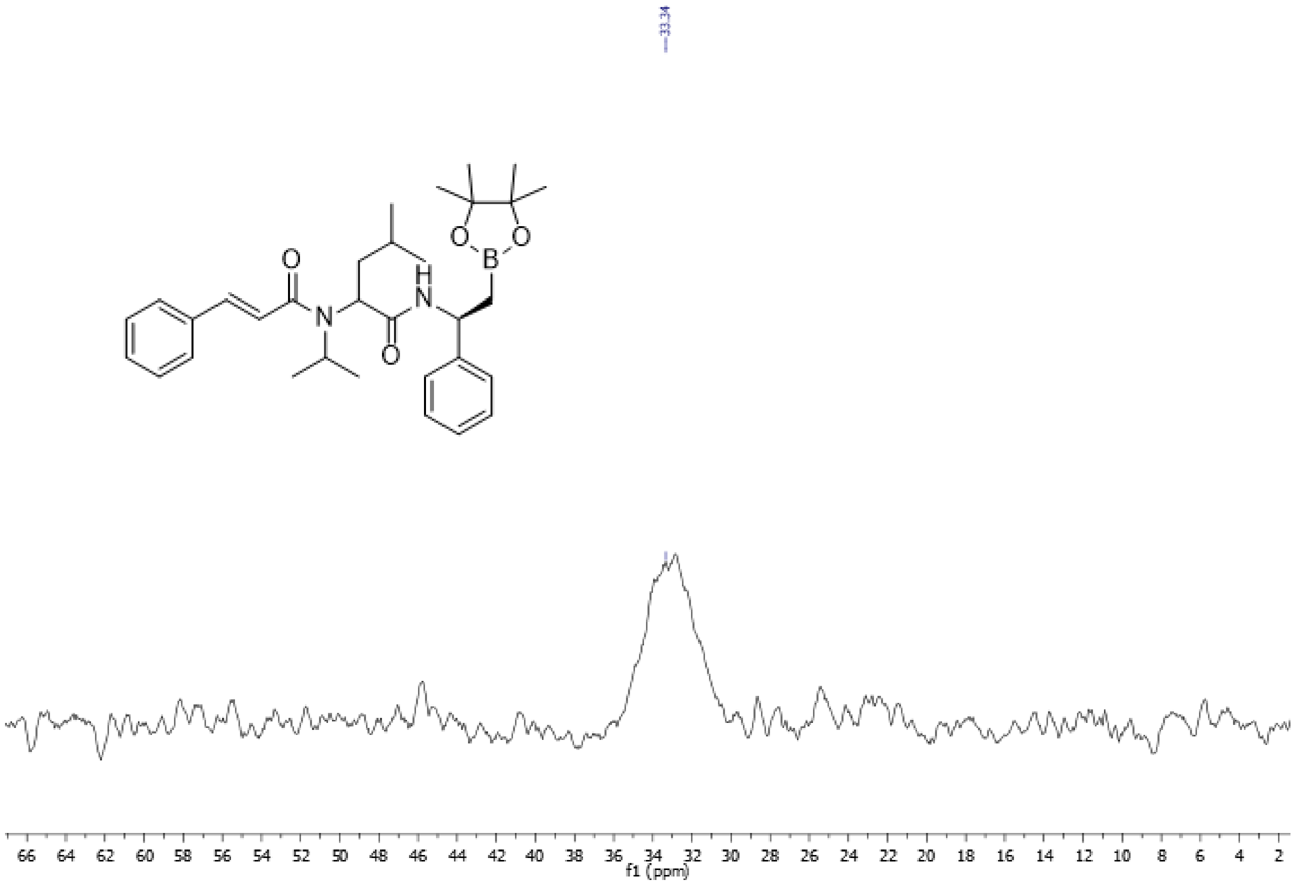

^{1}H NMR (400 MHz, $CDCl_3$, rotamers mixture 70:30) of compound **4g**

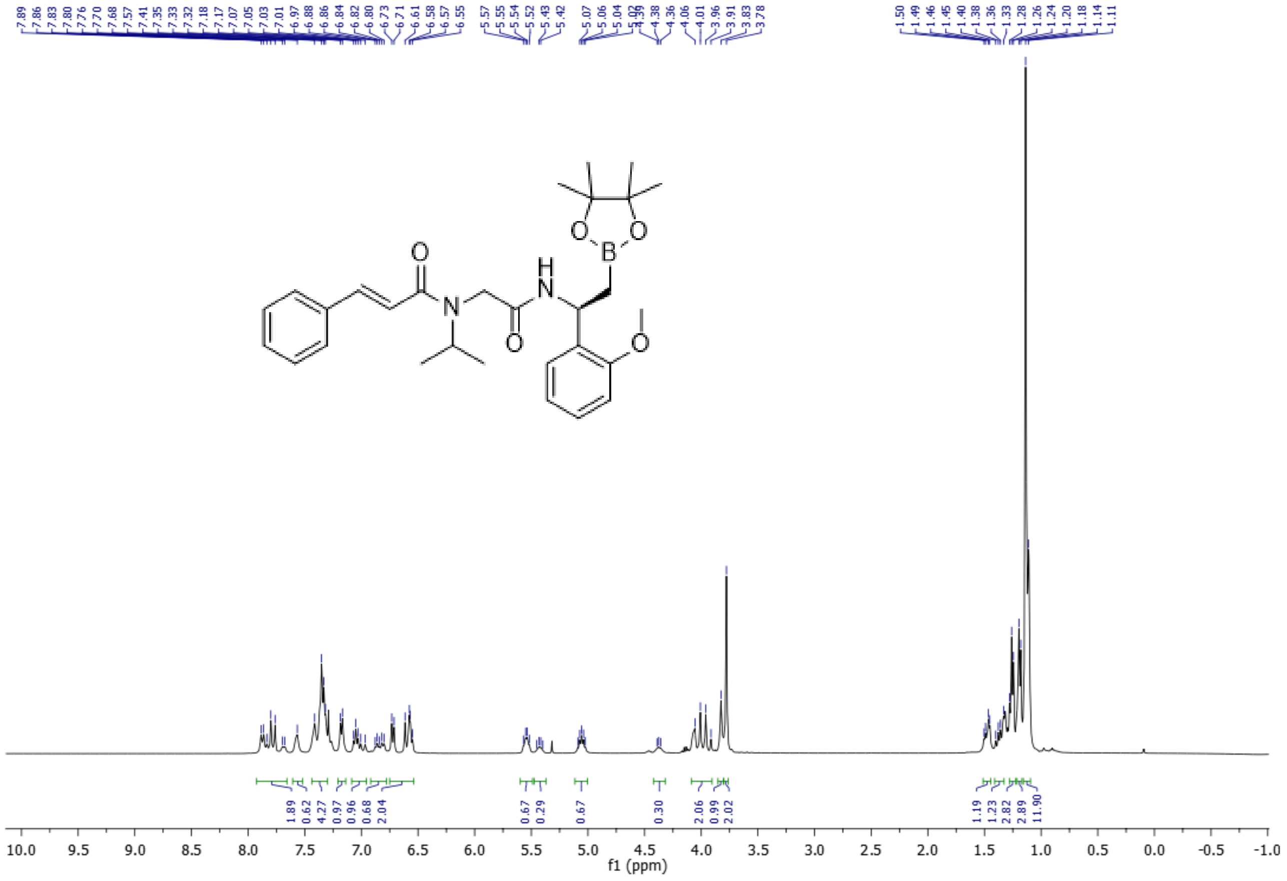

^{13}C NMR (101 MHz, $CDCl_3$, rotamers mixture 70:30) of compound **4g**

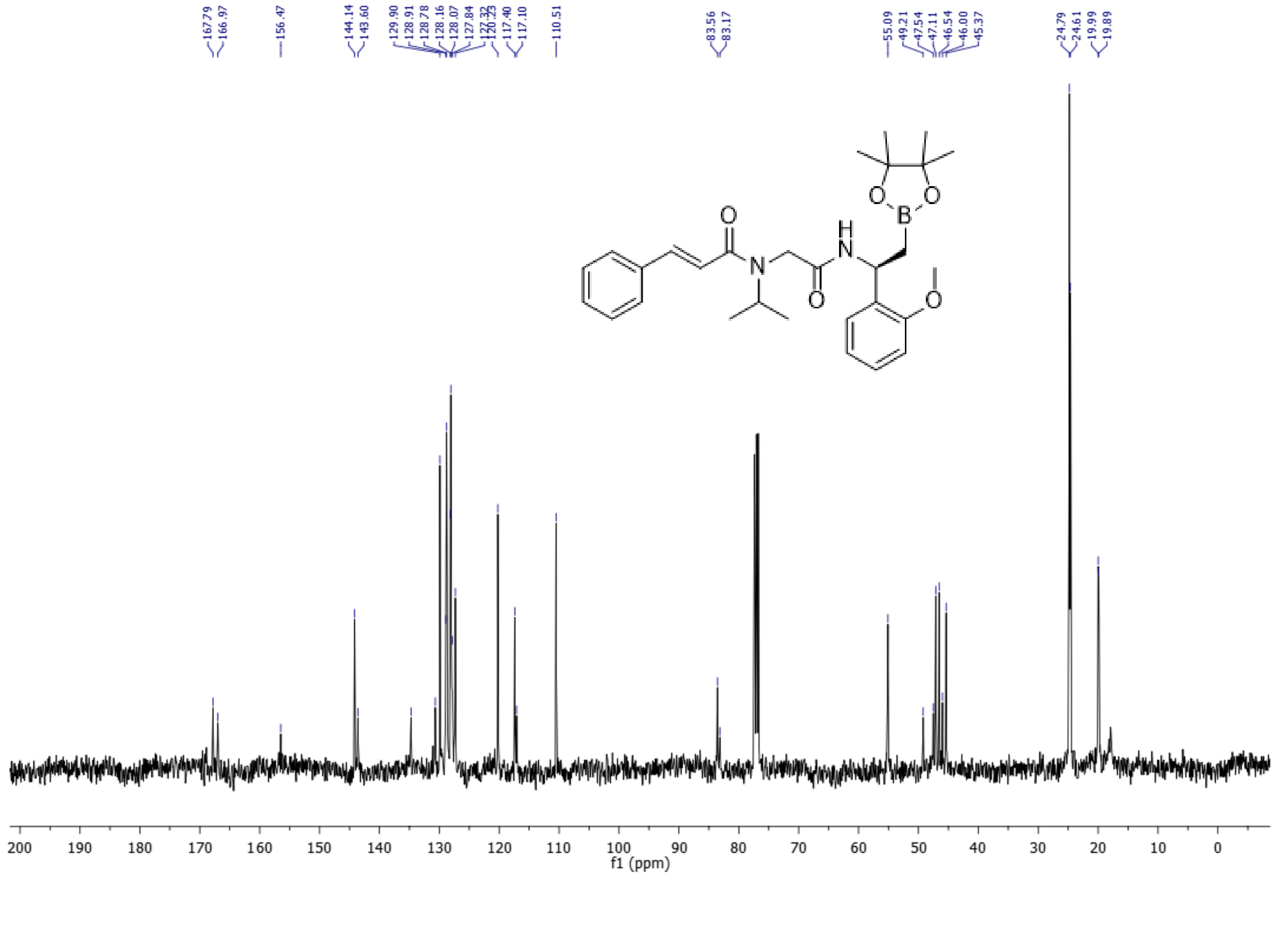

^{11}B NMR (128 MHz, $CDCl_3$) of compound **4g**

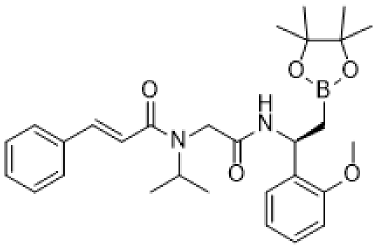

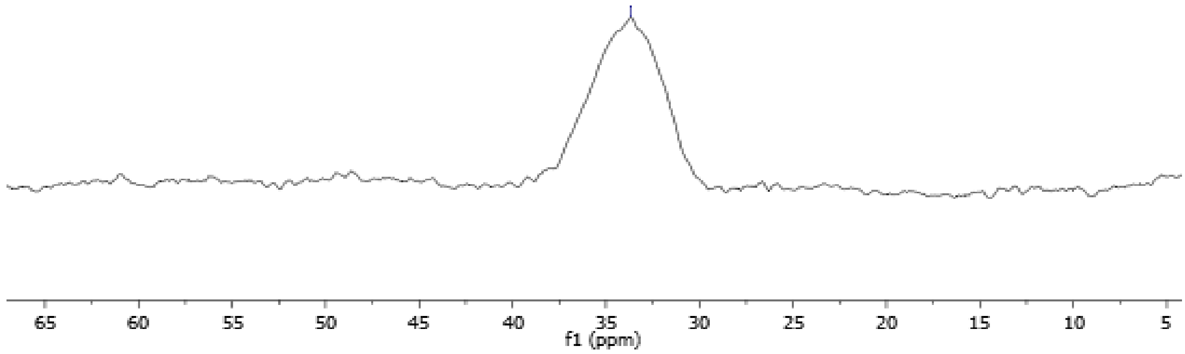

^{1}H NMR (400 MHz, $CDCl_3$, rotamers mixture 55:45) of compound **4h**

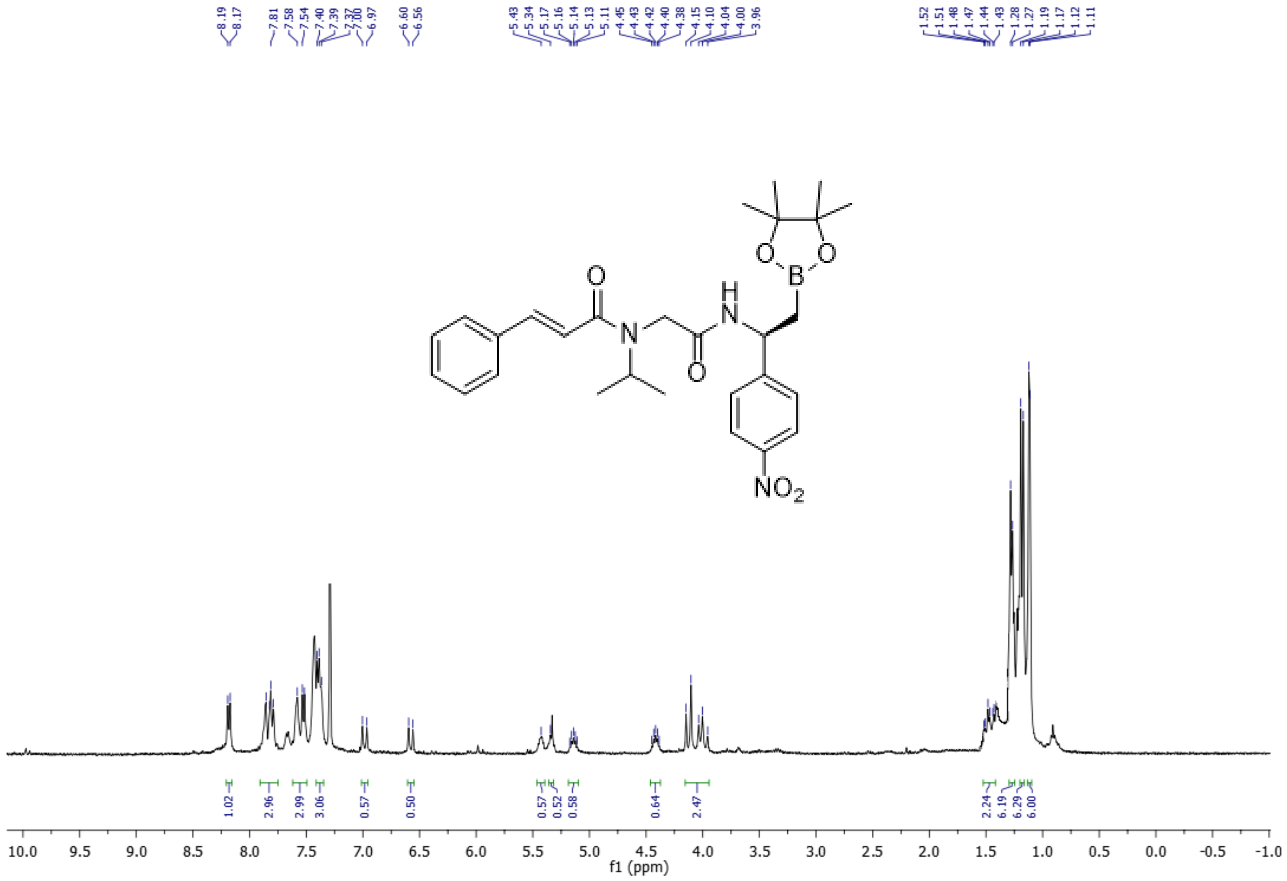

^{13}C NMR (101 MHz, $CDCl_3$, rotamers mixture 55:45) of compound **4h**

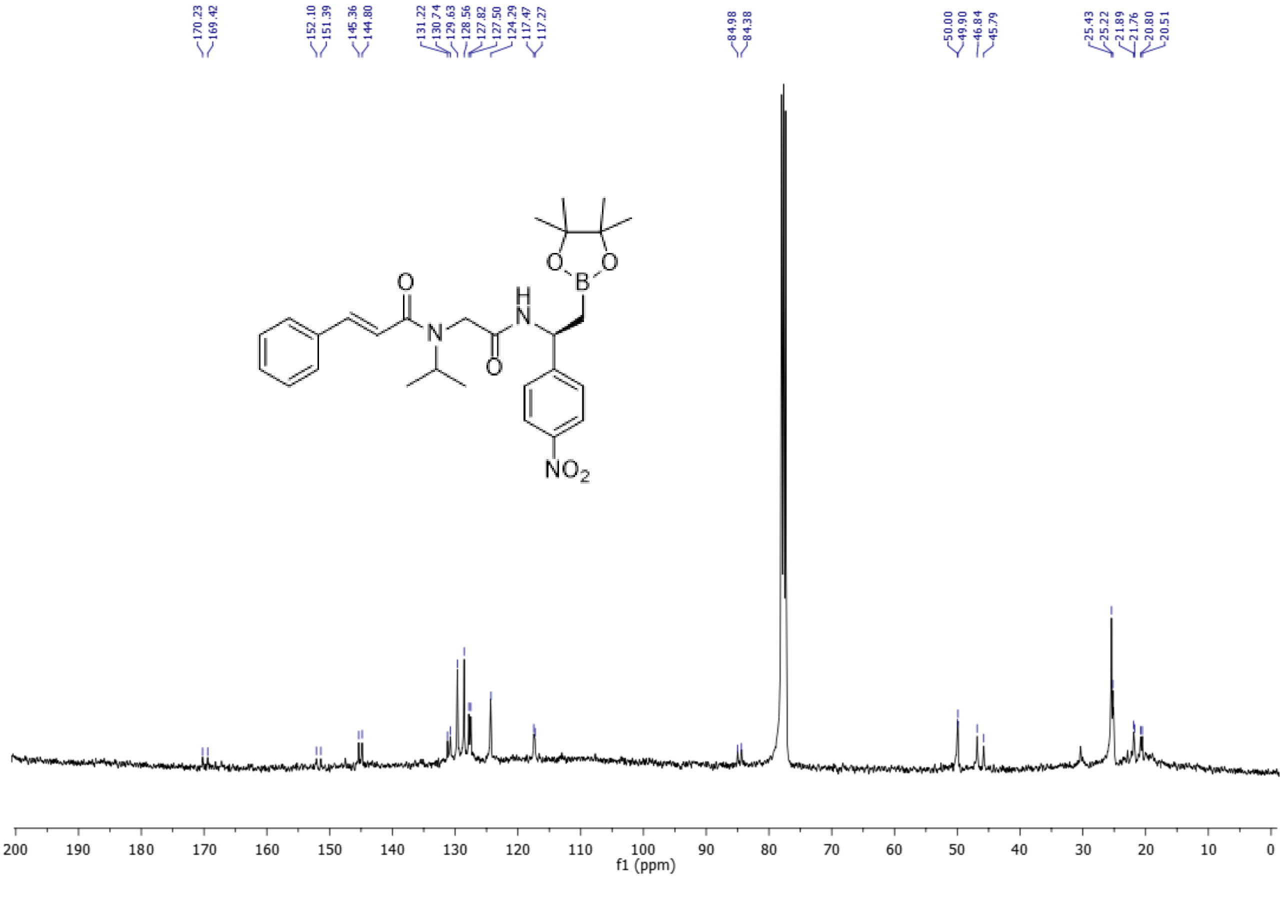

^{11}B NMR (128 MHz, $CDCl_3$) of compound **4h**

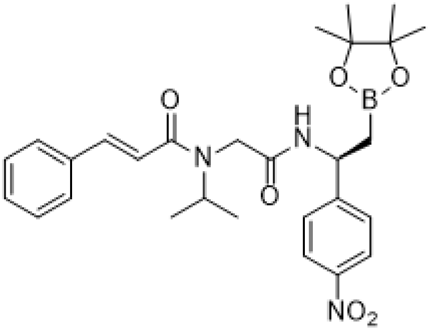

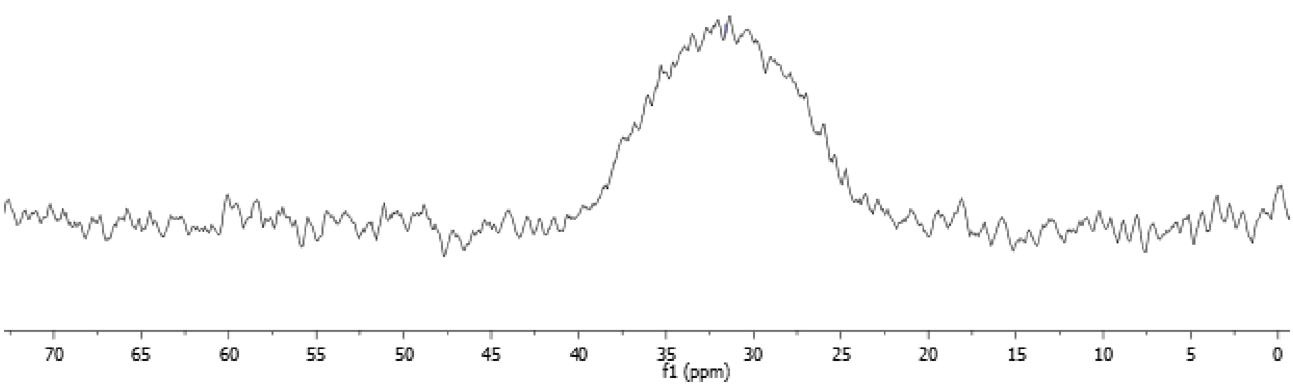

^{1}H NMR (400 MHz, $CDCl_3$, rotamers mixture 60:40) of compound **4i**

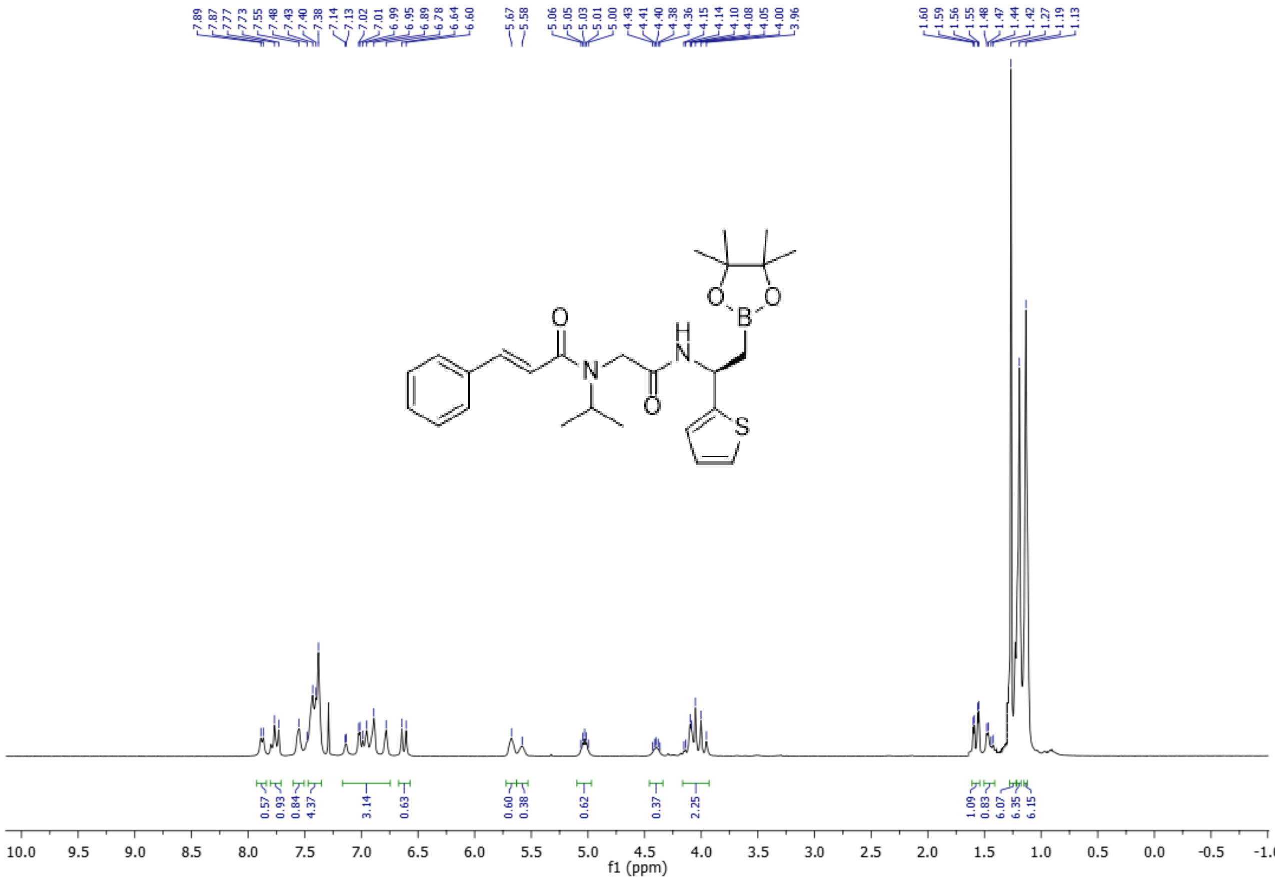

^{13}C NMR (101 MHz, $CDCl_3$, rotamers mixture 60:40) of compound **4i**

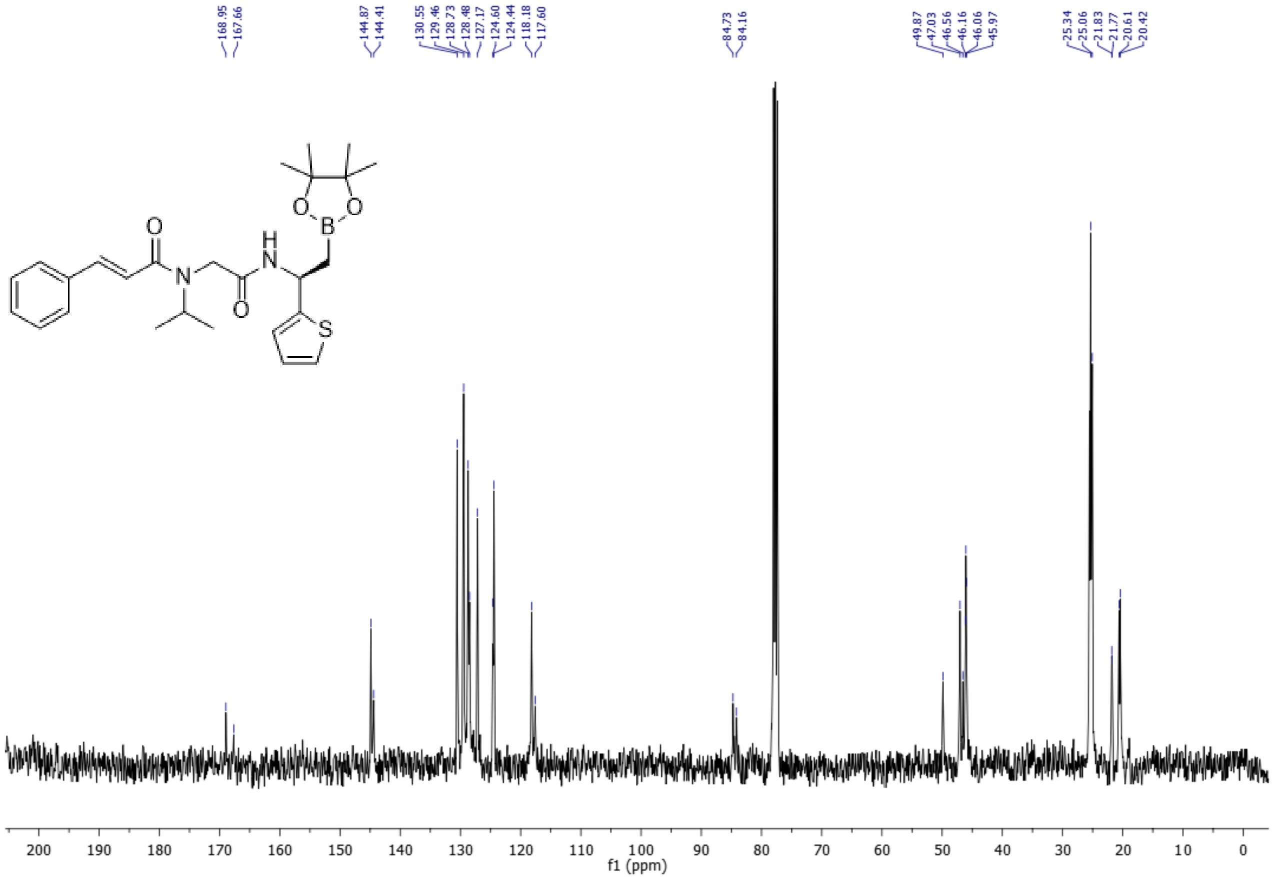

^{11}B NMR (128 MHz, $CDCl_3$) of compound **4i**

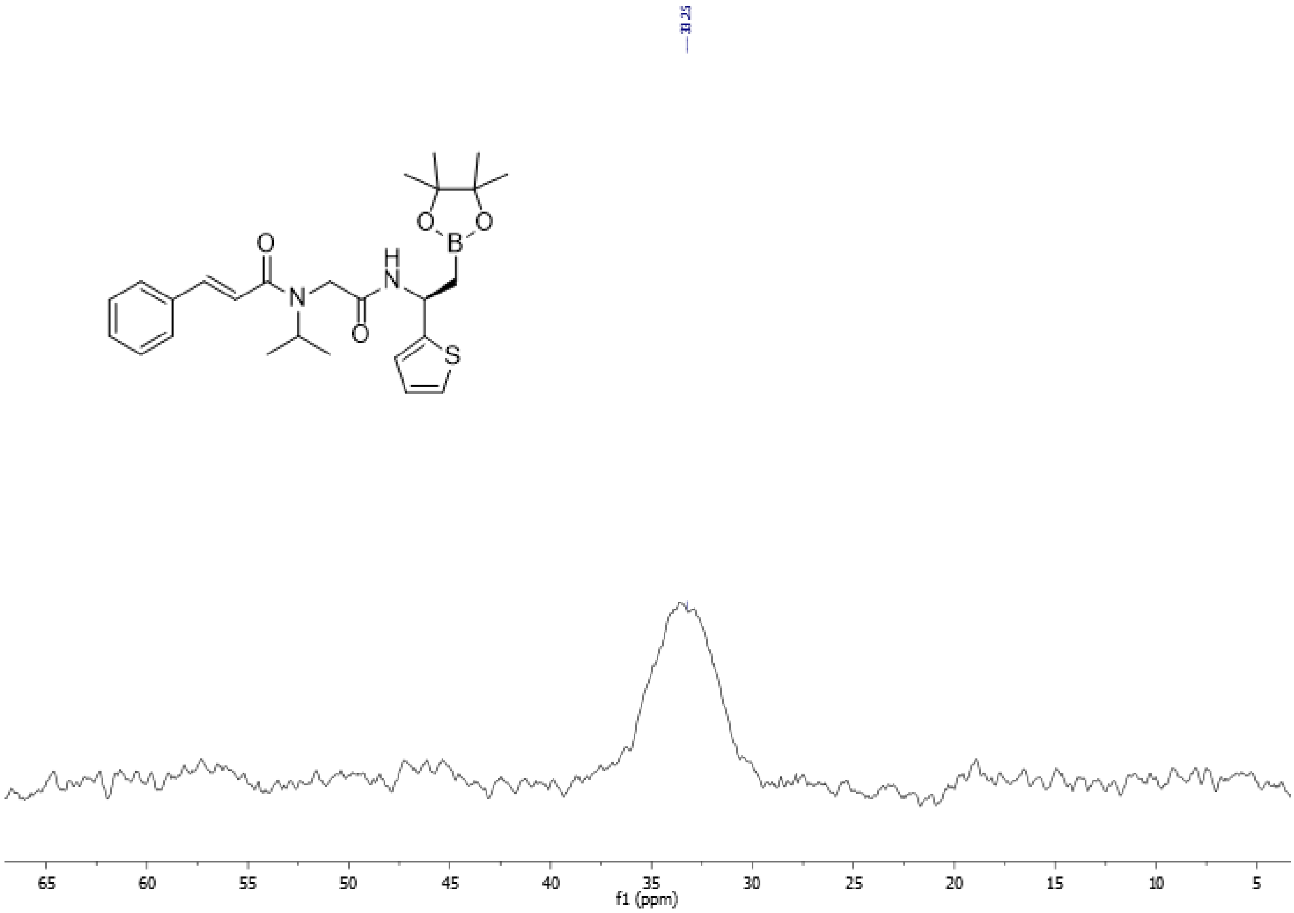

^{1}H NMR (400 MHz, $CDCl_3$, rotamers mixture 65:35) of compound **4j**

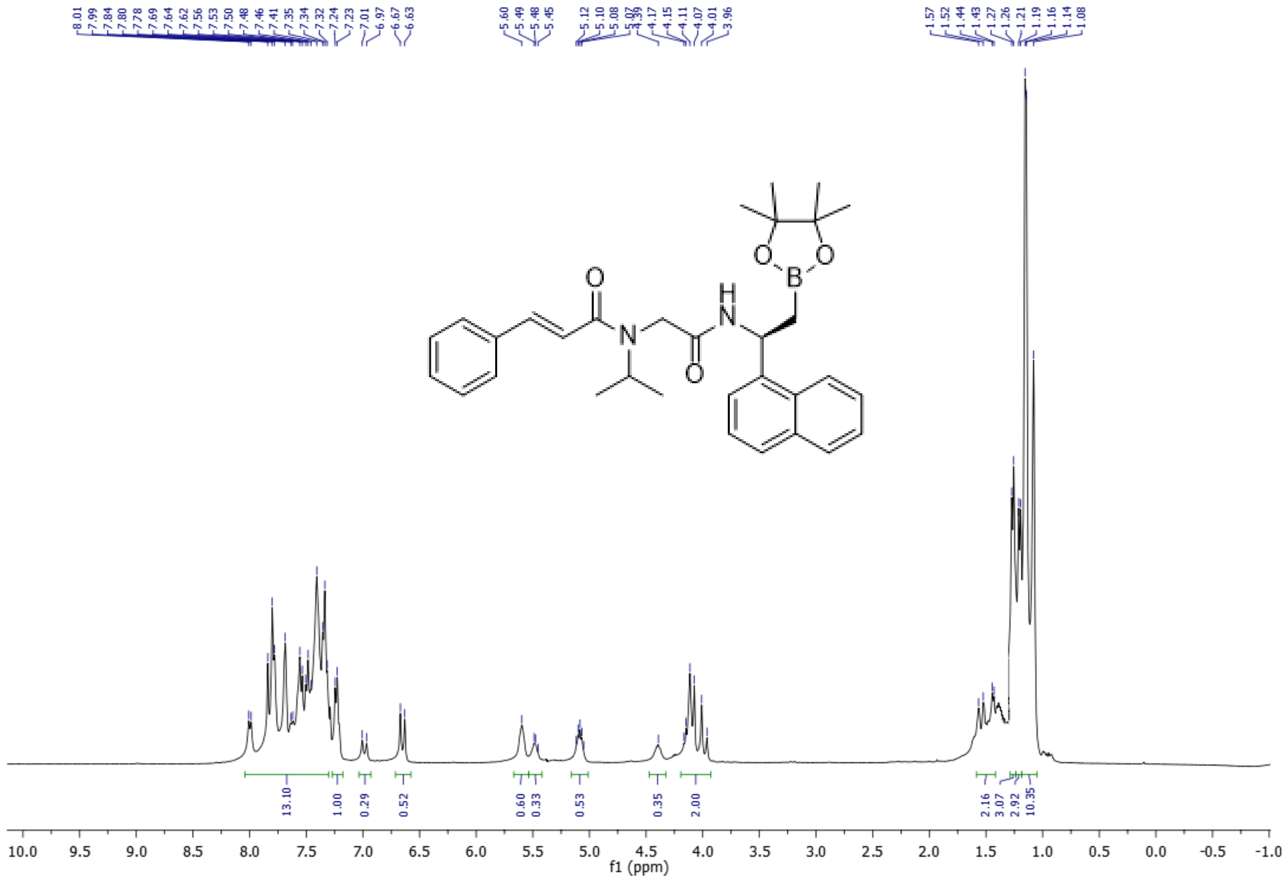

^{13}C NMR (101 MHz, $CDCl_3$, rotamers mixture 65:35) of compound **4j**

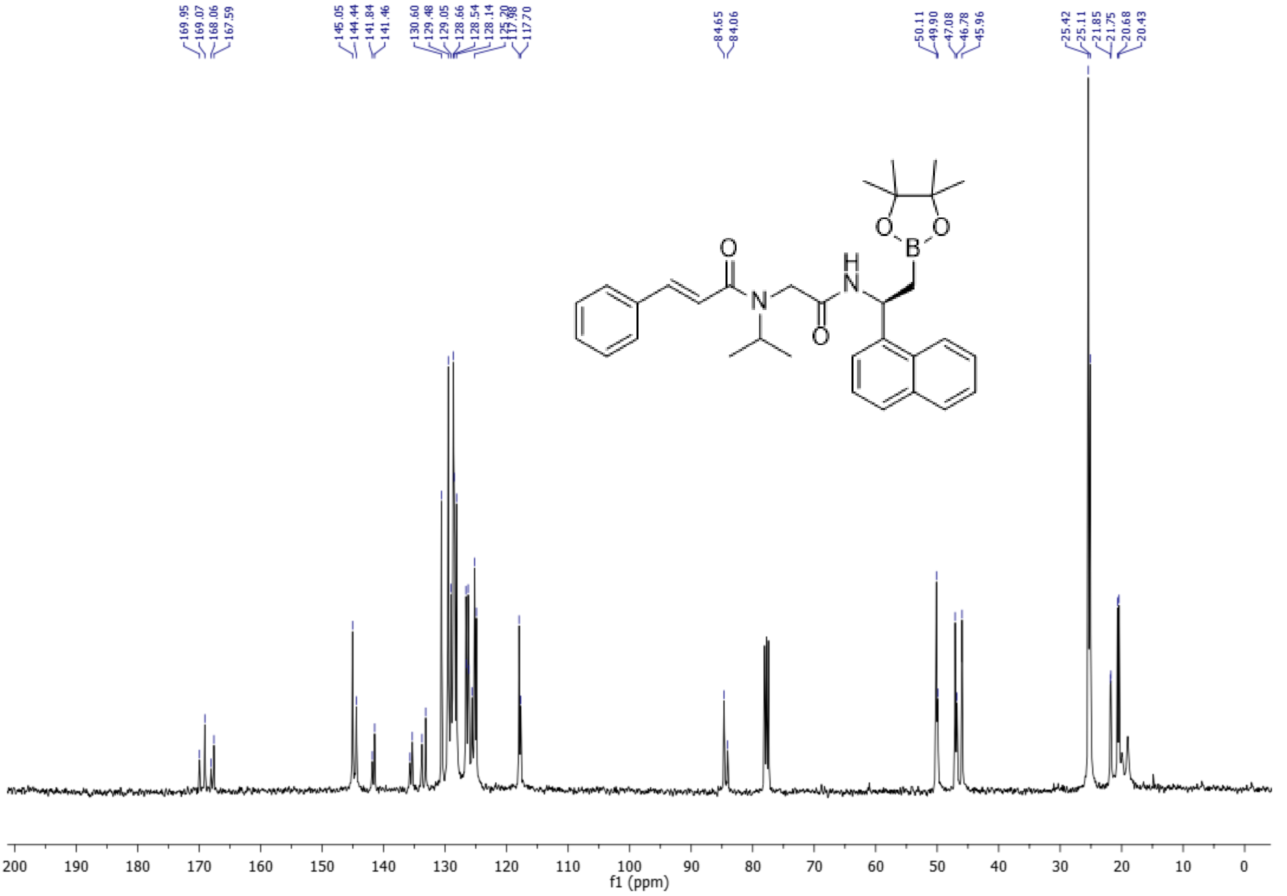

^{11}B NMR (128 MHz, $CDCl_3$) of compound **4j**

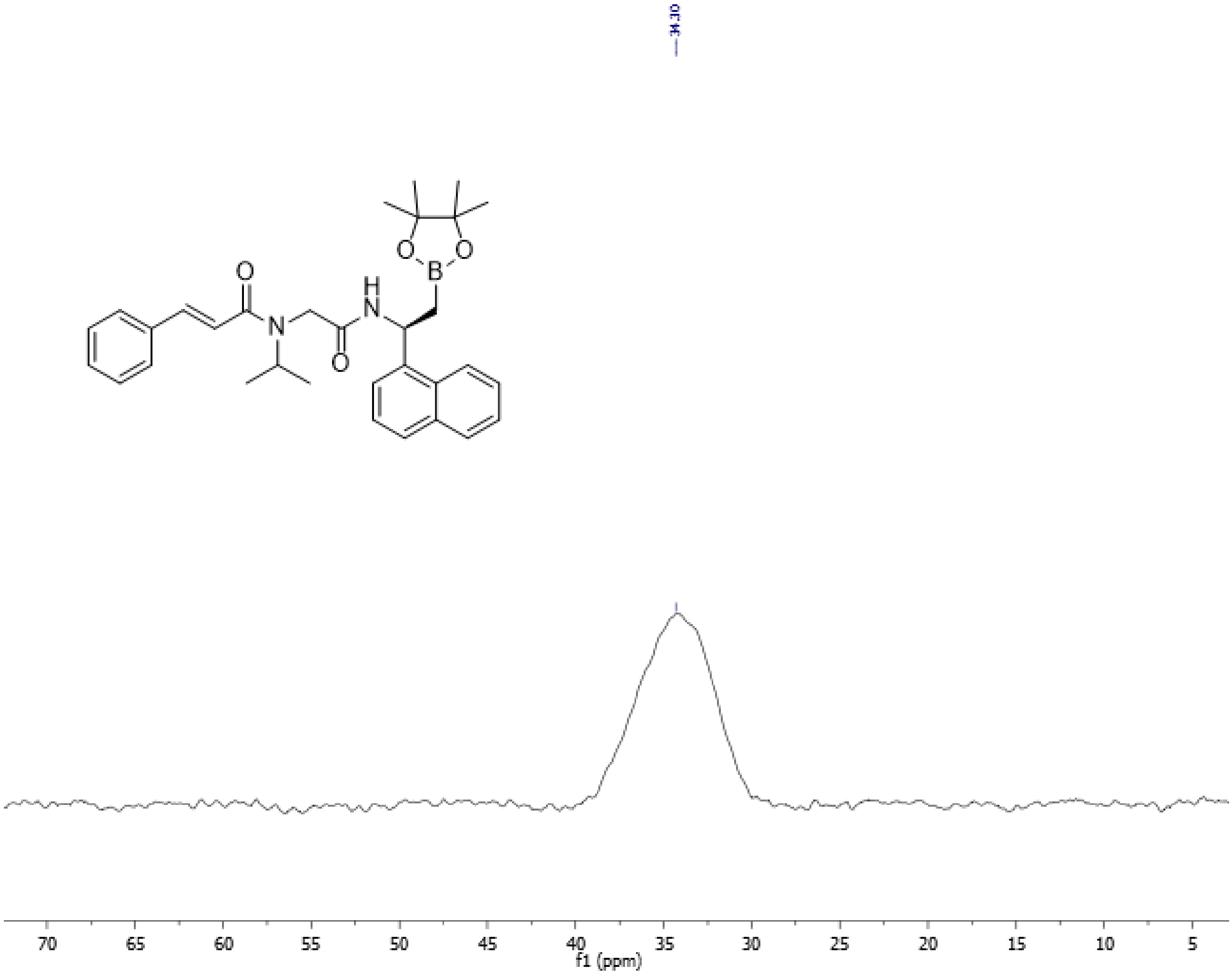

^{1}H NMR (400 MHz, $CDCl_3$) of compound **5a**

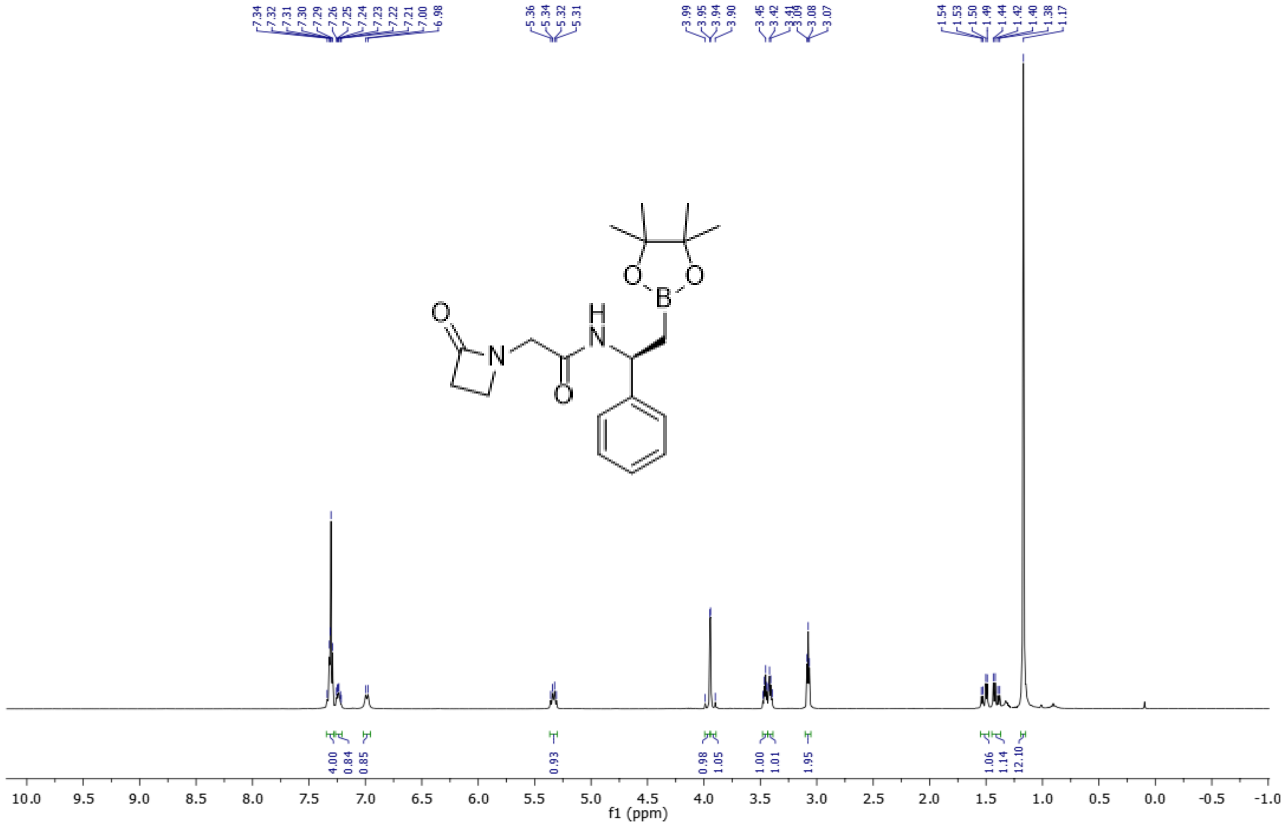

^{13}C NMR (101 MHz, $CDCl_3$) of compound **5a**

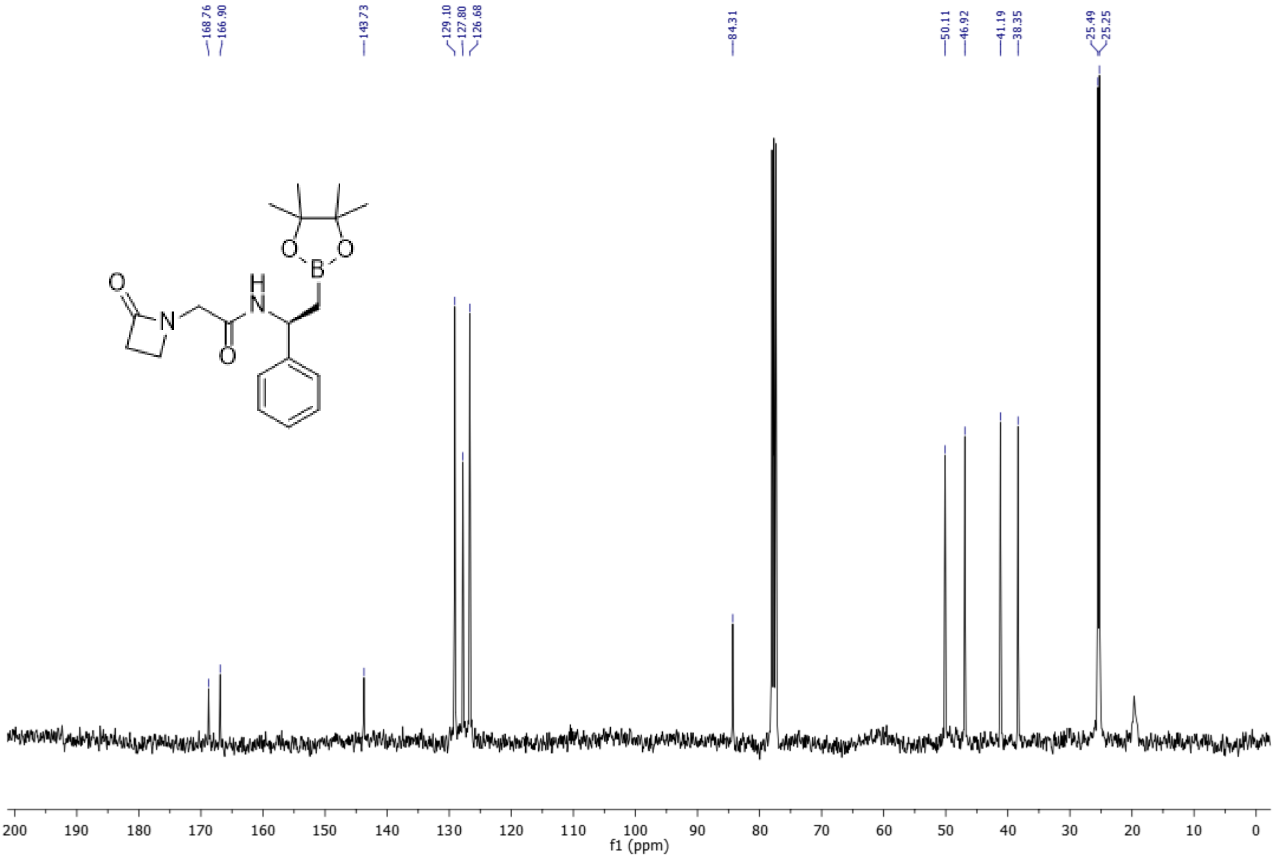

^{11}B NMR (128 MHz, $CDCl_3$) of compound **5a**

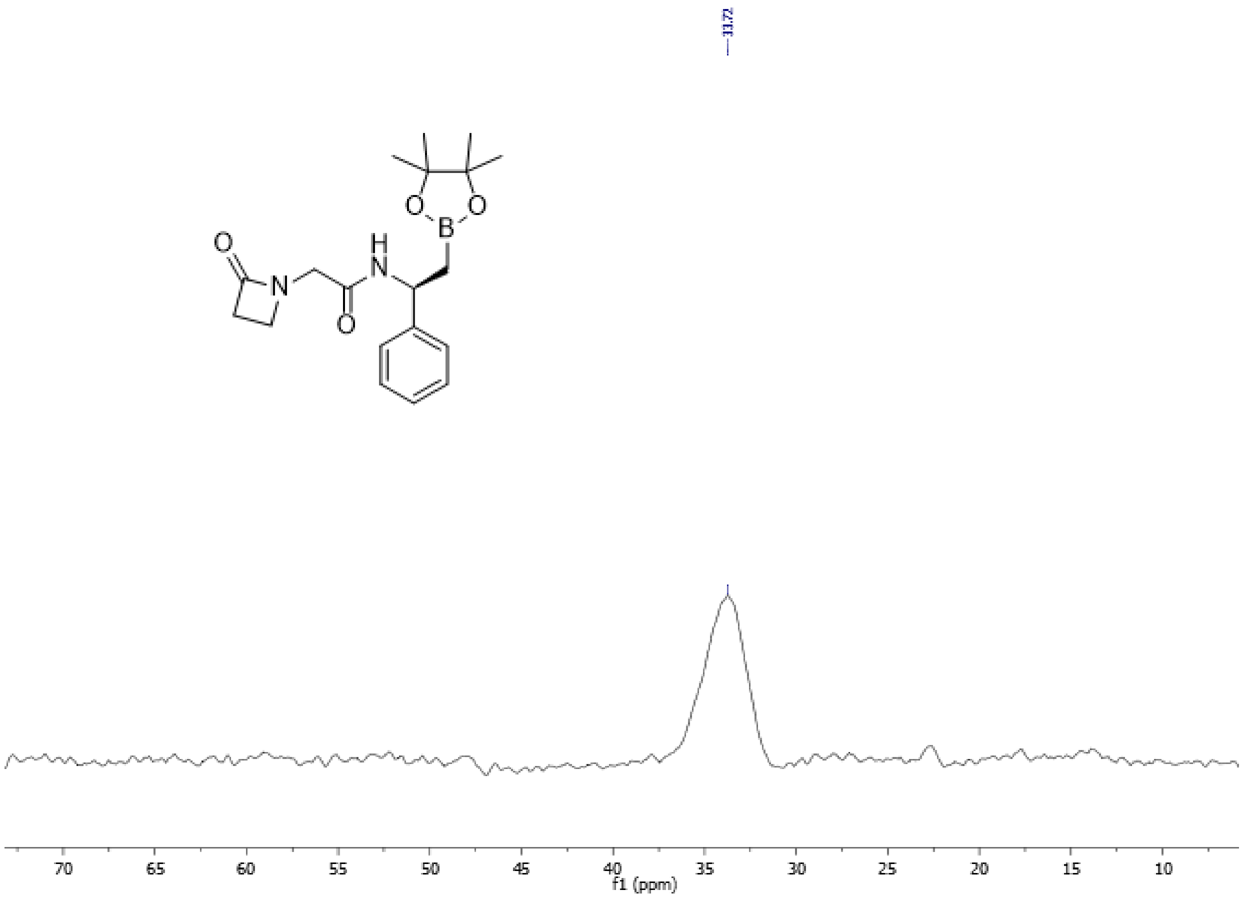

^{1}H NMR (400 MHz, $CDCl_3$) of compound **5b**

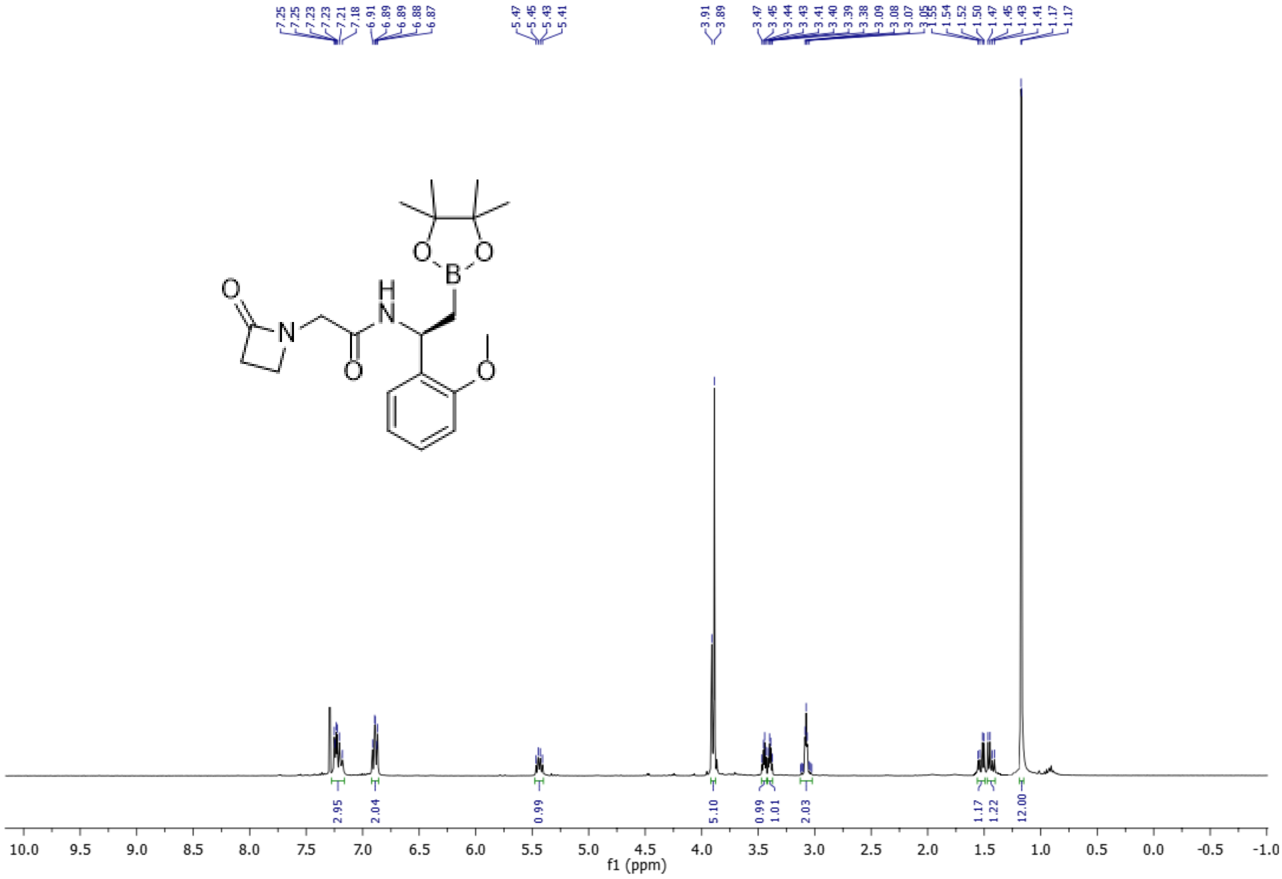

^{13}C NMR (101 MHz, $CDCl_3$) of compound **5b**

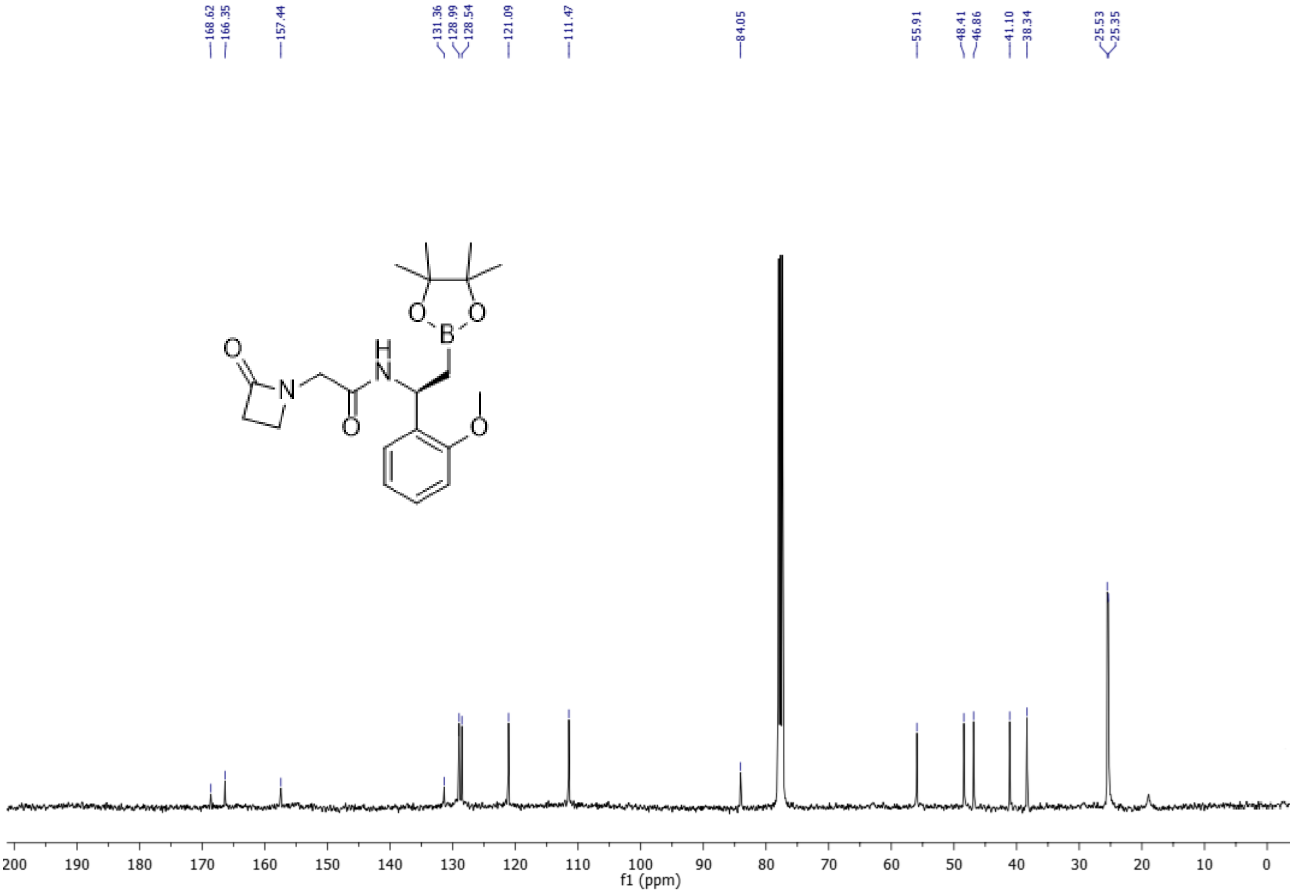

^{11}B NMR (128 MHz, $CDCl_3$) of compound **5b**

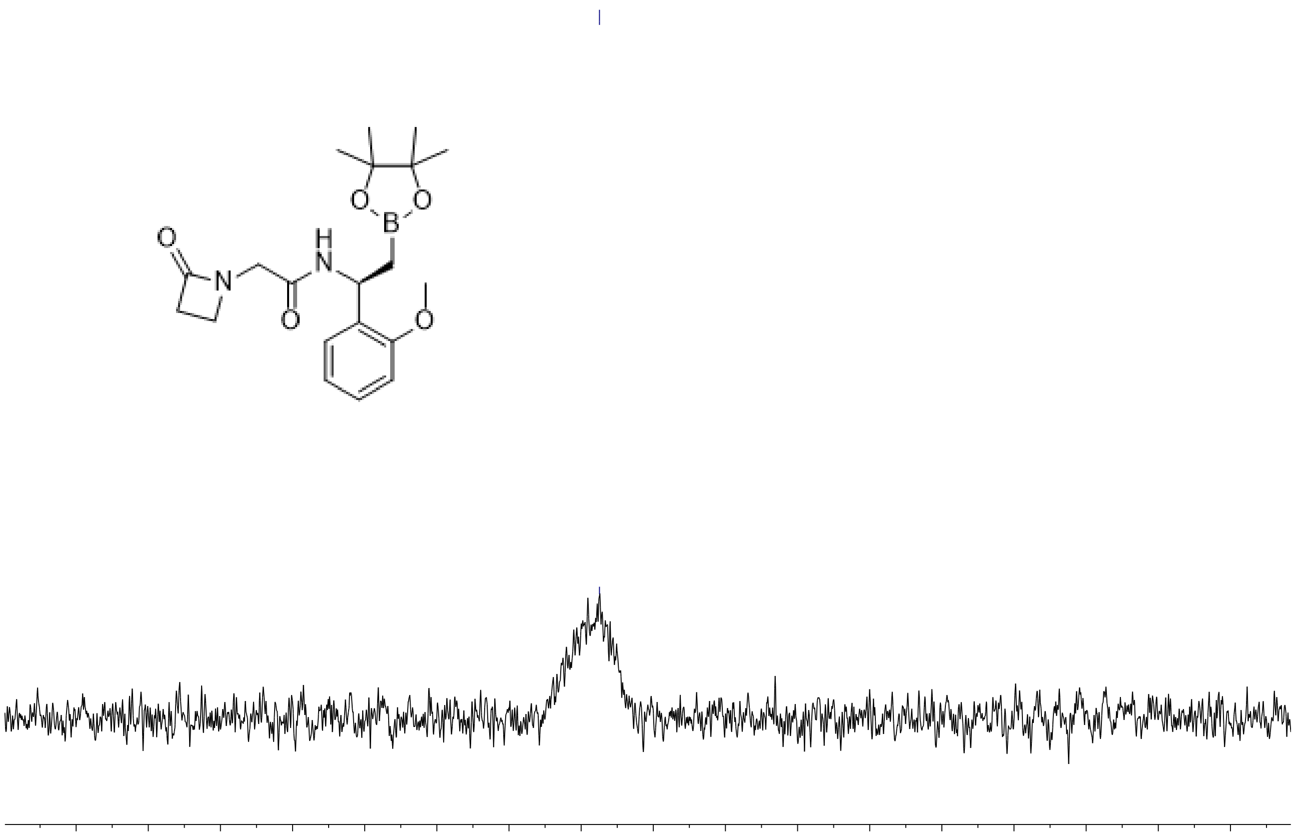

^{1}H NMR (400 MHz, $CDCl_3$) of compound **6**

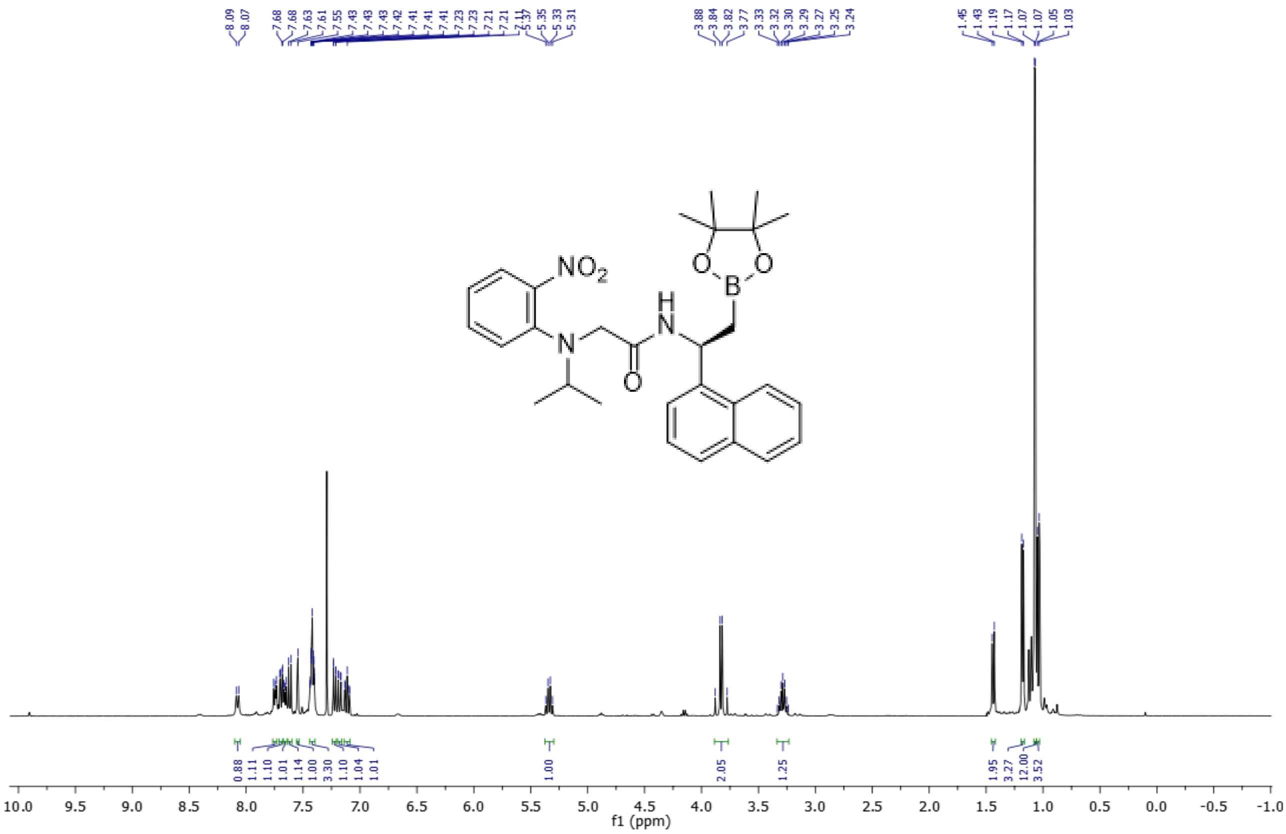

^{13}C NMR (101 MHz, $CDCl_3$) of compound **6**

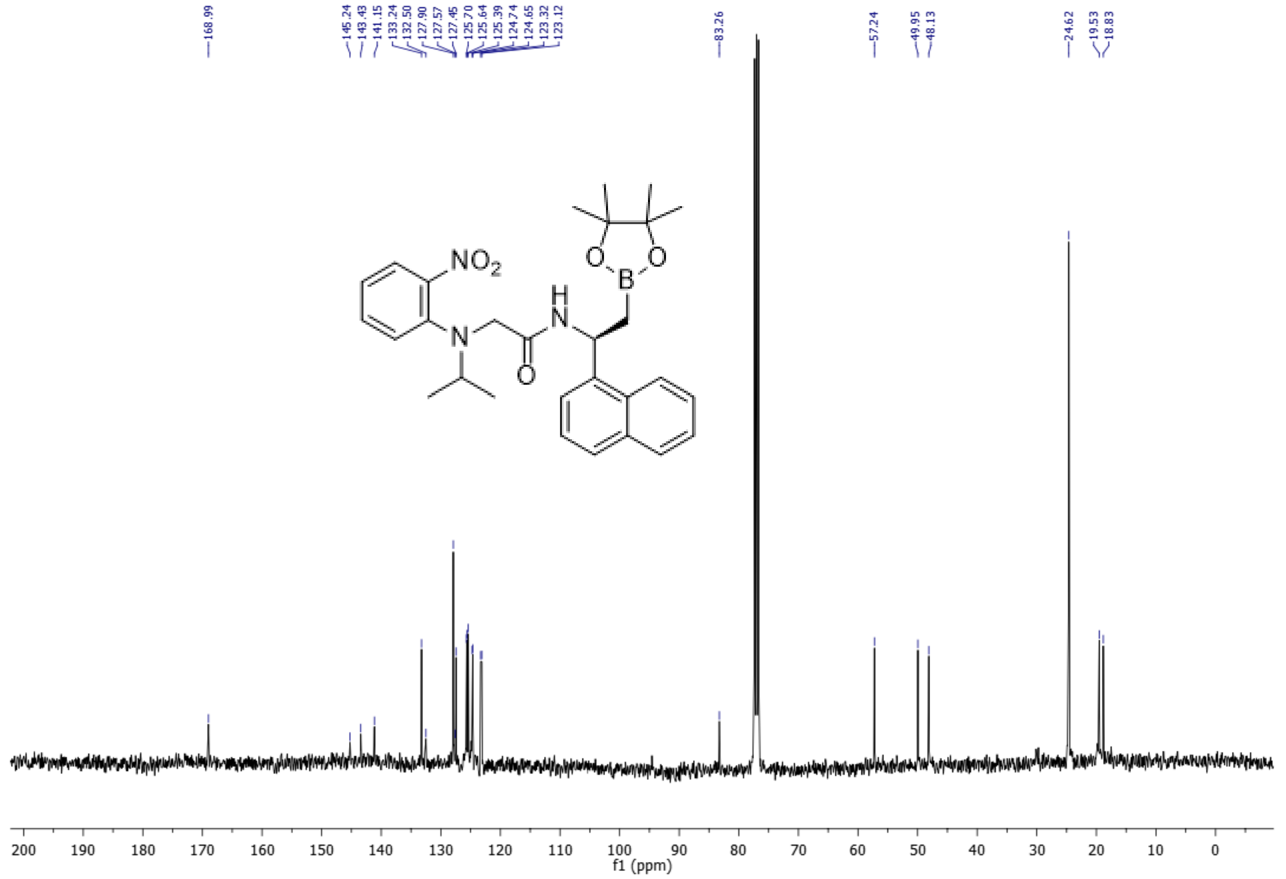

^{11}B NMR (128 MHz, $CDCl_3$) of compound **6**

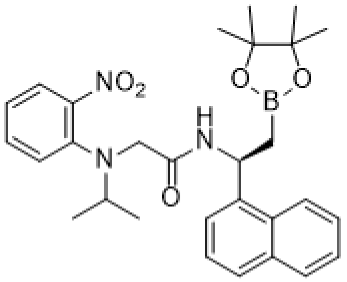

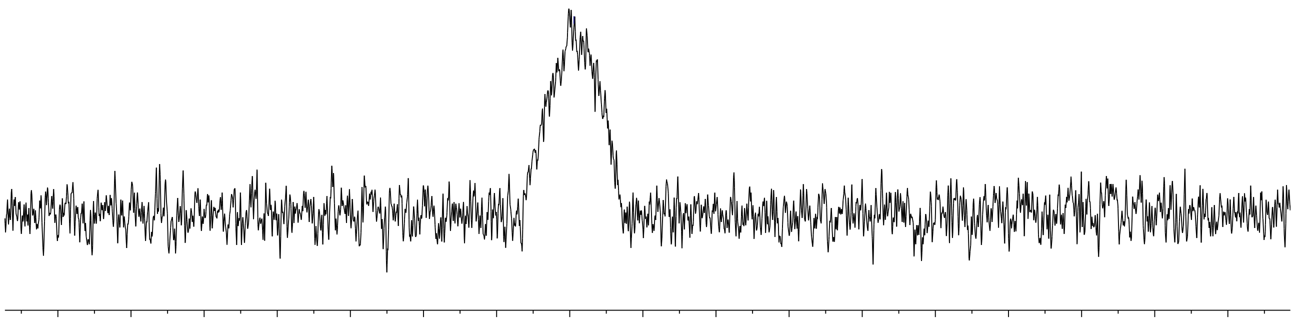

^{1}H NMR (400 MHz, $CDCl_3$) of compound **7**

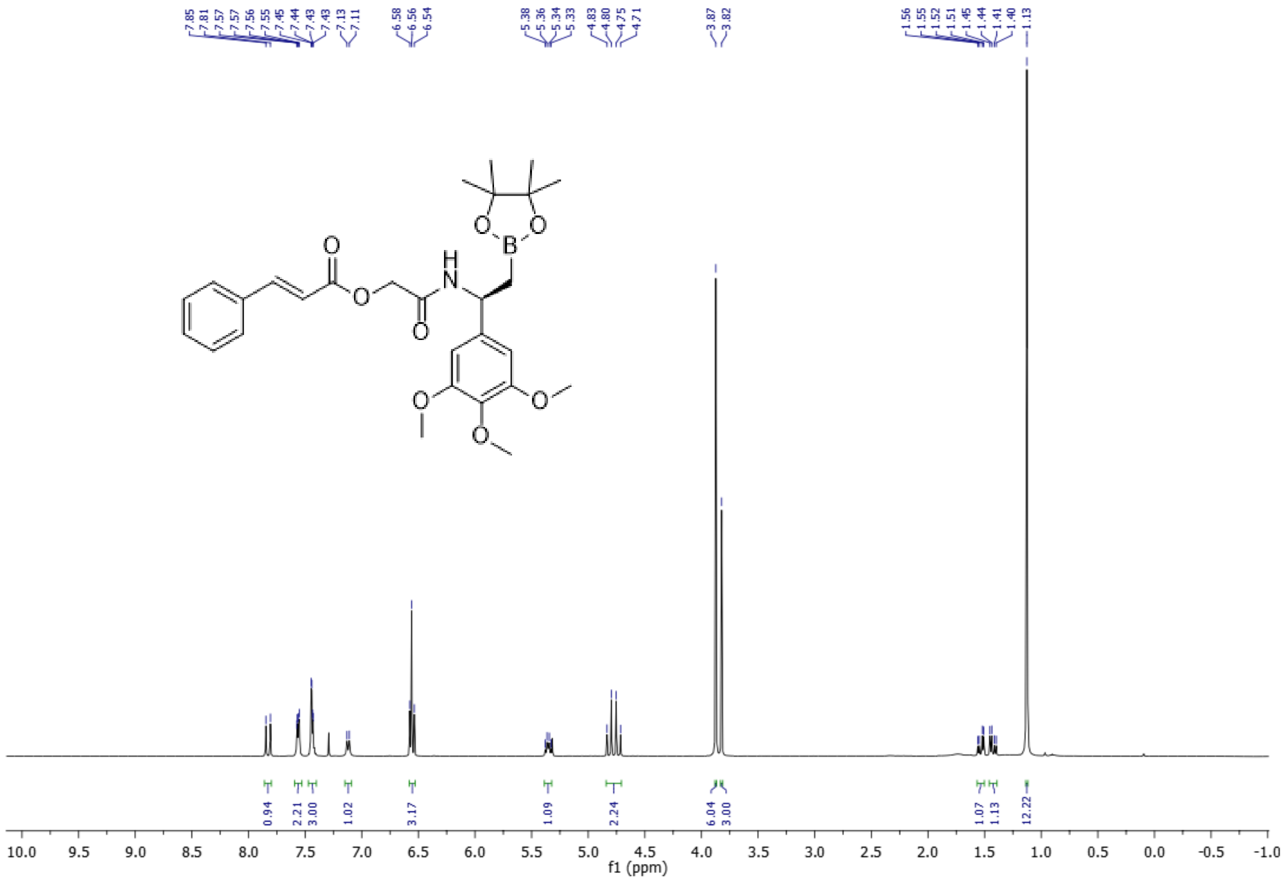

^{13}C NMR (101 MHz, $CDCl_3$) of compound **7**

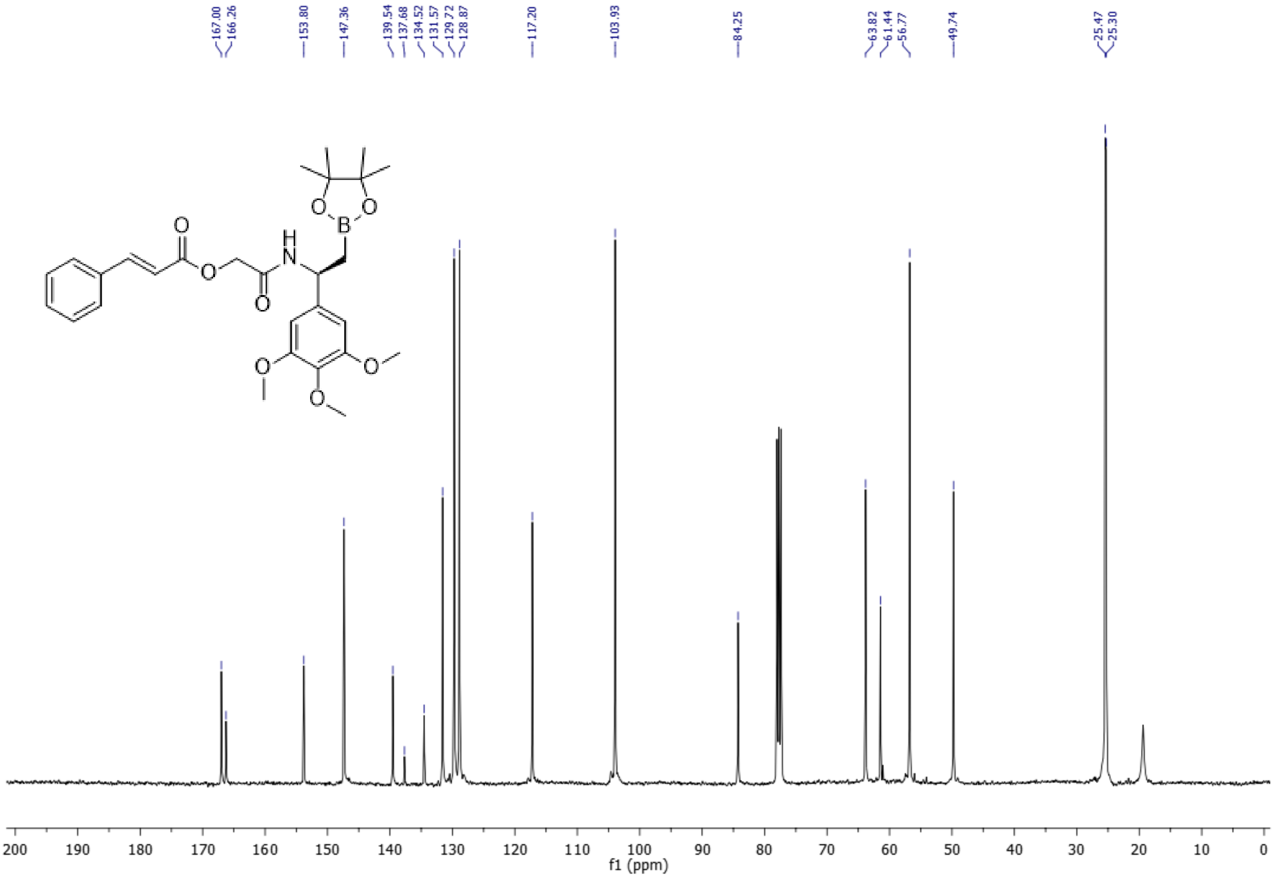

^{11}B NMR (128 MHz, $CDCl_3$) of compound **7**

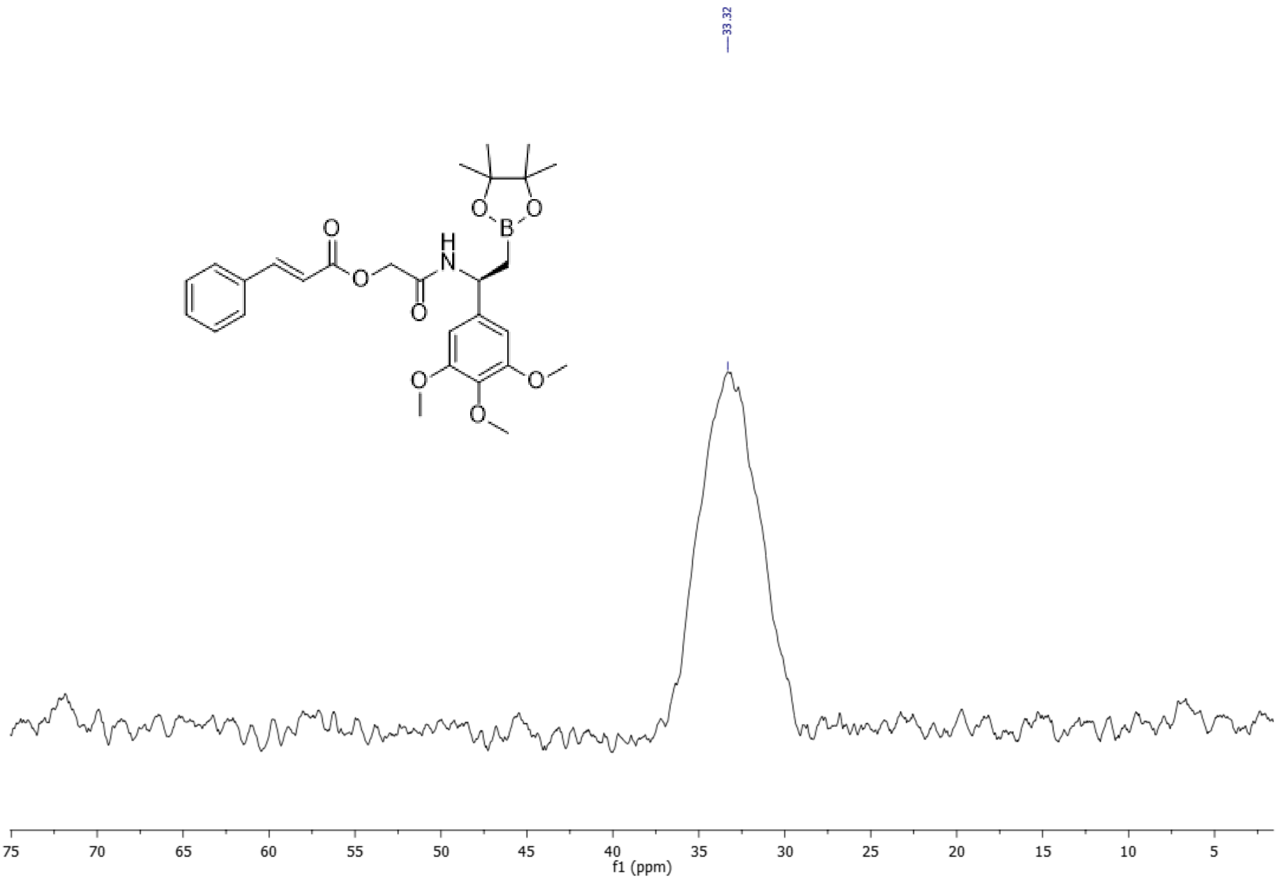

^{1}H NMR (400 MHz, $CDCl_3$) of compound **8**

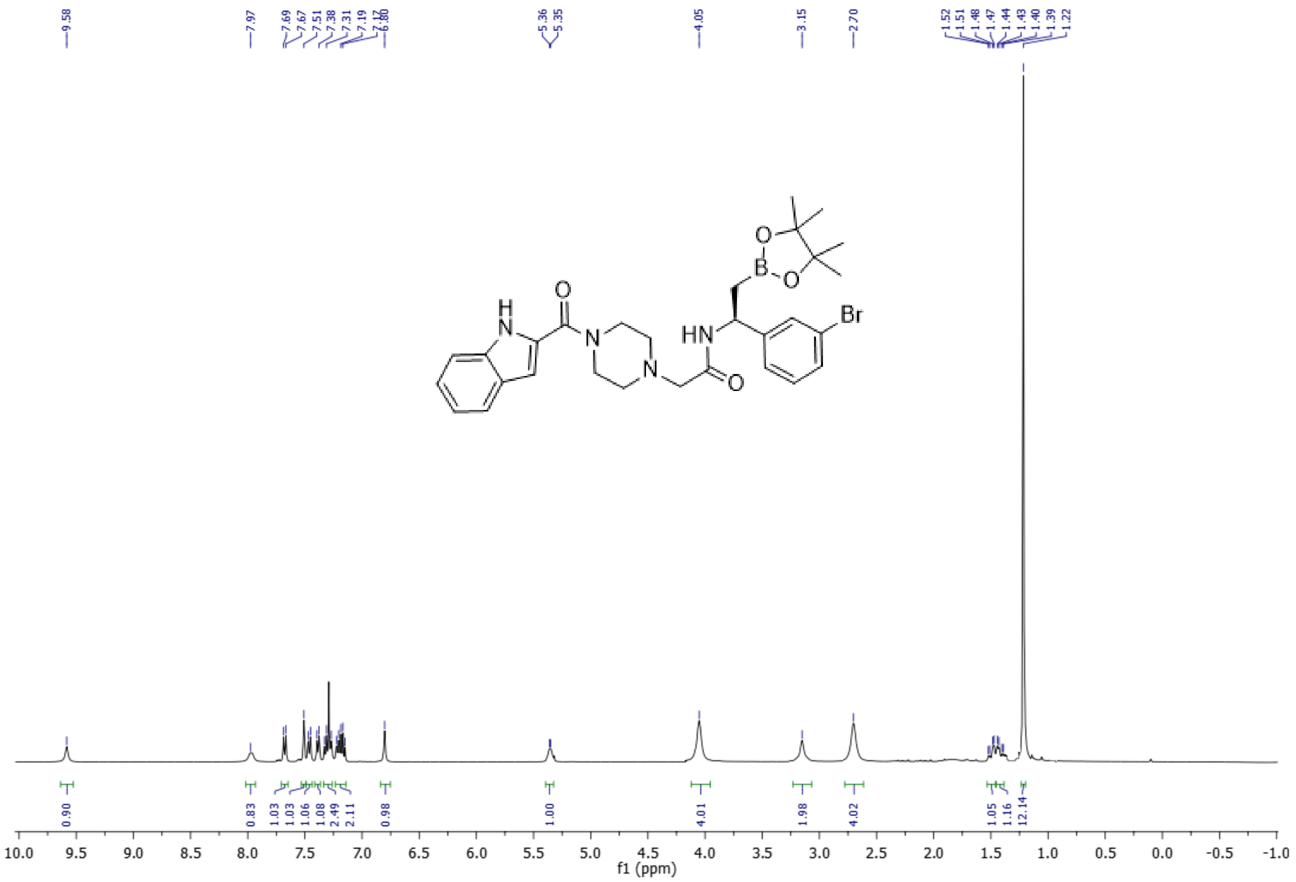

^{13}C NMR (101 MHz, $CDCl_3$) of compound **8**

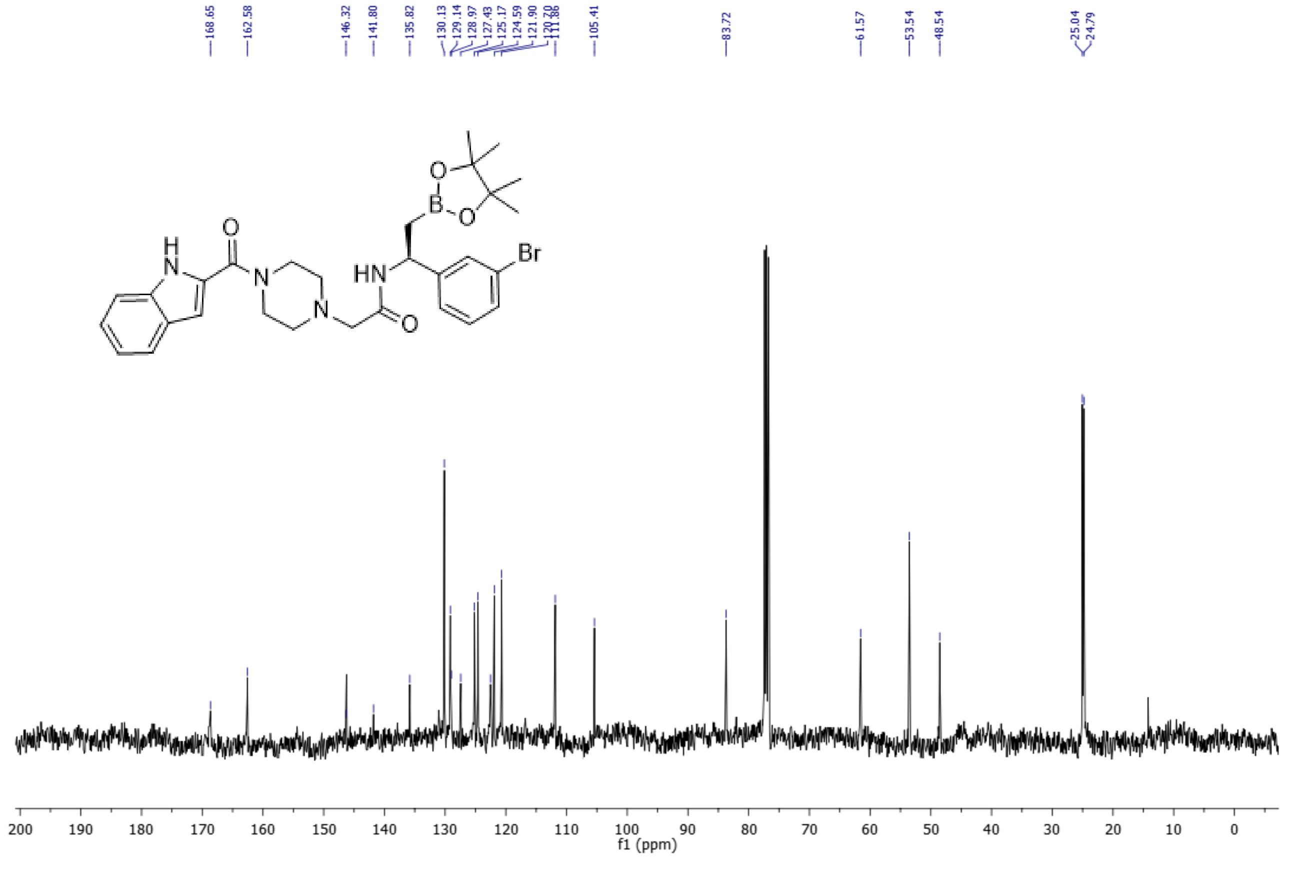

^{11}B NMR (128 MHz, $CDCl_3$) of compound **8**

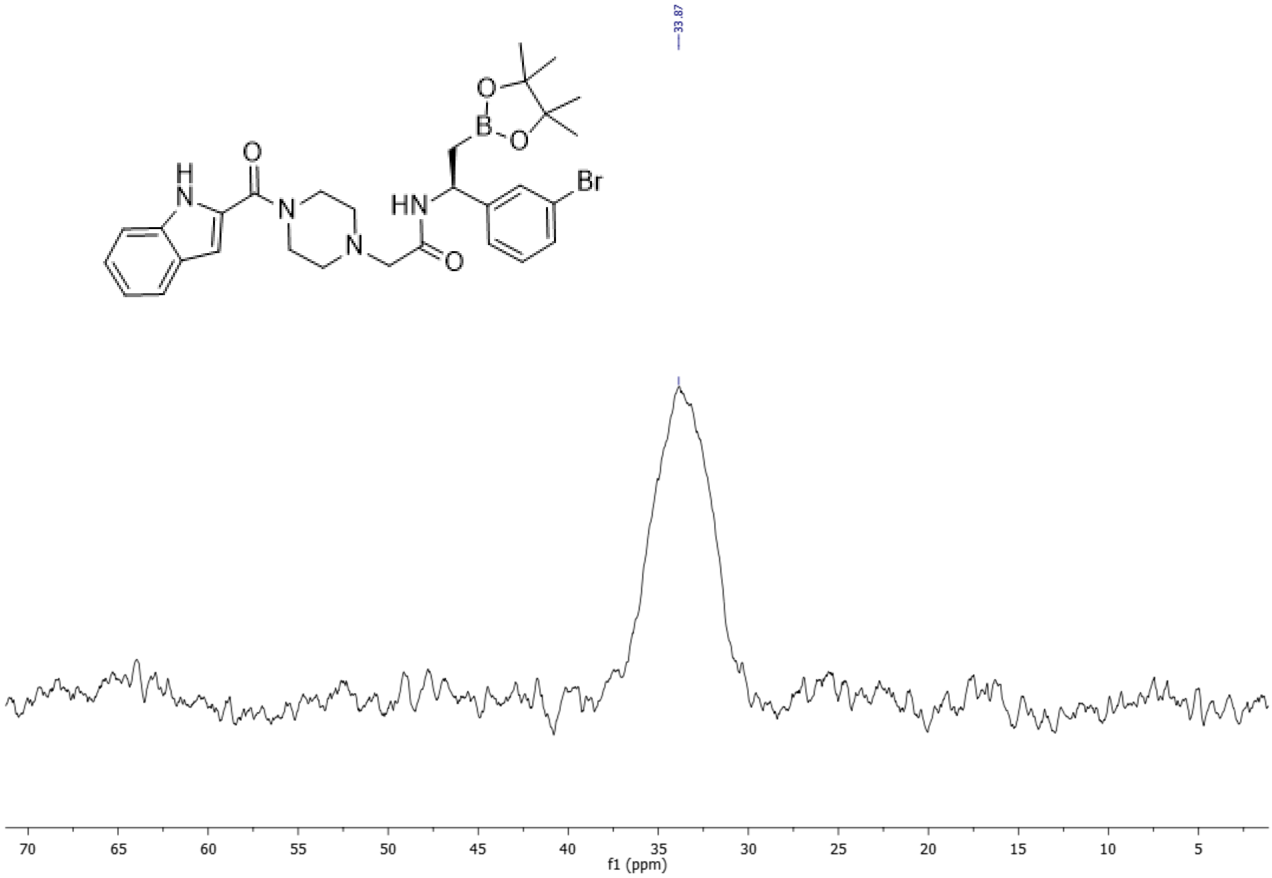

^{1}H NMR (400 MHz, $CDCl_3$) of compound **9**

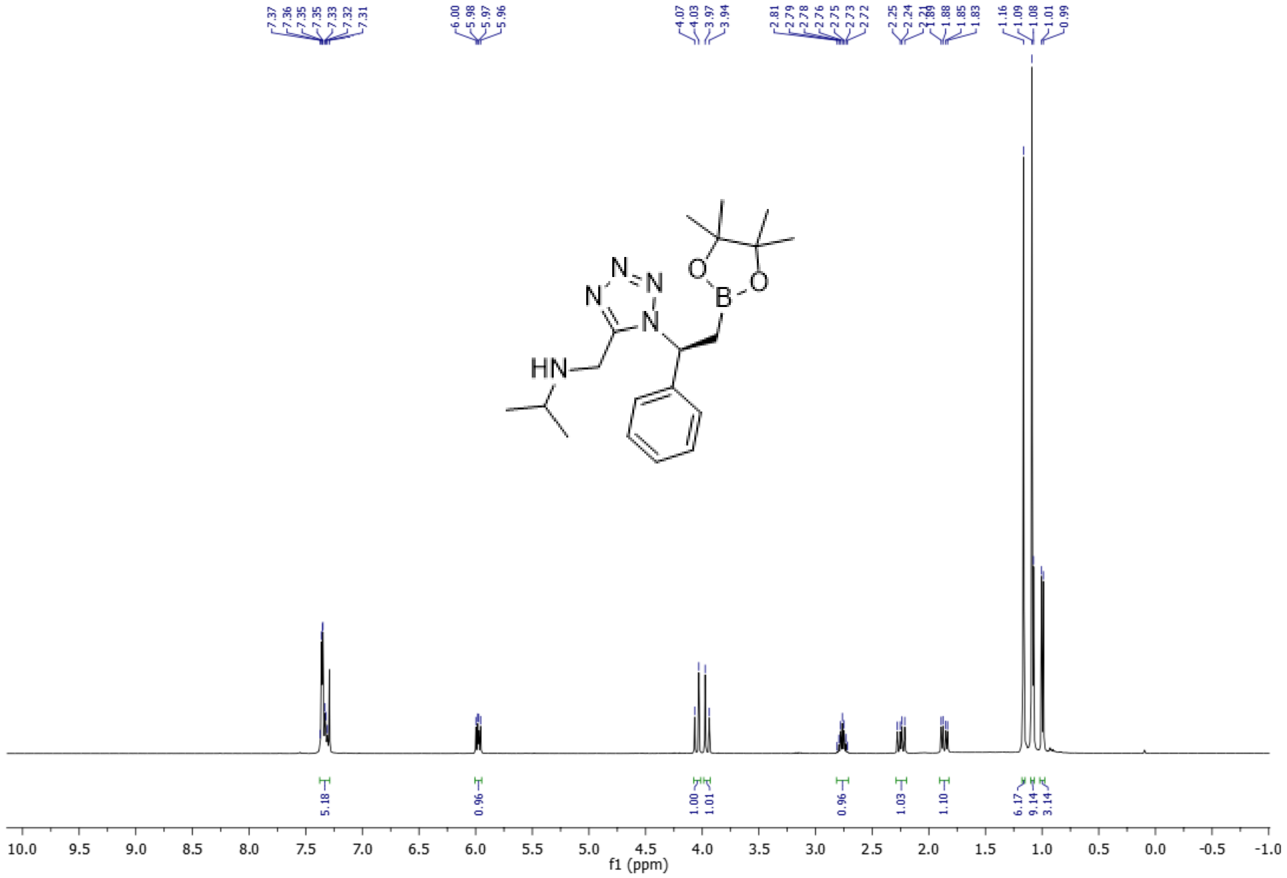

^{13}C NMR (101 MHz, $CDCl_3$) of compound **9**

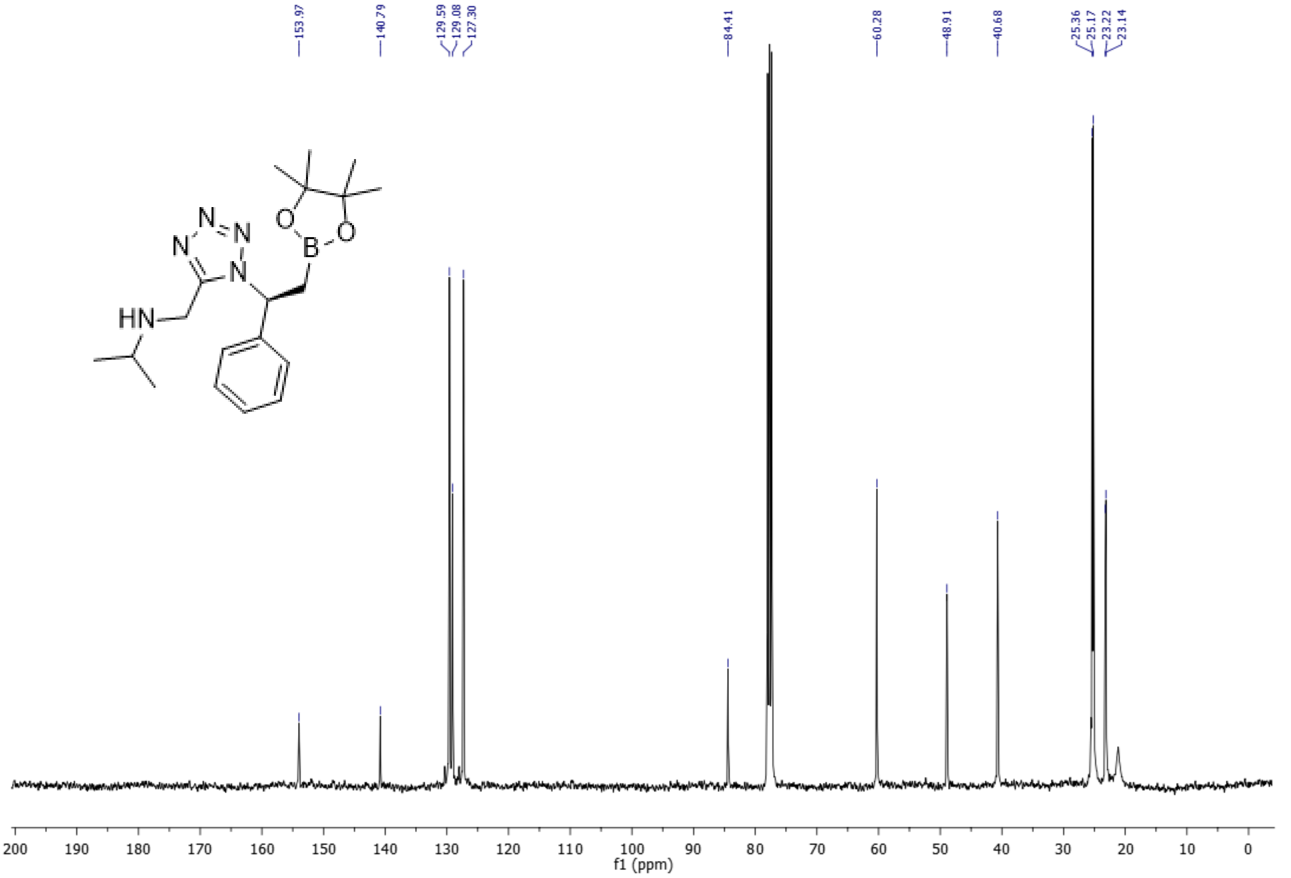

^{11}B NMR (128 MHz, $CDCl_3$) of compound **9**

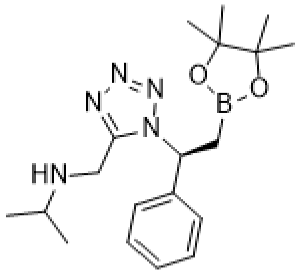

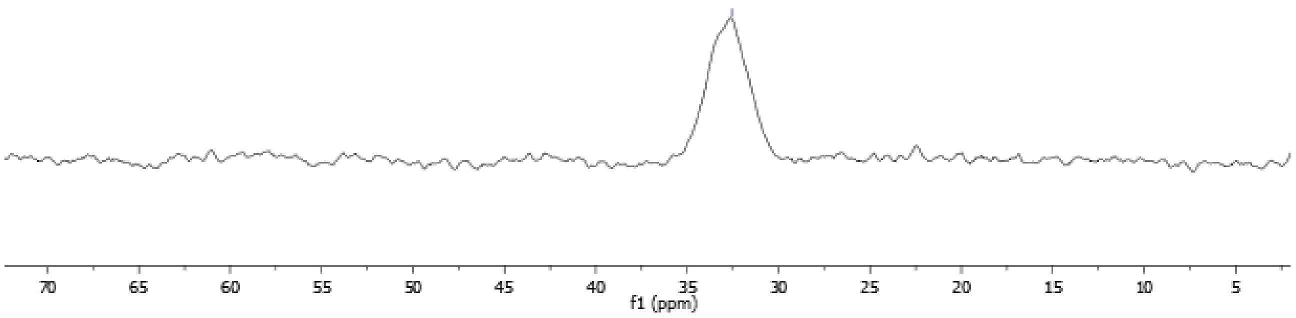

^{1}H NMR (400 MHz, $CDCl_3$) of compound **10**

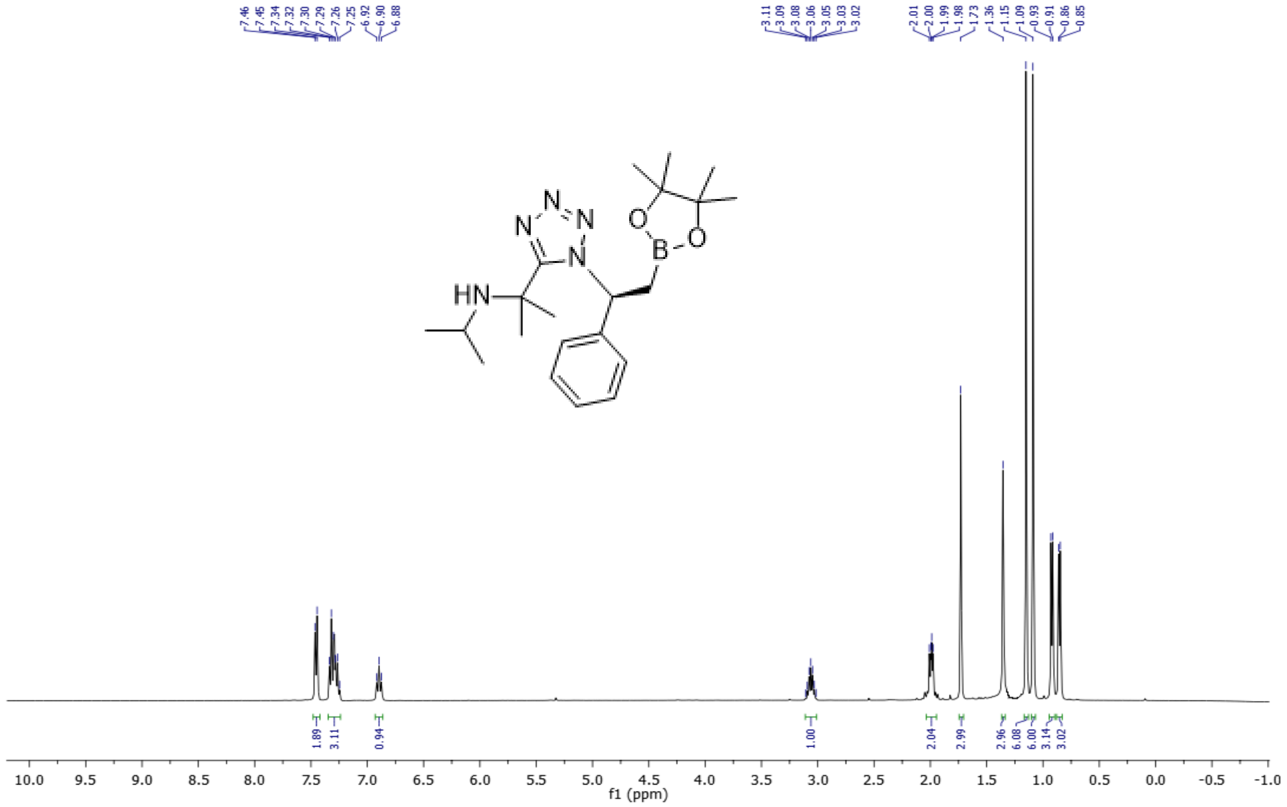

^{13}C NMR (101 MHz, $CDCl_3$) of compound **10**

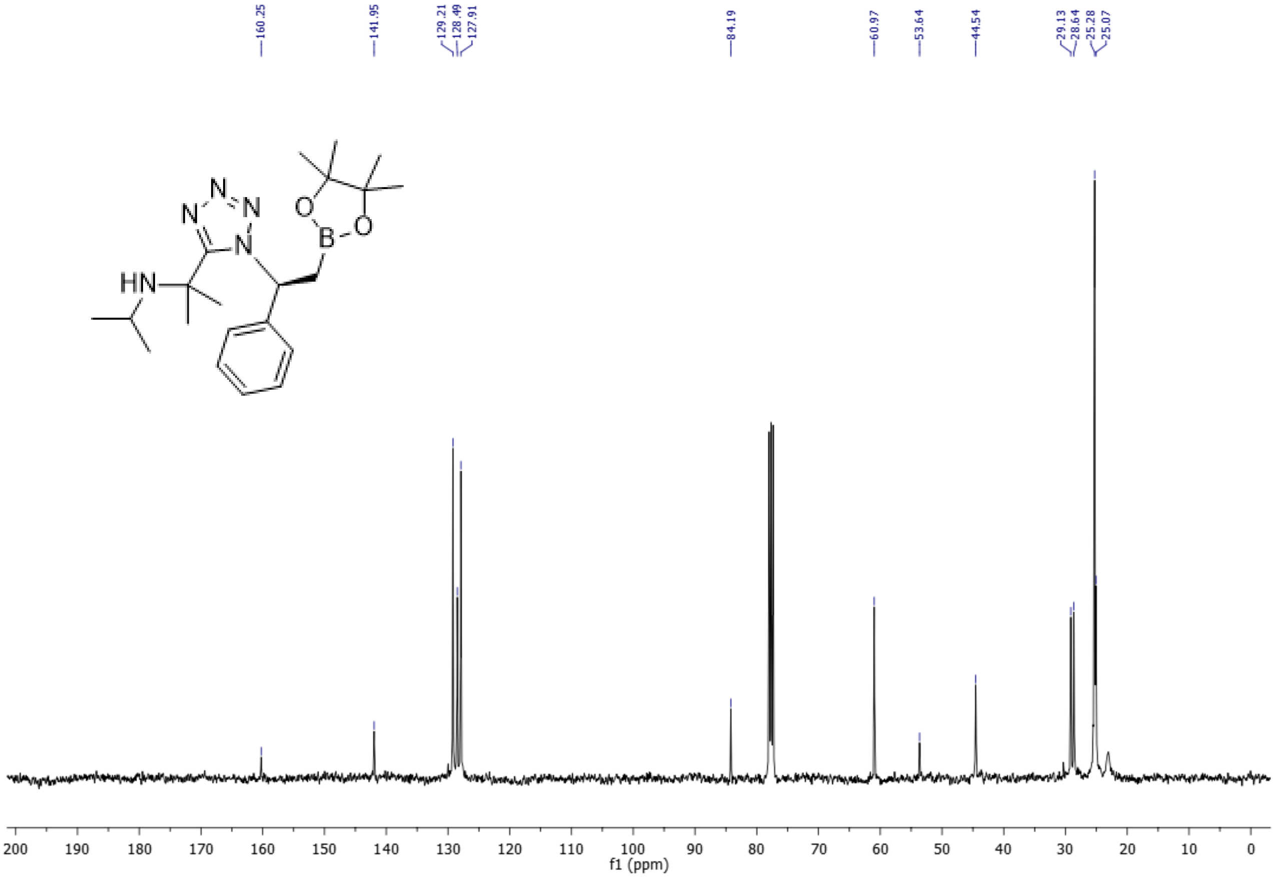

^{11}B NMR (128 MHz, $CDCl_3$) of compound **10**

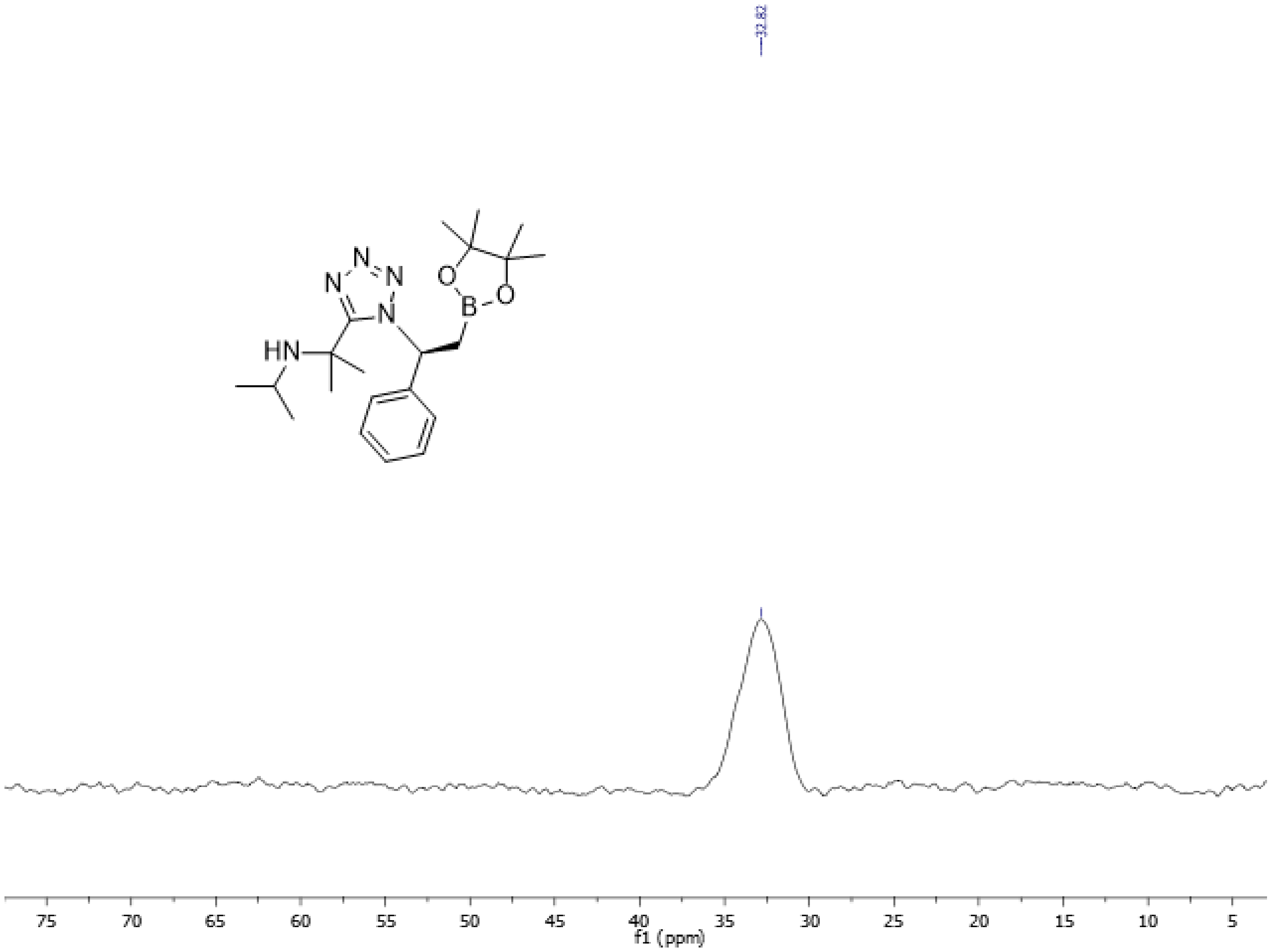

^{1}H NMR (400 MHz, $CDCl_3$) of compound **11a**

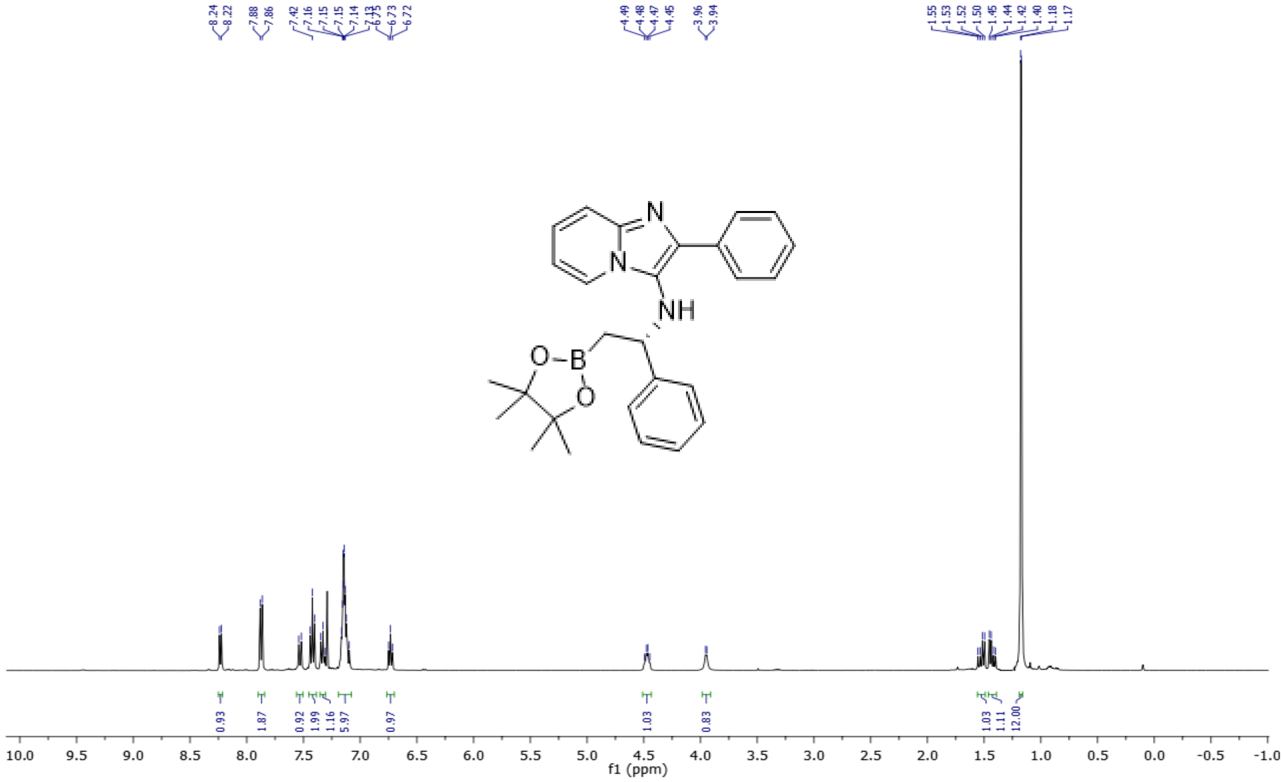

^{13}C NMR (101 MHz, $CDCl_3$) of compound **11a**

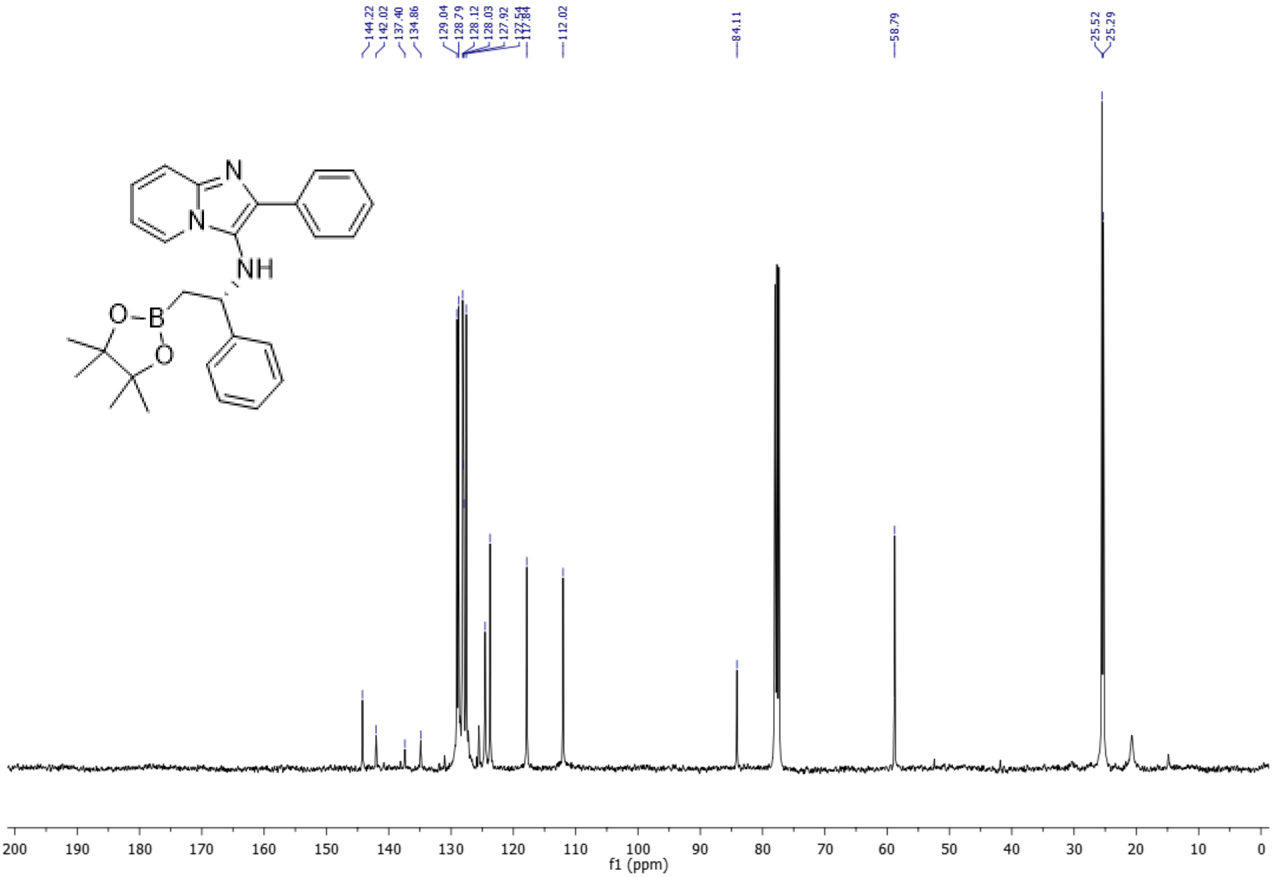

^{11}B NMR (128 MHz, $CDCl_3$) of compound **11a**

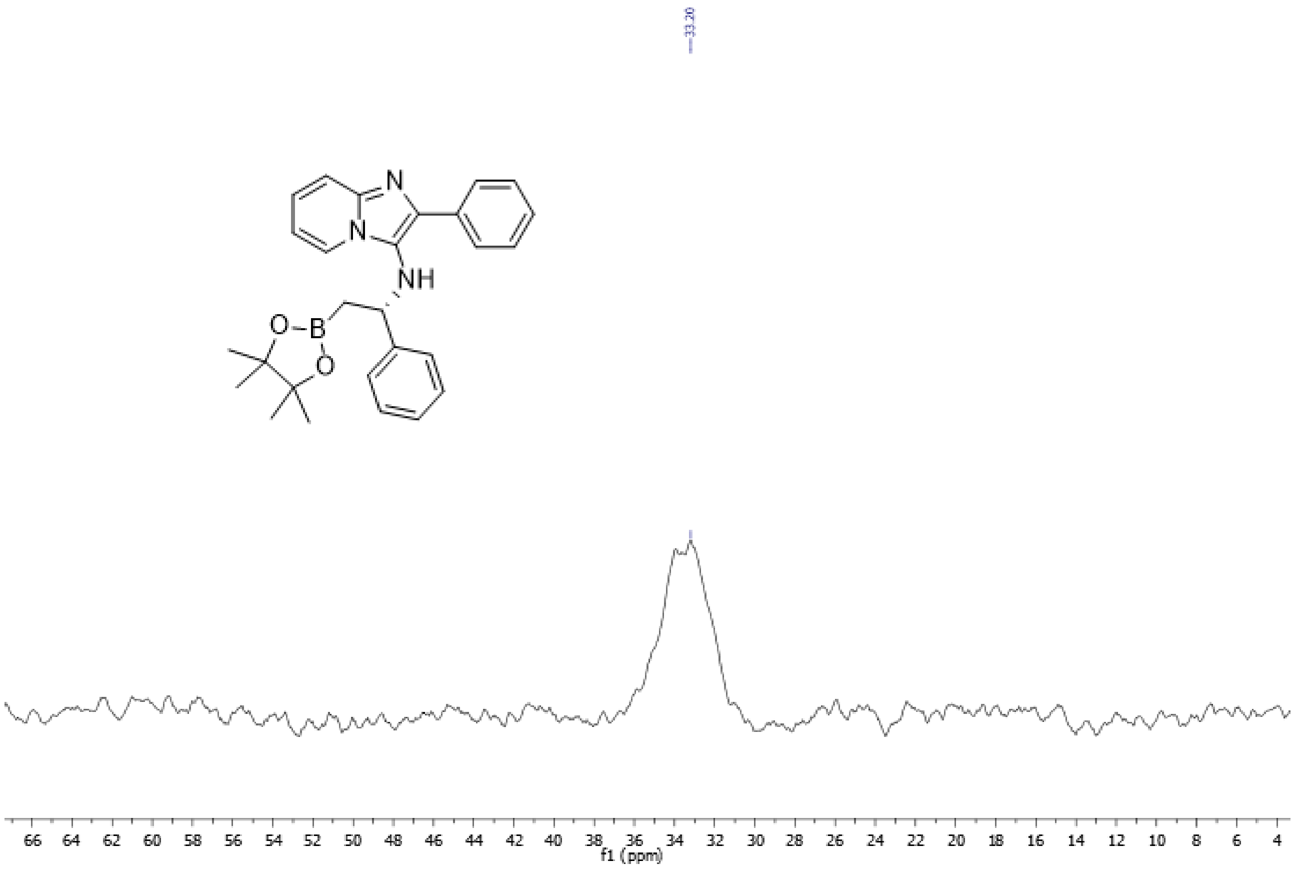

^{1}H NMR (400 MHz, $CDCl_3$) of compound **11h**

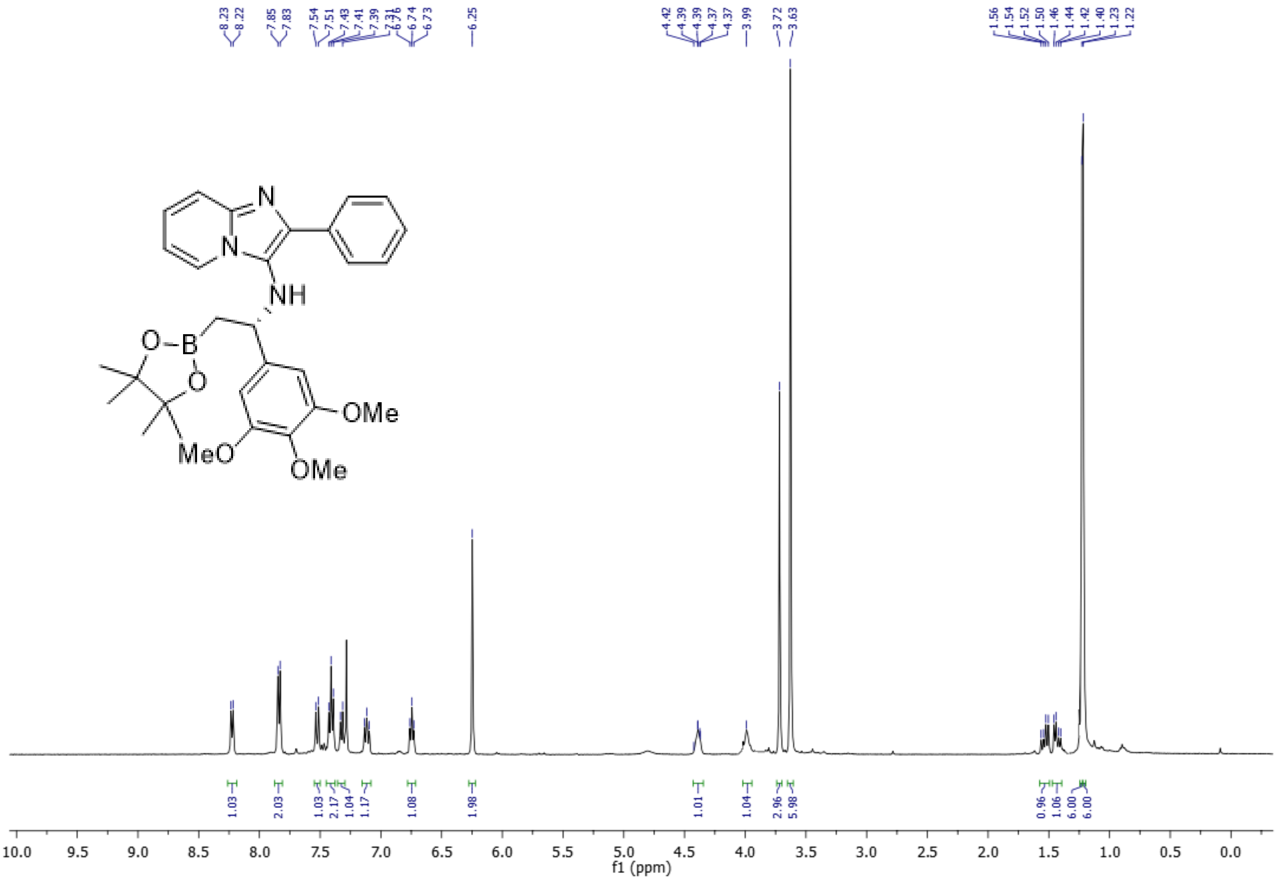

^{13}C NMR (101 MHz, $CDCl_3$) of compound **11h**

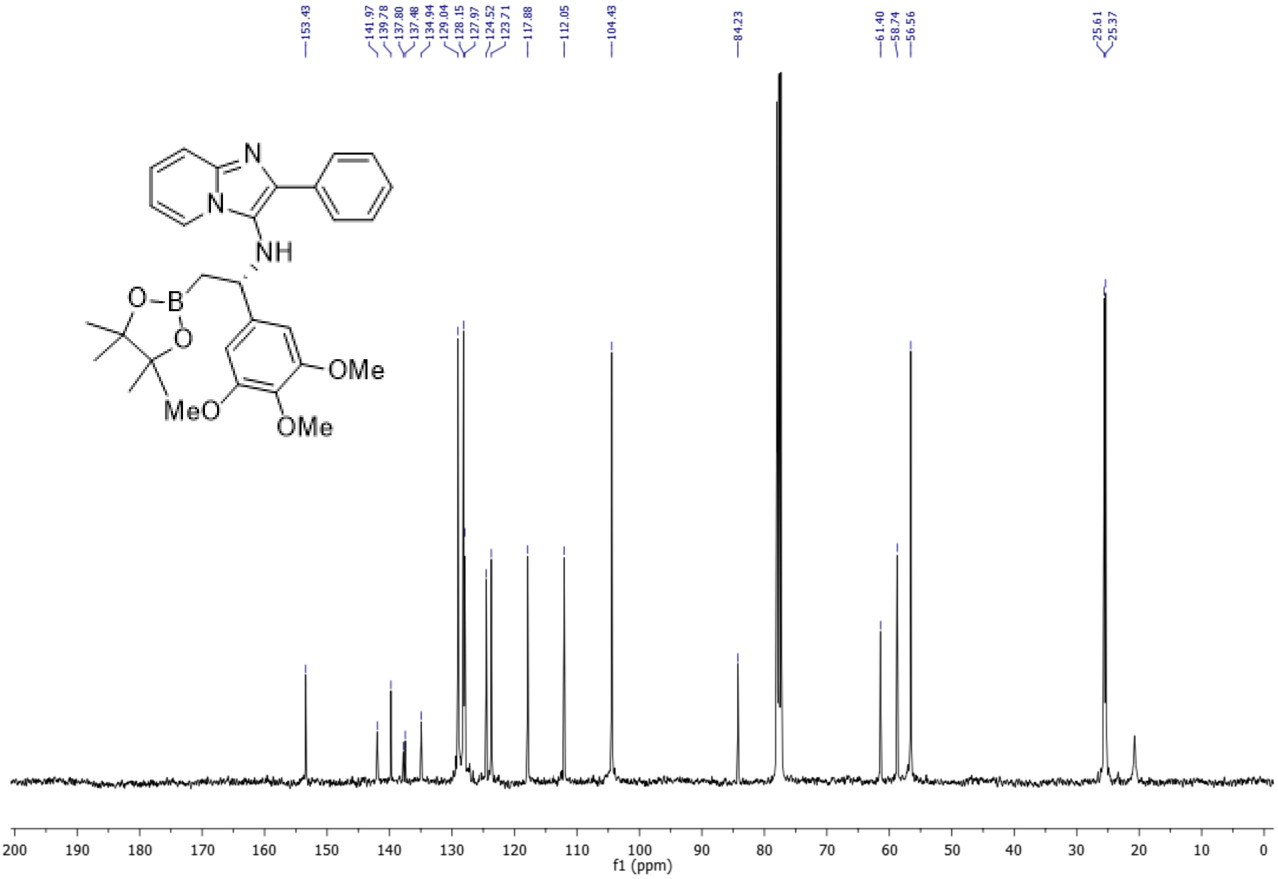

^{11}B NMR (128 MHz, $CDCl_3$) of compound **11h**

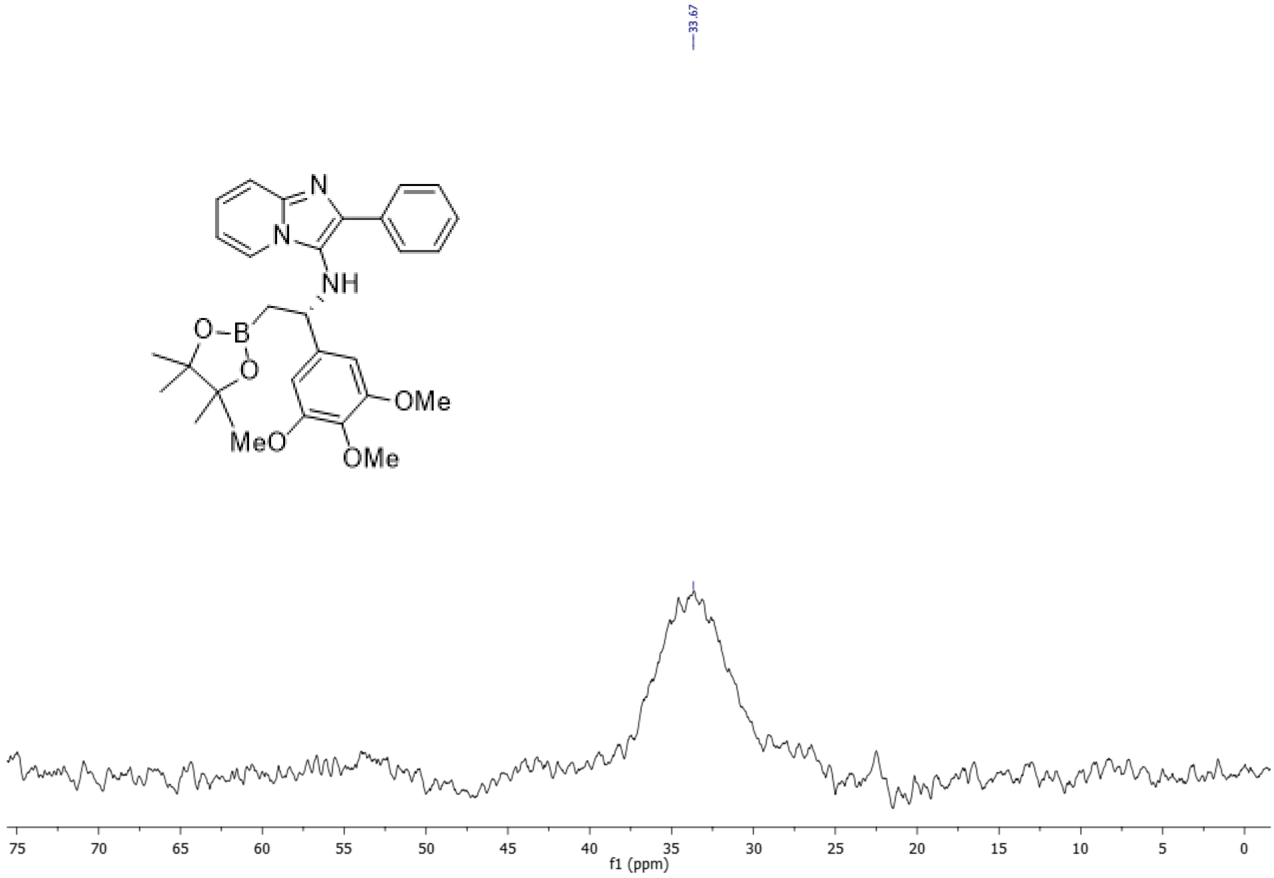

^{1}H NMR (400 MHz, Acetone d-6 + 1 drop of H_2O) of compound **12**

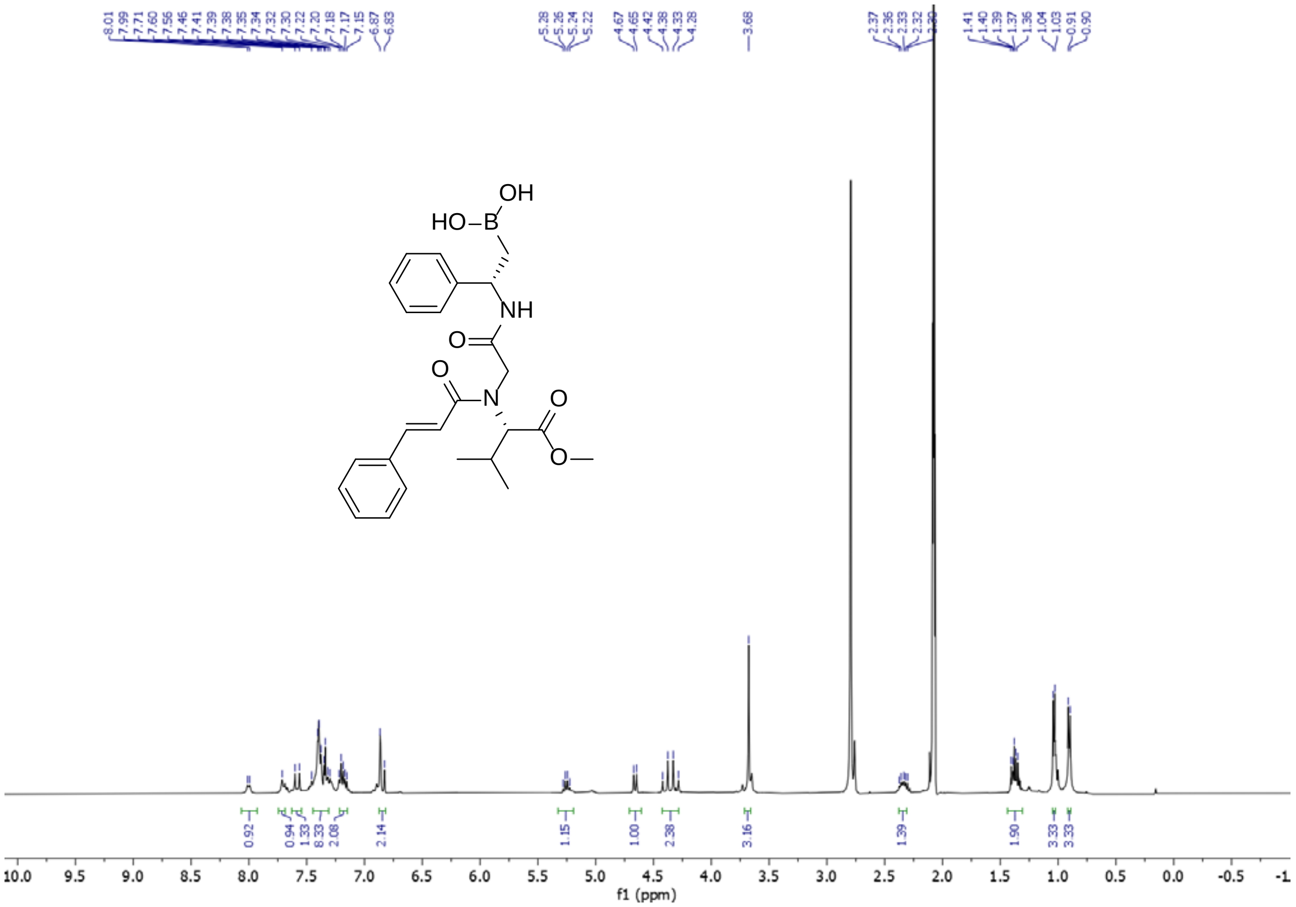

^{13}C NMR (101 MHz, Acetone d-6 + 1 drop of H_2O) of compound **12**

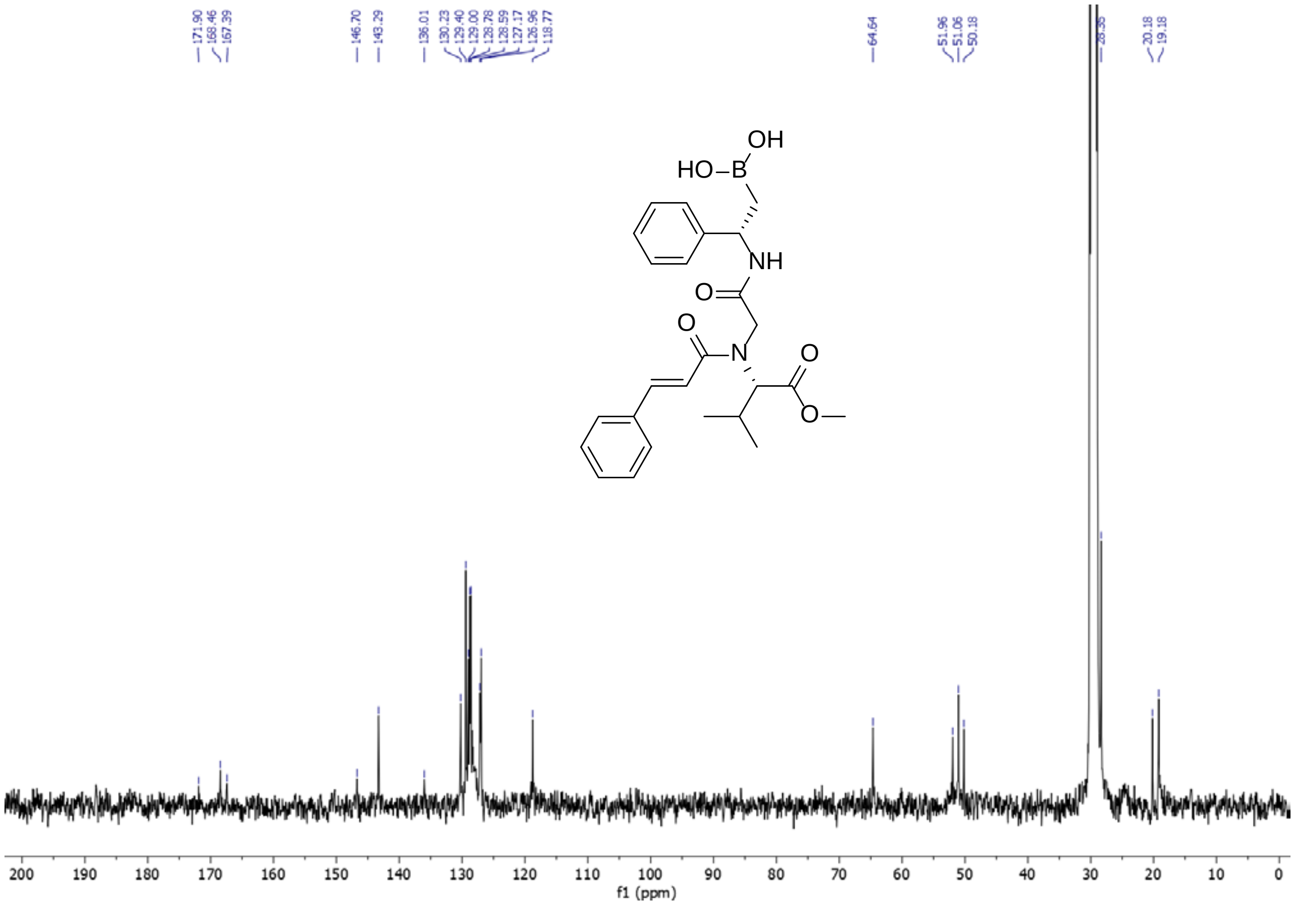

^{11}B NMR (128 MHz, Acetone d-6 + 1 drop of H_2O) of compound **12**

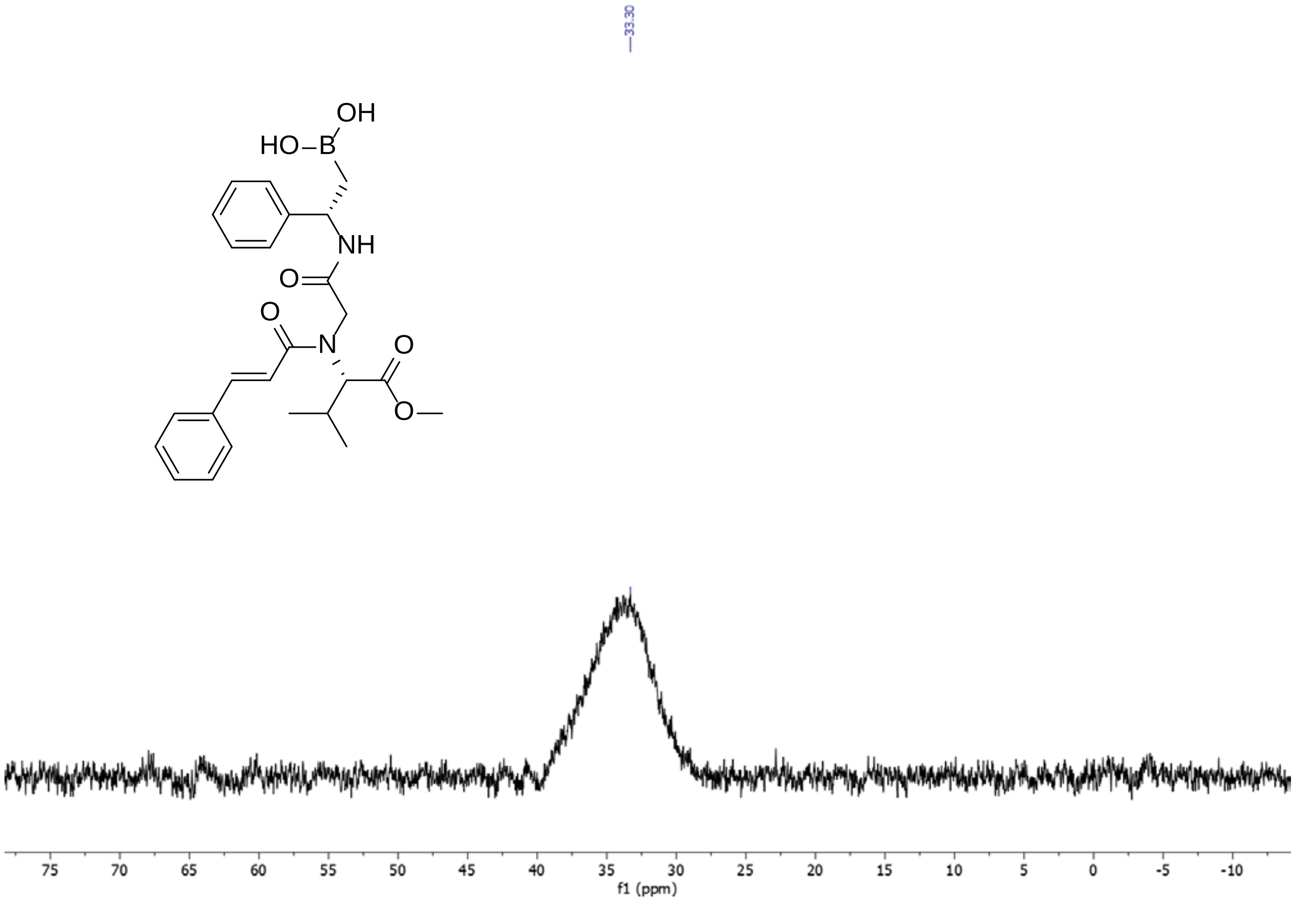

^{1}H NMR (400 MHz, $CDCl_3$) of compound **13**

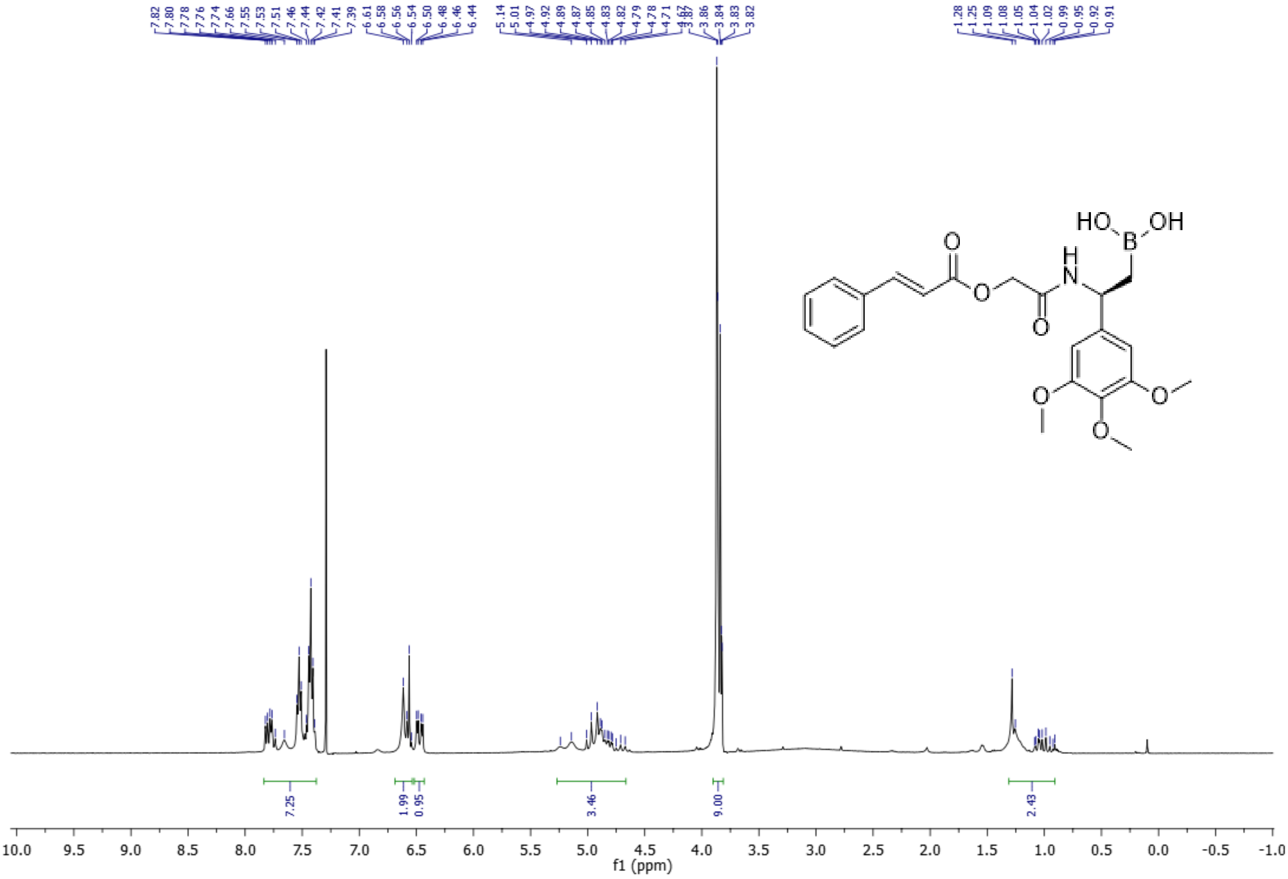

^{13}C NMR (101 MHz, $CDCl_3$) of compound **13**

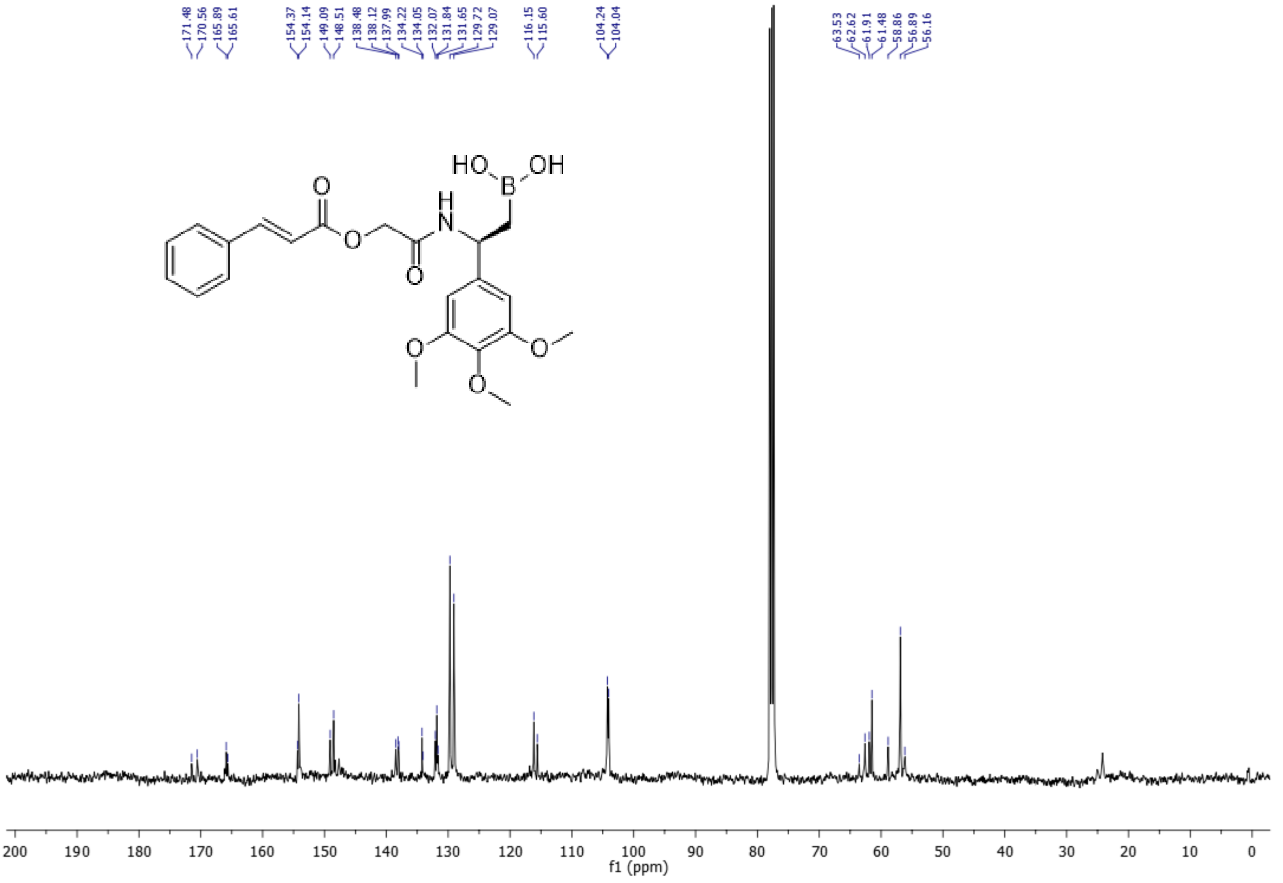

^{11}B NMR (128 MHz, $CDCl_3$) of compound **13**

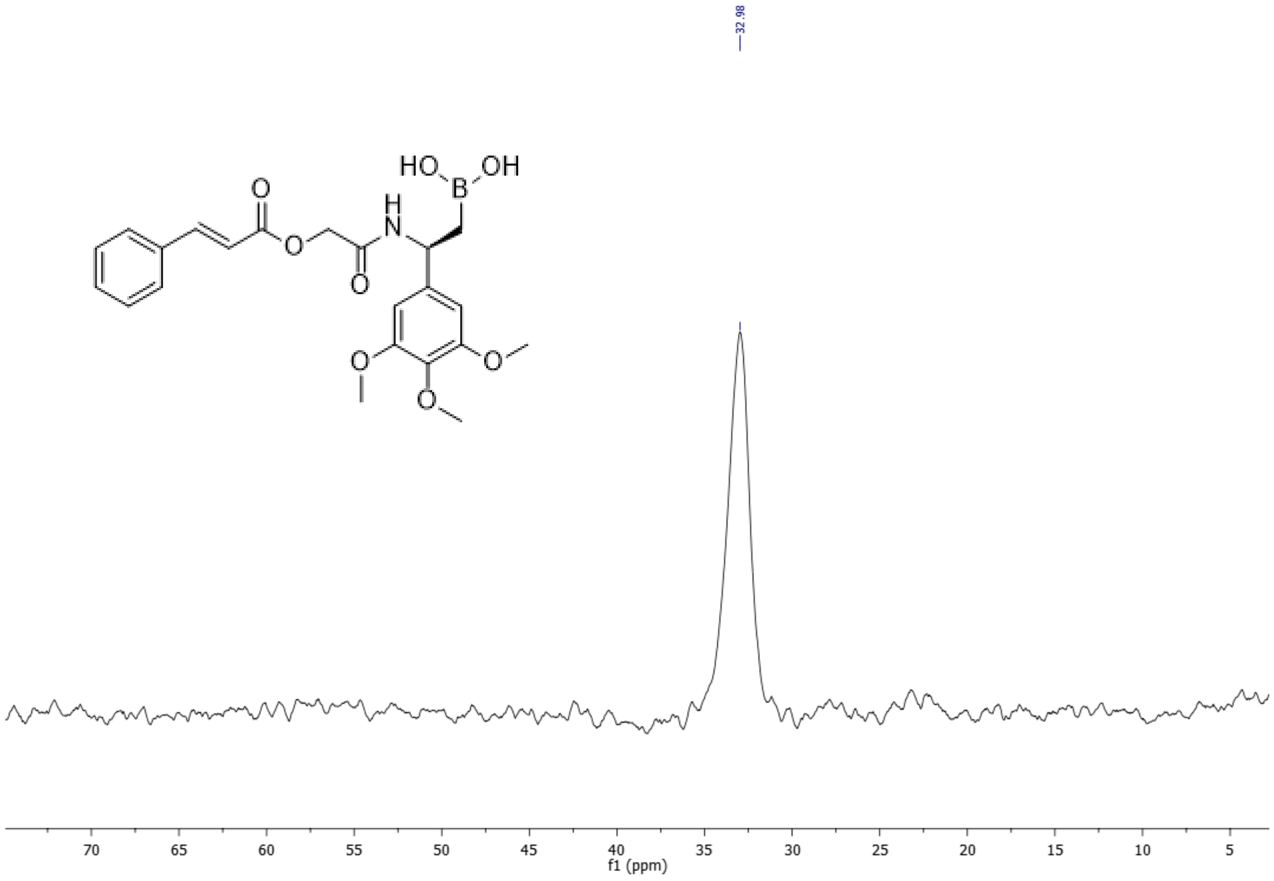

^{1}H NMR (400 MHz, D_2O+3 drops of DMSO d_6) of compound **14**

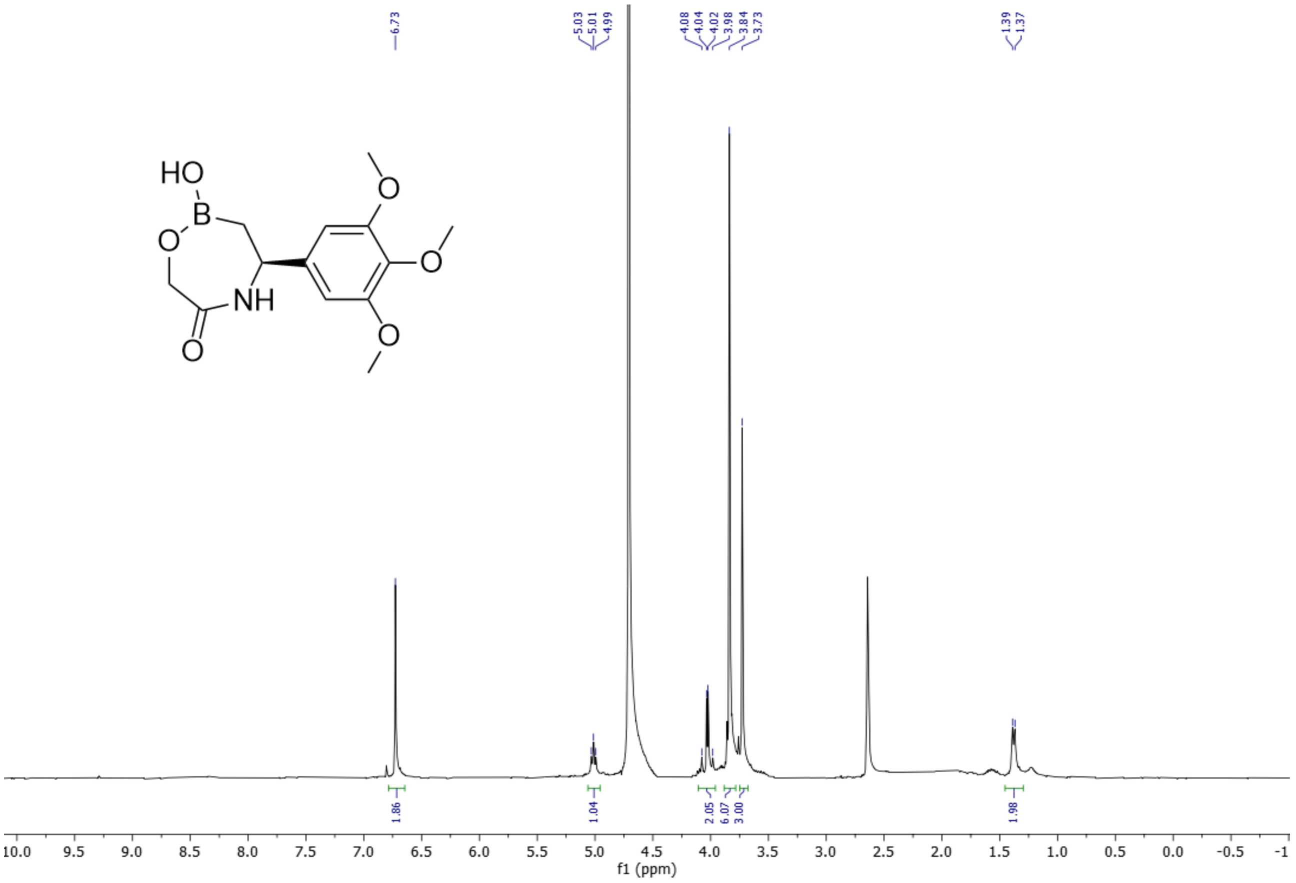

^{13}C NMR (101 MHz, D_2O+3 drops of DMSO d_6) of compound **14**

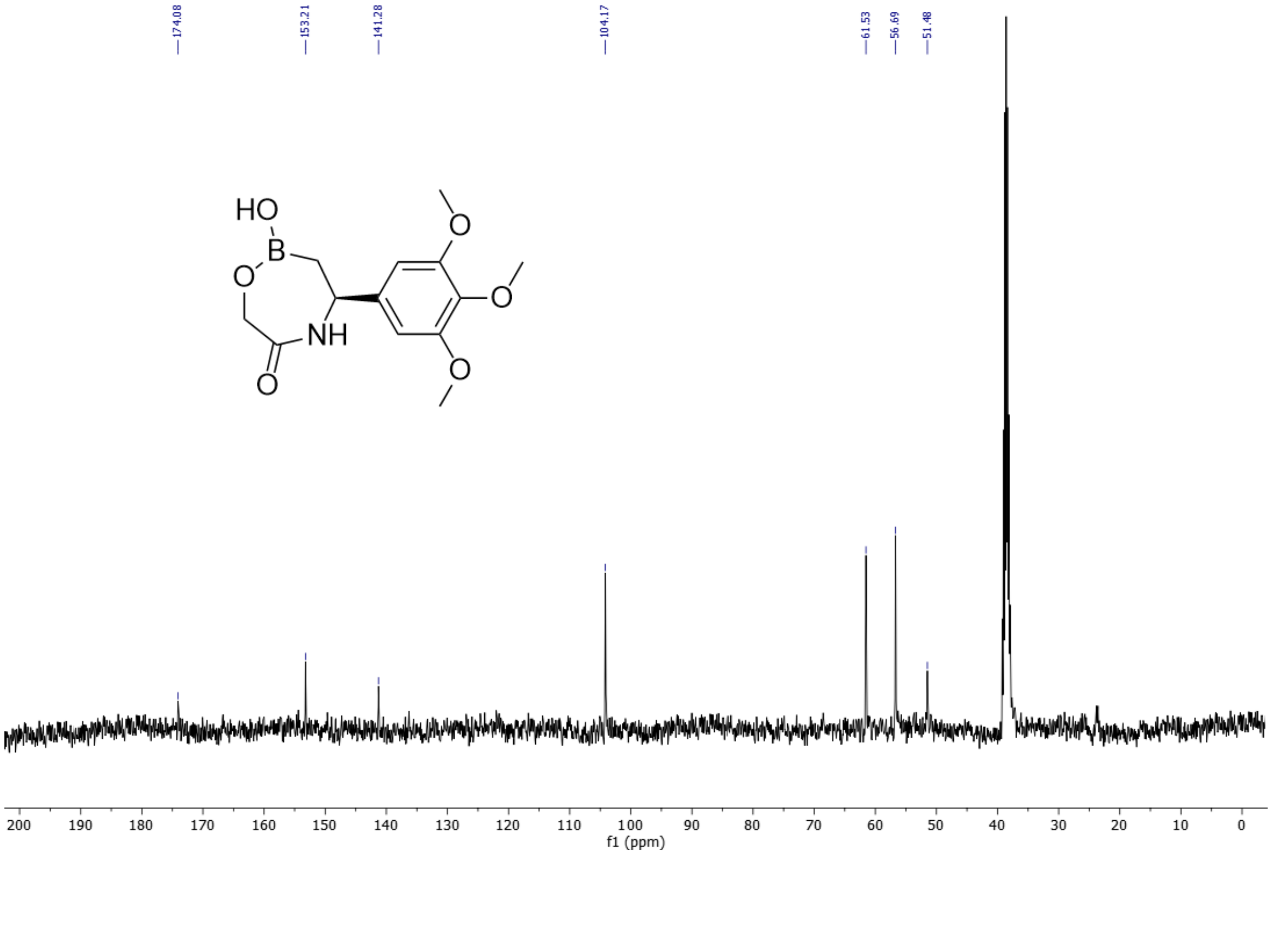

^{11}B NMR (128 MHz, D_2O+3 drops of DMSO d_6) of compound **14**

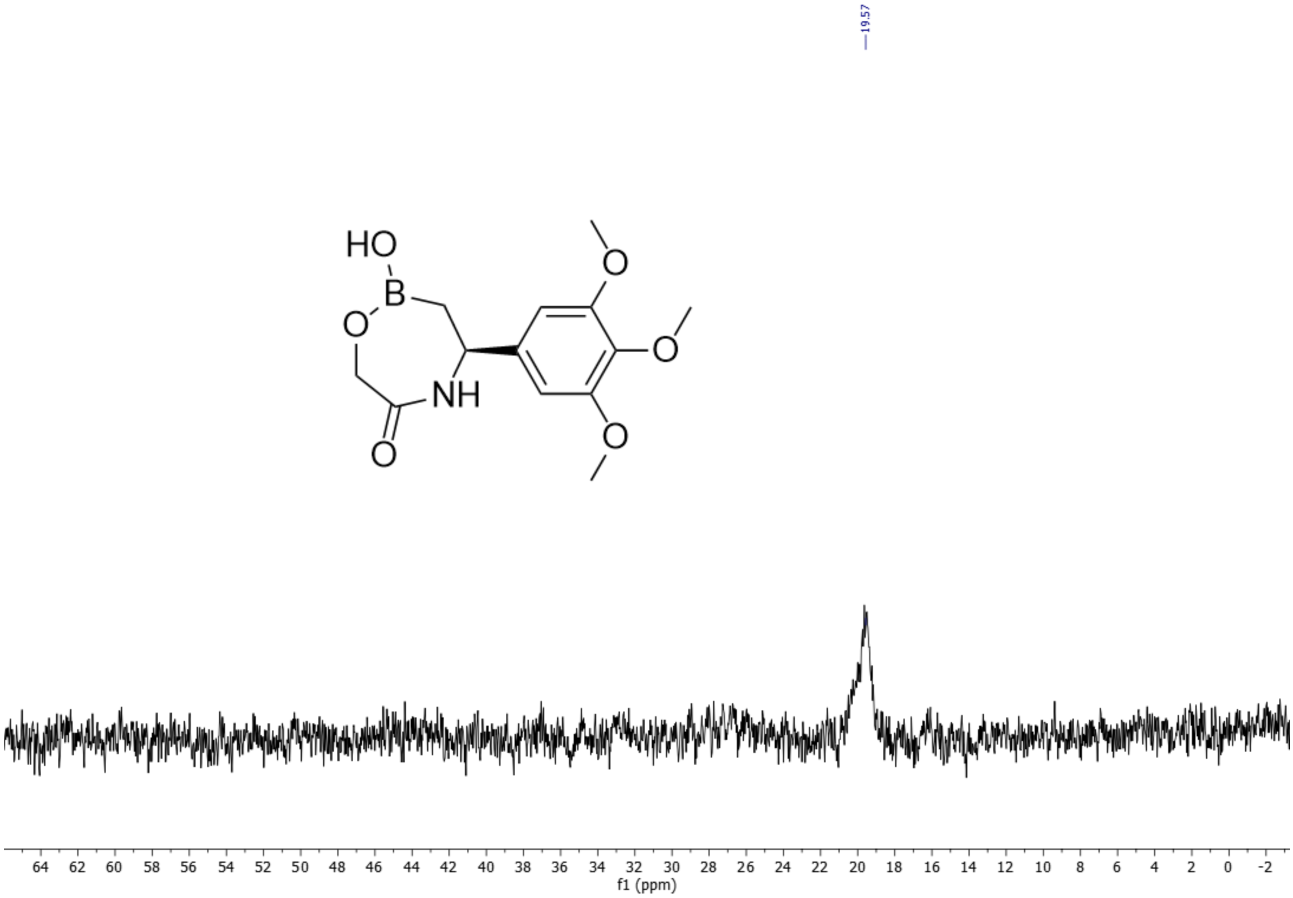

Crystallographic data of compound 12

CCDC 2192101 contains the supplementary crystallographic data for this paper. These data can be obtained free of charge from The Cambridge Crystallographic Data Centre via www.ccdc.cam.ac.uk/structures.

An X-ray quality sample of compound **12** (prism, colourless, transparent, with dimensions ≈ 0.550 x 0.050 x 0.050 mm) was mounted on top of a glass fibre with two component epoxy glue (Figure S1).

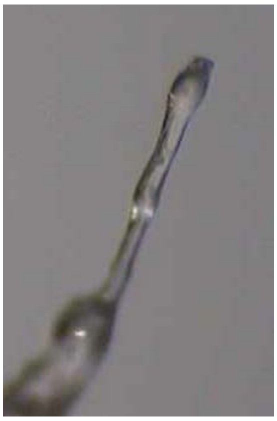

Figure S1. Sample of compound **12** employed for the X-ray analysis.

A 99 % complete data collection (3382 independent data, of which 1274 had $I > 2\sigma(I)$) was carried out up to a maximum resolution of 1 Å with a Bruker AXS three-circle diffractometer equipped with a normal focus sealed tube and an APEX-II CCD area detector. Diffraction intensities were recorded at room temperature with a nominal power of 50 kV x 30 mA of the X-ray generator, using graphite-monochromated Mo Kα radiation (λ = 0.71073 Å). A higher resolution could not be achieved due to the very low scattering power of the title compound. Data integration and reduction was carried out with the SAINT+ suite of programs;[1] diffracted intensities were corrected by absorption and beam anisotropy with SADABS.[1] The structure was solved with direct methods by shelxs and refined by nonlinear least squares with shelxl.[2,3]

The compound is chiral and crystallizes in the chiral acentric polar Sohncke $P2_1$ space group with two molecules in cell and one **12** molecule in the asymmetric unit. The unit cell parameters are a = 16.308(5) Å, b = 5.259(2) Å, c = 18.867(7) Å, β = 94.601(18) deg, V = 1612.9(10) Å^3, as estimated from 520 intense reflections among 5.0 and 23.6 deg in 2θ.

Disordered unresolved solvent is likely present in a large void (485.3 Å^3, 30.1 % of the cell volume); it was modelled indirectly, by applying a back-Fourier transform of the continuous density in the solvent-accessible region of the difference map (PLATON/SQUEEZE procedure).[4]

The final least-squares model employed 307 variable parameters, in conjunction with 117 restraints. The resolution cutoff results in poor precision of geometrical bond lengths and angles, as well as in a low accuracy of thermal motion parameters. The restraints were necessary to avoid instabilities of the least-squares procedure. Distances, angles and thermal motion parameters were restrained in phenyls and in the terminal $B(OH)_2$ substituent (DFIX, DANG, SIMU). A FLAT restraint was also imposed on both phenyl rings. Finally, The agreement factors were as low as R1(F) = 0.1133 for 1274 $F_o > 4\sigma(F_o)$, 0.2271 for all the 3382 independent data; $wR(F^2)$=0.3577 for all the independent

data; goodness-of-fit: 0.967, restrained: 0.973. The maximum and minimum residual Fourier peaks were $\Delta\rho_{MAX/MIN}$ = +0.19/ –0.22 e/Å^3.

Despite the poor resolution, the quality of the present experiment is high enough to unequivocally assess chemical connectivity, conformation and relative configuration of chiral centres of the title compound. No anomalous scatterers are present, thus the absolute structure cannot be determined reliably from the diffraction data alone. As the synthesis procedure ensures that the C11 asymmetric carbon (Figure S2) has S configuration, it is also possible to secure the absolute configuration of **12** as (S,S).

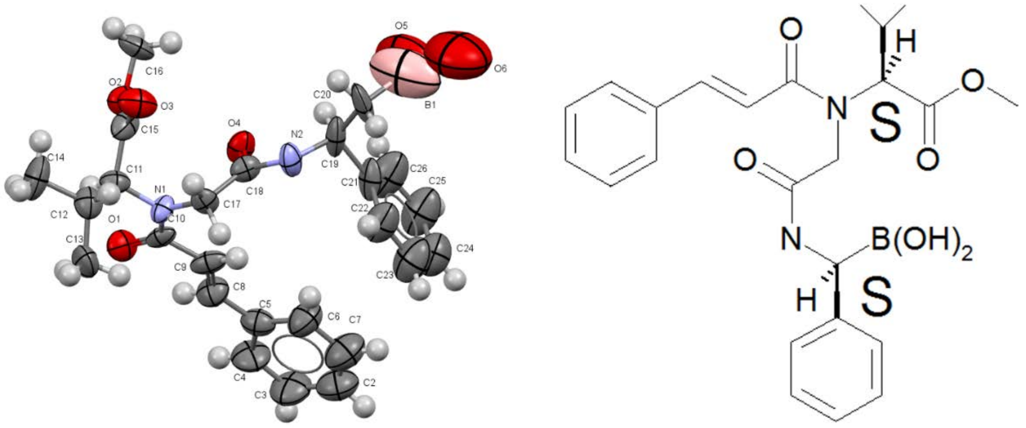

Figure S2. Left: Asymmetric unit of compound **12**, with the atom-numbering scheme. Thermal ellipsoids at RT were drawn at the 25 % probability level. Atoms are represented with the usual CPK colour code (C: grey; H: white, O: red, B: pink; N: blue). Right: Molecular structure of **12**, with CIP configurational descriptors at C11 and C19 asymmetric carbon atoms highlighted.

REFERENCES

(1) Bruker AXS Inc. SAINT and SADABS. *APEX II*. 2014.

(2) Sheldrick, G. M. Crystal Structure Refinement with SHELXL. *Acta Crystallogr. Sect. C Struct. Chem.* **2015**, *71* (Md), 3–8.

(3) Sheldrick, G. M. A Short History of SHELX. *Acta Crystallographica Section A: Foundations of Crystallography*. 2008.

(4) Spek, A. L. PLATON SQUEEZE: A Tool for the Calculation of the Disordered Solvent Contribution to the Calculated Structure Factors. *Acta Crystallogr. Sect. C Struct. Chem.* **2015**, *71*, 9–18.
